# Efficacy and safety of Treamid in the rehabilitation of patients after COVID-19 pneumonia: a phase 2, randomized, double-blind, placebo-controlled trial

**DOI:** 10.1186/s12967-022-03660-9

**Published:** 2022-11-03

**Authors:** Evgeny Bazdyrev, Maria Panova, Maria Brachs, Elena Smolyarchuk, Daria Tsygankova, Liudmila Gofman, Yana Abdyusheva, Fedor Novikov

**Affiliations:** 1 Kemerovo Cardiology Center: FGBNU Naucno-issledovatel’skij institut kompleksnyh problem serdecno-sosudistyh zabolevanij, 6, Sosnoviy Blvd., Kemerovo, 650002 Russia; 2 PHARMENTERPRISES LLC, Skolkovo Innovation Center, Bolshoi Blvd., 42 (1), Moscow, 143026 Russia; 3 Treamid Therapeutics GmbH, c/o CoLaborator (Bayer), Building S141, Muellerstr. 178, 13353 Berlin, Germany; 4 I.M. Sechenov First Moscow State Medical University (Sechenov University), Trubetskaya ul. 8, Moscow, 119991 Russia; 5 Kemerovo Regional Clinical Hospital Named After S.V. Belyaev, 22, Oktyabskiy pr., Kemerovo, 650066 Russia

**Keywords:** COVID, SARS-CoV-2, Post-COVID rehabilitation, Lung function, Lung fibrosis, Treamid, NCT04527354

## Abstract

**Background:**

Many patients who recovered from COVID are still suffering from pulmonary dysfunction that can be persistent even for months after infection. Therefore, treatment to prevent irreversible impairment of lung function is needed. Treamid (bisamide derivative of dicarboxylic acid, BDDA) was shown to have anti-inflammatory and antifibrotic effects in animal models of pulmonary fibrosis. This study was designed to assess the safety, tolerability, and efficacy of Treamid in the rehabilitation of patients after COVID pneumonia. The aim was to establish whether Treamid could be effective in ameliorating post-COVID sequelae.

**Methods:**

The phase 2, randomized, double-blind, placebo-controlled clinical trial was done at 8 medical centers in Russia. Patients with a diagnosis of COVID in the past medical history (with the first symptoms of COVID appear no earlier than 2 months before screening) and having fibrotic changes in the lungs, decreased lung function (percentage of predicted FVC and/or DLCO < 80%), and moderate or severe dyspnea according to mMRC scale were enrolled and randomly assigned in a 1:1 ratio (stratified by the initial degree of lung damage, age, and concomitant chronic diseases) by use of interactive responsive technology to peroral administration of Treamid 50 mg or placebo once a day for 4 weeks. The primary outcome was the proportion of patients who achieved clinically significant improvement in FVC and/or DLCO (defined as a relative increase in FVC of ≥ 10% or a relative increase in FVC in the range of ≥ 5 to < 10% plus a relative increase in DLCO of ≥ 15%) at week 4 compared with baseline. Secondary endpoints included changes from baseline in dyspnea scoring evaluated by the modified Borg and mMRC scales, pulmonary function (FEV_1_, FVC, FEV_1_/FVC ratio, DLCO, TLC, FRC), 6-min walk distance, the overall score of the KBILD questionnaire, and the proportion of patients with a reduction in the degree of lung damage assessed by CT scores. This trial was registered on ClinicalTrials.gov (Identifier: NCT04527354). The study was fully funded by PHARMENTERPRISES LLC.

**Results:**

12 out of 29 patients (41%) in Treamid group achieved clinically significant improvement in FVC and/or DLCO compared to 5 out of 30 patients (17%) in placebo group (p = 0.036). There was a significant decrease of dyspnea according to modified Borg scale observed in the Treamid group (− 0.9 ± 0.7 vs. − 0.4 ± 0.8, p = 0.018). No significant differences in the adverse events were noted. Exploratory analysis of the female population indicated superiority of Treamid over placebo by decreasing dyspnea and the extent of lung damage as well as increasing TLC.

**Conclusions:**

4 weeks oral administration of 50 mg Treamid was associated with clinically significant improvement in the post-COVID patients, evident by an increase in FVC and/or DLCO as well as decreasing dyspnea. Treamid was well tolerated and can be safely administered to patients discharged after COVID. Treamid was more effective in women visible by superior improvement of COVID sequalae after 4 weeks treatment. Considering that female gender is a risk factor associated with the development of post-COVID symptoms, Treamid might offer a pharmacological treatment for long-term sequalae after COVID and supports further investigation in future clinical trials in post-COVID patients.

**Supplementary Information:**

The online version contains supplementary material available at 10.1186/s12967-022-03660-9.

## Introduction

Since it was first reported in December 2019, coronavirus disease 2019 (COVID-19, COVID) caused by severe acute respiratory syndrome coronavirus-2 (SARS-CoV-2) is the third and largest coronavirus outbreak with over 483 million confirmed cases and 6.1 million deaths as of April 2022 [1]. It was shown previously that some viral infections can cause the so-called “post-viral syndrome”. Prior human coronavirus diseases (MERS, SARS) were characterized by persisting symptoms of fatigue, myalgia, and psychiatric impairments for up to 4 years after recovery [2, 3]. Thus, long-term consequences of COVID are recognized as a focus of attention [4–6].

Even though the development of post-COVID complications is not fully understood and it is uncertain how clinical manifestations will evolve over time, several studies reported initial data on post-COVID sequelae. Symptoms continuing for more than six months are now described as “post-COVID syndrome” or “long COVID” [7]. Despite almost all organs are at high risk of being affected by SARS-CoV-2 [8, 9] the respiratory system is the main target of the virus and therefore, virus-related side effects are most likely impacting lung function. Thus, many patients who have recovered from COVID are still suffering from cough and shortness of breath [10], and severe cases can even develop pulmonary fibrosis, which will subsequently lead to reduced lung function. Studies investigating long-term follow-ups in post-COVID patients showed that even in patients with mild disease progression one-third was suffering from lasting sequelae after recovery of the acute phase. In hospitalized patients, 76% of survivors were still presenting with at least one symptom after 6 months [11].

Reduction in the diffusion capacity of the lung for carbon monoxide (DLCO) was shown to be the most common consequence of the respiratory system after SARS-CoV-2 infection, persisting 6 months post-recovery in up to 50% of COVID survivors [12]. Moreover, in a prospective cohort study on 238 patients 1 year after hospitalization for COVID pneumonia, DLCO < 80% was observed in 96 patients (49%) and DLCO < 60% was reported in 20 patients (10%) [13]. This respiratory impairment may be observed as a consequence of diffuse alveolar and capillary damage, hyaline membrane formation, alveolar septal fibrous proliferation, and pulmonary consolidation. However, the exact mechanisms underlying the development of chronic lung sequelae after acute SARS-CoV-2 infection remains elusive. Early data of COVID patients showed a dysregulated inflammatory response during SARS-CoV-2 infection to be a major cause of severity and death of COVID [14]. This is probably why the use of systemic corticosteroids during hospitalization is associated with decreased long-term symptoms and higher survival rate which might be explained by alleviated inflammation leading to subsequently reduce organ and tissue damages [15]. Dysregulated immune response has also been regarded as one of the driving forces for developing post-COVID syndrome [16, 17]. In elderly, areas of abnormal lung imaging were found to be directly correlated with abnormal lung function tests, indicating that either incomplete tissue recovery or persistent tissue remodeling with fibrosis may cause impaired respiratory function [16].

Treamid (bisamide derivative of dicarboxylic acid, BDDA), a newly developed small molecular compound, showed strong anti-inflammatory and anti-fibrotic action in cellular and in vivo models. In a murine carrageenan air-pouch model, Treamid significantly reduced the influx of neutrophils and macrophages. Importantly, Treamid demonstrated beneficial effects in mouse models of lung injury, especially against bleomycin-induced lung fibrosis. Prophylactic intragastrical administration of Treamid (10 mg/kg) was shown to significantly decrease lung weight and Ashcroft score and reduce deposition of type 1 collagen compared to untreated control mice. Therapeutic administration of 10 and 25 mg/kg Treamid starting at day 8, resulted in decreased lung weight, reduced connective tissue area and parenchyma destruction, and normalized the microvascular architecture, further supporting anti-inflammatory and regenerative activity (unpublished data). Additionally, therapeutic administration of 10 mg/kg Treamid starting at day 14 lead to reduced lung inflammation and fibrosis as indicated by decreased levels of lymphocytes and macrophages infiltration, IL-13 concentration, total collagen, collagen type 1, hydroxyproline, and fibronectin in lung homogenates compared to untreated control mice [18]. The promising activity of Treamid in models of lung injury supported its investigation in patients with post-COVID syndrome. We hypothesized that Treamid would reduce the protracted inflammatory response after COVID-associated pneumonia, attenuate the residual lung inflammation and improve the diffusion capacity of the lungs. These effects were expected to boost lung tissue regeneration and exercise tolerance recovery.

This phase 2 clinical study was designed to assess the efficacy of Treamid and its tolerability and safety profile in patients discharged after COVID pneumonia.

## Methods

The trial protocol, protocol amendments and statistical analysis plan are available in Additional file 1. The study was registered on ClinicalTrials.gov (Identifier: NCT04527354).

### Study design and patients

This phase 2, randomized, double-blind, placebo-controlled trial was done at eight Russian clinical centers from September 01, 2020 to February 2, 2021. The phase II exploratory clinical trial has been developed in early 2020, at the start of the COVID-19 epidemic. Thus, the study was designed prior to reliable data on pathogenesis, severity and outcome of post-COVID syndrome. Therefore, for ethical reasons, we conducted the study using a minimum possible number of patients. We assumed that the results of the study would yield positive data and justify further investigation of the drug in patients with post-COVID syndrome. The clinical study protocol and the informed consent form were approved by independent ethics committee and the study was done in accordance with the ethical principles of Good Clinical Practice and the Declaration of Helsinki.

Eligible patients were aged 18–75 years and had received a diagnosis of COVID in the past medical history (confirmed by positive qualitative analysis of SARS-CoV-2 RNA by PCR method) with the first symptoms of COVID appear no earlier than 2 months before screening and having confirmed negative COVID test at screening. Both hospitalized (for up to 1 month, only patients with noninvasive oxygen supply) and non-hospitalized patients were included. Patients presenting at screening with fibrotic changes in the lungs (chest CT score of 1 or more and at least 5% of lung tissue involvement), decreased lung function (percentage of predicted FVC and/or DLCO < 80%), and mMRC dyspnea score of 2 (moderate) or 3 (severe)^1^ were included and randomized. Patients with known underlying health conditions of the lung or other organs leading to fibrosis were excluded from the study. All patients provided written informed consent before study participation. Patients were advised to continue the standard program of medical rehabilitation in an outpatient clinic or at home.

### Randomization and masking

The study consisted of a screening period of up to 4 weeks, a 4-week treatment period, and a follow-up period of up to 2 weeks. Eligible patients were randomly assigned in a 1:1 ratio (stratified by risk factors (age ≥ 60 years and/or presence of concomitant chronic diseases) and the initial degree of lung damage according to CT score) to receive either Treamid or placebo by use of a block randomization scheme generated by IWRS system. Patients, investigators, and staff undertaking laboratory analyses were masked to group allocation.

### Procedures

The survey program included collection of demographic data, medical history and concomitant therapy, physical examination, measurement of height, body weight and body mass index (BMI), assessment of vital signs, and blood oxygen saturation (SpO_2_), electrocardiography (ECG). SARS-CoV-2 RNA was assessed by real-time reverse transcription-PCR, two negative results were required 24 h apart.

Patients were randomly assigned to receive 50 mg Treamid or placebo once a day for 28 days (4 weeks). Treamid’s daily dose was selected based on preclinical PK/PD model and Phase 1 PK data (see Additional file 1: Appendix S17). Computed tomographic (CT) quantitative evaluation for lung injury was conducted according to the standard protocol and was evaluated in each center by physicians blinded to the patient data and treatment allocation. Spirometry and body plethysmography was performed according to the American Thoracic Society (ATS) guidelines [19]. Spirometry tests were used for forced vital capacity (FVC), forced expiratory volume in 1 s (FEV_1_), FEV_1_/FVC ratio evaluation. Total lung capacity (TLC) and functional residual lung capacity (FRC) were determined during the body plethysmography procedure. The diffusing capacity of the lungs for carbon monoxide (DLCO) evaluation was carried out by the method of a single breath-hold and corrected for hemoglobin level. All measurements of the pulmonary function tests were expressed as absolute values and percentage of predicted normal values (% predicted). The percentage of predicted normal values were calculated automatically based on age, sex, and height according to reference equations (see Additional file 2). The 6-min walk test (6MWT) was conducted according to the ATS guidelines [20]. The intensity of dyspnea was evaluated using the modified Medical Research Council (mMRC) [21] scale and modified Borg scale (after 6MWT) to determine breathlessness during exercise. The King’s Brief Interstitial Lung Disease (KBILD) questionnaire was used to estimate health-related quality of life [22, 23]. Baseline levels of all measurements, except for 6MWT, mMRC scale and KBILD questionnaire (all three recorded at randomization), were assessed at screening. All measurements were repeated after 2 and 4 weeks of treatment (CT only after 4 weeks, spirometry and mMRC additionally after 1 and 3 weeks).

The safety of Treamid was assessed via adverse event reporting, vital signs, focused physical examination, clinical laboratory testing (hematology, serum chemistry, and urinalysis), and a 12-lead electrocardiogram (ECG). Final safety assessments were done at week 6, 2 weeks after final dosing.

### Outcomes

The primary outcome was the proportion of patients who achieved clinically significant improvement in FVC and/or DLCO at week 4 compared with baseline. Clinically significant changes were defined as relative increase in FVC of ≥ 10% or relative increase in FVC in the range of ≥ 5 to < 10% and a concomitant relative increase in DLCO of ≥ 15% [24, 25]. Secondary efficacy outcomes included changes from baseline in: pulmonary function measured by spirometry (FEV_1_, FVC and FEV_1_/FVC ratio) and dyspnea evaluated by the mMRC scale at weeks 1, 2, 3 and 4, 6-min walk distance (6MWD), dyspnea during exercise evaluated by modified Borg scale, pulmonary function measured by body plethysmography (DLCO, TLC, FRC), the overall score of the KBILD questionnaire at weeks 2 and 4, and the proportion of patients with a reduction of the lung damage degree based on the CT scores (for the scores assignment see Additional file 2) at week 4.

Safety data were summarized descriptively. Adverse events were coded with the Medical Dictionary for Regulatory Activities (MedDRA), version 18.0. Treatment-emergent adverse events (after the first dose and within 2 weeks of the last dose), serious adverse events, and adverse events leading to study or treatment discontinuation were examined by frequency, severity, organ systems affected, and relationship to study medication. Adverse events grading was defined by Common Terminology Criteria for Adverse Events (CTCAE), version 4.03.

### Statistical analysis

Due to missing long-term results and general reliable data on post-COVID, only minimum possible number of patients was included. This decision was based on ethical reasons to avoid unnecessary patient exposure to a new investigational drug. Due to limited historical data, the population size was calculated based a single-arm, single-stage A´Hern design. The phase II exploratory clinical trial design was developed in the first half of 2020, at the start of the COVID19 epidemic. Since at that time there was no reliable data on pathogenesis and outcome of the disease, as well as on the epidemiology of the post-COVID syndrome, the study contained minimum possible number of patients. For these reasons the population size was determined according to single-arm, single-stage A’Hern design [26]. Due to limited data on the process of lung function recovery in patients discharged after COVID [27], it was assumed that the probability of achieving a clinically significant recovery of respiratory function (change in FVC and/or DLCO levels) in the placebo group would not exceed 25% and the expected probability of response in the Treamid group was taken as 50%. However, since the probability of spontaneous regress of post-COVID syndrome was not reliably determined at the time of the study design, a placebo group (receiving standard rehabilitation therapy) was included in the study. Taken an alpha level of 0.05 (one side) and a power of 80%, at least 26 patients must be included to confirm the hypothesis whether Treamid is beneficial in post-COVID patients. In addition, the effect of Treamid in comparison to standard rehabilitation therapy was expected to be clinically significant. Considering 13% for potential dropouts, enrolment of 60 patients was planned (30 patients at each group).

Subjects who received at least one dose of the study drug and were randomized in either placebo or Treamid group were included in the intention-to-treat (ITT) population. Subjects who completed the entire course of the study and had no significant violation to the protocol were included in the per-protocol set (PPS) population. Primary outcome was evaluated in the modified ITT (mITT) population^2^ and was supported by the analyses in the PPS population. Secondary outcomes were evaluated in the mITT population only. All patients who received at least one dose of study drug were included in the safety set (SS) population.

Categorical variables were presented as frequencies (n, %), continuous variables were presented as mean (M) ± standard deviation (SD). A χ^2^ test (frequencies > 5) or Fisher’s exact test (frequencies ≤ 5) was performed to compare response rates between groups and for categorical variables of the secondary endpoints. Student’s t-test or Mann–Whitney test was performed for continuous data. Significant tests were two-sided and statistical significance set to 0.05. All statistical analyses were conducted using SAS, version 9.4.

Since female gender was shown as one of the main risk factors for most long-term symptoms after COVID [28] the effectivity of Treamid was evaluated in women (19 patients in Treamid group and 14 in placebo group). Medians and means were calculated to compare changes in women. Statistical significance of differences in medians was assessed using median test and median regression (29) with the p-values calculated with Wild method based on 1,000,000 bootstrap samples [30].

Linear models or median regression models, if the residual normality test was violated, were used for estimating the mean or median changes in modified Borg dyspnea scale, TLC and lung damage adjusted for baseline values as covariates with gender and treatment group as fixed factors. In each case, a choice was made from eight models that considered different variants of interaction terms (see Additional file 2: Appendixes S1–S4). These eight models were compared in terms of Akaike information criterion (AIC) (i.e., models with the lower AIC values offer best fits) as implemented in AICcmodavg package (version 2.3-1) in R [31]. Of the selected models (difference in AIC values less than one), the one with the highest proportion of variance explained (R^2^ or pseudo-R^2^) was chosen for the calculation of adjusted mean or median changes for women of Treamid and placebo groups with corresponding 95% confidence intervals using emmeans package (version 1.7.0) in R [32]. The corresponding pairwise comparisons of adjusted changes in modified Borg dyspnea score, TLC and lung damage for women of Treamid and placebo groups were made without p-value adjustment. Post-hoc statistical analysis was performed using the free statistics software R (version 4.1.0).

### Role of the funding source

The study was fully funded by PHARMENTERPRISES LLC. The funder had no role in study design, data collection, or writing of the report. The funder participated in writing the article. All authors had access to the raw data and study results. Authors are responsible for the integrity of the data and the accuracy of the data analysis.

## Results

### Study enrollment and patient characteristics

**Fig. 1:**
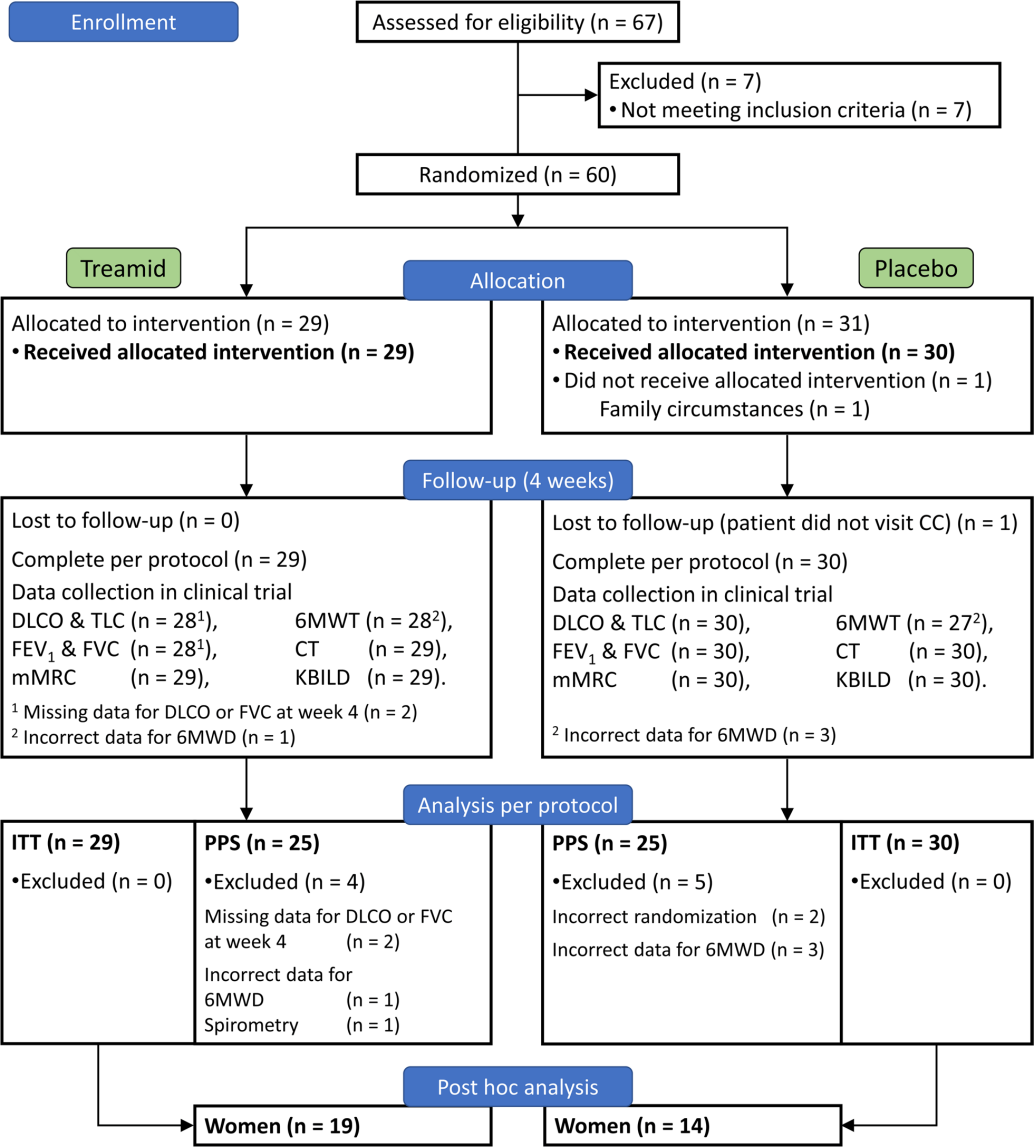
Flowchart of the study: a total of 67 subjects were screened and 60 subjects were randomized in 1:1 fashion to receive peroral Treamid or placebo. *CC* clinical center. For other abbreviations see description for Table 1

Overall, 67 Caucasian patients were enrolled from 8 Russian clinical centers and 60 of them were randomly assigned to receive Treamid (n = 29) or placebo (n = 31) (Fig. 1). The modified intention-to-treat (mITT) population contained 29 patients in the Treamid group and 30 in the placebo group (one patient in the placebo group withdrew the consent due to family circumstances). Per protocol set (PPS) consisted of 25 patients in each group. 4 patients in Treamid group were excluded from PPS due to missing or incorrect data and 5 patients in placebo group were excluded from PPS due to incorrect randomization or missing data (Fig. 1, a complete list of protocol deviations and reasons for patients’ exclusion are provided in Additional file 2). The safety population (SS) included 60 patients (29 in Treamid and 31 in placebo) who received at least one dose of study drug.

The study population (mITT) consisted of 56% women, mean age was 55 ± 11 years, mean level of pulmonary parenchyma damage was 28.7 ± 20.9% and mean DLCO at baseline was 73.8 ± 14.8% of predicted (Table 1, for baseline characteristics of PPS see Additional file 2: Table S1). No significant differences in baseline characteristics between the two groups were observed (Table 1).

**Table 1:** Demographics and baseline clinical characteristics of mITT population by study cohort

Parameter	Treamid (n = 29)	Placebo (n = 30)	p-value^a^	Overall (n = 59)
Age (year)	54 ± 10	56 ± 12	0.526 (t)	55 ± 11
Male, n (%)	10 (34.5)	16 (53.3)	0.145 (c)	26 (44.1)
BMI (kg/m^2^)	28.5 ± 4.9	27.8 ± 4.5	0.584 (t)	28.1 ± 4.7
6MWD (m)	423.6 ± 80.2	418.9 ± 96.7	0.844 (t)	421.3 ± 87.9
6MWD (%, predicted)^1^	78.3 ± 14.8	76 ± 18.6	0.609 (t)	77.2 ± 16.6
Borg scale (score)	2.2 ± 0.8	1.8 ± 1.1	0.183 (t)	2.0 ± 1.0
mMRC scale (score)	2.0 ± 0.0	2.1 ± 0.3	0.168 (u)	2.0 ± 0.2
KBILD questionnaire (score)	50.2 ± 4.5	52.8 ± 6.7	0.089 (t)	51.5 ± 5.8
Pulmonary function				
FEV1 (l)	2.7 ± 0.7	2.9 ± 0.8	0.235 (t)	2.8 ± 0.7
FVC (l)	3.3 ± 0.8	3.5 ± 0.9	0.330 (t)	3.4 ± 0.8
FEV1/FVC, %	80.1 ± 11.0	82.1 ± 9.8	0.466 (t)	81.1 ± 10.3
TLC (l)	5.6 ± 1.4	6.0 ± 1.2	0.223 (t)	5.8 ± 1.3
FRC (l)	3.1 ± 0.9	3.4 ± 1.1	0.481 (u)	3.3 ± 1.0
DLCO (mmol/min/kPa)	19.9 ± 5.1	20.3 ± 5.5	0.822 (t)	20.1 ± 5.2
Pulmonary function (%, predicted)				
FEV1	87.5 ± 17.8	91.1 ± 17.0	0.439 (t)	89.3 ± 17.3
FVC	86.0 ± 14.0	87.4 ± 16.3	0.932 (u)	86.7 ± 15.1
TLC	96.6 ± 13.8	98.5 ± 14.9	0.596 (u)	97.6 ± 14.2
FRC	100.8 ± 21.9	108.6 ± 34.7	0.601 (u)	104.8 ± 29.1
DLCO	73.9 ± 15.7	73.7 ± 14.1	0.559 (u)	73.8 ± 14.8
Lung damage, %	27.9 ± 20.7	29.6 ± 21.5	0.757 (t)	28.7 ± 20.9
CT score				
1	17 (58.6)	15 (50.0)	0.506 (c)	32 (54.2)
2	9 (31.0)	12 (40.0)	0.472 (c)	21 (35.6)
3	3 (10.3)	3 (10.0)	1.000 (f)	6 (10.2)

Data are mean ± SD or n (%). Baseline defined as the mean assessments at screening (or at randomization for 6MWD, modified Borg scale, mMRC scale and KBILD)

*6MWD* distance walked in 6 min walk test, *BMI* body mass index, *CT* computed tomography, *DLCO* diffusing capacity of the lungs for carbon monoxide, adjusted for blood hemoglobin concentration; *FEV1* forced expiratory volume in 1 s, *FRC* functional residual capacity, *FVC* forced vital capacity; *K-BILD* King’s brief interstitial lung disease questionnaire, *mMRC* modified Medical Research Council dyspnea scale; *TLC* total lung capacity

^1^Calculated according the formula 7.57 × Height − 5.02 × Age − 1.76 × mass − 309 (for men) and 2.11 × Height − 5.78 × Age − 2.29 × mass + 667 (for women) [33]

^a^Test is put in brackets: c—Chi-squared test, f—Fisher's exact test, t—Student's t-test, u—Mann–Whitney test

### Efficacy outcomes

**Fig. 2:**
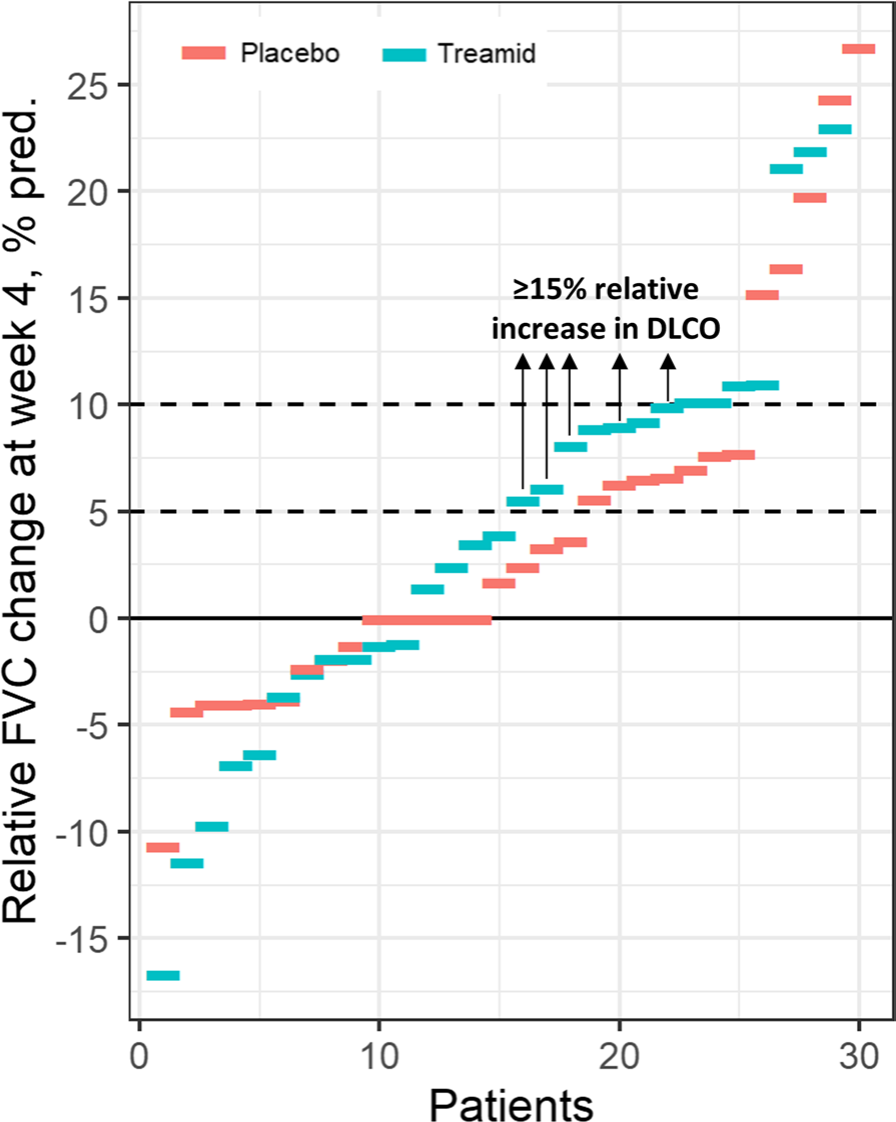
Relative FVC increase in Treamid and placebo groups of mITT population, patients in Treamid group with 5–10% relative increase in FVC along with clinically significant relative increase in DLCO (≥15%) are marked with arrows

After 4-weeks treatment, clinically significant improvement in the primary endpoint was observed in the Treamid group compared to placebo in mITT population (41% vs. 17%, p = 0.036, see Additional files. Table S5). However, the occurrence of clinically significant events was different between Treamid and placebo groups. While the frequency of a ≥ 10% relative improvement in FVC was quite similar between treatment groups, the frequency of a clinically significant improvement in DLCO was considerably higher in Treamid compared to placebo group (34% vs. 13%, p = 0.056). Patients with 5–10% relative increase in FVC and simultaneous relative increase in DLCO of ≥ 15% were found only in Treamid group (marked with arrows in Fig. 2).

Similar results were reported for PPS population: 11 out of 25 patients (44%) in Treamid group and 4 out of 25 patients (16%) in placebo group achieved clinical response (p = 0.031).

Changes in analyzed parameters from baseline to week 4 in responders and non-responders is provided in Additional file 2: Appendix S15.

Stratification of the study population by baseline DLCO (high/normal DLCO ≥ 80% and low DLCO < 80%, 23 patients in Treamid group and 25 patients in placebo group) revealed significant differences in response rates. In patients with low DLCO Treamid treatment led to an improvement in 39% patients compared to 12% patients in placebo group (p = 0.030, Additional file 2: Table S2). No significant difference in response rate was visible in the subgroup of patients with a normal DLCO range at baseline.

In terms of secondary endpoints, Treamid showed beneficial effects over placebo after 4-weeks by decreasing dyspnea after exercise according to Borg scale (− 0.9 ± 0.7 vs. − 0.4 ± 0.8, p = 0.018 (Table 2). However, there were no significant differences in the predicted 6MWD between groups (21.3 ± 23.1% predicted vs. 19.7 ± 16.5% predicted, p = 0.720).

**Table 2:** Change from baseline to weeks 2 and 4 of parameters in mITT population (М ± SD or n (%))

Parameter	Change from baseline to week 2		p-value^a^	Change from baseline to week 4		p-value^a^
	Treamid	Placebo		Treamid	Placebo	
6MWD (m)	39.8 ± 54.2	38.0 ± 40.7	0.98 (u)	78.6 ± 75.2	71.8 ± 50.2	0.960 (u)
6MWD (%, predicted)^a^	10.6 ± 14.0	11.0 ± 13.5	0.822 (u)	21.3 ± 23.1	19.7 ± 16.5	0.720 (u)
**Borg scale (score)**	− 0.5 ± 0.9	− 0.4 ± 0.8	0.411 (u)	− 0.9 ± 0.7	− 0.4 ± 0.8	**0.018 (u)**
mMRC scale (score)	− 0.6 ± 0.5	− 0.6 ± 0.5	0.717 (u)	− 1.1 ± 0.5	− 1.1 ± 0.6	0.683 (u)
KBILD questionnaire (total score)	7.4 ± 5.2	5.7 ± 3.9	0.585 (u)	13.8 ± 7.1	11.7 ± 10.0	0.117 (u)
Breathlessness and activities	9.7 ± 8.7	8.7 ± 8.1	0.638 (u)	17.4 ± 9.1	16.5 ± 13.4	0.383 (u)
Chest symptoms	13.3 ± 11.7	10.7 ± 9.5	0.471 (u)	22.1 ± 16.7	18.3 ± 14.3	0.134 (u)
Psychological symptoms	8.8 ± 7.3	6.0 ± 7.6	0.387 (u)	16.7 ± 9.7	13.1 ± 13.2	0.145 (u)
Pulmonary function						
FEV1 (l)	0.1 ± 0.2	0.1 ± 0.2	0.617 (u)	0.1 ± 0.3	0.1 ± 0.3	0.678 (u)
FVC (l)	0.1 ± 0.3	0.2 ± 0.4	0.755 (u)	0.1 ± 0.3	0.1 ± 0.3	0.725 (u)
FEV1/FVC, %	0.6 ± 6.8	− 1.5 ± 5.9	0.466 (u)	2.3 ± 13.8	− 0.9 ± 4.5	0.936 (u)
TLC (l)	0.3 ± 0.5	0.1 ± 0.7	0.149 (u)	0.3 ± 0.4	0.3 ± 0.8	0.210 (u)
FRC (l)	0.0 ± 0.4	0.0 ± 0.6	0.431 (u)	0.1 ± 0.5	0.0 ± 0.7	0.604 (u)
DLCO (mmol/min/kPa)	2.0 ± 2.7	1.7 ± 2.8	0.451 (u)	2.0 ± 2.4	1.5 ± 2.4	0.493 (t)
Pulmonary function (%, predicted)^a^						
FEV1	3.7 ± 9.0	4.8 ± 10.4	0.731 (u)	6.1 ± 19.1	3.4 ± 8.7	1.000 (u)
FVC	4.3 ± 9.6	7.0 ± 12.0	0.838 (u)	4.0 ± 9.8	4.4 ± 8.8	0.805 (u)
TLC	5.1 ± 11.3	2.0 ± 11.8	0.171 (u)	6.6 ± 8.9	5.3 ± 12.8	0.255 (u)
FRC	1.2 ± 12.0	− 2.4 ± 15.0	0.216 (u)	2.6 ± 16.4	0.6 ± 16.4	0.421 (u)
DLCO	8.7 ± 11.9	10.7 ± 17.2	0.864 (u)	10.4 ± 11.6	9.9 ± 14.1	0.575 (u)
Lung damage, %				− 14.0 ± 12.0	− 14.3 ± 12.9	0.962 (u)
CT score						
Increase or no change				9 (31)	10 (33)	
Decrease				20 (69)	20 (67)	0.850 (c)

Data are mean ± SD or n (%). For abbreviations see Table 1

^a^Data are given in relation to baseline level. Data in bold indicates significant p-value (< 0.05)

^a^Test is put in brackets: c—Chi-squared test, t—Student’s t-test, u—Mann–Whitney test

There was no difference in clinical response regarding pulmonary function (FEV1, FVC, TLC, FRC, and DLCO) or general dyspnea according to mMRC scale after 4 weeks of therapy between both groups (Table 2). A comparable decrease in lung tissue damage assessed by CT score was visible between the groups, 20 out of 29 (69%) patients in Treamid group compared to 20 out of 30 (67%) patients in placebo group. The health status evaluated by KBILD questionnaire indicates clinical superiority of Treamid, however, without being significant.

## The effectiveness of Treamid in women (exploratory analysis)

### Comparison of mean and median change in women

A recent longitudinal cohort study including more than 2500 adults identified female gender as one of the main risk factors for different long-lasting post-COVID symptoms [28]. Subgroup analysis in women (Table 3, Additional file 2: Table S3) revealed significant decrease in dyspnea according to modified Borg scale in the Treamid group compared to placebo (mean changes: − 1.2 ± 0.5 vs. − 0.5 ± 0.9, p = 0.010; median changes: − 1.0 (− 1.5; − 1.0) vs. − 0.5 (− 1.0; 0.0), p = 0.005), increasing TLC level (mean changes: 8.1 ± 10.5 vs. − 0.9 ± 5.3, p = 0.004; median changes: 7.6 (− 0.7; 13.7) vs. − 1.3 (− 5.1; 1.7), p = 0.035), as well as decreasing lung damage (mean changes: − 17.1 ± 11.7 vs. − 10.5 ± 12.6, p = 0.073; median changes: − 15.0 (− 25.0; − 6.5) vs. − 5.0 (− 24.0; 0.0), p = 0.039).

**Table 3:** Baseline values and corresponding changes after 4-week treatment period in women

Parameter	Baseline values			Change to Week 4			
	Treamid	Placebo	p-value^a^	Treamid	Placebo	Treamid superiority	p-value^a^
Borg scale (score)	n = 19	n = 14		n = 19	n = 14		
	2.2 ± 0.9	2.1 ± 1.1	0.748 (w)	− 1.2 ± 0.5	− 0.5 ± 0.9	0.7	0.010* (u)
	2.0 (2.0; 3.0)	2.0 (1.0; 2.9)		− 1.0 (− 1.5; − 1.0)	− 0.5 (− 1.0; 0.0)	0.5	0.005* (m); 0.208 (mr)
6MWD (% pred.)	n = 19	n = 14		n = 19	n = 14		
	81.3 ± 15.3	80.9 ± 16.2	0.952 (t)	22.4 ± 23.7	15.5 ± 11.0	6.9%	0.815 (u)
	84.6 (76.0; 90.1)	80.4 (74.3; 92.1)		13.4 (6.7; 28.3)	14.3 (7.3; 19.7)	− 0.9%	0.883 (m); 0.993 (mr)
TLC (% pred.)	n = 19	n = 14		n = 18	n = 14		
	94.6 ± 14.1	102.6 ± 16.9	0.117 (w)	8.1 ± 10.5	− 0.9 ± 5.3	9.0%	0.004* (t)
	90.0 (84.8; 105.0)	98.1 (91.5; 105.6)		7.6 (− 0.7; 13.7)	− 1.3 (− 5.1; 1.7)	8.9%	0.035* (m); 0.049* (mr)
FVC (% pred.)	n = 19	n = 14		n = 19	n = 14		
	85.1 ± 13.8	81.0 ± 9.9	0.502 (w)	3.3 ± 9.3	3.6 ± 6.4	− 0.3%	0.901 (t)
	79.0 (75.0; 89.8)	78.0 (75.0; 88.3)		4.7 (− 2.4; 9.2)	1.7 (0.0; 6.2)	3%	0.355 (m); 0.436 (mr)
DLCO (% pred.)	n = 19	n = 14		n = 18	n = 14		
	73.2 ± 17.3	72.6 ± 12.9	0.536 (w)	10.6 ± 12.6	9.5 ± 17.7	1.1%	0.595 (u)
	66.0 (61.6; 77.6)	72.2 (64.9; 78.8)		10.3 (− 0.7; 20.0)	5.5 (− 0.9; 13.7)	4.8%	0.483 (m); 0.437 (mr)
Lung damage (%)	n = 19	n = 14		n = 19	n = 13		
	27.5 ± 21.5	27.4 ± 24.7	0.739 (w)	− 17.1 ± 11.7	− 10.5 ± 12.6	6.6%	0.073 (u)
	25.0 (9.0; 49.0)	24.5 (5.0; 46.2)		− 15.0 (− 25.0; − 6.5)	− 5.0 (− 24.0; 0.0)	10.0%	0.039* (m); 0.073 (mr)

Baseline values and change in parameters for women (М ± SD and median (Q1; Q3))

Data in bold indicates significant p-value (< 0.05) according to at least one of the tests used

^a^Test is put in brackets: m—median test, mr—median regression, t—Student’s t-test, u—Mann–Whitney test

### Comparison of adjusted means

#### Change in modified Borg dyspnea scale

According to the best-fit linear model (init:Group + init:Sex + Group:Sex, R^2^adj. = 53.6%), coefficient of interaction of treatment group and gender was statistically significant (− 0.95, p = 0.004, Table 4). According to least-squares adjusted mean change in dyspnea Borg score Treamid outperformed placebo by 0.6 (p = 0.001, Table 4). Also, according to linear models using as covariates baseline values of lung damage, DLCO or 6MWD instead of initial Borg score, the decrease in dyspnea was more pronounced in more severely affected women of the Treamid group compared to Placebo (see Additional file 1: Appendix S5). Thus, according to least-squares adjusted mean analysis Treamids’ superiority over Placebo by decreasing dyspnea was more pronounce in patients with higher area lung damage or decreased DLCO or 6MWD.

**Table 4:** Summary of the exploratory analysis in women

Predicted parameter	Characteristics of the model			Treamid superiority in women, p-value^b^
	Formular^a^	R^2^ (R^2^adj.), % F-statistic (df), p-value	Coefficient of Group:Sex interaction, p-value	
Dyspnea Borg scale score	init:Group + init:Sex + Group:Sex	57.0 (53.6) F (4,50) = 16.6 p < 0.001***	−0.95 0.004**	0.6 0.001**
TLC, % pred.	init:Group + Group * Sex (median regression)	16.5 (pseudo-R^2^) F (5,52) = 5.59 p < 0.001***	11.37 0.028*	8.6 0.009**
Lung damage, %	init:Group + Group * Sex	22.9 (15.5) F (5,52) = 3.09 p = 0.016*	− 15.50 0.015*	6.4 0.127

Data in bold indicates significant p-value (< 0.05)

^a^“:”—interaction term without main effect, “*”—interaction term with main effect

^b^Calculated Treamid superiority in women adjusted for global mean of baseline values (2.01 for Borg scale, 97.9 for TLC, and 28% for lung damage)

#### Change in TLC

According to the best-fit median regression model (init:Group + Group * Sex, pseudo-R^2^ = 16.5%), coefficient of interaction of treatment group and gender was statistically significant (11.37, p = 0.028) indicating increased effectivity of Treamid in women (Table 4). Difference of adjusted medians revealed 8.6% superiority of Treamid group in women (p = 0.009, Table 4).

#### Change in lung damage

According to the best-fit linear model (init:Group + Group * Sex, R^2^adj. = 15.5%), interaction term of treatment group and gender was statistically significant (− 15.50, p = 0.015, Table 4). According to least-squares adjusted mean change in lung damage, Treamid outperformed placebo by 6.4%, but the effect was not statistically significant (p = 0.127, Table 4). The superiority of Treamid in decreasing lung damage was more pronounced for women with a more severe baseline lung damage (according to least-squares mean differences adjusted to different initial lung damage values, Additional file 2: Appendix S6). Moreover, a tendency of improved lung function was visible in women in Treamid group: a decrease in lung damage was accompanied with an increase in FVC as well as an increase of TLC was accompanied with an increase in FVC. This was not observed in placebo treated women (see Additional file 2: Figure S1).

### Safety analysis

The median dosing time for each patient was 28 days and was consistent with the anticipated amount based on the dosing schedule. Vital signs such as body temperature, heart rate, and blood pressure showed no clinically and statistically significant deviations compared with baseline values over the study period in any treatment group. The frequency of observed adverse events (AE) in the two groups of the SS population is summarized in Table 5. The overall frequency of AEs was similar in both groups: 34.5% (10 patients) in the Treamid group and 29.0% (9 patients) in placebo group (p = 0.650, Table 5). All observed AEs were mild or moderate. 18 mild AEs were observed in 10 patients (34.5%) in Treamid group, and 16 mild AEs were observed in 9 patients (29.0%) in placebo group.

**Table 5:** Summary of the observed adverse events in Treamid and placebo groups of SS population

Adverse events	Treamid group (n = 29)			Placebo group (n = 31)			p-value^a^
AE, n (%) E	10	(34.5%)	21	9	(29.0%)	16	0.650 (c)
Grade 1	10	(34.5%)	18	9	(29.0%)	16	0.650 (c)
Grade 2	2	(6.9%)	3	0	(0.0%)	0	0.229 (f)
Respiratory tract infection	1	(3.4%)	1	0	(0.0%)	0	0.483 (f)
Conjunctivitis	1	(3.4%)	1	0	(0.0%)	0	0.483 (f)
Ulcerative keratitis	1	(3.4%)	1	0	(0.0%)	0	0.483 (f)
Leading to temporary treatment discontinuation	0	(0.0%)	0	1	(3.2%)	2	0.483 (f)
Increased level of AST	0	(0.0%)	0	1	(3.2%)	1	0.483 (f)
Increased level of CPK	0	(0.0%)	0	1	(3.2%)	1	0.483 (f)
Treatment-related AE, n (%) E	1	(3.4%)	2	0	(0.0%)	0	0.483 (f)
Grade 1	1	(3.4%)	2	0	(0.0%)	0	0.483 (f)
Increased level of CPK	1	(3.4%)	1	0	(0.0%)	0	0.483 (f)
Gynecomastia	1	(3.4%)	1	0	(0.0%)	0	0.483 (f)

*AE* adverse event, *AST* aspartate transaminase, *CPK* creatine phosphokinase

^a^Test is put in brackets: c—Chi-squared test, f—Fisher’s exact test

The most frequently reported mild AEs were laboratory abnormalities. 9 AEs (including increased serum level of alanine transaminase, hemoglobin, gamma-glutamyl transferase, creatinine, creatine phosphokinase, urea, hematocrit, and decreased serum level of hemoglobin) were observed in 5 patients (17.2%) in the Treamid group and 12 AEs (including increased serum level of sodium, potassium, alanine transaminase, aspartate transaminase, gamma-glutamyl transferase, creatinine, creatine phosphokinase, urea, decreased serum level of hemoglobin, decreased level of albumin in the urine, and QT prolongation on the ECG) in 7 patients (22.6%) in the placebo group. Additional mild AEs included pharyngitis, pain in the upper abdomen, back pain, headache, dysgeusia, and sleep drunkenness in Treamid group and respiratory tract infection, myodynia, and mitral valve prolapse in placebo group. Two AEs (increased levels of aspartate transaminase and creatine phosphokinase) leading to temporary treatment discontinuation were observed in one patient (3.2%) in placebo group. Two mild adverse events where treatment-related (increased level of creatine phosphokinase and gynecomastia) and were reported in one patient in Treamid group (3.4%).

Most observed AEs were completely resolved or were resolved with sequelae (14 out of 21 AE in Treamid group and 12 out of 16 AE in placebo group). AEs that were ongoing or whose outcomes were unknown at the end of the study were mainly related to laboratory parameters (changes in complete blood count and biochemical test).

## Discussion

To date, over 300 million people worldwide have recovered from COVID, but concern remains that some organs, including the lungs, might suffer long-term impairment during post-COVID rehabilitation. A systematic review examining seven studies regarding effects of COVID on pulmonary function revealed that the most affected parameter during COVID rehabilitation was the diffusion capacity (DLCO), evident in approximately 40% of patients [34].

Regarding big numbers of affected people, potential drugs addressing long-lasting effects are of high unmet need. Our drug Treamid showed a promising potential for treatment of pulmonary-related pathologies of SARS-CoV-2 origin. However, as reported by different experts, current animal models all have limited translational value regarding pulmonary diseases. To run a first proof-of-concept study in humans in a newly emerging area, with a broad range of differentially affected tissues and outcomes, we first tested Treamid in a small cohort, investigating valuable endpoints and validating the translational potential of the effective dose from preclinical studies.

In this clinical trial the efficacy of Treamid treatment in patients recovered after COVID pneumonia was assessed based on frequency of clinically significant improvement of FVC and/or DLCO, defined as relative increase in FVC of ≥ 10% or a relative increase in FVC in the range of ≥ 5 to < 10% and a concomitant relative increase in DLCO of ≥ 15% compared to baseline [24, 25]. After a 4 week treatment period patients receiving Treamid had a significant higher clinical response compared to placebo controls. Besides the significant improvement in the primary endpoint, Treamid administration led to a decrease in dyspnea assessed by modified Borg scale. In contrast, there was no difference between Treamid and placebo in the mMRC scoring. The modified Borg scale is assessing dyspnea after a certain work load (here, 6MWT) whereas the mMRC scale is used to assess dyspnea in a daily-life setting [35]. A main problem using the mMRC, is the limited number of levels and thereby the potential of missing small and moderate changes. This might explain the discrepancy between the significant improvement of dyspnea in modified Borg scale without changes in mMRC [35]. The significant improvement of dyspnea assessed by modified Borg scale can be considered as a minimally clinical important difference [36], which is given as a change of 1 unit. However, due to the missing linearity of the Borg scale, changes are more pronounced at the higher end of the scale, reflecting patients with more severe symptoms [37].

Taken together the improvement of FVC and DLCO as well as the decrease in dyspnea, suggests that Treamid is an effective treatment in patients with long-term impairment of lung function after COVID pneumonia. As the majority of patients included this trial never smoked (81%) and did not have pathologies of the cardiovascular system (86%), it is likely that smoking status and the state of the cardiovascular system did not affect the results (for a detailed analysis of the effects of smoking and hypertension, see Additional file 2: Appendixes S13 and S14).

Although male sex is recognized as a predictor for increased COVID disease severity and mortality, female sex has been associated with a greater risk for persistent diffusion impairments months into recovery and predicts impaired DLCO 12 months after discharge [13, 38]. To test the effectivity of Treamid in potentially more severe patients, we conducted an exploratory analysis on the female population. The results indicate that Treamid was more effective regarding improvement of dyspnea measured by modified Borg scale as well as improved lung function and decreased lung damage in the female population. As previous findings indicated significantly lower TLC in subjects after mild to moderate COVID, the potential of Treamid to improve TLC in women may be advantageous [39, 40]. In line with previous reported higher post-COVID rates in women, Treamid might over a specific treatment option in this population and opens the potential for further clinical trials using Treamid for the rehabilitation of patients after COVID pneumonia.

An exploratory analysis of the effect of the age, time of hospitalization, and time since the onset of the first symptoms revealed no statistically significant differences between subpopulations (Additional file 2: Appendix S16, Additional file 1: Table S16.1)). However, in elder individuals and patients with a longer hospitalization period Treamid showed about 10% higher clinical efficacy compared to placebo. The time after onset of symptoms seemed not affect treatment efficacy.

The safety analysis did not reveal any clinically and statistically significant differences between the Treamid and placebo groups and additionally supports further clinical trials using Treamid in post-COVID rehabilitation.

Potential limitation of this exploratory trial are the small group sizes together with a high proportion of patients with normal or minor pulmonary abnormalities presenting a higher probability of spontaneous resolution. To validate the results of the exploratory study, a new Multicenter, Randomized, Double-blind, Placebo-controlled Study (NCT05516550) to Evaluate the Efficacy and Safety of Treamid in the Treatment of 412 Patients With Persistent Lung Damage and Reduced Exercise Tolerance Following Acute Coronavirus Infection was initiated in August 2022. To evaluate the long-lasting effects of the Treamid, a 4-week follow-up period is planned in this study.

The exploratory analysis in women was performed post-hoc and sample size was calculated based on the primary clinical endpoint. Therefore, the here present findings regarding Treamids’ efficacy in women are considered preliminary and need further support from larger populations.

The here described data clearly showed the beneficial effects of Treamid in the treatment of long-COVID patients by improving lung function as well as the clinical outcome of dyspnea. Based on this study, we were able to design a second phase II study, enrolling more patients with a defined primary endpoint and focusing on lung function and dyspnea. We therefore consider the presented data valuable for clinical development of a new potential drug for pulmonary-associated long-COVID symptoms.

Taken together, further large-scale multicenter randomized trials with more balanced populations are needed to thoroughly evaluate the effects of Treamid on the treatment of patients with post-COVID consequences.

## Conclusions

In summary, we have shown that peroral administration of 50 mg Treamid for 4 weeks was associated with clinically significant increase in FVC and/or DLCO, as well as decreasing dyspnea measured by modified Borg scale. Treamid was well tolerated and can be safely administered to patients discharged after COVID. An exploratory analysis showed that Treamid was more effective in women, decreasing dyspnea measured by Borg scale, lung damage and increasing TLC. Considering that according to the latest data female sex is a predictor of more severe functional impairment, the data presented here gives a potential great opportunity to treat this population, however more data is needed to validate these first findings.

## Supplementary Information

**Additional file 1. **The trial protocol, protocol amendments and statistical analysis plan.

**Additional file 2. **Supplementary Tables and Figures.

## Footnotes

1 For the CT, mMRC and modified Borg scale scoring see Additional file 2.

2 *One patient in the placebo group did not receive the drug and was therefore not included in the efficacy analysis.*
