# Supplementary material for: Efficacy and safety of Treamid in the rehabilitation of patients after COVID-19 pneumonia: a phase 2, randomized, double-blind, placebo-controlled trial
Source: J Transl Med. 2022 Nov 3;20:506. doi: 10.1186/s12967-022-03660-9 (PMC9632561; doi:10.1186/s12967-022-03660-9)
Supplement: Supplementary file 1 — Additional file 1. The trial protocol, protocol amendments and statistical analysis plan. [file 12967_2022_3660_MOESM1_ESM.docx]

**Supplement 1 for**

**Efficacy and Safety of Treamid in the Rehabilitation of Patients After COVID-19 Pneumonia: a phase 2, randomized, double-blind, placebo-controlled trial**

*Evgeny Bazdyrev^1^, Maria Panova^2^, Maria Brachs^3^, Elena Smolyarchuk^4^, Daria Tsygankova^1^, Liudmila Gofman^5^, Yana Abdyusheva^2^, Fedor Novikov^2^*

1. Research Institute for Complex Issues of Cardiovascular Diseases, 6, Sosnoviy Blvd., 650002 Kemerovo, Russia
2. PHARMENTERPRISES LLC, Skolkovo Innovation Center, Bolshoi Blvd., 42 (1), 143026 Moscow, Russia; m.panova@pharmenterprises.ru (M.P.); fnovikov@pharmenterprises.ru (F.N.); ya.abdyusheva@pharmenterprises.ru (Y.A.);
3. Treamid Therapeutics GmbH, c/o CoLaborator (Bayer), Building S141, Muellerstr. 178, 13353 Berlin, Germany
4. I.M. Sechenov First Moscow State Medical University (Sechenov University). Trubetskaya ul. 8, Moscow, 119991 Russia
5. Kemerovo Regional Clinical Hospital named after S.V. Belyaev, 22, Oktyabskiy pr., 650066, Kemerovo, Russia

Table of contents

[**Appendix 1** **Protocol of the clinical study** **COVID-TRE-03** 5](#_Toc102834680)

[**Appendix 2 Amendments to the Protocol** 75](#_Toc102834701)

[**Appendix 3 Statistical Methods Documentation** 151](#_Toc102834729)

# Appendix 1 Protocol of the clinical study COVID-TRE-03

**PROTOCOL OF THE CLINICAL STUDY**

**COVID-TRE-03**

| **Study Title:** | A multicenter, randomized, double-blind, placebo-controlled pilot study to evaluate the efficacy and safety of Treamide in the rehabilitation of patients after COVID-19 pneumonia |
| --- | --- |
| **Study Number:** | COVID-TRE-03 |
| **Study phase:** | II |
| **Name of the drug:** | Treamide |
| **Planned indication:** | Pulmonary fibrosis after COVID-19 pneumonia |
| **Sponsor:** | LLC "PHARMENTERPRISES" |
| **The contact person:** | Nebolsin Vladimir Evgenievich  Russia, 121205, Moscow,  The territory of the innovation center Skolkovo  Bolshoi boulevard, 42, building 1, office. 771, 772  Tel.: +7 (985) 728-75-72 |
| **Protocol date:** | July 20, 2020 |

**Confidentiality Statement**

By receiving this document, you agree that it is the Confidential Information of PHARMENTERPRICES LLC. You must not directly or indirectly publish, distribute or otherwise disclose its contents or make available to third parties any part of this document. You must also use this document only for the purpose for which it was provided to you. You may disclose information only after the written permission of PHARMENTERPRICES LLC, or representatives of authorized government bodies, or by decision of the judicial authorities. The fact of any disclosure of the information contained in this document, authorized or not, must be immediately brought to the attention of PHARMENTERPRICES LLC.

PROTOCOL APPROVAL PAGE

| Signature | (signed) |  | Date | *23.07. 2020* |
| --- | --- | --- | --- | --- |
| Nebolsin V.E.  General Manager  LLC "PHARMENTERPRISES" | |  |  |  |
|  |  |  |  |  |
| Signature | (signed) |  | Date | *23.07. 2020* |
| Trakhtenberg Yu.A.  Medical director  IPHARMA LLC | |  |  |  |
|  |  |  |  |  |
| Signature | (signed) |  | Date | *23.07. 2020* |
| Kasyanova O.V.  Medical Advisor  IPHARMA LLC | |  |  |  |
|  |  |  |  |  |
| Signature | (signed) |  | Date | July 23, 2020 |
| Ezhova E.V.  Project Manager  IPHARMA LLC | |  |  |  |

STATEMENT FROM PRINCIPAL INVESTIGATOR

| **FULL NAME.** : | |
| --- | --- |
| *I,* the undersigned, certify that I have read and understood this protocol. I agree to follow the COVID-TRE-03 protocol: "A multicenter, randomized, double-blind, placebo-controlled pilot study to evaluate the efficacy and safety of Treamide in the rehabilitation of patients after COVID-19 pneumonia."  I undertake to conduct this study in accordance with the requirements of the Good Clinical Practice Guidelines of the International Conference on Harmonization (ICH GCP) and the Eurasian Economic Union (EAEU), the principles set forth in the Declaration of Helsinki, as well as in accordance with the current legislation and applicable regulatory requirements of the Russian Federation. | |
| **Date:** | **Signature:** |

CLINICAL STUDY CONTACT INFORMATION

| Sponsor | LLC "PHARMENTERPRISES"  Russia, 121205, Moscow,  The territory of the innovation center Skolkovo  Bolshoi boulevard, 42, building 1, office. 771, 772 |
| --- | --- |
| Nebolsin V.E. | General Manager  Tel.: +7 (985) 728-75-72  Email address: nve1970@mail.ru |
| **CRO, MEDICAL EXAMINATION, PHARMACOVIGILANCE DATA PROCESSING AND BIOSTATISTICS** | IPHARMA LLC  Russia, 143026, Moscow,  The territory of the innovation center Skolkovo  st. Nobel, d.7  Tel.: +7 (495) 276-11-43  *Fax +7(495) 276-11-47*  Address for sending a safety report:  SAE@ipharma.ru |
| Vostokova N.V. | Chief Operations Officer  Tel.: +7 (926) 098-36-33  Email address: nv@ipharma.ru |
| Trakhtenberg Yu.A. | Medical director  Tel.: +7 (926) 098-21-07  Email address: jat@ipharma.ru |
| Ezhova E.V. | Project Manager  Tel.: +7 (926) 098-06-09  e-mail: eev@ipharma.ru |
| Kasyanova O.V. | Medical Advisor  Tel.: +7 (925) 421-52-23  Email address: okas@ipharma.ru |
| Kholkin P.V. | Director of Data Management  Tel.: +7 (926) 208-85-82  e-mail: khopv@ipharma.ru |
| Egorova A.N. | biostatistician  Tel.: +7 (926) 010-42-11  E-mail address: ean@ipharma.ru |
| **BIOANALYTICAL LABORATORY** | LLC NII KhimRar  Russia, 141401, MO, city of Khimki, Khimki, st. Rabochaya, d. 2A, building 1  Tel.: +7 (495) 925-3074  *Fax +7(495) 626-9780* |
| Koryakova A.G. | Head of Bioanalytics Laboratory  Tel.: +7 (495) 925-3074  Email address: agk@iihr.ru |
| **{U>WAREHOUSE<U}** | Koreks LLC  Russia, 142110, Moscow region, Podolsk, st. Stantsionnaya, 22, building 10  Tel.: +7 (499) 647-59-41  E-mail: info@corex-depot.com |
| Clinical sites | According to the approved list of centers |

**SYNOPSIS**

**Sponsor:** PHARMENTERPRICES LLC

**Investigational product name:** Treamide, film-coated tablets

**Active ingredient:** XC268BG

**Study Title:**

A multicenter, randomized, double-blind, placebo-controlled pilot study to evaluate the efficacy and safety of Treamide in the rehabilitation of patients after COVID-19 pneumonia

**Study Number:** COVID-TRE-03

**Research phase:** II

**Main goal**:

To evaluate the efficacy of Treamide versus placebo in patients with COVID-19 pneumonia based on the frequency of achieving a clinically significant change in forced vital capacity (FVC) and/or diffusive capacity of the lungs (DLCO) at Week 4 from baseline.

Clinically significant changes are considered to be a relative increase in FVC by ≥ 10% ***or*** a relative increase in FVC in the range from ≥ 5% to < 10% and a relative increase in DLCO by ≥ 15% [13, 14].

**Additional goals:**

To evaluate the efficacy and safety of Treamide versus placebo in patients with COVID-19 pneumonia based on the following parameters:

- Change in distance traveled in 6 minutes (6MWD) in Week 2 and Week 4 from baseline (based on the 6 Minute Walk Test);
- Change in Borg score at Week 2 and Week 4 from baseline (based on the 6 Minute Walk Test);
- Relative change in spirometry lung function parameters (forced expiratory volume in one second (FEV1), FVC, FEV1/FVC) at Week 1, Week 2, Week 3, and Week 4 from baseline;
- Relative change in body plethysmography lung function parameters at Week 2 and Week 4 from baseline (DLCO, total lung capacity (TLC), expiratory reserve volume (FRC));
- Change in the degree of lung damage according to computed tomography (CT) at Week 4 from baseline;
- Change in severity on the mMRC Breathlessness Scale at Week 1, Week 2, Week 3, and Week 4 from baseline;
- Change in total score on the King's Interstitial Lung Disease Questionnaire (KBILD) at Week 2 and Week 4 from baseline;
- Residual concentration Ctrough of the active substance of the drug Treamide (XC268BG);
- The frequency of adverse events (AE) and serious adverse events (SAE) of varying severity according to subjective complaints, physical examination, vital signs, laboratory tests and ECG.

**Rationale:**

Starting in December 2019, the novel coronavirus SARS-CoV-2 has caused an outbreak of a respiratory disease called COVID-19 in almost every country in the world.

Clinical and epidemiological observations of patients with COVID-19 indicate that SARS-CoV-2 infection can range from mild signs of respiratory disease to severe progressive pneumonia, multiple organ failure, and death [1, 2, 3, 4, 5]. The presence of foci of consolidation and fibrosis in the lungs according to chest CT results in the need for long-term rehabilitation of patients who have had COVID-19 pneumonia.

According to modern concepts, pulmonary fibrosis is the process of formation of fibrous (scar) tissue in the lungs, which leads to impaired respiratory function. With fibrosis, the elasticity and extensibility of the lung tissue decreases, the passage of oxygen and carbon dioxide through the wall of the alveoli (lung vesicles, in which the inhaled air contacts the blood) is difficult.

Patients who have undergone COVID-19 are characterized by the development of pulmonary edema, accompanied by the presence of neutrophil-macrophage inflammation [6], as well as changes in respiratory function, in particular, a decrease in forced expiratory volume (FEV1) [7], a decrease in forced vital capacity (FVC) [7] and a maximum expiratory flow rate of 50% forced vital capacity (MEF50) [8].

Currently, any specific pharmacotherapy for patients at the stage of rehabilitation after suffering from COVID-19 pneumonia has not been described. Thus, the search for effective and safe drugs that could be effective in patients with fibrosis remains an urgent task [16].

Antifibrotic therapy, which has demonstrated activity in a model of pulmonary fibrosis induced by intratracheal administration of bleomycin, may be useful in the treatment of COVID-19 both in the acute phase of the disease and in the prevention and treatment of long-term complications [9, 10, 11]. Indirect confirmation of this fact is the conduct of clinical trials of drugs developed for the treatment of chronic fibrotic diseases, in particular, drugs Nintedanib [10] and Pirfenidone [11] for the treatment of pulmonary fibrosis caused by COVID-19 infection.

PHARMENTERPRICES LLC is developing a new drug Treamide for the correction of fibrotic changes in the lungs at the stage of rehabilitation of patients after COVID-19 pneumonia.

The innovative drug Treamide is a bis-amide derivative of dicarboxylic acid. It was developed for use in patients with metabolic syndrome and age-related androgen deficiency. The mechanism of action of the drug formed the basis of its preclinical evaluation with the aim of repositioning for use in patients with COVID-19 pneumonia.

According to preclinical data, Treamide has anti-inflammatory and antifibrotic effects in models of lung injury, prevents tissue destruction, and improves respiratory function. Under conditions of pneumofibrosis, Treamide significantly (p<0.05) reduces the levels of total collagen, type I collagen, hydroxyproline, and the amount of deposited connective tissue in the lungs in mice. In a model of pulmonary fibrosis induced by intratracheal administration of bleomycin in mice, Treamide showed pronounced antifibrotic activity at a dose of 10 mg/kg per day. The model of fibrosis induced by intratracheal administration of bleomycin reproduces well the fibrotic response after acute lung injury (characteristic, in particular, of COVID-19 infection), rather than *de novo* progressive fibrosis [12]. It is important to note that the introduction of Treamide at a lower dose of 1 mg/kg did not lead to significant changes in the lung tissue in mice on the 21st day of the experiment. A potentially effective dose of 10 mg/kg per day in mice is approximately equivalent to 50 mg per day in humans.

Possible mechanisms of action of Treamide are an inhibitory effect on the migration of inflammatory cells to the lungs, as well as a decrease in the activity of synthesis and deposition of connective tissue.

The basis of the regenerative effects of Treamide is an inhibitory effect on the Notch-mediated mechanism for the development of pulmonary fibrosis and stimulation of the differentiation of lung stem cells (CD45-CD117+) and VEGF2+ endothelial cells.

In a phase I clinical study in healthy volunteers, Treamide showed good tolerability and a favorable safety profile at doses of 5 mg to 50 mg per day in single and multiple doses for 14 days. 4 AEs (asymptomatic Grade 3 and 4 laboratory changes) were reported only in the low-dose 5 mg cohort; No AEs associated with Treamide have been reported.

Based on data on the efficacy of Treamide in the most relevant disease model (a model of pulmonary fibrosis induced by intratracheal administration of bleomycin), as well as clinical data on the safety of Treamide at a dose of 50 mg once a day for 14 days in healthy volunteers, a pilot study is planned. phase II clinical trial to evaluate the safety and efficacy of Treamide at a dose of 50 mg once a day for 4 weeks in patients with fibrotic lung damage caused by the development of COVID-19 infection.

After the completion of this study, with the confirmation of the pilot hypothesis (proof-of-concept), it is planned to continue evaluating the efficacy and safety of Treamide in a multicenter, placebo-controlled, randomized phase III clinical trial of the efficacy and safety of Treamide in patients with fibrotic changes in the lungs after suffering COVID-19 pneumonia. .

**Study Design:**

This study is a multicenter, randomized, double-blind, placebo-controlled phase II pilot study to evaluate the efficacy and safety of Treamide in the rehabilitation of patients after COVID-19 pneumonia [15].

The study will be conducted in approximately 6-10 Russian clinical centers.

A total of 60 patients are planned to be included in the study. Considering a possible 10% dropout at the study selection stage, approximately 67 patients with pulmonary fibrotic changes after COVID-19 pneumonia will be screened.

***Screening***

At the Week -2 visit, following the signing of the Patient Information Sheet and the Informed Consent Form, screening procedures will be performed to assess inclusion/non-inclusion criteria. Screening procedures include collection of demographic data, medical history and concomitant therapy, physical examination, measurement of height, body weight and determination of BMI, assessment of vital signs and SpO2, ECG, mMRC dyspnea score, clinical and biochemical blood tests, urinalysis. Women of childbearing potential will be given a pregnancy test.

All patients will undergo a qualitative determination of SARS-CoV-2 RNA by PCR. The material for laboratory testing will be a swab from the nasopharynx and / or oropharynx. Confirmation of two consecutive negative PCR results at least 24 hours apart is required for inclusion in the study. In the case of previously confirmed elimination of the SARS-CoV-2 virus according to medical records, one determination of SARS-CoV-2 RNA by PCR with a negative result will be sufficient.

Patients will be assessed using the mMRC Breathlessness Scale, chest CT, spirometry, and body plethysmography with DLCO. These examinations should be conducted as close as possible to the planned date of randomization (not earlier than 5 days before it).

**In the event that screening procedures are initiated as part of the patient's discharge from the hospital after hospitalization of a patient diagnosed with COVID-19 pneumonia, sufficient time should be allowed for the completion of quarantine measures (about 2 weeks) before assessing the extent of damage and lung function.**

**The screening may use the results of examinations carried out as part of routine medical practice before obtaining informed consent (taking into account the window of the screening visit), except for the assessment on the mMRC Breathlessness Scale, CT of the chest, spirometry and body plethysmography with the definition of DLCO - these examinations should be carried out as much as possible close to the planned date of randomization (no earlier than 5 days before it).**

Patients who meet all eligibility criteria will be included in the study.

***Study therapy period***

At visit Week 0 prior to study therapy, registration of AEs and concomitant therapy, physical examination, body weight measurement, vital signs and SpO2 assessment, mMRC dyspnoea score, ECG, clinical and biochemical blood tests, and urinalysis will be performed. Women of childbearing potential will be given a pregnancy test. Patients will complete a KBILD questionnaire, followed by a 6 Minute Walk Test, which measures the distance the patient walks in 6 minutes and is assessed using the Borg Scale.

After completing all the necessary visit procedures, patients will be randomized into two groups in a 1:1 ratio.

Group 1 Treamide 50 mg - 30 patients

Group 2 Placebo - 30 patients

Patients will be given the required amount of study drug until the next visit, according to the therapy group.

The duration of study therapy will be 4 weeks. Patients will be recommended to continue the standard program of medical rehabilitation in a day hospital or outpatient setting (Stage 3 according to the Interim Guidelines "Medical Rehabilitation for Novel Coronavirus Infection (COVID 19)", Version 1 dated 05/21/2020 or current at the time of the study) [ sixteen].

Patients will visit the study site once a week during the study therapy phase. At Week 1, Week 2, and Week 3 visits, registration of AEs and concomitant therapy, study medication, body weight, vital signs and SpO2, mMRC dyspnea score, and spirometry will be performed.

At visit Week 2, a physical examination, ECG, clinical and biochemical blood tests, PK study, urinalysis, body plethysmography with DLCO will also be performed. Patients will complete the KBILD questionnaire. There will also be a 6 Minute Walk Test, which will measure the distance the patient walks in 6 minutes and evaluate with the Borg Scale. Women of childbearing potential will be given a pregnancy test.

At visit Week 4 (end of therapy), registration of AEs and concomitant therapy, registration of the study drug, physical examination, measurement of body weight, assessment of vital signs and SpO2, ECG, clinical and biochemical blood tests, PK study, urinalysis will be carried out. Patients will be assessed using the mMRC Breathlessness Scale, chest CT, spirometry, and body plethysmography with DLCO. Patients will complete a KBILD questionnaire, followed by a 6 Minute Walk Test, which measures the distance the patient walks in 6 minutes and is assessed using the Borg Scale. After completion of study therapy, patients will continue treatment in accordance with general recommendations. Women of childbearing potential will be given a pregnancy test.

***PK study***

A blood draw for the Ctrough PK study will be performed on all patients prior to PI administration at Week 0, Week 2, and Week 4.

***Follow-up period.***

Patient follow-up will continue for another two weeks. At Week 6, registration of AEs and concomitant therapy, physical examination, measurement of body weight, assessment of vital signs and SpO2, mMRC dyspnea score, ECG, clinical and biochemical blood tests, urinalysis will be carried out. Women of childbearing potential will be given a pregnancy test.

**Study population:**

The study is planned to include 60 patients with fibrotic changes in the lungs after suffering from COVID-19 pneumonia.

To be included in this clinical trial, patients must meet the following inclusion/exclusion criteria.

**Inclusion criteria:**

1. Signed Patient Information Sheet and informed consent form for participation in the study.
2. Men and women aged 18 to 75 inclusive.
3. Fibrotic changes in the lungs after suffering COVID-19 pneumonia:
4. The diagnosis of COVID-19 in history was confirmed by a positive qualitative analysis of SARS-CoV-2 RNA by PCR;
5. The appearance of the first symptoms of COVID-19 no more than 2 months before screening;
6. Fibrotic changes in the lungs, characteristic of COVID-19, were confirmed by chest CT at screening.
7. Negative test for COVID-19 at screening (confirmed).
8. Severity 2 (moderate) or 3 (severe) on the mMRC Breathlessness Scale at screening and randomization.
9. Decreased lung function FVC and/or DLCO < 80% predicted at screening.
10. Consent of patients to use adequate methods of contraception during the entire study and within 3 months after its completion. Adequate methods of contraception include the use of:

- oral or transdermal contraceptives;
- condom or diaphragm (barrier method) with sospermicide;
- intrauterine device.

**Non-inclusion сriteria:**

1. Women who are pregnant or breastfeeding or planning to become pregnant during a clinical study; women of childbearing potential (including those who are not surgically sterilized and who are less than 2 years postmenopausal) who do not use adequate contraceptive methods.
2. The use of invasive mechanical ventilation (iALV), plasma transfusion (including convalescent plasma) and other blood components during COVID-19 therapy.
3. History of chronic respiratory disease, including idiopathic pulmonary fibrosis (IPF), asthma, chronic obstructive pulmonary disease (COPD), or pulmonary hypertension, diagnosed prior to COVID-19.
4. Severe cardiovascular disease at present or within 6 months prior to screening, including: NYHA class III or IV chronic heart failure, clinically significant ventricular arrhythmias (ventricular tachycardia, ventricular fibrillation), unstable angina pectoris, myocardial infarction, heart and coronary surgery, significant valvular heart disease, uncontrolled arterial hypertension with systolic blood pressure > 180 mm Hg. and diastolic blood pressure > 110 mmHg, pulmonary embolism (PE) or deep vein thrombosis.
5. Nephrotic syndrome, moderate to severe chronic renal failure, or significant kidney disease with GFR < 60 ml/min at screening.
6. Cirrhosis of the liver in history; an increase in alanine aminotransferase (ALT) and / or aspartate aminotransferase (Act) by 3 or more times from the upper limit of normal (ULN) at screening; an increase in the level of total bilirubin by 2 or more times from ULN at screening.
7. Hemoglobin level < 90 g/l at screening.
8. Severe diseases of the central nervous system, including a history of seizures or conditions that can lead to their development; stroke or transient ischemic attack within 6 months prior to screening; traumatic brain injury or loss of consciousness within 6 months prior to screening; a brain tumor.
9. Signs of significant uncontrolled comorbidity, such as disorders of the nervous system, kidneys, liver, endocrine system and gastrointestinal tract, which, in the opinion of the Investigator, could prevent the patient from participating in the study.
10. Malignant neoplasms requiring chemotherapy within 6 months prior to screening.
11. History of HIV infection.
12. Prostate cancer or benign prostatic hyperplasia (BPH) with a residual urine volume of more than 100 ml in history in men.
13. Hypersensitivity or intolerance to any of the components of the study drug.
14. Participation in other clinical trials within 2 months prior to screening.
15. Taking the following drugs: bronchodilators, anticholinergics, corticosteroids, cytostatics, colchicine, cyclosporine A, interferon-γ-1b, bosentan, macitentan, etanercept, sildenafil, imatinib, n-acetylcysteine, warfarin, ambrisentan, nintedanib, pirfenidone 1 month before screening [17].
16. inability to read or write; unwillingness to understand and follow study protocol procedures; non-compliance with drug regimens or procedures that, in the opinion of the Investigator, may affect the results of the study or patient safety and prevent the patient from continuing to participate in the study; any other medical or serious psychiatric condition that makes the patient unsuitable for participation in the clinical study, limits the eligibility of obtaining informed consent, or may affect the patient's ability to participate in the study.

**Investigational drugs, doses and route of administration:**

**Investigational Drugs:** Treamide or Placebo

Active ingredient: XC268BG

Dosage form: film-coated tablets

Dosage: 50mg or 0mg

How to use: orally, once a day, in the morning, 30 minutes before breakfast

Storage conditions: In a place protected from light, at a temperature not exceeding 25°C.

Keep out of reach of children

Producer: JSC "IIHR" and CJSC "OHFK" Russia (by order of LLC "PHARMINTERPRICEZ", Russia)

**Duration of treatment:**

Each patient will participate in the study for approximately 8 weeks: screening 2 weeks, study therapy period 4 weeks, follow-up period 2 weeks. The start of enrollment in the study is scheduled for August 2020. It is planned that all patients will complete all study visits by November 2020.

**Primary Endpoint:**

Frequency of achieving a clinically significant change in FVC and/or DLCO at Week 4 from baseline. Clinically significant changes are considered to be a relative increase in FVC by ≥ 10% or a relative increase in FVC in the range from ≥ 5% to < 10% and a relative increase in DLCO by ≥ 15% [13, 14].

**Secondary Endpoints:**

- Mean change in distance walked in 6 minutes (6MWD) in Week 2 and Week 4 from baseline (based on the 6 Minute Walk Test) [19];
- Mean change in Borg score at Week 2 and Week 4 from baseline (based on the 6 Minute Walk Test);
- Mean relative change in spirometry lung function parameters (FEV1, FVC, FEV1/FVC) at Week 1, Week 2, Week 3, and Week 4 from baseline;
- Mean relative change in body plethysmography lung function parameters at Week 2 and Week 4 from baseline (DLCO, TLC, and FRC);
- Rate of reduction in lung injury (improvement) on CT scan at Week 4 from baseline;
- Mean change in severity on the mMRC Breathlessness Scale at Week 1, Week 2, Week 3, and Week 4 from baseline;
- Mean change in total score on the KBILD Questionnaire in Week 2 and Week 4 from baseline;
- Ctrough the active ingredient XC268BG.

**Safety ratings:**

Safety will be assessed based on the incidence and severity of AEs and SAEs based on subjective complaints, vital signs, SpO2, physical examination, laboratory findings, and ECG.

**Statistical methods:**

Analysis of the research results will be carried out in accordance with the approved Statistical Analysis Plan.

The safety population would be all patients who received at least one dose of study drug.

The Full Analysis Set (FAS) is defined as all randomized patients who received at least one dose of study drug and have at least one post-baseline efficacy score.

Per Protocol (PP) populations correspond to patients in the entire analysis population who will receive the study therapy in full and will not have significant deviations from the protocol.

All patients who received at least one dose of study drug and for whom sufficient PK samples were obtained to evaluate at least one PK parameter will be included in the pharmacokinetics population.

**Sample size**

The sample size was determined in accordance with the methodology of one-stage non-comparative design of phase II clinical trials (RP A’Hern, 2001).

At the moment, the process of recovery of lung function in patients who have undergone COVID-19 is not well understood [20]. In this study, 25% of patients in the Placebo group and 50% of patients in the Treamide group are expected to achieve a clinically significant change in FVC and/or DLCO at Week 4 from baseline. For α = 0.05 (one-sided), power 80%, p _0_ =25% and p _1_ =50%, 26 patients must be included in the analysis to test the hypothesis. 11 or more answers in 26 included patients will allow us to reject the null hypothesis (_W0_: p ≤ p_0_ ) in favor of the alternative one (_W1_: p ≥ p_1_) and thus make a positive decision regarding the possibility of further study of Treamide in phase III.

Taking into account the possible early withdrawal (impossibility of evaluating the primary endpoint) not exceeding 13%, 30 patients will be included in each group of study therapy. Thus, 60 patients will be randomized into the study.

**Efficacy analysis**

According to the RP algorithm A'Hern for single-stage phase II studies with unilateral α=0.05 and 80% power, 11 or more of 26 patients included in the analysis who achieved a clinically significant change in FVC and/or DLCO at Week 4 from baseline are sufficient to accept decisions on the advisability of further study of this dose in phase III (taking into account the evaluation of additional parameters of efficacy and safety). If the placebo arm also has a Week 4 response rate of 11 or more in the 26 included patients, then the primary endpoint assessment may be deferred to an earlier study visit.

If the number of responses to therapy in the Treamide group of 26 included patients is 10 or less, it will be concluded that there is no sufficient efficacy.

For secondary endpoints, between-group comparisons of frequencies and categorical data will be made using chi- ^square^ test or Fisher's exact test, continuous data using non-parametric Mann-Whitney test or Student's t-test. Descriptive statistics will be used to assess FC.

**Safety analysis**

Study drug dosing information, including daily doses, time of exposure, and total dose, will be presented descriptively for each cohort.

Safety will be assessed based on the incidence of AEs and SAEs based on subjective complaints, vital signs, SpO2, physical examination, laboratory findings, and ECG. The number and percentage of patients with AEs and SAEs will be tabulated by organ system class and preferred term (according to MedDRA), by study drug association, and severity. The results of laboratory studies will be summarized in tables of changes. Vital and laboratory parameters, SpO2 and ECG data will be presented using descriptive statistics.

**Number and date of the current version of the protocol:**

Protocol COVID-TRE-03 dated July 20, 2020

TABLE OF CONTENTS

[PROTOCOL APPROVAL PAGE 2](#_Toc102121286)

[STATEMENT FROM PRINCIPAL INVESTIGATOR 3](#_Toc102121287)

[CLINICAL STUDY CONTACT INFORMATION 4](#_Toc102121288)

[SYNOPSIS 5](#_Toc102121289)

[TABLE OF CONTENTS 14](#_Toc102121290)

[LIST OF ABBREVIATIONS AND DEFINITIONS OF TERMS 17](#_Toc102121291)

[1 INTRODUCTION 19](#_Toc102121292)

[2 OBJECTIVES OF THE CLINICAL STUDY 21](#_Toc102121293)

[2.1 primary goal 21](#_Toc102121294)

[2.2 Additional goals 21](#_Toc102121295)

[3 RESEARCH PLAN 22](#_Toc102121296)

[3.1 General design and study plan 22](#_Toc102121297)

[3.1.1 Screening 22](#_Toc102121298)

[3.1.2 Study therapy period 22](#_Toc102121299)

[3.1.3 Pharmacokinetic study 23](#_Toc102121300)

[3.1.4 Follow-up period. 23](#_Toc102121301)

[3.2 Study Design Rationale 26](#_Toc102121302)

[3.2.1 Rationale for placebo 26](#_Toc102121303)

[3.2.2 Randomization 26](#_Toc102121304)

[3.2.3 Investigational drug, doses, duration of administration 27](#_Toc102121305)

[3.2.4 Substantiation of the relevance of the preclinical model 27](#_Toc102121306)

[3.2.5 Endpoints 28](#_Toc102121307)

[3.2.6 Plan for further clinical development 29](#_Toc102121308)

[3.3 Study duration and dates 29](#_Toc102121309)

[3.4 Previous application experience 29](#_Toc102121310)

[3.4.1 Preclinical studies 29](#_Toc102121311)

[3.4.2 Clinical researches 32](#_Toc102121312)

[3.4.3 Risk/benefit ratio 33](#_Toc102121313)

[4 POPULATION SELECTION 35](#_Toc102121314)

[4.1 Study population 35](#_Toc102121315)

[4.2 Inclusion Criteria 35](#_Toc102121316)

[4.3 Non-inclusion Criteria 35](#_Toc102121317)

[4.4 Exclusion of patients from the study after randomization 36](#_Toc102121318)

[5 STUDY DRUG 38](#_Toc102121319)

[5.1 Description of study drug 38](#_Toc102121320)

[5.2 Study drug administration 38](#_Toc102121321)

[5.3 Method of allocation of patients to treatment groups 38](#_Toc102121322)

[5.4 Blinding of study drug 39](#_Toc102121323)

[5.5 dazzle 39](#_Toc102121324)

[5.6 Compliance with the therapy 39](#_Toc102121325)

[5.7 Concomitant therapy 39](#_Toc102121326)

[5.8 Prohibited Therapy 39](#_Toc102121327)

[5.9 Packaging and labeling of investigational product and concomitant therapy 39](#_Toc102121328)

[5.10 Storage and record keeping of the study drug 40](#_Toc102121329)

[6 DESCRIPTION OF PROCEDURES 41](#_Toc102121330)

[6.1 Informed consent 41](#_Toc102121331)

[6.2 Patient Registration 41](#_Toc102121332)

[6.3 Demographics and medical history 41](#_Toc102121333)

[6.4 Physical examination 41](#_Toc102121334)

[6.5 Vital signs and measurement of blood oxygen saturation (SpO2) 41](#_Toc102121335)

[6.6 Electrocardiography 41](#_Toc102121336)

[6.7 CT scan of the chest 42](#_Toc102121337)

[6.8 Analyzes performed in the laboratory 43](#_Toc102121338)

[6.8.1 Laboratory parameters 43](#_Toc102121339)

[6.8.2 PCR for SARS-CoV-2 RNA 43](#_Toc102121340)

[6.8.3 Pharmacokinetic study 44](#_Toc102121341)

[6.8.4 Receipt, preparation, storage and dispatch of biosamples 44](#_Toc102121342)

[6.9 mMRC (modified Medical Research Council) breathlessness scale 44](#_Toc102121343)

[6.10 6 Minute Walk Test (6MWD, Borg Scale) 45](#_Toc102121344)

[6.11 Questionnaire KBILD 45](#_Toc102121345)

[6.12 Spirometry (FEV1, FVC, FEV1/FVC) 45](#_Toc102121346)

[6.13 Body plethysmography (DLCO, TLC, FRC) 46](#_Toc102121347)

[6.14 Assessment of adverse events (AEs) 46](#_Toc102121348)

[6.14.1 Definitions of AE 46](#_Toc102121349)

[6.14.2 Pregnancy 47](#_Toc102121350)

[6.14.3 AE reporting 47](#_Toc102121351)

[6.14.4 Severity assessment 48](#_Toc102121352)

[6.14.5 Association of AEs with study drug 48](#_Toc102121353)

[6.14.6 Reporting SAEs/Pregnancy 48](#_Toc102121354)

[6.14.7 Withdrawal of patients from the study 49](#_Toc102121355)

[6.14.8 Procedures for dealing with emergencies 49](#_Toc102121356)

[6.14.9 Registration deadlines for AEs 49](#_Toc102121357)

[6.14.10 Expectancy 49](#_Toc102121358)

[6.14.11 Clinically Significant Laboratory Abnormalities 49](#_Toc102121359)

[6.15 Early withdrawal of patients from the study or withdrawal of the study drug 49](#_Toc102121360)

[6.16 Validity of measurements 50](#_Toc102121361)

[7 RESEARCH PROCEDURES 51](#_Toc102121362)

[7.1 Screening 51](#_Toc102121363)

[7.2 Study therapy period 51](#_Toc102121364)

[7.2.1 Visit 2, Week 0 51](#_Toc102121365)

[7.2.2 Visit 3, Week 1, Visit window ± 1 day 52](#_Toc102121366)

[7.2.3 Visit 4, Week 2, Visit window ± 2 days 52](#_Toc102121367)

[7.2.4 Visit 5, Week 3, Visit window ± 1 day 53](#_Toc102121368)

[7.2.5 Visit 6, Week 4, Visit window ± 2 days (End of therapy) 53](#_Toc102121369)

[7.3 observation period. 54](#_Toc102121370)

[7.3.1 Visit 7, Week 6, Visit window ± 3 days 54](#_Toc102121371)

[7.4 Unscheduled examinations 54](#_Toc102121372)

[7.5 Early termination of the study 54](#_Toc102121373)

[8 QUALITY ASSURANCE 55](#_Toc102121374)

[9 PLANNED STATISTICAL METHODS 56](#_Toc102121375)

[9.1 General provisions 56](#_Toc102121376)

[9.2 Determination of the sample size 56](#_Toc102121377)

[9.3 Randomization 56](#_Toc102121378)

[9.4 Populations for analysis 56](#_Toc102121379)

[9.4.1 Full analysis set (FAS) 56](#_Toc102121380)

[9.4.2 Protocol Population (Per Protocol, PP) 56](#_Toc102121381)

[9.4.3 Safety Population 56](#_Toc102121382)

[9.4.4 Population pharmacokinetics 56](#_Toc102121383)

[9.5 Patient distribution, demographic characteristics and baseline analysis 56](#_Toc102121384)

[9.6 Procedures for accounting for missing, unanalysable and doubtful data 57](#_Toc102121385)

[9.7 Performance analysis 57](#_Toc102121386)

[9.7.1 Primary efficacy endpoint 57](#_Toc102121387)

[9.7.2 Secondary efficacy endpoints 57](#_Toc102121388)

[9.8 Safety analysis 57](#_Toc102121389)

[9.9 Pharmacokinetic analysis 58](#_Toc102121390)

[10 ADMINISTRATIVE PROCEDURES 59](#_Toc102121391)

[10.1 Legal aspects 59](#_Toc102121392)

[10.1.1 Responsibilities of the Explorer 59](#_Toc102121393)

[10.2 Monitoring procedures 59](#_Toc102121394)

[10.3 Data registration in eCRF 59](#_Toc102121395)

[10.4 Storage of documentation 59](#_Toc102121396)

[10.5 Ethical aspects 60](#_Toc102121397)

[10.5.1 Independent Ethics Committee 60](#_Toc102121398)

[10.5.2 Informed Consent 60](#_Toc102121399)

[10.6 Research funding 60](#_Toc102121400)

[11 PLAN OF PUBLICATIONS 62](#_Toc102121401)

[12 REFERENCES 63](#_Toc102121402)

**LIST OF TABLES**

[Table 1. Schedule of procedures according to the COVID-TRE-03 protocol 25](#_Toc102121403)

[Table 2. List of indicators of laboratory tests of blood and urine 43](#_Toc102121404)

[Table 3. AE severity assessment 48](#_Toc102121405)

[Table 4. Assessing causality with study drug use 48](#_Toc102121406)

**LIST OF FIGURES**

[Picture 1. COVID-TRE-03 Study Design 24](#_Toc102121407)

**LIST OF APPS**

[Appendix 1. mMRC (modified Medical Research Council) dyspnea score 66](#_Toc102121408)

[Annex 2. 6-minute walk test (6MWD, Borg scale) (sample) 67](#_Toc102121409)

[Annex 3. King Interstitial Lung Disease Scoring Questionnaire (KBILD) - Sample 69](#_Toc102121410)

LIST OF ABBREVIATIONS AND DEFINITIONS OF TERMS

| 6MWD | 6-min walk distance |
| --- | --- |
| CD117 | Protein Tyrosine Kinase Kit |
| CD45 | Tyrosine protein phosphatase |
| DLCO | Diffusing capacity of the lungs for carbon monoxide |
| EMEA | European Medicines Agency |
| ERV | Residual air in the lungs |
| FEV | Forced expiratory volume |
| FEV1 | Forced expiratory volume in one second of a forced expiratory maneuver |
| FRC | Functional residual lung capacity |
| IWRS | Interactive Web Response System |
| KBILD | King's brief interstitial lung disease scoring questionnaire |
| mMRC | Shortness of breath scale (modified medical research council) |
| NCI-CTCAE | Common Terminology Criteria for Adverse Events National Cancer Institute, USA |
| Notch | Human transmembrane receptor protein (notch homolog 1, translocation-associated (Drosophila)) |
| per os | oral intake |
| RV | Total expiratory reserve volume |
| TLC | Total lung capacity |
| VEGF | Vascular endothelial growth factor |
| BP | Blood pressure |
| BAD | Biologically active additives |
| HIV | Human immunodeficiency virus |
| VMR | Temporary guidelines. |
| WHO | World Health Organization |
| E/T | Early termination |
| EAEU | **EURASIAN ECONOMIC UNION** |
| IVL | Artificial lung ventilation |
| IPF | Idiopathic pulmonary fibrosis |
| KT | CT scan |
| MSME | International Health Regulations |
| NIVL | Non-invasive ventilation |
| AR | Adverse reaction |
| IEC | Independent Ethics Committee (local) |
| AE | Adverse event |
| ARDS | Acute Respiratory Distress Syndrome |
| ORIT | Department of intensive care and intensive care |
| OF | End of therapy |
| PCR | Polymerase chain reaction |
| RNA | Ribonucleic acid |
| CHNR | Serious unexpected adverse reaction |
| CHP | Serious adverse reaction |
| SAE | Serious adverse event |
| SOP | Standard Operating Procedures |
| TD | Therapeutic dose |
| RR | Respiration rate |
| HR | Heart rate |
| eCRF | Electronic Case Report Form |
| ECG | - electrocardiogram |

1. **INTRODUCTION**

Starting in December 2019, the novel coronavirus SARS-CoV-2 has caused an outbreak of a respiratory disease called COVID-19 in almost every country in the world.

Clinical and epidemiological observations of patients with COVID-19 indicate that SARS-CoV-2 infection can range from mild signs of respiratory disease to severe progressive pneumonia, multiple organ failure, and death [1, 2, 3, 4, 5]. The presence of foci of consolidation and fibrosis in the lungs according to chest CT results in the need for long-term rehabilitation of patients who have had COVID-19 pneumonia.

According to modern concepts, pulmonary fibrosis is the process of formation of fibrous (scar) tissue in the lungs, which leads to impaired respiratory function. With fibrosis, the elasticity and extensibility of the lung tissue decreases, the passage of oxygen and carbon dioxide through the wall of the alveoli (lung vesicles, in which the inhaled air contacts the blood) is difficult.

Patients who have undergone COVID-19 are characterized by the development of pulmonary edema, accompanied by the presence of neutrophil-macrophage inflammation [6], as well as changes in respiratory function, in particular, a decrease in forced expiratory volume (FEV1) [7], a decrease in forced vital capacity (FVC) [7] and maximum expiratory flow rate at the level of 50% forced vital capacity (MEF50) [8].

Currently, any specific pharmacotherapy for patients at the stage of rehabilitation after suffering from COVID-19 pneumonia has not been described. Thus, the search for effective and safe drugs that could be effective in patients with fibrosis remains an urgent task [16].

Antifibrotic therapy, which has demonstrated activity in a model of pulmonary fibrosis induced by intratracheal administration of bleomycin, may be useful in the treatment of COVID-19 both in the acute phase of the disease and in the prevention and treatment of long-term complications [9]. Indirect confirmation of this fact is the conduct of clinical trials of drugs developed for the treatment of chronic fibrotic diseases, in particular, drugs Nintedanib [10] and Pirfenidone [11] for the treatment of pulmonary fibrosis caused by COVID-19 infection.

PHARMENTERPRICES LLC is developing a new drug Treamide for the correction of fibrotic changes in the lungs at the stage of rehabilitation of patients after COVID-19 pneumonia.

The innovative drug Treamide is a bis-amide derivative of dicarboxylic acid. It was developed for use in patients with metabolic syndrome and age-related androgen deficiency. The mechanism of action of the drug formed the basis of its preclinical evaluation with the aim of repositioning for use in patients with COVID-19 pneumonia.

According to preclinical data, the drug Treami shows anti-inflammatory and antifibrotic effects in models of lung damage, prevents tissue destruction, and improves respiratory function. Under conditions of pneumofibrosis, Treamide significantly (p<0.05) reduces the levels of total collagen, type I collagen, hydroxyproline, and the amount of deposited connective tissue in the lungs in mice. In a model of pulmonary fibrosis induced by intratracheal administration of bleomycin in mice, Treamide showed pronounced antifibrotic activity at a dose of 10 mg/kg per day. The model of fibrosis induced by intratracheal administration of bleomycin reproduces well the fibrotic response after acute lung injury (characteristic, in particular, of COVID-19 infection), rather than *de novo* progressive fibrosis [12]. It is important to note that the introduction of Treamide at a lower dose of 1 mg/kg did not lead to significant changes in the lung tissue in mice on the 21st day of the experiment. A potentially effective dose of 10 mg/kg per day in mice is approximately equivalent to 50 mg per day in humans.

Possible mechanisms of action of Treamide are an inhibitory effect on the migration of inflammatory cells to the lungs, as well as a decrease in the activity of synthesis and deposition of connective tissue.

The basis of the regenerative effects of Treamide is an inhibitory effect on the Notch-mediated mechanism for the development of pulmonary fibrosis, and stimulation of the differentiation of lung stem cells (CD45-CD117+) and VEGF2+ endothelial cells.

In a phase I clinical study in healthy volunteers, Treamide showed good tolerability and a favorable safety profile at doses of 5 mg to 50 mg per day in single and multiple doses for 14 days. 4 AEs (asymptomatic grade 3 and 4 laboratory changes) were reported only in the low dose cohort; No AEs associated with Treamide have been reported.

Based on preclinical data on the efficacy of Treamide in the most relevant disease model (a model of pulmonary fibrosis induced by intratracheal administration of bleomycin), as well as clinical data on the safety of Treamide at a dose of 50 mg once daily in humans, a phase II pilot clinical study is planned. to assess the safety and efficacy of Treamide at a dose of 50 mg once a day for 4 weeks in patients with fibrotic lung damage caused by the development of COVID-19 infection.

After the completion of this study, with the confirmation of the pilot hypothesis (proof-of-concept), it is planned to continue evaluating the efficacy and safety of Treamide in a multicenter, placebo-controlled, randomized phase III clinical trial of the efficacy and safety of Treamide in patients with fibrotic changes in the lungs after suffering COVID-19 pneumonia. .

1. OBJECTIVES OF THE CLINICAL STUDY
   1. **primary goal**

The primary objective of the study is to evaluate the efficacy of Treamide versus placebo in patients with COVID-19 pneumonia based on the rate of achieving a clinically significant change in forced vital capacity (FVC) and/or diffusive capacity of the lungs (DLCO) at Week 4 relative to initial values.

Clinically significant changes are considered to be a relative increase in FVC by ≥ 10% ***or*** a relative increase in FVC in the range from ≥ 5% to < 10% and a relative increase in DLCO by ≥ 15% [13, 14].

- 1. **Additional goals**

Additional study objectives include evaluating the efficacy and safety of Treamide versus placebo in patients with COVID-19 pneumonia based on the following parameters:

- Change in distance traveled in 6 minutes (6MWD) in Week 2 and Week 4 from baseline (based on the 6 Minute Walk Test);
- Change in Borg score at Week 2 and Week 4 from baseline (based on the 6 Minute Walk Test);
- Relative change in spirometry lung function parameters (forced expiratory volume in one second (FEV1), FVC, FEV1/FVC) at Week 1, Week 2, Week 3, and Week 4 from baseline;
- Relative change in body plethysmography lung function parameters at Week 2 and Week 4 from baseline (DLCO, total lung capacity (TLC), expiratory reserve volume (FRC));
- Change in the degree of lung damage according to computed tomography (CT) at Week 4 from baseline;
- Change in severity on the mMRC Breathlessness Scale at Week 1, Week 2, Week 3, and Week 4 from baseline;
- Change in total score on the King's Interstitial Lung Disease Questionnaire (KBILD) at Week 2 and Week 4 from baseline;
- PK parameters of the active substance of the drug Treamide (XC268BG);
- The frequency of adverse events (AE) and serious adverse events (SAE) of varying severity according to subjective complaints, physical examination, vital signs, laboratory tests and ECG.

1. RESEARCH PLAN
   1. **General design and study plan**

This study is a multicenter, randomized, double-blind, placebo-controlled, phase II pilot study to evaluate the efficacy and safety of Treamide in the rehabilitation of patients after COVID-19 pneumonia [15].

The study will be conducted in approximately 6-10 Russian clinical centers.

A total of 60 patients are planned to be included in the study. Considering a possible 10% dropout at the study selection stage, approximately 67 patients with pulmonary fibrotic changes after COVID-19 pneumonia will be screened.

- - 1. **Screening**

At the Week -2 visit, following the signing of the Patient Information Sheet and the Informed Consent Form, screening procedures will be performed to assess inclusion/non-inclusion criteria. Screening procedures include the collection of demographic data, medical history and concomitant therapy, physical examination, measurement of height, body weight and BMI, assessment of vital signs and SpO2, ECG, mMRC dyspnea score, clinical and biochemical blood tests, urinalysis. Women of childbearing potential will be given a pregnancy test.

All patients will undergo a qualitative determination of SARS-CoV-2 RNA by PCR. The material for laboratory testing will be a swab from the nasopharynx and / or oropharynx. Confirmation of two consecutive negative PCR results at least 24 hours apart is required for inclusion in the study. In the case of previously confirmed elimination of the SARS-CoV-2 virus according to medical records, one determination of SARS-CoV-2 RNA by PCR with a negative result will be sufficient.

Patients will be assessed using the mMRC Breathlessness Scale, chest CT, spirometry, and body plethysmography with DLCO. These examinations should be conducted as close as possible to the planned date of randomization (not earlier than 5 days before it).

**In the event that screening procedures are initiated as part of a patient's discharge from the hospital after hospitalization for COVID-19 pneumonia, sufficient time should be allowed for the completion of quarantine measures (about 2 weeks) before assessing the extent of damage and lung function.**

**The screening may use the results of examinations carried out as part of routine medical practice before obtaining informed consent (taking into account the window of the screening visit), except for the assessment on the mMRC Breathlessness Scale, CT of the chest, spirometry and body plethysmography with the definition of DLCO - these examinations should be carried out as much as possible close to the planned date of randomization (no earlier than 5 days before it).**

Patients who meet all eligibility criteria will be included in the study.

- - 1. **Study therapy period**

At visit Week 0 prior to study therapy, registration of AEs and concomitant therapy, physical examination, body weight measurement, vital signs and SpO2 assessment, mMRC dyspnoea score, ECG, clinical and biochemical blood tests, and urinalysis will be performed. Women of childbearing potential will be given a pregnancy test. Patients will complete a KBILD questionnaire, followed by a 6 Minute Walk Test, which measures the distance the patient walks in 6 minutes and is assessed using the Borg Scale.

After completing all visit procedures, patients will be randomized into two groups in a 1:1 ratio.

Group 1 Treamide 50 mg - 30 patients

Group 2 Placebo - 30 patients

Patients will be given the required amount of study drug until the next visit, according to the therapy group.

The duration of study therapy will be 4 weeks. Patients will be recommended to continue the standard program of medical rehabilitation in a day hospital or outpatient setting (Stage 3 according to the Interim Guidelines "Medical Rehabilitation for Novel Coronavirus Infection (COVID 19)", Version 1 dated 05/21/2020 or current at the time of the study) [ sixteen].

Patients will visit the study site once a week during the study therapy phase. At Week 1, Week 2, and Week 3 visits, registration of AEs and concomitant medications, study medication, body weight, vital signs and SpO2, mMRC dyspnea score, and spirometry will be performed. Women of childbearing potential will be given a pregnancy test.

At the Week 2 visit, physical examination, ECG, CBC, CBC, PK blood sampling, urinalysis, body plethysmography with DLCO will also be performed. Patients will complete the KBILD questionnaire. There will also be a 6 Minute Walk Test, which will measure the distance the patient walks in 6 minutes and evaluate with the Borg Scale. Women of childbearing potential will be tested for pregnancy

At visit Week 4 (end of therapy), registration of AEs and concomitant therapy, accounting for study drug, physical examination, measurement of body weight, assessment of vital signs and SpO2, ECG, clinical and biochemical blood tests, blood sampling for PK, urinalysis . Patients will be assessed using the mMRC Breathlessness Scale, chest CT, spirometry, and body plethysmography with DLCO. Patients will complete a KBILD questionnaire, followed by a 6 Minute Walk Test, which measures the distance the patient walks in 6 minutes and is assessed using the Borg Scale. After completion of study therapy, patients will continue treatment in accordance with general recommendations. Women of childbearing potential will be tested for pregnancy

- - 1. **Pharmacokinetic study**

A blood draw for the Ctrough PK study will be performed on all patients prior to PI administration at Week 0, Week 2, and Week 4.

- - 1. **Follow-up period.**

Patient follow-up will continue for another two weeks. At Week 6, registration of AEs and concomitant therapy, physical examination, measurement of body weight, assessment of vital signs and SpO2, mMRC dyspnea score, ECG, clinical and biochemical blood tests, urinalysis will be carried out. Women of childbearing potential will be given a pregnancy test.

Figure 1 shows the Schematic of the Clinical Study.

The schedule of examinations and the time of their implementation are presented in Table 1.

Picture 1. COVID-TRE-03 Study Design

|  | Screening | Investigational Therapy | | | | Follow-up |  |
| --- | --- | --- | --- | --- | --- | --- | --- |
|  |  |  | | | |  |  |
|  | ~67 patients | Treamide 50 mg (n=30) | | | |  |  |
|  |  | Placebo (n=30) | | | |  |  |
|  |  |  | | | |  |  |
| Visit | V 1 | V 2 | V 3 | V 4 | V 5 | V 6 | V 7 |
| Week | W-2 | W0 | W1 | W2 | W3 | W4 | W6 |

Table 1. Schedule of procedures according to the COVID-TRE-03 protocol

| **Procedures** | **Screening[[1]](#footnote-1)** | **Investigational Therapy** | | | | | **Follow-up** | **E/T** |
| --- | --- | --- | --- | --- | --- | --- | --- | --- |
| Visit (V) | V 1 | V 2 | V 3 | V 4 | V 5 | V 6 | V 7 |  |
| Week (W) | W-2 | W0 | W1 | W2 | W3 | W4 (ET) | W6 |  |
| Visit/procedures window | Day -28...0[[2]](#footnote-2) |  | ± 1 day | ± 2 days | ± 1 day | ± 2 days | ± 3 days |  |
| Informed consent | X |  |  |  |  |  |  |  |
| Demographic data | X |  |  |  |  |  |  |  |
| Medical history (including history of COVID-19) | X |  |  |  |  |  |  |  |
| Physical examination | X | X |  | X |  | X | X | X |
| Body weight, height, BMI[[3]](#footnote-3) | X | X | X | X | X | X | X | X |
| Vital signs, SpO2 | X | X | X | X | X | X | X | X |
| SARS-CoV-2 RNA by PCR[[4]](#footnote-4) | X |  |  |  |  |  |  |  |
| Complete Blood Count[[5]](#footnote-5) | X | X |  | X |  | X | X | X |
| Blood chemistry; | X | X |  | X |  | X | X | X |
| PK study[[6]](#footnote-6) |  | Х |  | Х |  | Х |  |  |
| General urine analysis | X | X |  | X |  | X | X | X |
| Pregnancy test[[7]](#footnote-7) | X | X |  | X |  | X | X | X |
| 12-lead ECG | X | X |  | X |  | X | X | X |
| mMRC breathlessness scale | Х | X | X | X | X | X | X | X |
| 6 Minute Walk Test (6MWD, Borg Scale) |  | X |  | X |  | X |  | X |
| Questionnaire KBILD |  | X |  | X |  | X |  | X |
| chest CT | X |  |  |  |  | X |  | X |
| Spirometry (FEV1, FVC, FEV1/FVC) | X | X | X | X | X | X |  | X |
| Body plethysmography (DLCO, TLC, FRC) | X |  |  | X |  | X |  | X |
| Evaluation of inclusion / non-inclusion criteria | X | X |  |  |  |  |  |  |
| Randomization |  | X |  |  |  |  |  |  |
| Distribution of the study drug |  | X | X | X | X |  |  |  |
| Study drug return and compliance assessment |  |  | X | X | X | X |  | X |
| Concomitant therapy | X | X | X | X | X | X | X | X |
| Adverse Events | X | X | X | X | X | X | X | X |

Abbreviations: Η - week, D - day, ET - end of therapy, D/3 - early termination.

- 1. **Study Design Rationale**

This study is double-blind, randomized, placebo-controlled. The placebo control design was chosen to objectify the findings.

This study is a phase II pilot study in patients with pulmonary fibrosis after prior COVID-19 pneumonia. This study will provide initial data on the efficacy and safety of Treamide 50 mg once daily with a view to further evaluating efficacy and safety in phase III.

Patients with fibrotic changes in the lungs after suffering from COVID-19 pneumonia were selected as the study population. The diagnosis of COVID-19 in history should be confirmed by a positive qualitative analysis of SARS-CoV-2 RNA by PCR; the onset of the disease (appearance of the first symptoms) no more than 2 months before screening. Fibrotic changes in the lungs, characteristic of COVID-19, should be confirmed by chest CT at screening.

- - 1. **Rationale for placebo**

According to the WMA Declaration of Helsinki, the use of a placebo in a study is possible when, for compelling and scientifically sound methodological reasons, the use of a placebo is necessary to evaluate the efficacy or safety of the intervention under study. In this case, patients receiving placebo will not be exposed to the additional risk of causing serious or irreversible damage to health as a result of not receiving the best of the interventions already proven.

Subject to this requirement, only patients with confirmed negative SARS-CoV-2 RNA quantification by PCR at screening and with a decrease in FVC and/or DLCO lung function < 80% predicted will be included in the study.

According to the concept of the Belmont Report [21], in relation to this study, a placebo-controlled design is preferable because:

- Currently, any specific pharmacotherapy for patients at the stage of rehabilitation after suffering from COVID-19 pneumonia has not been described [16];
- the risks of taking a placebo are low (pulmonary rehabilitation for patients who have undergone new community-acquired pneumonia COVID-19 does not currently provide for specific pharmacotherapy) [16];
- The effectiveness of a drug with a similar mechanism of action (Nintedanib) has not yet been proven, the study of the efficacy and safety of Nintedanib in the treatment of pulmonary fibrosis in patients with moderate to severe COVID-19 will be completed no earlier than August 2020 [22].

Thus, the use of placebo in this study is ethical and evidence-based, which complies with the requirements of the WMA Declaration of Helsinki.

In addition, if the patient's condition deteriorates due to increased respiratory symptoms, decreased lung function, or episodes of acute respiratory deterioration, the patient will be withdrawn from the study and, if necessary, admitted to a hospital for medical care. Criteria for early withdrawal of patients from the study are presented in section 6.14.7.

- - 1. **Randomization**

Patients will be randomized into two groups in a 1:1 ratio using the IWRS system. Randomization will be stratified by

1. presence or absence of risk factors (age ≥ 60 years and/or presence of concomitant chronic diseases)
2. and the initial degree of lung damage according to CT data (CT 1, CT 2, CT 3 and CT 4)

The stratification method will be described in more detail in the randomization protocol.

The stratification will make the groups comparable in terms of these indicators and thus will allow to level the possible influence of these factors on the response rate in both groups of study therapy.

- - 1. **Investigational drug, doses, duration of administration**

The drug Treamide will be taken orally, once a day, in the morning, 30 minutes before breakfast. The duration of taking the drug Treamide will be 4 weeks (28 days). This time interval was chosen as necessary and sufficient to obtain primary data on the effectiveness of the drug Treamide in patients with fibrotic changes in the lungs after suffering COVID-19 pneumonia based on the data of preclinical and clinical studies, as well as on the basis of the duration of the course of treatment with registered drugs with a similar mechanism of action [ 17].

In a phase I study in healthy volunteers, the safety and tolerability of Treamide 50 mg administered once daily for 14 days of the study was shown. None of the AEs reported during the study met the criteria for SAEs and, in the opinion of the Investigators, were not related to study medication or placebo. According to a study of FC in healthy volunteers, with a single dose of Treamide at a dose of 50 mg, T1 / 2 was 11.6 hours, with multiple doses - 12.1 hours. The cumulation factor (Fc) was 1.17. With repeated administration of 50 mg 1 time per day, the total exposure of the drug Treamide in blood plasma increases by 20% compared with a single dose. Thus, it is assumed that taking once a day is reasonable [23].

According to preclinical data, Treamide has anti-inflammatory and antifibrotic effects in models of lung injury, prevents tissue destruction, and improves respiratory function. In a model of pulmonary fibrosis induced by intratracheal administration of bleomycin, Treamide at a dose of 10 mg/kg (corresponding to a dose of 50 mg per day in terms of a person) prevented the infiltration of the interstitium of the alveoli and alveolar ducts by inflammatory cells and the development of fibrosing alveolitis in the lung tissue of mice. In cytometric studies, a decrease in the number of alveolar and interstitial macrophages, dendritic cells and lymphocytes in the lungs of mice of the experimental group was found compared to the pathological control. Treamide at a dose of 10 mg/kg statistically significantly (p<0.05) inhibited the synthesis of total collagen, type I collagen, hydroxyproline and the deposition of fibrous masses in the interstitium of the lungs of animals with pulmonary fibrosis induced by intratracheal administration of bleomycin. It is important to note that the introduction of the drug at a lower dose of 1 mg/kg did not lead to significant changes in the lung tissue in mice on the 21st day of the experiment.

Thus, based on data on the efficacy of Treamide in the most relevant model of the disease (a model of pulmonary fibrosis induced by intratracheal administration of bleomycin), as well as clinical data on the safety of Treamide at a dose of 50 mg once a day in humans, it is planned to conduct pilot clinical study to evaluate the safety, tolerability and efficacy of Treamide at a dose of 50 mg once a day for 28 days in patients with fibrotic lung damage caused by the development of COVID-19 infection.

- - 1. **Substantiation of the relevance of the preclinical model**

Among the currently used models of experimentally induced pulmonary fibrosis, the bleomycin-induced fibrosis model is the most commonly used [26,27]. The pathogenesis of pulmonary fibrosis induced by intratracheal administration of bleomycin is based on the ability of the drug to cause direct cell damage due to the induction of DNA strand breaks, the formation of free radicals, and subsequent oxidative stress [28]. Overproduction of reactive oxygen species due to metal ion chelation leads to epithelial cell death (days 1-3), excessive infiltration of lung inflammatory cells (neutrophils - days 3-9, lymphocytes on day 6), increased expression of profibrotic cytokines (including IL -1b, IL-6, IL-13, TGF-β) and ultimately to fibroblast activation, extracellular matrix deposition and fibrosis development (days 10-21 with a peak around day 14), these processes are detailed in the molecular [ 27, 29, 30] and histological [31, 32] levels.

It is important to note that the model of fibrosis induced by intratracheal administration of bleomycin reproduces well the fibrotic response after acute lung injury (characteristic, in particular, for COVID-19 infection), rather than de novo progressive fibrosis [34]. During the development of COVID-19 infection, the initial rapid viral replication causes massive death of epithelial and endothelial cells, which leads to increased vascular permeability and induces aberrant production of pro-inflammatory cytokines and chemokines. Excessive production of chemokines (in particular IL-1b, IL-6, IL-13, TGF-β) leads to aberrant chemotaxis of monocytes, macrophages and neutrophils, which produce a large number of reactive oxygen species and cause further damage to surrounding tissues and increase the death of epithelial and endothelial cells [35].

Thus, bleomycin-induced development of oxidative stress associated with damage and death of epithelial and endothelial cells, increased vascular permeability and aberrant infiltration of inflammatory cells in lung tissue well reflects the pathogenetic features of the development of COVID-19 infection [34,33]. Antifibrosis Therapy Demonstrated Activity in Model

pulmonary fibrosis induced by intratracheal administration of bleomycin may be useful in the treatment of COVID-19 both in the acute phase of the disease and in the prevention and treatment of long-term complications [33]. Indirect confirmation of this fact is the conduct of clinical trials of drugs developed for the treatment of chronic fibrotic diseases, in particular, drugs Nintedanib [36] and Pirfenidone [37] for the treatment of pulmonary fibrosis caused by COVID-19 infection.

- - 1. **Endpoints**

There are currently no specific guidelines for selecting the primary endpoint in studies in patients with COVID-19-associated pulmonary fibrosis. The primary end point for preliminary efficacy in this study was the frequency of achieving a clinically significant change in FVC and/or DLCO at Week 4 from baseline. This endpoint is a surrogate for patient progress assessments used in phase II studies in patients with IPF. This parameter is pharmacodynamic and allows assessing the direct effect of the study therapy on the function and diffusion capacity of the lungs, which indirectly indicates a possible clinical improvement in the patient's condition. The use of a surrogate endpoint is acceptable in phase II studies for preliminary evaluation of a pilot hypothesis (proof-of-concept) and a decision on the possibility of further study of the drug in phase III studies. Clinically significant changes are considered to be a relative increase in FVC by ≥ 10% *or* a relative increase in FVC in the range from ≥ 5% to < 10% and a relative increase in DLCO by ≥ 15% [13, 14].

In addition, additional efficacy parameters will be assessed in the study based on the 6 Minute Walk Test, lung function and diffusivity assessment, CT scan of the lungs, as well as the mMRC Breathlessness Scale and the KBILD Questionnaire. Based on the preliminary efficacy data obtained in this study, the optimal duration of treatment will be selected for further study and the most relevant endpoint, which will be used as primary in the phase III clinical trial.

The incidence of AEs and SAEs will be assessed based on subjective complaints, physical examination, vital signs, laboratory tests, and ECG.

- - 1. **Plan for further clinical development**

In case of successful completion of this study, it is planned to conduct a multicenter, double-blind, placebo-controlled, randomized Phase III clinical trial to evaluate the efficacy and safety of Treamide in patients with pulmonary fibrosis after COVID-19 pneumonia. In parallel, documents will be submitted for obtaining a temporary registration certificate for the Treamide drug as part of the procedure for Decree of the Government of the Russian Federation of 03.04.2020 N 441.

- 1. **Study duration and dates**

Each patient will participate in the study for approximately 8 weeks: screening 2 weeks, study therapy period 4 weeks, follow-up period 2 weeks. The start of enrollment in the study is scheduled for August 2020. It is planned that all patients will complete all study visits by November 2020.

- 1. Previous application experience
     1. Preclinical studies
        1. Preclinical pharmacology

The mechanism of action of Treamide is associated with the inhibition of notcW1-mediated proliferation of various types of progenitor notcW1+ cells and the induction of their differentiation into specialized (notcW1-) cells. This mechanism has been shown in *in vitro experiments* and confirmed in *in vivo* disease models.

Inhibition of notcW1-mediated proliferation and a decrease in the total number of notcW1+ cells, which occurs under the action of Treamid, leads to suppression of notcW1+-mediated production of connective tissue growth factor [38, 39] at an early stage of the disease, as well as components of the extracellular matrix (fibronectin and type I collagen) at an early stage of the disease. later stages of pathology. In addition, the suppression of notcW1+ leads to a decrease in the activation and migration of macrophages into lung tissue. Since macrophages play a key role in the synthesis of the extracellular matrix (macrophage arginase is the most important enzyme in the synthesis of proline and collagen), as well as in the development of the inflammatory response and the production of interleukins, a decrease in the migration of macrophages into lung tissues leads to the suppression of fibrosis.

In addition, activation of differentiation, leading to an increase in the number of mature epithelial and endothelial cells in lung tissue, is the central mechanism for accelerating lung regeneration in pulmonary fibrosis, which was confirmed by an increase in the number of CD31+ mature endothelial cells in lung tissue.

In in vitro experiments, the direct effect of the drug Treamide on the precursors of endothelial and epithelial cells of the lungs of intact control and pathological control was evaluated. In the culture of epithelial and endothelial cells isolated from the lungs of mice of the experimental group on the 21st day of the experiment, Treamide did not affect CD45-Ter119-CD49f+ epithelial progenitors and CD45-CD31+CD34+ endothelial progenitor cells expressing notcW1. Meanwhile, in vitro, the direct action of the substance reduced the number of angiogenesis precursor cells (CD45-CD117+Flk1+) and lung stem cells (CD45-CD117+) expressing notcW1. Thus, the regenerative effects of Treamide may be based on an inhibitory effect on the notch-mediated mechanism of IPF development, and stimulation of the differentiation of lung stem cells (CD45-CD117+) and VEGF2+ endothelial cells.

The study of the antifibrotic properties of the drug Treamide on the model of bleomycin-induced pneumofibrosis was carried out on mice. The model of fibrosis induced by intratracheal administration of bleomycin reproduces well the fibrotic response after acute lung injury (characteristic in particular for COVID-19 infection) rather than *de novo* progressive fibrosis [12]. The introduction of the drug at a dose of 1 mg/kg did not lead to significant changes in the lung tissue in mice on the 21st day of the experiment. Treamide at a dose of 10 mg/kg statistically significantly (p<0.05) inhibited the synthesis of total collagen, collagen type I, hydroxyproline and the deposition of fibrous masses in the interstitium of the lungs, as well as tissue destruction in the lungs in animals with pulmonary fibrosis induced by intratracheal administration of bleomycin .

The anti-inflammatory properties of Treamide were studied in a mouse model of bleomycin-induced pneumofibrosis. The introduction of Treamide at a dose of 10 mg/kg in the inflammation phase contributed to a decrease in the level of IL-13 and connective tissue growth factor, as well as to the normalization of the Clara cell protein level in lung homogenates of mice with pneumofibrosis compared with pathological control.

Also, the anti-inflammatory properties of the drug Treamide were studied on a model of neutrophil-macrophage inflammation of the lung, accompanied by impaired respiratory function in guinea pigs. The course administration of Treamide significantly increased the mid-expiratory airflow rate (EF50) and peak expiratory flow (PEF) to the intact level. The results obtained allow us to conclude that the pharmaceutical substance of Treamide has a pronounced anti-inflammatory effect and its pharmacological activity in relation to the normalization of respiratory function in pathologies accompanied by a decrease in peak expiratory flow rate (PEF) and mid-expiratory airflow rate (EF50), which may indicate a therapeutic potential drug for COVID-19 induced pulmonary dysfunction.

- - - 1. **Preclinical pharmacokinetics**

The study of the metabolism and pharmacokinetics of the drug substance Treamide included the determination of drug stability in human liver microsomes, rat and human hepatocytes, artificial gastric juice, binding to rat and human plasma proteins, inhibition of human liver cytochromes, permeability and Pgp-substrate specificity in the intestinal cell model Caco-2 line, study of pharmacokinetic parameters, excretion and distribution after a single intragastric administration to rats and accumulation after repeated intragastric administration to rats. The listed preclinical studies of metabolism and pharmacokinetics have shown that the drug is practically not metabolized, has low bioavailability, but, nevertheless, is significantly distributed in such animal organs as the testes, prostate gland and bone marrow.

A comparative study of the pharmacokinetics of Treamide when administered orally to dogs in 15 mg of the pharmaceutical substance and the finished dosage form (3 tablets of 5 mg) did not show statistically significant differences in the parameters T _max_, C _max_, AUC _0-t_ and AUC _0-inf_, T _1 / 2_, MRT _0-inf_.

- - - 1. **Toxicology**

A study of the acute toxicity of the pharmaceutical substance of Treamide in mice showed that the degree of intoxication is dose-dependent. For male and female mice, LD50 when administered intragastrically is more than 5500 mg/kg; LD50 for male mice with intraperitoneal injection is 1074±167 mg/kg; for females - 1000±167 mg/kg. Thus, the active substance of Treamide is a moderately toxic compound and belongs to the III class of toxicity in accordance with GOST 12.1.007-76.

A study of the acute toxicity of the pharmaceutical substance of Treamide in rats showed the absence of toxic effects of the substance when administered intragastrically (LD50 more than 5500 mg/kg). The degree of intoxication with intraperitoneal administration is dose-dependent. LD50 for females is 1324±199 mg/kg, for male rats - 1173±169 mg/kg. Thus, in accordance with GOST 12.1.007-76, the active substance of Treamide is a low-toxic compound and belongs to the IV class of toxicity.

A study of the chronic toxicity of Treamide in rats (3 months) showed that with intragastric administration of therapeutic doses (TD) - 5 mg/kg, as well as 5TD (25 mg/kg) and 10TD (50 mg/kg) for 3 months, the drug Treamide does not have any toxic effect on the functional state of organs and body systems of experimental animals.

The study of chronic toxicity of GLF preparation Treamide (5 mg tablets and 50 mg tablets) with intragastric administration was carried out in male rabbits (1 month, 3 months) and female rabbits (3 months). The results showed that the drug Treamide in TD for 4 weeks does not have a toxic effect on the organs and systems of male rabbits, with the exception of a slight increase in the level of glucose and cholesterol in the blood, as well as an increase in the weight gain of rabbits in the process of animal growth; within 3 months does not have a pronounced toxic effect on the functional state of the organs and systems of the body of male rabbits and female rabbits.

A study of the chronic toxicity of GLF of the drug Treamide when administered orally in Beagle dogs for 6 months at doses of 1.6 mg/kg (TD) and 16 mg/kg (10TD) to males and 16 mg/kg (10TD) to females showed that Treamide in TD and 10TD had no toxic effects on the organs and systems of male and female dogs.

The study of the reproductive toxicity of the Treamide drug substance in rats included: the study of the effect on reproductive (generative) function, the study of embryo- and fetotoxic effects recorded in the antenatal period of development and the study of antenatal effects recorded in the postnatal period of development. The data obtained allow us to conclude that the substance of the preparation Treamide intragastrically in TD (4.3 mg/kg) and 10TD (43.0 mg/kg) does not have reproductive toxicity.

The study of the effect of Treamide intragastrically at 3 doses (0.5, 5 and 50 mg/kg) on the generative function of male rats included an assessment of the fertility index and the pregnancy index when mating male rats of experimental groups with intact females for 10 days. The results allowed us to conclude that the administration of Treamide at the stage of progenesis (the duration of the entire cycle of spermatogenesis) at doses of 0.5, 5, and 50 mg/kg does not have a toxic effect on the reproductive system of male rats.

The study of the allergenicity of the pharmaceutical substance Treamide in TD and 10TD intragastrically once a day for 14 days was performed on male and female guinea pigs, Balb/c mice, CBA/CaLac mice and outbred mice in the following temps: 1) reaction general anaphylaxis (anaphylactic shock) in guinea pigs; 2) active cutaneous anaphylaxis in mice; 3) indirect reaction of degranulation of mouse mast cells; 4) "delayed" type hypersensitivity reaction (DTH) in mice; 5) inflammatory response to concanavalin A; 6) conjunctival test on guinea pigs; 7) method of skin applications on guinea pigs. The drug Treamide in the studied dosages and under the selected experimental conditions did not have allergenic properties.

The mutagenic activity of the pharmaceutical substance of Treamide was studied in the *Salmonella* /microsome test (Ames test) and in the cytogenetic test for accounting for chromosomal aberrations in mouse bone marrow cells. The results of the study of the drug Treamide in the Ames test showed that it does not induce gene mutations on *Salmonella typhimurium* strains TA 97, TA 98 and TA 100 at doses of 0.5-5000 μg / cup in conditions: without and in the presence of a metabolic activation system. In a cytogenetic test for accounting for chromosomal aberrations in mouse bone marrow cells, it was shown that a single and 5-fold intragastric administration of Treamide to mice in TD (0.71 mg/kg), as well as a single intragastric administration at a dose of 1/5 of LD50 ( 1000 mg/kg) does not induce chromosomal aberrations in animal bone marrow cells. Thus, the active substance of Treamide does not have a mutagenic effect.

Evaluation of the carcinogenic activity of Treamide drug substance was carried out on the basis of the results of a battery of short-term screening tests, including the study of the mutagenic activity of Treamide drug and the study of its ability to induce DNA damage in bone marrow, liver, kidney and spleen cells of mice (DNA comet test). The study showed that Treamide does not induce DNA damage in the cells of the bone marrow, liver, kidneys and spleen of mice. Thus, Treamide does not have carcinogenic properties.

The study of the immunotoxicity of the pharmaceutical substance of Treamide included an assessment of the immunotropic potential after a single and 14-day intragastric administration. The study showed that Treamide does not have an immunotoxic effect.

- - 1. **Clinical researches**

Investigational drug Treamide was studied in humans in one phase I clinical trial (METS-TRE-01; approval of the Ministry of Health of the Russian Federation for conducting clinical trial No. 52 of January 25, 2016) in healthy volunteers (single and multiple doses).

The aim of this study was to study the safety profile, pharmacokinetics and tolerability of Treamide in single and multiple doses in healthy volunteers. The study had a double-blind design. The study included 3 consecutive cohorts of volunteers who received the study drug at a dose of 5 mg, 15 mg and 50 mg or placebo. The 5 mg and 15 mg cohorts included 5 volunteers each, and the 50 mg cohort included 10 volunteers. Treamide/placebo ratio in the 5 mg and 15 mg cohorts was 4:1, in the 50 mg cohort it was 8:2. Thus, 20 volunteers took part in the study, of which 4 volunteers took Treamide at doses of 5 mg, 15 mg and placebo, 8 volunteers took Treamide at a dose of 50 mg.

The study consistently examined the safety, tolerability and pharmacokinetics of Treamid. Volunteers received study drug once at a cohort dose and then, after a 6-day break, continued daily study drug at the same dose for 14 days.

Inclusion of volunteers in subsequent dose cohorts was possible only after a positive decision of the safety data monitoring committee.

Twenty safety population volunteers completed the study according to protocol, with no early dropouts. AEs were reported in volunteers from all dose cohorts and were not reported in volunteers who received placebo.

In general, Treamide showed a favorable safety profile and good tolerability in the study. No SAEs were noted during all phases of the study, **no AEs were related to study medication and did not require treatment, and all AEs resolved**. Four AEs rated as CTCAE 4.03 Grade 3 or 4 were reported in volunteers in the lowest laboratory dose cohort of 5 mg and were asymptomatic.

During the study, 13 AEs were registered in 7/16 (43.8%) volunteers who received Treamid:

- at a dose of 5 mg in 3/4 (75.0%) volunteers, 6 AEs were registered (1 AE "Chickenpox" of moderate severity, 2 AEs "Increased CPK levels" of severe severity, 2 AEs "Hyperkalemia" of moderate and severe severity, 1 AE "Hypernatremia" life-threatening severity);
- at a dose of 15 mg in 2/4 (50.0%) of volunteers, 5 AEs were registered (2 AEs "Indigestion" of mild severity, 1 AE "Discomfort in the epigastric region" of mild severity, 1 AE "Nausea" of mild severity and 1 AE " Increasing the level of creatine phosphokinase in the blood "of moderate severity;
- at a dose of 50 mg in 2/8 (25.0%) volunteers, 2 AEs were registered (1 AE "Hyperbilirubinemia" of moderate degree and 1 AE "Increased level of creatine phosphokinase in the blood" of moderate degree.

Significant differences in the main PK parameters with single and multiple dosing of Treamide were not observed. With an increase in the dose of Treamide from 5 to 15 mg, stationary C _max_ and AUC _0-t_ increase proportionally, however, at a dose of 50 mg, a deviation from linearity is observed: the values of C _max_ and AUC _0-t_ are lower than expected. With a single dose of the drug, the linearity of the pharmacokinetics of the drug Treamide is apparent, due, obviously, to an underestimated value of the average C _max_ and AUC _0-t_ in the 15 mg cohort on Day 1 due to subjects 204 and 205.

The values of dose-normalized C _max_ and AUC _0-t_ do not remain constant over the entire interval of the studied dose range - they decrease at a dose of 50 mg.

There is no pronounced dose dependence for _Tmax_. With a single dose, T _max_ averaged 1.3-2.5 hours, with multiple doses - 1.0 - 1.75 hours. For T1 / 2, there is a tendency to increase with increasing dose from 5 to 50 mg. With a single dose, T1 / 2 increased from 4.6 in the 5 mg cohort to 11.6 hours in the 50 mg cohort, with multiple doses, from 4.3 hours to 12.1 hours, respectively.

The cumulation factor (Fc) in the studied dose range of 5-50 mg is 1.15-1.17. Thus, with multiple doses of the drug once a day, the total exposure of the active substance of the drug Treamide in blood plasma increases by 20% compared with a single dose.

Thus, the data obtained during the clinical trial allow us to conclude that Treamide in daily doses of 5 mg, 15 mg and 50 mg has a favorable safety profile, good tolerability and can be recommended for further clinical trials.

- - 1. **Risk/benefit ratio**

This study is aimed at obtaining data on the efficacy and safety of Treamide when used in patients with fibrotic changes in the lungs after suffering COVID-19 pneumonia.

Treamide suppresses notcW1-mediated proliferation of progenitor cells, which leads to a decrease in the production of key profibrous cytokines (TGF-beta, IL-13), a decrease in the severity of the inflammatory response and suppression of lung tissue destruction at the initial stage of pathology development. Also, inhibition of notcW1-mediated proliferation under the action of Treamide leads to suppression of the synthesis of extracellular matrix components (fibronectin and type I collagen) and the deposition of fibrous masses in lung tissues. In addition, activation of the differentiation of progenitor cells leads to an increase in the number of mature epithelial and endothelial cells in the lung tissue and accelerates the processes of lung regeneration.

**Thus, it is assumed that Treamide will be effective in patients with fibrotic changes in the lungs after suffering from COVID-19 pneumonia and will provide an earlier and / or complete restoration of respiratory function and diffusive capacity of the lungs, which in turn will lead to a decrease or elimination of shortness of breath, including during physical activity**.

In the course of preclinical studies of general and specific toxicity for the most sensitive animal species - rabbits, the minimum dose without an observed negative effect (DBNOE) was determined - 23 mg / kg. In this case, the equivalent dose for a person with a body weight of 70 kg (EDH) is 519 mg.

In the conducted preclinical studies, Treamide did not have a toxic effect on the functional state of the organs and systems of the body of experimental animals; did not have a local irritating and allergenic effect; did not have mutagenic, carcinogenic and immunotoxic effects; did not have a toxic effect on reproductive function.

In a Phase I clinical trial, Treamide generally demonstrated a favorable safety profile and good tolerability. There were no SAEs during the study, no AEs were related to study medication or required treatment, and all AEs resolved. Most AEs were mild to moderate in severity; The 4 AEs, the severity of which corresponded to grade 3 or 4 according to CTCAE 4.03, were asymptomatic and did not require discontinuation of the drug or treatment.

Based on the AEs reported in the Phase I clinical trial, changes in laboratory parameters (including CPK, bilirubin, and electrolytes) will also be monitored in this trial.

Prescribing Placebo to patients in this study can be considered justified, since at present no specific pharmacotherapy for patients after COVID-19 pneumonia has been described, including as part of pulmonary rehabilitation [16].

Only those patients who agree to use adequate methods of contraception during the entire study period and for 3 months after its completion will be included in this study.

Thus, the overall risk/benefit ratio appears to be favorable for participants in this clinical trial.

1. POPULATION SELECTION
   1. **Study population**

The study is planned to include 60 patients with fibrotic changes in the lungs after suffering from COVID-19 pneumonia.

To be included in this clinical trial, patients must meet the following inclusion/exclusion criteria.

- 1. **Inclusion Criteria**

To participate in the study, patients must meet the following criteria:

1. Signed Patient Information Sheet and informed consent form for participation in the study.
2. Men and women aged 18 to 75 inclusive.
3. Fibrotic changes in the lungs after suffering COVID-19 pneumonia:
4. The diagnosis of COVID-19 in history was confirmed by a positive qualitative analysis of SARS-CoV-2 RNA by PCR;
5. The appearance of the first symptoms of COVID-19 no more than 2 months before screening;
6. Fibrotic changes in the lungs, characteristic of COVID-19, were confirmed by chest CT at screening.
7. Negative test for COVID-19 at screening (confirmed).
8. Severity 2 (moderate) or 3 (severe) on the mMRC Breathlessness Scale at screening and randomization.
9. Decreased lung function FVC and/or DLCO < 80% predicted at screening.
10. Consent of patients to use adequate methods of contraception during the entire study and within 3 months after its completion. Adequate methods of contraception include the use of:

- oral or transdermal contraceptives;
- condom or diaphragm (barrier method) with spermicide;
- intrauterine device.
  1. Non-inclusion Criteria

A patient will be considered ineligible for the study if one of the following criteria is met:

1. Women who are pregnant or breastfeeding or planning to become pregnant during a clinical study; women of childbearing potential (including those who are not surgically sterilized and who are less than 2 years postmenopausal) who do not use adequate contraceptive methods.
2. The use of invasive mechanical ventilation (iALV), plasma transfusion (including convalescent plasma) and other blood components during COVID-19 therapy.
3. History of chronic respiratory disease, including idiopathic pulmonary fibrosis (IPF), asthma, chronic obstructive pulmonary disease (COPD), or pulmonary hypertension, diagnosed prior to COVID-19.
4. Severe cardiovascular disease at present or within 6 months prior to screening, including: NYHA class III or IV chronic heart failure, clinically significant ventricular arrhythmias (ventricular tachycardia, ventricular fibrillation), unstable angina pectoris, myocardial infarction, heart and coronary surgery, significant valvular heart disease, uncontrolled arterial hypertension with systolic blood pressure > 180 mm Hg. and diastolic blood pressure > 110 mmHg, pulmonary embolism (PE) or deep vein thrombosis.
5. Nephrotic syndrome, moderate to severe chronic renal failure, or significant kidney disease with creatinine > 1.5 mg/dL (132 µmol/L) or GFR < 60 mL/min at screening.
6. Cirrhosis of the liver in history; an increase in alanine aminotransferase (ALT) and / or aspartate aminotransferase (ACT) by 3 or more times from the upper limit of normal (ULN) at screening; an increase in the level of total bilirubin by 2 or more times from ULN at screening.
7. Hemoglobin level < 90 g/l at screening.
8. Severe diseases of the central nervous system, including a history of seizures or conditions that can lead to their development; stroke or transient ischemic attack within 6 months prior to screening; traumatic brain injury or loss of consciousness within 6 months prior to screening; a brain tumor.
9. Signs of significant uncontrolled comorbidity, such as disorders of the nervous system, kidneys, liver, endocrine system and gastrointestinal tract, which, in the opinion of the Investigator, could prevent the patient from participating in the study.
10. Malignant neoplasms requiring chemotherapy within 6 months prior to screening.
11. History of HIV infection.
12. Prostate cancer or benign prostatic hyperplasia (BPH) with a residual urine volume of more than 100 ml in history in men.
13. Hypersensitivity or intolerance to any of the components of the study drug.
14. Participation in other clinical trials within 2 months prior to screening.
15. Taking the following drugs: bronchodilators, anticholinergics, corticosteroids, cytostatics, colchicine, cyclosporine A, interferon-γ-1b, bosentan, macitentan, etanercept, sildenafil, imatinib, n-acetylcysteine, warfarin, ambrisentan, nintedanib, pirfenidone 1 month before screening [17].
16. inability to read or write; unwillingness to understand and follow study protocol procedures; non-compliance with drug regimens or procedures that, in the opinion of the Investigator, may affect the results of the study or patient safety and prevent the patient from continuing to participate in the study; any other medical or serious psychiatric condition that makes the patient unsuitable for participation in the clinical study, limits the eligibility of obtaining informed consent, or may affect the patient's ability to participate in the study.
    1. **Exclusion of patients from the study after randomization**

If the patient's condition deteriorates due to increased respiratory symptoms, decreased lung function, or episodes of acute respiratory deterioration, the patient will be removed from the study and, if necessary, admitted to a hospital for medical care. Criteria for early withdrawal of patients from the study are presented in Section 6.15.

Pulmonary fibrosis progression is defined by the occurrence of any of the following criteria [17]:

- relative reduction in FVC by ≥ 10%;
- a relative reduction in FVC ranging from ≥ 5% to < 10% and a relative reduction in DLCO by > 15%.

1. STUDY DRUG
   1. Description of study drug

| Investigated drugs: | Treamide or placebo | | |
| --- | --- | --- | --- |
| Drug composition | *Active ingredient:* Treamide (ХС268БГ) - 50 mg. Placebo - 0 mg. | |  |
|  |  | *Excipients:* |  |
|  |  | cellulose microcrystalline |  |
|  |  | pregelatinized starch |  |
|  |  | Sodium starch glycolate |  |
|  |  | Talc |  |
|  |  | Magnesium stearate |  |
|  |  | **Colloidal silicon dioxide** |  |
|  |  | *Shell:*  Opadry II white [hypromellose 34.0%, lactose monohydrate 28.0%, titanium dioxide 26.0%, macrogol 12.0%] |  |
| Pharmaceutical form | Film-coated tablets | | |
| Appearance: | Tablets are round, biconvex, film-coated white. Tablet core white or almost white | | |
| **Packaging.** | Blister Alu/Alu, №10 | | |
| Method of administration: | Orally, once a day, in the morning, 30 minutes before breakfast | | |
| Manufacturer: | JSC IIHR, Russia,  OCPC CJSC, Russia  (by order of PHARMENTERPRICES LLC, Russia) | | |
| Storage conditions: | In a place protected from light, at a temperature not exceeding 25 ° C. Keep out of the reach of children | | |

- 1. **Study drug administration**

Study drug will begin at Visit 2, Week 0. Patients will receive either Treamide or Placebo for 4 weeks. Study therapy will be blinded by placebo masking. Patients will receive individual packs of study medication containing Treamide 50 mg or placebo.

Patients will be instructed to take study drug 1 tablet once daily in the morning 30 minutes before meals.

Patients will receive their first dose of study drug at the study site. Subsequent admission will be carried out independently. On Visit Day 4, the drug will be taken after blood sampling for general clinical and biochemical tests. Last dose of study drug on the day before Visit 6, Week 4.

- 1. **Method of allocation of patients to treatment groups**

Will be randomized into two groups in a 1:1 ratio using the IWRS system. Each group will eventually include 30 patients. Randomization will be stratified by

1) the presence or absence of risk factors (age ≥ 60 years and / or the presence of concomitant chronic diseases)

2) and the initial degree of lung damage according to CT data (CT 1, CT 2, CT 3 and CT 4)

The stratification method will be described in more detail in the randomization protocol.

After randomization, patients will receive the required amount of study drug until the next visit, according to the treatment group. Instructions for randomization and operation of the IWRS system will be provided to the investigator prior to the start of the study.

- 1. **Blinding of study drug**

This study is double-blind. Clinical center staff and patients will not know which therapy is prescribed for each individual patient. Blinding will be provided by placebo masking (each patient will take tablets corresponding to Treamide 50 mg or placebo) and dispensing the drug by IWRS by individual package number.

- 1. **dazzle**

The code for a specific patient can only be opened in an emergency if knowledge of the patient's specific therapy could affect his treatment. To open the code, there will be a special procedure for requesting the code through IWRS. The reason for opening the code should be described in detail in the patient's primary documentation. The need to blind the therapy of a particular patient should, if possible, be discussed with the Medical Monitor in advance or notify him no later than 24 hours after opening the code.

- 1. **Compliance with the therapy**

The first dose of study drug must be taken by the patient at the study site under the supervision of the Investigator (Visit 2, Week 0). Compliance with the recommendations for self-administration of investigational drug will be verified by the Investigator at visits based on the results of accounting for the returned investigational drug.

Compliance calculation will be carried out on visits according to the formula:

Compliance = (N issued - N returned) / N calculated x 100%

where N is issued. = number of tablets dispensed, N return. = number of returned tablets, N calc. = Estimated number of tablets the patient should have taken since the previous visit.

Patient compliance should be between 80% and 120%. If necessary, an additional conversation should be held with the patient about the correct administration of the drug. In the event of repeated violation of the study drug regimen and/or failure to follow the Investigator's recommendations, the patient must be early withdrawn from the study as agreed with the Sponsor or its representative.

- 1. **Concomitant therapy**

Concomitant therapy for acute or chronic conditions may be continued as indicated. All concomitant drugs and biologically active additives (BAA) must be registered in the primary documentation and in the eCRF.

The name (preferably the name of the active substance), dosage, frequency of administration, route of administration, indication for use (including the underlying disease, concomitant condition, adverse event or prophylaxis), the start and end date of the concomitant drug should be recorded in the primary documentation and in eCRF. The study should reflect any changes in concomitant therapy. If concomitant therapy is ongoing at the end of the study, this should be noted in the eCRF.

- 1. **Prohibited Therapy**

Throughout the study, patients **should not** receive other study drugs, as well as drugs from the following groups: bronchodilators, anticholinergics, glucocorticosteroids, cytostatics, colchicine, cyclosporine A, interferon-γ-1b, bosentan, macitentan, etanercept, sildenafil, imatinib, n -acetylcysteine, warfarin, ambrisentan, nintedanib, pirfenidone [17].

- 1. **Packaging and labeling of investigational product and concomitant therapy**

The study drug will be provided by the Sponsor. The study drug will be packaged in aluminum blisters and labeled in accordance with applicable law and applicable regulations.

- 1. **Storage and record keeping of the study drug**

The investigator will be responsible for arranging the storage of the study product at the study site throughout the study. Limited access conditions and appropriate temperature conditions must be provided. Temperature control should be carried out using thermometers that record the minimum and maximum temperatures for the reporting period. Temperature monitoring data should be recorded regularly in a temperature log. At the end of the study, after the final count has been made, the study drug must be returned to the Sponsor or its representative or destroyed at the center at the discretion of the Sponsor.

The investigator will be responsible for maintaining investigational product records to ensure they are properly received, stored, distributed and returned.

1. **DESCRIPTION OF PROCEDURES**

All research procedures will be carried out in accordance with the regulatory requirements and recommendations of the Good Clinical Practice Guidelines of the International Conference on Harmonization (ICH GCP) and the Eurasian Economic Union (EAEU), the principles set forth in the Declaration of Helsinki, as well as in accordance with current legislation and applicable regulatory requirements of the Russian Federation. The Investigator must agree to conduct monitoring, audits and inspections in the research center and at any time, upon request, provide direct access to the study materials to the Sponsor and its representatives, the Independent Ethics Committee and authorized bodies.

- 1. **Informed consent**

Informed consent will be obtained at screening prior to any study-related procedures. If screening procedures are carried out as part of a patient’s discharge from the hospital after treatment for COVID-19 pneumonia, it is necessary to provide for the procedure and signing of the informed consent form in a “clean zone” to ensure the safety of primary documentation and prevent its contamination.

- 1. **Patient Registration**

Each patient who signs the Patient Information Sheet and the informed consent form will be assigned an individual registration number consisting of four digits 0-0 O: the first two digits correspond to the center number, the second two digits - the patient's serial screening number in this center (01, 02, 03 etc.). Instructions for registering patients will be provided to the Investigator prior to the commencement of the study.

- 1. **Demographics and medical history**

To assess whether patients meet inclusion/non-inclusion criteria, patient demographics (gender, date of birth/age, race) and a complete medical and epidemiological history, duration of disease symptoms, significant acute and chronic diseases and conditions (e.g. menopause), surgical interventions, allergic reactions and smoking status. All new diagnoses and conditions identified at the screening (including the results of laboratory and instrumental studies) should be attributed to the patient's medical history.

- 1. **Physical examination**

Physical examination includes evaluation of general appearance, condition of the skin and mucous membranes, eyes, ears, nose and throat, as well as an examination of the cardiovascular, musculoskeletal, respiratory system, gastrointestinal tract and nervous system.

Clinically significant changes in the physical examination compared to baseline should be considered as AEs and recorded appropriately in the primary documentation and in the eRCI.

- 1. **Vital signs and measurement of blood oxygen saturation (SpO2)**

This examination includes the measurement of axial body temperature, as well as blood pressure (BP), pulse, respiratory rate and blood oxygen saturation (SpO2) in a sitting position after 10 minutes of rest.

Clinically significant changes in vital signs from baseline values should be regarded as AEs and recorded accordingly in the primary documentation and in the eCRF.

- 1. **Electrocardiography**

Electrocardiography (ECG) in 12 leads is performed with the patient in the supine position after 10 minutes of rest. The electrodes should be placed on the same points throughout the study. To record an ECG, you must use an electrocardiograph with automatic interval assessment. The following intervals will be assessed on the ECG: HR, PQ, QRS, QT, QTc(B).

Parameter QTc( B ) will be calculated automatically in eCRF according to the formula:

Bazett's formula: QTc (B) = QT / √RR

The RR parameter for calculating QTc( B ) will also be calculated automatically in eCRF using the formula:

|  | *RR* | = | 60 |
| --- | --- | --- | --- |
|  |  |  | HR |

The Investigator must review the ECG record, in case of deviations, evaluate their clinical significance, sign and date the conclusion. Significant changes on the ECG should be rechecked, repeated studies should be carried out.

Clinically significant changes in the ECG compared to baseline should be regarded as AEs and recorded accordingly in the primary documentation and in the eRCI.

- 1. **CT scan of the chest**

Computed tomography (CT) of the chest will be used to assess lung involvement. The procedure will be performed at each center according to standard CT protocol. The assessment will be performed by a qualified radiologist at the center.

The assessment will be carried out in accordance with the classification according to the degree of changes detected.

| **Computed tomography of the chest** | | | | |
| --- | --- | --- | --- | --- |
| **The main manifestations of viral pneumonia** | **Frosted glass** | **Consolidation** | **Other additional features** | **Involvement of the lung parenchyma** |
| **CT 0**  The norm and absence of CT signs of viral pneumonia against the background of a typical clinical picture and a relevant epidemiological history | No | No | No | No |
| **CT1**  Ground-glass opacities Involvement of the lung parenchyma < 25% Or absence of CT findings with typical clinical presentation and relevant epidemiological history | Yes | no, single small size | Single zones of small size of reticular changes | < 25% |
| **CT2**  Ground glass seal zones  Parenchymal involvement  lung 25-50% | Yes | Yes  single | Single zones of small reticular changes, there may be a reverse "halo" | < 50% |
| **CT3**  Ground-glass compaction zones Consolidation zones Involvement of the lung parenchyma 50-75% Increase in the volume of the lesion up to 50% in 2448 hours against the background of respiratory disorders, if the studies are performed in dynamics | Yes | yes, possibly massive | Areas of reticular changes, may be reversible "halo" May be air bronchogram symptom Minimal hydrothorax not associated with pneumonia | 50-75% |
| **CT 4**  Diffuse ground-glass thickening of the lung tissue and consolidation in combination with reticular changes Hydrothorax (bilateral, predominant on the left) Involvement of the parenchyma lung ≥ 75% | Yes | Yes, dominated by massive | Areas of reticular changes  be reversed "halo", Air bronchogram symptom Hydrothorax predominantly on the left | ≥ 75% |

The imaging records, description, and CT report should be evaluated by the Investigator. Changes to the CT must be properly recorded in the primary documentation and in the eCRF.

- 1. **Analyzes performed in the laboratory**

All laboratory studies provided for in the protocol will be performed in the local laboratory of the research centers.

Before the start of the study, the local laboratory of each research center will be required to provide the required certificates and laboratory standards. Upon receipt of the laboratory report, the Investigator will have to review it, in case of deviations of the indicators from the normal values, assess their clinical significance, sign and date the report. Clinically significant changes in laboratory values from baseline should be considered an AE (eg, if laboratory abnormalities result in medical intervention, treatment delay, treatment interruption, hospitalization, or complications).

- - 1. **Laboratory parameters**

Necessary laboratory tests will be carried out at certain visits in accordance with the Procedure Schedule. Blood samples for analysis will be taken on an empty stomach in accordance with the center's regulatory procedures.

Table 2 presents the blood and urine laboratory values that will be assessed in this clinical trial.

Table 2. List of indicators of laboratory tests of blood and urine

| **- Complete Blood Count** | **Blood chemistry;** | | | **Special Studies** | **General urine analysis** |
| --- | --- | --- | --- | --- | --- |
|  | General | Hepatic status | Kidney status |  |  |
| Hemoglobin  Hematocrit  Red blood cells  White blood cells  Neutrophils  Lymphocytes  Monocytes  Eosinophils  Basophils  Platelets  ESR | Glucose  Total protein  Sodium  Potassium  Chlorine  CPK | Total bilirubin  Alkaline phosphatase  ALT  AST  GGT | Blood urea nitrogen  Serum creatinine  GFR (according to the Cockcroft-Gault formula) | Qualitative determination of SARS-CoV-2 RNA | General properties: color, transparency, specific gravity, pH, protein, glucose, bilirubin, urobilinogen, ketone bodies, nitrites, hemoglobin  Sediment microscopy: epithelium, erythrocytes, leukocytes, casts, bacteria, salts |

*rapid test or enzyme immunoassay

Abbreviations: ESR - erythrocyte sedimentation rate, CPK - creatine phosphokinase, ALT - alanine aminotransferase, AST - aspartate aminotransferase, GGT - gamma-glutamyl transferase, GFR - glomerular filtration rate

A pregnancy test is only given to women of childbearing potential (including women less than two years of menopause). The test is performed directly at the center using a test strip in a urine sample.

- - 1. **PCR for SARS-CoV-2 RNA**

Qualitative determination of SARS-CoV-2 RNA will be carried out by PCR in the local laboratory of the center, or will be sent by the centers to laboratories accredited by Rospotrebnadzor in accordance with the routine practice of the center. The material for the study will be a swab from the nasopharynx and / or oropharynx according to the standard technique.

A swab from the nasopharynx or oropharynx (pharynx) is taken with a sterile swab, which, after taking the material, is placed in a sterile plastic tube with a transport medium (taking into account the manufacturer's recommendations, the test systems / reagent kits used).

Confirmation of two consecutive negative PCR results at least 24 hours apart is required for inclusion in the study. In the case of previously confirmed elimination of the SARS-CoV-2 virus according to medical records, one determination of SARS-CoV-2 RNA by PCR with a negative result within the study will be sufficient.

- - 1. **Pharmacokinetic study**

All patients will have a blood sample taken on the morning of Week 0, Week 2 and Week 4 before their next dose of study drug to determine the concentration of the active substance XC268BG and calculate the PK parameter Residual concentration Ctrough.

Receipt, sample preparation, storage and shipment of samples for PK testing will be carried out in accordance with regulatory requirements and laboratory guidelines.

To evaluate pharmacokinetic parameters, HPLC/MS/MS will be used using a QTRAP 5500 system (Applied Biosystems) with an Agilent 1290 chromatograph (Agilent Technologies).

Investigational therapy blinding will be performed prior to conducting the PK study and after closing the database. PK analysis for patients receiving placebo will only be performed if there is doubt about the correctness of the blinding procedure. To confirm the absence of an investigational drug in the blood plasma of patients receiving placebo, an analysis can be performed at points corresponding to the calculated Tmax values.

- - 1. **Receipt, preparation, storage and dispatch of biosamples**

Receipt, preparation, storage and shipment of biosamples will be carried out in accordance with regulatory requirements and laboratory guidelines.

The total volume of blood collected from each patient during scheduled study visits will be approximately 102 ml.

Patients who will have additional PK sampling at Week 0 will have approximately 102 mL of blood collected during their scheduled study visits.

| **Blood volume for various research procedures, ml** | Screening | **Investigational Therapy** | | | | | **Follow-up** |  |
| --- | --- | --- | --- | --- | --- | --- | --- | --- |
| Visit (V) | V 1 | V 2 | V 3 | V 4 | V 5 | V 6 | V 7 |  |
| Week (W) | W-2 | W0 | W1 | W2 | W3 | W4 (ET) | W6 | Total, ml: |
| - Complete Blood Count | 6 | 6 |  | 6 |  | 6 | 6 |  |
| Blood chemistry; | 12 | 12 |  | 12 |  | 12 | 12 |  |
| PK study (all patients) |  | 4 |  | 4 |  | 4 |  | 102 |

- 1. **mMRC (modified Medical Research Council) breathlessness scale**

The severity of breathlessness will be assessed using the mMRC Breathlessness Scale (Appendix 1). The patient must choose one of the five responses presented on the scale, characterizing the shortness of breath that he is currently experiencing. Scores on the scale characterize the severity of dyspnea from 0 (no dyspnea) to 4 (very severe dyspnea). Each point on the scale is accompanied by explanations that allow the patient to make the most accurate choice.

The examiner must review the scale completed by the patient and sign and date the form. Clinically significant changes in the severity of dyspnea from baseline should be regarded as AEs and recorded appropriately in the primary documentation and in the eRCI.

- 1. **6 Minute Walk Test (6MWD, Borg Scale)**

The 6-minute walking test is based on measuring the walking distance (6-minute walking distance - 6MWD) with turns along a long straight corridor (≥30 m), at the patient's own pace. Allows you to evaluate submaximal exercise tolerance, which corresponds to the ability to perform daily work.

To assess dyspnea on exertion, the Borg Scale is used, which is assessed by the patient immediately before and after the end of the test. This is a 12-point ordinal scale (from 0 to 10 with an intermediate value of 0.5). Before starting 6MWD, you need to show the scale to the patient and ask him to determine the degree of respiratory discomfort and general fatigue on it. At the end of testing, the patient should be reminded of their initial scores and asked to re-evaluate these symptoms.

Detailed instructions for the 6 Minute Walk Test and a sample Borg Scale are provided in Appendix 2.

The test should be stopped immediately if the patient develops: chest pain, severe shortness of breath, spasm of the muscles of the lower extremities, imbalance (stability), profuse sweating, sudden pallor, or a decrease in hemoglobin oxygen saturation (when using a pulse oximeter).

Clinically significant changes in the patient's condition during the test should be regarded as AEs and recorded accordingly in the primary documentation and in the eRCI.

- 1. **Questionnaire KBILD**

The King Interstitial Lung Disease Scoring Inventory (KBILD) is a short 15-item questionnaire. For each question, the patient must choose one of seven possible answers. A sample KBILD questionnaire is provided in Annex 3.

- 1. **Spirometry (FEV1, FVC, FEV1/FVC)**

Pulmonary function will be assessed using regularly calibrated spirometry equipment in accordance with the Russian Respiratory Society Federal Clinical Guidelines for the Use of Spirometry, 2013 [41]

Spirometry readings will include:

- Formed expiratory volume in 1 second (FEV1)
- Forced vital capacity (FVC)
- FEV1/FVC

Spirometry should be taken in the morning at about the same time during the study. In the case of an upper respiratory tract infection, a routine pulmonary function assessment should be rescheduled and performed no earlier than 7 days after resolution of symptoms.

Lung function estimates should use height measured at screening and body weight measured directly at the visit.

Tests must meet quality criteria for technical acceptability and reproducibility. To obtain reproducible results, at least three technically satisfactory maneuvers must be obtained that meet the acceptance criteria; the best (greatest) result must be registered in the CRF.

The investigator must review the spirometry report, evaluate the test results, and sign and date the report. Changes in spirometry values from baseline, regarded as AEs, should be recorded appropriately in the primary documentation and in the CRF.

- 1. **Body plethysmography (DLCO, TLC, FRC)**

Evaluation of lung function using a body plethysmograph in accordance with the guide "Functional diagnostics in pulmonology" ed. Z.R. Aisanova, A.V. Chernyaka, 2016 [42].

Body plethysmography allows you to fully determine all respiratory volumes, including those that cannot be obtained with spirography. The procedure is carried out using a body plethysmograph, which includes a body camera (where the patient sits) with a pneumotograph and a computer that displays data.

In this study, total lung capacity (TLC) and functional residual lung capacity (FRC) will be assessed. As part of the body plethysmography procedure, the lung diffusing capacity for carbon monoxide (DLCO) will also be assessed.

Tests must meet quality criteria for technical acceptability and reproducibility. To assess the DLCO parameter, a hemoglobin value is required, so the results of a complete blood count should be available at the time of the body plethysmography.

The Investigator will review the indicators, in case of deviations, evaluate their significance, sign and date the conclusion. Changes in DLCO, TLC, or FRC from baseline that are considered AEs should be recorded appropriately in primary documentation and in the eCRF.

- 1. **Assessment of adverse events (AEs)**
     1. **Definitions of AE**

**Adverse event (AE)** - Any adverse medical event identified in a clinical trial subject after use of a medicinal product, which may not have a causal relationship with its use. An AE can be any adverse symptom (including a laboratory abnormality), complaint, or disease.

In this study, AE registration will be carried out from the moment the patient signs the informed consent form and up to 30 days after the patient's last visit to the study center or the last protocol procedure. All AEs will be monitored during the investigational therapy phase; at the follow-up stage - only SAE.

**Adverse reaction (HP)** - all adverse reactions associated with the use of any dose of the study drug. In other words, if there is at least a minimal possibility of a causal relationship between the study drug and the AE, i.e. relationship is not excluded.

**Serious Adverse Event (SAE) and/or Serious Adverse Reaction (SAR)** - Any adverse medical event that, regardless of study drug dose:

- led to death;
- poses a threat to life;
- requires hospitalization or its extension;
- has resulted in permanent or significant disability or disability, or
- is a congenital anomaly or birth defect
- other important medical cases

Significant medical events that do not pose an immediate threat to life, do not lead to death or hospitalization, but put the patient at risk or require interventions aimed at preventing the above outcomes, can also be classified as SAE/SAR. Examples of such phenomena can be allergic bronchospasm, convulsions, malignant neoplasms.

**NOTE:** Hospitalization for social reasons, visits to a day hospital, and hospitalization or surgery planned prior to enrollment in a study for treatment of a preexisting condition are not considered SAEs.

**Unanticipated adverse events** - An adverse event whose nature or severity is not consistent with known product information (eg, an Investigator's Brochure for an unregistered investigational product, or a package insert for an approved medicinal product). This group also includes adverse events that are mentioned in the Investigator's Brochure as being characteristic of this drug class, or expected due to the pharmacological properties of the study drug, but not previously observed.

- - 1. **Pregnancy**

Pregnancy is not an AE, but is an event requiring urgent reporting to the Sponsor. In case of pregnancy of the patient or partner of the study patient, the course of pregnancy will be monitored up to 30 days after its resolution. Relevant information will be recorded in the primary documentation, in the eCRF, as well as in the pregnancy registration form. Reporting the pregnancy of a patient/partner of a study patient must be sent to the Sponsor within the same time frame as reporting an SAE.

Throughout the study and for 3 months after its completion, patients, including men and women of childbearing potential, must use adequate methods of contraception (oral or transdermal contraceptives; condom or diaphragm (barrier method) with spermicide; intrauterine device).

- - 1. **AE reporting**

During each visit and telephone contact, patients should report any AEs in response to open-ended, non-leading questions (eg, "How have you been since your last visit?"). For each patient-reported AE, the investigator should collect and record in the primary documentation and in the eCRF all relevant information, including diagnosis or symptoms, date of onset and end date, outcome, severity, presence of severity criteria, circumstances that may indicate a possible association investigational drug or concomitant therapy, underlying diseases or comorbid conditions, study procedures or other causes, investigational drug activity, drug therapy, medical interventions, laboratory and instrumental findings for AEs, and other circumstances that will help maximize fully describe the event.

An increase in the severity of an ongoing AE should be considered as a new AE. The date of the change in severity will be considered the start date of the new AE, and the previous day will be considered the end date of the original AE. The start date of the SAE is the day the severity criterion occurs. The previous condition, if applicable, should be reported as a non-serious AE.

If the AE is serious, the Investigator must also complete the SAE report form and send it to the Sponsor in a timely manner. New SAE information that becomes known at a later date must be registered and communicated to Sponsor in the same manner and within the same time frame. Also, the Sponsor must be provided with certified copies of the primary documents for this SAE (discharge summary, autopsy data, death certificate, etc.).

AEs should be observed and reported from the time the patient signed the informed consent until 30 days after the last visit or procedure related to the study. All AEs are monitored during the investigational therapy phase. During the follow-up phase, only SAEs are monitored. AEs and SAEs should be monitored until resolution or stabilization of the patient's condition.

The monitor is responsible for verifying that the eCRF data and SAE reporting forms are consistent with the primary documentation.

- - 1. **Severity assessment**

The severity of the AE will be assigned according to the criteria presented in Table 3 (corresponding to the NCI-CTCAE classification). The use of the classifier itself for grading the severity of AE is not required.

Table 3. AE severity assessment

| **AE Severity** | | **Definition (corresponds to one or more**  **criteria)** |
| --- | --- | --- |
| Degree 1 | Mild | - Asymptomatic or mild symptoms - Only clinical or diagnostic observation is needed - Medical intervention not indicated |
| Degree 2 | Moderate | - Minimal, local or non-invasive intervention indicated - Limits age-appropriate important   daily activity |
| Degree 3 | Severe/Medically Significant Non-Life Threatening AE | - Hospitalization or its extension is indicated - AE leading to disability/disability - Limits the ability for daily self-care |
| Degree 4 | Life-threatening consequences | - Requires immediate medical attention |
| Degree 5 | Death associated with AE | - AE with fatal outcome |

- - 1. Association of AEs with study drug

For each AE, the Investigator should evaluate its possible association with the investigational product according to the categories presented below - Table 4.

Table 4. Assessing causality with study drug use

| Category of AE | Definition |
| --- | --- |
| 1. Related | - occurs in a certain significant time interval after the use of the study drug; - accompanies a known reaction to the use of the investigational drug; - resolved after discontinuation of the drug and - reoccurs after restarting study drug (depending on the nature of the AE, repeat use may not be possible) |
| 2. Possibly related | - Occurs within a significant time interval after administration of the study drug - accompanies a known reaction to the use of the investigational drug or drugs of the same pharmacological group - could also be related to the patient's disease or condition, concomitant therapy or procedure |
| 3. Unlikely related | - there is sufficient information indicating that there is no causal relationship with the investigational product - possible other cause |
| 4. Not connected | - no temporal association with study drug (eg, AE occurring in a patient who did not receive study drug) - another cause is known |

- - 1. **Reporting SAEs/Pregnancy**

All SAEs, regardless of severity and association with study drug, as well as pregnancy of the patient/partner of the study patient, must be reported to the Sponsor or his representative within 24 hours from the moment the Investigator receives information about their occurrence. SAEs must be entered into the eCHR in the SAE reporting section of the form. Pregnancy data must also be entered in the eCRF in the Pregnancy Reporting section of the form. If necessary, the Investigator may contact the Medical Monitor for advice or clarification.

Reporting SAEs/Pregnancy

Company: LLC "IFARMA"

Medical Monitor: Kasyanova O.V.

Phone: +7 (495) 276-11-43

Mobile: +7 (925) 421-52-23

*Fax +7(495) 276-11-47*

Email e-mail: SAE@ipharma.ru

The Investigator must instruct each patient to promptly report the occurrence of an SAE or pregnancy during the study and within 30 days of the last study site visit or last study procedure. The investigator should record on the SAE/pregnancy report form all available information about the event. Each SAE reporting form should include, at a minimum, the following data: patient information, event name, severity criteria, association with investigational drug, and outcome at the time of reporting.

- - 1. **Withdrawal of patients from the study**

The patient must be withdrawn from the study if deemed medically necessary by the Investigator or if the patient has withdrawn consent (reasons for early withdrawal are described in Section 6.15). If possible, the Investigator should first discuss the exclusion of the patient with the Medical Monitor. Otherwise, the investigator must discuss the patient's exclusion with the Medical Monitor within 24 hours.

- - 1. **Procedures for dealing with emergencies**

The investigator is responsible for obtaining information about all urgent medical conditions of patients during the course of the study. The text of the Patient Information Sheet and the informed consent form contains the contact information of the Investigator. Patients will be advised to contact the Investigator in the event of any medical emergencies during the study.

- - 1. **Registration deadlines for AEs**

In this study, AE registration will be carried out from the moment the patient signs the informed consent form and up to 30 days after the patient's last visit to the study center or the last protocol procedure.

- - 1. **Expectancy**

Please refer to Section 3.4 "Previous Application Experience". At the moment, there have been no specific adverse reactions to Treamide that could be attributed to the expected adverse reactions.

- - 1. **Clinically Significant Laboratory Abnormalities**

When evaluating the results of laboratory tests, the Investigator may not classify single violations of the reference limits of laboratory parameters as deviations if they are not accompanied by any clinical manifestations, do not require additional examination or treatment, and are not confirmed by the values of interrelated laboratory parameters (clinically insignificant deviations).

Any clinically significant laboratory abnormalities noted at screening should be attributed to the patient's medical history. After screening, new or worsening clinically significant laboratory abnormalities should be reported as AEs in the primary documentation and in the CRF.

- 1. **Early withdrawal of patients from the study or withdrawal of the study drug**

The patient has the right to refuse participation in the study at any stage and without explanation.

A patient should be excluded from a clinical trial if:

- Investigator considers it medically necessary
- The patient withdraws his consent
- Patient fails to comply with prescribed doses of study drug or study-related procedures
- An AE or SAE has occurred that could adversely affect the safety and well-being of the patient
- Decrease in FEV1 by > 15% of baseline or < 50% of predicted
- Lower respiratory tract infection

The following reasons warrant immediate discontinuation of the study drug:

- Patient started another study drug
- The onset of pregnancy in the patient
- Significant protocol violations that may affect patient safety and the integrity of study data

The Investigator should, if possible, discuss the need for exclusion of the patient with the Medical Monitor in advance or communicate the exclusion of the patient within 48 hours. The reason for exclusion of the patient should be indicated in the primary documentation and in the CRF. If a patient withdraws from the study for more than one reason, only the underlying reason should be reported. The reason for withdrawal of consent by a patient due to an AE or SAE should be the occurrence of an AE or SAE.

In addition, the Sponsor has the right to terminate the study at any time. The Investigator has the right to terminate the study at any time for medical or regulatory reasons. The completion of the study should only take place after mutual consultation between the Investigator and the Sponsor.

If the study is terminated early, all patients must undergo early termination visit procedures and all study materials must be returned to the Sponsor or its representative.

Follow-up for AEs, SAEs, and pregnancy ongoing at the time of termination of the study should be carried out according to the Protocol, unless a different safety follow-up procedure is established by mutual agreement between the Investigator and the Sponsor.

- 1. **Validity of measurements**

1. Evaluation of the efficacy and safety of therapy for fibrotic changes after suffering COVID-19 pneumonia was proposed in accordance with the Interim Guidelines “Medical Rehabilitation in New Coronavirus Infection (COVID-19)” and recommendations for conducting clinical trials in patients with IPF [13,16]. Double-blind placebo control will allow to maximally objectify the obtained data on the efficacy and safety of the study drug.
2. RESEARCH PROCEDURES
   1. **Screening**

**(Visit 1, Week -2, Visit window -28 days)**

- Procedure for obtaining informed consent
- Collection of demographic data and medical history
- Physical examination
- Body weight, height, BMI
- Vital Signs Assessment
- Pulse oximetry with SpO2 measurement
- Assessment of the patient's condition using the mMRC Breathlessness Scale
- Nasopharyngeal and/or oropharyngeal swab for PCR for SARS-CoV-2 RNA
- Taking blood and urine samples for laboratory tests: general and biochemical blood tests, general urinalysis
- Pregnancy test for women of childbearing potential (including women less than two years of menopause)
- 12-lead ECG
- CT scan of the chest
- Spirometry (FEV1, FVC, FEV1/FVC)
- Body plethysmography (DLCO, TLC, FRC)
- Evaluation of inclusion / non-inclusion criteria
- Evaluation of AEs and concomitant therapy

**In the event that screening procedures are initiated as part of the patient's discharge from the hospital after hospitalization of a patient diagnosed with COVID-19 pneumonia, sufficient time should be allowed for the completion of quarantine measures (about 2 weeks) before assessing the extent of damage and lung function.**

The screening may use the results of examinations carried out as part of routine medical practice before obtaining informed consent (taking into account the window of the screening visit), except for the assessment on the mMRC Breathlessness Scale, CT of the chest, spirometry and body plethysmography with the definition of DLCO - these examinations should be carried out as much as possible close to the planned date of randomization (no earlier than 5 days before it).

After the Investigator receives the results of all studies and confirms compliance with all inclusion/non-inclusion criteria, patients will be included in the study.

- 1. **Study therapy period**
     1. **Visit 2, Week 0**
- Physical examination
- Body mass
- Vital Signs Assessment
- Pulse oximetry with SpO2 measurement
- Assessment of the patient's condition using the mMRC Breathlessness Scale
- Assessment of the patient's condition using the KBILD questionnaire
- 6 Minute Walk Test (6MWD, Borg Scale)
- Spirometry (FEV1, FVC, FEV1/FVC)
- 12-lead ECG
- Taking blood and urine samples for laboratory tests: general and biochemical blood tests, general urinalysis
- Obtaining a blood sample from all patients for the PK study of the C _trough_ parameter before taking the study drug
- Pregnancy test for women of childbearing potential (including women less than two years of menopause)
- Final assessment of inclusion/non-inclusion criteria
- Patient Randomization
- Distribution of the study drug
- Initiation of investigational therapy according to treatment regimen

•

- - 1. **Visit 3, Week 1, Visit window ± 1 day**
- Evaluation of AEs and concomitant therapy
- Vital Signs Assessment
- Body mass
- Pulse oximetry with SpO2 measurement
- Assessment of the patient's condition using the mMRC Breathlessness Scale
- Spirometry (FEV1, FVC, FEV1/FVC)
- Study drug return and compliance assessment
- Release of the investigational drug
  - 1. **Visit 4, Week 2, Visit window ± 2 days**
- Evaluation of AEs and concomitant therapy
- Physical examination
- Body mass
- Vital Signs Assessment
- Pulse oximetry with SpO2 measurement
- Assessment of the patient's condition using the mMRC Breathlessness Scale
- Assessment of the patient's condition using the K-BILD questionnaire
- 6 Minute Walk Test (6MWD, Borg Scale)
- Spirometry (FEV1, FVC, FEV1/FVC)
- 12-lead ECG
- Body plethysmography (DLCO, TLC, FRC)
- Taking blood and urine samples for laboratory tests: general and biochemical blood tests, general urinalysis
- Obtaining a blood sample from all patients for the PK study of the Ctrough parameter prior to taking the study drug
- Pregnancy test for women of childbearing potential (including women less than two years of menopause)
- Final assessment of inclusion/non-inclusion criteria
- Study drug return and compliance assessment
- Release of the investigational drug
  - 1. **Visit 5, Week 3, Visit window ± 1 day**
- Evaluation of AEs and concomitant therapy
- Vital Signs Assessment
- Body mass
- Pulse oximetry with SpO2 measurement
- Assessment of the patient's condition using the mMRC Breathlessness Scale
- Spirometry (FEV1, FVC, FEV1/FVC)

•

- Study drug return and compliance assessment
- Release of the investigational drug
  - 1. **Visit 6, Week 4, Visit window ± 2 days (End of therapy)**
- Evaluation of AEs and concomitant therapy
- Physical examination
- Body mass
- Vital Signs Assessment
- Pulse oximetry with SpO2 measurement
- Assessment of the patient's condition using the mMRC Breathlessness Scale
- 6 Minute Walk Test (6MWD, Borg Scale)
- Assessment of the patient's condition using the K-BILD questionnaire
- Nasopharyngeal and/or oropharyngeal swab for PCR for SARS-CoV-2 RNA

•

- Taking blood and urine samples for laboratory tests: general and biochemical blood tests, general urinalysis
- Obtaining a blood sample from all patients for the PK study parameter C _trough_
- Pregnancy test for women of childbearing potential (including women less than two years of menopause)
- 12-lead ECG
- CT scan of the chest
- Spirometry (FEV1, FVC, FEV1/FVC)
- Body plethysmography (DLCO, TLC, FRC)
- Study drug return and compliance assessment

Thereafter, patients will continue treatment in accordance with applicable general guidelines.

- 1. **observation period.**
     1. **Visit 7, Week 6, Visit window ± 3 days**
- Evaluation of **SAEs** and concomitant therapy alone
- Physical examination
- Body mass
- Vital Signs Assessment
- Pulse oximetry with SpO2 measurement
- Assessment of the patient's condition using the mMRC Breathlessness Scale
- Taking blood and urine samples for laboratory tests: general and biochemical blood tests, general urinalysis
- Pregnancy test for women of childbearing potential (including women less than two years of menopause)
- 12-lead ECG
  1. **Unscheduled examinations**

At the discretion of the Investigator, unscheduled examinations may be performed on patients at any time during the course of the clinical trial for safety reasons, if a repeat examination or procedure is necessary. The Investigator can carry out the necessary procedures, including laboratory and instrumental studies. Unscheduled examinations should be registered in the primary documentation and in the eCRF. Conducting unscheduled examinations should not affect the schedule of scheduled examinations provided for in the protocol of this clinical trial.

- 1. **Early termination of the study**

Early termination of study participation after Visit 2 and at least one dose of study drug should be performed, if possible, at an early termination visit (EC). As part of an early end visit, if possible, follow the End of Therapy visit procedures (section 7.2.5).

1. **QUALITY ASSURANCE**

This clinical trial will be conducted in accordance with the Standard Operating Procedures (SOPs) of the Sponsor and/or its representative, the Good Clinical Practice Guidelines of the International Conference on Harmonization (ICH GCP) and the Eurasian Economic Union (EAEU), the principles set forth in the Declaration of Helsinki, and also in accordance with applicable law and applicable regulatory requirements of the Russian Federation. Compliance will be ensured through audits of study sites and study data.

The Investigator will enter the data required by this protocol into the eCRF provided by the Sponsor or its representative. Monitors should visit each study site at the frequency described in the monitoring plan to check the eCRF for completeness and accuracy. Any discrepancies between the original documentation and the completed eCRF should be asked clarifying questions and the Investigator should answer and/or correct these discrepancies. After entering all the necessary information into the eCRF and resolving all clarifying questions, the Investigator must sign the eCRF for each patient. Upon completion of the study, the database (eCRF) will be provided to the Sponsor, as well as to the research centers.

1. PLANNED STATISTICAL METHODS
   1. **General provisions**

A statistical analysis plan will be prepared before the database of this study is closed for final analysis. Any deviations from the planned analysis will be described and justified in the overall final study report.

- 1. **Determination of the sample size**

The sample size was determined in accordance with the methodology of one-stage non-comparative design of phase II clinical trials (RP A’Hern, 2001).

At the moment, the process of recovery of lung function in patients who have undergone COVID-19 is not well understood [20]. In this study, 25% of patients in the Placebo group and 50% of patients in the Treamide group are expected to achieve a clinically significant change in FVC and/or DLCO at Week 4 from baseline. For α = 0.05 (one-tailed), power 80%, p0=25% and p1=50%, 26 patients must be included in the analysis to test the hypothesis. 11 or more responses in 26 included patients will allow us to reject the null hypothesis (Н _0_ : p ≤ p _0_ ) in favor of the alternative one (Н _1_ : p ≥ p _1_ ) and thus make a positive decision regarding the possibility of further study of Treamide in phase III.

Taking into account the possible early withdrawal (impossibility of evaluating the primary endpoint) not exceeding 13%, 30 patients will be included in each group of study therapy. Thus, 6o patients will be randomized into the study.

- 1. **Randomization**

Will be randomized into two groups in a 1:1 ratio using the IWRS system. Randomization will be stratified by risk factors (age ≥ 60 years and/or chronic comorbidity) and baseline CT lung involvement.

- 1. **Populations for analysis**
     1. **Full analysis set (FAS)**

The Full Analysis Set (FAS) is defined as all randomized patients who received at least one dose of study drug and have at least one post-baseline efficacy score. The FAS population is the main population for efficacy analysis.

- - 1. **Protocol Population (Per Protocol, PP)**

Per Protocol (PP) populations correspond to patients in the entire analysis population who will receive the study therapy in full and will not have significant deviations from the protocol. Major protocol violations will be described in more detail in the Statistical Analysis Plan and identified prior to closing the database.

- - 1. **Safety Population**

All patients who received at least one dose of study drug will be included in the safety analysis.

- - 1. **Population pharmacokinetics**

All patients who received at least one dose of study drug and for whom sufficient PK samples were obtained to evaluate at least one PK parameter will be included in the pharmacokinetics population.

- 1. **Patient distribution, demographic characteristics and baseline analysis**

Patient distribution, demographics, and baseline characteristics will be presented using descriptive statistics. The number and percentage of patients taking concomitant medications will be presented in frequency tables by therapeutic class and name of the active substance. The number of patients with pre-existing and comorbid medical conditions will be presented. Dosing information, including daily doses, exposure time, and total dose, will be presented descriptively by treatment group.

- 1. **Procedures for accounting for missing, unanalysable and doubtful data**

Missing data will not be replaced.

- 1. **Performance analysis**
     1. **Primary efficacy endpoint**

The primary endpoint is the frequency of achieving a clinically meaningful change in FVC and/or DLCO at Week 4 from baseline. Clinically significant changes are considered to be a relative increase in FVC by ≥ 10% or a relative increase in FVC in the range from ≥ 5% to < 10% and a relative increase in DLCO by > 15% [13, 14].

According to the RP algorithm A'Hern for single-stage phase II studies with unilateral α=0.05 and 80% power, 11 or more of 26 patients included in the analysis who achieved a clinically significant change in FVC and/or DLCO at Week 4 from baseline are sufficient to accept decisions on the advisability of further study of this dose in phase III (taking into account the evaluation of additional parameters of efficacy and safety). If the placebo arm also has a Week 4 response rate of 11 or more in the 26 included patients, then the primary endpoint assessment may be deferred to an earlier study visit.

If the number of responses to therapy in the Treamide group of 26 included patients is 10 or less, it will be concluded that there is no sufficient efficacy.

- - 1. **Secondary efficacy endpoints**

Secondary efficacy endpoints include:

- Average change in distance walked in 6 minutes (6MWD) in Week 2 and Week 4 from baseline (based on the 6 Minute Walk Test);
- Mean change in Borg score at Week 2 and Week 4 from baseline (based on the 6 Minute Walk Test);
- Mean relative change in spirometry lung function parameters (FEV1, FVC, FEV1/FVC) at Week 1, Week 2, Week 3, and Week 4 from baseline;
- Mean relative change in body plethysmography lung function parameters at Week 2 and Week 4 from baseline (DLCO, TLC, and FRC);
- Rate of reduction in lung injury (improvement) on CT scan at Week 4 from baseline;
- Mean change in severity on the mMRC Breathlessness Scale at Week 1, Week 2, Week 3, and Week 4 from baseline;
- The mean change in total scores on the KBILD Questionnaire in Week 2 and Week 4 from baseline.

For secondary endpoints, intergroup comparisons of frequencies and categorical data will be made using the chi-square test or Fisher's exact test, continuous data using the non-parametric Mann-Whitney test or Student's t-test.

- 1. **Safety analysis**

Study drug dosing information, including daily doses, time of exposure, and total dose, will be presented descriptively for each cohort.

Safety will be assessed based on the incidence of AEs and SAEs based on subjective complaints, physical examination, vital signs, laboratory tests, and ECG. The number and percentage of patients with AEs and SAEs will be tabulated by organ system class and preferred term (according to MedDRA), by study drug association, and severity. The results of laboratory studies will be summarized in tables of changes. Vital and laboratory parameters, SpO2 and ECG data will be presented using descriptive statistics.

- 1. **Pharmacokinetic analysis**

The following PK parameters of the study drug will be assessed: C _max_, T _max_, AUC _0-t_ and C _trough_. Validated software ™ WinNonlin ® (CERTARA, Pharsight, USA) will be used to calculate pharmacokinetic parameters. The analysis will be carried out using descriptive statistics methods.

The study therapy will be blinded prior to the PK study. PK analysis for patients receiving placebo will only be performed if there is doubt about the correctness of the blinding procedure. To confirm the absence of the study drug in the blood plasma of patients who received placebo, analysis can be performed at points corresponding to the calculated Tmax values.

1. ADMINISTRATIVE PROCEDURES
   1. **Legal aspects**

The names of all patients must be kept strictly confidential. Patients are identified by their assigned number, initials and date of birth. Patients should be advised that any information held by the study site, the Sponsor or its representatives will be held in strict compliance with the principle of confidentiality in accordance with regulatory requirements.

- - 1. **Responsibilities of the Explorer**

The investigator is required to conduct the clinical trial in accordance with the requirements of this protocol, the Good Clinical Practice Guidelines of the International Conference on Harmonization (ICH GCP) and the Eurasian Economic Union (EAEU), the principles laid down in the Declaration of Helsinki, as well as in accordance with the current legislation and applicable regulations. requirements.

The Investigator must agree to conduct monitoring, audits and inspections in the research center and at any time, upon request, provide direct access to the study materials to the Sponsor and its representatives, the Independent Ethics Committee and authorized bodies.

The investigator must keep the originals of all informed consent forms signed as part of the study, as well as a complete list of study subjects, including full name, assigned number, initials, date of birth, address, and telephone number, so that they can be identified later, if necessary. These documents must not be copied for distribution to the Sponsor or its representatives.

- 1. **Monitoring procedures**

The Investigator must provide direct access to all primary documentation, the eCRF and the Investigator File to the Sponsor and its representatives, the IEC and authorized bodies. Primary documentation includes source documents, data and records, including medical histories, outpatient records, laboratory records, notes, drug dispensing journals, records of automatic devices, verified and certified copies or extracts, photo negatives, microfilms or magnetic media, CT scans. , any records related to the patient, including those stored in a pharmacy, laboratories and departments of instrumental diagnostics used in a clinical trial.

Monitoring will be conducted by the Sponsor or its representative in accordance with their SOP and the Monitoring Plan developed for this study.

- 1. **Data registration in eCRF**

The investigator should transfer the data obtained during the clinical trial from the primary documentation to the eCRF to the extent required. The rules for completing the eCRF will be reviewed by the Investigator during the Investigator meeting and/or during the initiation visit. If the Investigator delegates the authority to complete the eCRF to other research facility staff, their names, titles, signatures, and initials must be entered on the study facility staff assignment sheet and provided to the Sponsor or its representative.

When corrections are made to the eCRF, a documentary trace must remain, i.e. Before and after the change, reason (if applicable), date and who made the change should be visible. Completed eCRFs must be signed by the Investigator.

- 1. **Storage of documentation**

Primary documentation, eCRF data, and the Investigator's File must be kept at the research facility or in a specially designed archive with limited access for at least 25 years after the completion of the study or until the date specified by the Sponsor. The sponsor must be informed if the documentation is transferred to other persons or to another institution. Materials related to the clinical study will be kept by the Sponsor and its representatives in accordance with regulatory requirements.

- 1. **Ethical aspects**
     1. **Independent Ethics Committee**

Prior to the commencement of a clinical trial, all study sites will receive written approval from Independent Ethics Committees (IECs) in accordance with the Good Clinical Practice Guidelines of the International Conference on Harmonization (ICH GCP) and the Eurasian Economic Union (EAEU), the principles set out in the Declaration of Helsinki, and also in accordance with applicable law and applicable regulatory requirements of the Russian Federation. The following documents must be submitted to the IEC for review: study protocol and amendments to it, patient information sheet and informed consent form, written materials to be provided to patients, investigator's brochure, information on the safety of the use of the investigational drug, information on payments and compensation to patients, scientific biography of the Investigator and other documents on request.

A list of IEC members and a statement of its organization and operation in accordance with the principles of good clinical practice and regulatory requirements must be provided to the Sponsor.

- - 1. **Ethical Conduct of Clinical Research**

The procedures described in the clinical trial protocol relating to its conduct, evaluation and documentation of results are designed to ensure that the Sponsor and Investigator follow the International Conference on Harmonization (ICH GCP) and the Eurasian Economic Union (EAEU) Good Clinical Practice Guidelines. This clinical study will also be conducted in accordance with applicable law and applicable regulatory requirements. This includes the possibility of audits and inspections by representatives of the Sponsor and/or authorized bodies. The Investigator must agree to conduct monitoring, audits and inspections in the research center and at any time, upon request, provide direct access to the study materials to the Sponsor and its representatives, the Independent Ethics Committee and authorized bodies.

- - 1. **Informed Consent**

Before starting a clinical investigation, the Investigator must obtain written approval from the IEC of the Patient Information Sheet and Informed Consent Form, as well as any other written information to be provided to patients. The IEC's written approval and approved documents must be included in the Study File.

The process of obtaining informed consent must be carried out in accordance with the Good Clinical Practice Guidelines of the International Conference on Harmonization (ICH GCP) and the Eurasian Economic Union (EAEU), the principles set out in the Declaration of Helsinki, as well as in accordance with the current legislation and applicable regulatory requirements of the Russian Federation .

The informed consent form must be hand-signed and dated by the patient prior to the commencement of any study procedures, and must be retained with the patient's primary documentation. The process of obtaining informed consent should be described in detail in the primary documentation, including the fact that the patient consented to participate in this clinical study, the date of signing and the version of the informed consent form.

- 1. **Research funding**

The study is being conducted and fully funded by PHARMENTERPRICES LLC. All costs associated with the conduct of the study, including the payment of state fees, insurance premiums, payments to research centers, Investigators and third parties involved in the study, as well as the provision of the study drug and other study materials, are covered by PHARMENTERPRICES LLC.

Participation in the study does not require any financial costs from patients. All research procedures, including visits to the center, laboratory and instrumental studies, will be performed free of charge. Participation in this study does not imply the payment of monetary rewards to patients.

The life and health of patients participating in this clinical trial will be insured in accordance with the legislation of the Russian Federation (Article 44 of the Federal Law "On the Circulation of Medicines" No. 61-FZ of April 12, 2010, Government Decree No. 714 of September 13, 2010 d. subject to current changes). Upon signing the informed consent form, patients will receive a Compulsory Life and Health Insurance Policy for a patient participating in clinical trials of a medicinal product.

1. **PLAN OF PUBLICATIONS**

Both complete and partial research results obtained under this protocol, as well as any other information provided by the Sponsor for the study, must not be published or transferred to a third party without the consent of the Sponsor. Investigators involved in the study are required to provide the Sponsor with complete and accurate data obtained during the course of the study.

1. **REFERENCES**
2. MINISTRY OF HEALTH OF THE RUSSIAN FEDERATION. Temporary guidelines. Prevention, diagnosis and treatment of new coronavirus infection (COVID-19). Version 7.0 from 06/03/2020
3. Wang D., et al. Clinical characteristics of 138 hospitalized patients with 2019 novel coronavirus-infected pneumonia in Wuhan, China. JAMA. 2020 Mar 17; 323(11): 10611069.
4. Huang C., et al. Clinical features of patients infected with 2019 novel coronavirus in Wuhan, China. Lancet 2020;395(10223):497-506.
5. Li Q. et al. Early transmission dynamics in Wuhan, China, of novel coronavirus-infected pneumonia. N Engl J Med. 2020 Mar 26;382(13):1199-1207.
6. Chen N. et al. Epidemiological and clinical characteristics of 99 cases of 2019 novel coronavirus pneumonia in Wuhan, China: a descriptive study. Lancet

2020;395(10223):507-13.

1. Miriam Merad, Jerome C Martin, Pathological Inflammation in Patients With COVID-19: A Key Role for Monocytes and Macrophages, Nat Rev Immunol. 2020 May 6;1-8. https://www.nature.com/articles/s41577-020-0331-4.
2. Kai Liu et al. Respiratory Rehabilitation in Elderly Patients With COVID-19: A Randomized Controlled Study Complement Ther Clin Pract. 2020 May;39:101166, https://doi.org/10.1016/j.ctcp.2020.101166
3. Mo, X., Jian, W., Su, Z., Chen, M., Peng, H., Peng, P., Zhong, N. Abnormal pulmonary function in COVID-19 patients at time of hospital discharge. Eur Respir J. 2020 Jun 18;55(6):2001217
4. George, P. M., Wells, A. U., Jenkins, R. G. Pulmonary fibrosis and COVID-19: the potential role for antifibrotic therapy. Lancet Respir Med. 2020 May 15
5. Efficacy and Safety of Nintedanib in the Treatment of Pulmonary Fibrosis in Patients With Moderate to Severe COVID-19. https://clinicaltrials.gov/ct2/show/NCT04338802
6. A Study to Evaluate the Efficacy and Safety of Pirfenidone With Novel Coronavirus Infection. https://clinicaltrials.gov/ct2/show/NCT04282902
7. Spagnolo, P., Balestro, E., Aliberti, S., et al. Pulmonary fibrosis secondary to COVID-19: a call to arms? Lancet Respir Med. 2020 May 15
8. Raghu, G., Collard, H. R., Anstrom, K. J., et al. Idiopathic Pulmonary Fibrosis: Clinically Meaningful Primary Endpoints in Phase 3 Clinical Trials. Am J Respir Crit Care Med. 2012 May 15; 185(10): 1044-1048.
9. Yamano, Y., Taniguchi, H., Kondoh, Y., et al. Multidimensional improvement in connective tissue disease-associated interstitial lung disease: Two courses of pulse dose methylprednisolonefollowedbylow-doseprednisoneand

tacrolimus. Respirology. 2018 Nov;23(11):1041-1048

1. WHO R&D Blueprint/novel Coronavirus/COVID-19 Therapeutic Trial Synopsis/Draft February 18,2020. https://www.who.int/docs/default-source/blue-print/covid-19- therapeutic-trial-synopsis.pdf?sfvrsn=44b83344_1&download=true
2. Temporary guidelines. Medical rehabilitation for novel coronavirus infection (COVID 19). Version 1 (05/21/2020).
3. Clinical guidelines: Idiopathic pulmonary fibrosis. Russian Respiratory Society, 2016.
4. American Thoracic Society. ATS Statement: Guidelines for the Six-Minute Walk Test. Am J Respir Crit Care Med Vol 166. pp 111-117, 2002 DOI: 10.1164/rccm.166/1/111 Internet address: www.atsjournals.org
5. Chikina S.Yu. Out-of-laboratory stress tests in pathology of the respiratory system. Creative. surg. and oncol. 2010;1:820-95. Available at: https://cyberleninka.ru/article/n/vnelaboratornye-nagruzochnye-testy-pri-patologii-organov-dyhaniya
6. Liu, K., Zhang, W., Yang, Y., Zhang, J., Li, Y., & Chen, Y. (2020). Respiratory rehabilitation in elderly patients with COVID-19: A randomized controlled study. ComplementaryTherapiesinClinicalPractice,39,101166. doi:10.1016/j.ctcp.2020.101166
7. National Commission for the Protection of Human Subjects of Biomedical and Behavioral Research. The Belmont Report: ethical principles and guidelines for the protection of human subjects of research. Washington, DC: Dept of Health, Education, and Welfare (DHEW) publication nos. (OS) 78-0012, appendix I; (OS) 78-0013, appendix II; and (OS) 78-0014; 1978
8. Efficacy and Safety of Nintedanib in the Treatment of Pulmonary Fibrosis in Patients With ModeratetoSevereCOVID-19. https://clinicaltrials.gov/ct2/show/record/NCT04338802?term=nintedanib&draw=2&rank=12&view=record
9. Rules of Good Clinical Practice of the Eurasian Economic Union. Approved by the Decision of the Council of the Eurasian Economic Commission dated November 3, 2016 No. 79.
10. Collard HR, King TE Jr, Bartelson BB, Vourlekis JS, Schwarz MI, Brown KK. Changes in clinical and physiologic variables predict survival in idiopathic pulmonary fibrosis. Am J Respir Crit Care Med 2003; 168: 538-542.
11. King TE Jr, Safrin S, Starko KM, Brown KK, Noble PW, Raghu G, Schwartz DA. Analyzes of efficacy end points in a controlled trial of interferon-gamma1b for idiopathic pulmonary fibrosis. Chest 2005; 127: 171- 177.
12. Bethany B Moore, Cory M Hogaboam. “Murine models of pulmonary fibrosis”. Am J Physiol Lung Cell Mol Physiol. 2008 Feb;294(2):L152-60,
13. Amber L Degryse, William E Lawson. “Progress toward improving animal models for idiopathic pulmonary fibrosis”. Am J Med Sci. 2011 Jun;341(6):444-9
14. Antje Moeller, Kjetil Ask, David Warburton, Jack Gauldie, Martin Kolb. “The bleomycin animal model: a useful tool to investigate treatment options for idiopathic pulmonary fibrosis?” Int J Biochem Cell Biol. 2008;40(3):362-82
15. Yan Cai, Lei Zhu, Fan Zhang, Gang Niu, Seulki Lee, Shioko Kimura, Xiaoyuan Chen. “Noninvasive monitoring of pulmonary fibrosis by targeting matrix metalloproteinases (MMPs)”, Mol Pharm. 2013 Jun 3;10(6):2237-47,
16. Viranuj Sueblinvong, David C Neujahr, S Todd Mills, Susanne Roser-Page, Jeffrey D Ritzenthaler, David Guidot, Mauricio Rojas, Jesse Roman. “Predisposition for disrepair in the aged lung“ Am J Med Sci. 2012 Jul;344(1):41-51
17. William E Lawson, James E Loyd. “The genetic approach in pulmonary fibrosis: can it provide clues to this complex disease?” Proc Am Thorac Soc. 2006 Jun;3(4):345-9,
18. William E Lawson, Vasiliy V Polosukhin, Georgios T Stathopoulos, Ornella Zoia, Wei Han, Kirk B Lane, Bo Li, Edwin F Donnelly, George E Holburn, Kenneth G Lewis, Robert D Collins, William M Hull, Stephan W Glasser, Jeffrey A Whitsett, Timothy S Blackwell. “Increased and advanced pulmonary fibrosis in surfactant protein C-deficient mice following intratracheal bleomycin” Am J Pathol . 2005 Nov;167(5):1267-77
19. Peter M George, Athol U Wells, R Gisli Jenkins. “Pulmonary fibrosis and COVID-19: the potential role for antifibrotic therapy” Lancet Respir Med. 2020 May 15;S2213- 2600(20)30225-3
20. Paolo Spagnolo, Elisabetta Balestro, Stefano Aliberti, Elisabetta Cocconcelli, Davide Biondini, Giovanni Della Casa, Nicola Sverzellati, Toby M Maher. “Pulmonary fibrosis secondary to COVID-19: a call to arms?” Lancet Respir Med. 2020 May 15;S2213- 2600(20)30222-8
21. FN Novikov, VS Stroylov, IV Svitanko, VE Nebolsin, "Molecular bases of COVID-19 pathogenesis", RUSS CHEM REV, 2020, 89 (8), 858-878
22. Efficacy and Safety of Nintedanib in the Treatment of Pulmonary Fibrosis in Patients With Moderate to Severe COVID-19. Sponsor: Huilan Zhang. ClinicalTrials.gov Identifier: NCT04338802. April 2020
23. A Study to Evaluate the Efficacy and Safety of Pirfenidone With Novel Coronavirus Infection. Sponsor: Huilan Zhang. ClinicalTrials.gov Identifier: NCT04282902. February 25, 2020
24. Jessica Wagner, C Leah Kline, Lanlan Zhou, Vladimir Khazak, Wafik S El-Deiry. “Antitumor effects of ONC201 in combination with VEGF-inhibitors significantly impacts colorectal cancer growth and survival in vivo through complementary non-overlapping mechanisms.” J Exp Clin Cancer Res. 2018: 37, 1.
25. Yi Wang , Ruo-Wu Shen , Bing Han , Zhen Li , Le Xiong , Feng-Yu Zhang , Bei-Bei Cong, Bei Zhang. “Notch signaling mediated by TGF-β/Smad pathway in concanavalin A- induced liver fibrosis in rats”. World J Gastroenterol. 2017 Apr 7; 23(13): 2330-2336
26. Guidelines for the provision and maintenance of peripheral venous access. Developed and approved by the Association of Nurses of Russia. http://medsestre34.ru/assets/files/files/method_rekom.pdf
27. Russian respiratory society. FEDERAL CLINICAL RECOMMENDATIONS FOR THE USE OF THE SPIROMETRY METHOD, 2013
28. A series of monographs of the Russian Respiratory Society, ed. A.G. Chuchalin. Functional diagnostics in pulmonology. Ed. Z.R. Aisanova, A.V. Chernyak. 2016. - 184 p., ill.

Appendix 1. mMRC (modified Medical Research Council) dyspnea score

| Degree | Severity | Appearance |
| --- | --- | --- |
| 0 | No | I only feel short of breath when I exert myself heavily |
| 1 | light | I get out of breath when I walk quickly on level ground or climb a gentle hill |
| 2 | average | Because of my shortness of breath, I walk more slowly on level ground than people of the same age, or I stop breathing when I walk on level ground at my normal pace |
| 3 | heavy | I am out of breath after walking about 100m or after walking for a few minutes on level ground |
| 4 | very heavy | I am too short of breath to leave the house or suffocate when I get dressed or undressed |

Source: Chronic obstructive pulmonary disease, Clinical guidelines. Russian respiratory society.

Annex 2. 6-minute walk test (6MWD, Borg scale) (sample)

The 6MWD (6-minute walk) test is carried out in a level, straight, closed hallway with a hard floor that is easy to walk on. Weather permitting, the test can be performed outdoors. The corridor is marked every 3 m. The beginning and end of the distance are marked with cones (similar to the orange cones used in road repairs), which are placed at a distance of 0.5 m from the ends of the corridor.

Necessary equipment

1. Chronometer or stopwatch.
2. Two small cones to indicate turning points.
3. Mechanical distance counter (pedometer).
4. A chair that easily moves along the distance.
5. Source of oxygen.
6. Tonomanometer.
7. Telephone.
8. Portable defibrillator.
9. Emergency aid.

Preparing the Patient for 6MWD

1. Comfortable clothing that does not restrict movement.
2. Shoes that are comfortable for walking.
3. The patient is allowed to use walking aids (canes, etc.) that he uses on a daily basis.
4. The patient's usual regimen of medication is maintained.
5. Before the test, the patient is allowed to take a light breakfast (lunch).
6. Vigorous physical activity is not recommended for 2 hours before the test.

Testing

Prior to the start of the study, the patient should sit quietly in a chair near the starting line for at least 10 minutes. During this time, it is necessary to evaluate the contraindications for the study, measure the pulse and pressure, and make sure that the patient's clothing and shoes are suitable for the test.

Pulse oximetry should preferably be carried out during the entire test. To monitor saturation, the Investigator does not need to walk the entire distance with the patient. The pulse oximeter should be attached to the patient's clothing, be light (no more than 900 g) and comfortable in shape so that the patient does not have to hold it, and so that it does not interfere with walking.

To assess dyspnea on exertion, either the Borg scale or the visual analogue scale is used. Before starting 6MWD, you need to show the scale to the patient and ask him to determine the degree of respiratory discomfort and general fatigue on it. At the end of testing, the patient should be reminded of their initial scores and asked to re-evaluate these symptoms.

**Borg scale for assessing dyspnea**

| **Points** | **Severity of shortness of breath** |
| --- | --- |
| 0 | No shortness of breath at all |
| 0.5 | Very, very light (almost imperceptible) |
| 1 | Very mild shortness of breath |
| 2 | Mild (mild) shortness of breath |
| 3 | Average shortness of breath |
| 4 | Moderate shortness of breath |
| 5 | severe shortness of breath |
| 6 | Fairly severe shortness of breath |
| 7 | Very severe shortness of breath |
| 8 | Very, very severe shortness of breath |
| 9 | Extremely severe shortness of breath |
| 10 | Unbearable (extremely severe) shortness of breath |

The examiner should instruct the patient as follows: “The aim of this study is to walk as fast as possible within 6 minutes. This is a long enough time for walking, so you yourself determine the intensity of your load. You may experience increased shortness of breath or fatigue. At the same time, you can slow down the pace of walking, and if necessary, stop and rest. During rest, you can lean against the wall, but then you should continue walking. You need to walk back and forth along the corridor, bypassing the cones. You need to turn around the cones quickly and then without delay continue to move in the opposite direction. It is recommended to walk one circle and demonstrate to the patient how to walk.

When the patient starts walking, you need to turn on the timer.

It is impossible even with gestures to demand from the patient to increase the speed of walking. If the patient stopped during the study and needs to rest, the timer is not turned off for this time. If the patient refuses to continue walking (or the investigator believes that the test should be terminated), the chair should be moved and the patient should be seated in it and the distance covered, the time and the reason for the premature termination of the study should be recorded in the protocol. For 15 sec. before the end of the test, the patient should be warned so that, after the request to stop, he stops where he will be at that moment, after which the Investigator himself approaches the patient and, if necessary, rolls up a chair to him. The stopping point is marked on the floor with a piece of bright tape or another marker.

After the test is completed, the saturation, pulse rate, degree of dyspnea and fatigue are again measured on the Borg scale or visual analog scale, and the patient is also asked about the reasons that prevented him from walking faster, and indicate them in the protocol.

To calculate the distance traveled, use the readings of a pedometer or markings on the wall of the corridor.

Annex 3. King Interstitial Lung Disease Scoring Questionnaire (KBILD) - Sample

Please circle the answer that best fits

1. For the past 2 weeks, I have been out of breath when climbing stairs or climbing a slope or hill.

1. Every time 2. In most cases 3. Several Times 4. Sometimes 5. Sometimes 6. Rarely 7. Never
2. In the last 2 weeks, due to lung disease, my chest was squeezed.

1. All the time 2. Most of the time 3. Good part of the time 4. Sometimes 5. A little time 6. Almost all the time 7. Never in my life

1. In the past 2 weeks, have you been worried about the severity of your lung complaint?
2. All the time 2. Most of the time 3. Good part of the time 4. Sometimes 5. A little time 6. Almost all the time 7. Never in my life
3. In the past 2 weeks, have you avoided doing things that make you choke?

1. All the time 2. Most of the time 3. Good part of the time 4. Sometimes 5. A little time 6. Almost all the time 7. Never in my life

1. In the past 2 weeks, have you felt that you are in control of your lung condition?

1. Never all the time 2. Almost all the time 3. A little time 4. Sometimes 5. Good part of the time 6. Most of the time 7. All this time

1. In the past 2 weeks, has your lung condition made you feel depressed?

1. All the time 2. Most of the time 3. Good part of the time 4. Sometimes 5. A little time 6. Almost all the time 7. Never in my life

1. For the past 2 weeks I have felt the need to breathe, described as "shortness of breath".

1. All the time 2. Most of the time 3. Good part of the time 4. Sometimes 5. A little time 6. Almost all the time 7. Never in my life

1. For the past 2 weeks, my lung condition has been making me feel anxious.

1. All the time 2. Most of the time 3. Good part of the time 4. Sometimes 5. A little time 6. Almost all the time 7. Never in my life

1. In the past 2 weeks, how often have you experienced "wheezing" or whistling sounds from your chest?

1. All the time 2. Most of the time 3. Good part of the time 4. Sometimes 5. A little time 6. Almost all the time 7. Never in my life

1. In the past 2 weeks, how often have you felt like your lung condition was getting worse?
2. All the time 2. Most of the time 3. Good part of the time 4. Sometimes 5. A little time 6. Almost all the time 7. Never in my life
3. In the past 2 weeks, has your lung condition interfered with your work or other daily tasks?

1. All the time 2. Most of the time 3. Good part of the time 4. Sometimes 5. A little time 6. Almost all the time 7. Never in my life

1. Did you expect your lung complaints to get worse in the last 2 weeks?

1. All the time 2. Most of the time 3. Good part of the time 4. Sometimes 5. A little time 6. Almost all the time 7. Never in my life

1. In the past 2 weeks, how much did your lung condition limit you from carrying things like groceries?

1. All the time 2. Most of the time 3. Good part of the time 4. Sometimes 5. A little time 6. Almost all the time 7. Never in my life

1. In the past 2 weeks, has your lung condition made you think more about death?
2. All the time 2. Most of the time 3. Good part of the time 4. Sometimes 5. A little time 6. Almost all the time 7. Never in my life
3. Has your financial situation deteriorated due to lung disease?
4. Significant amount 2. Large quantity 3. Significant amount 4. Reasonable amount 5. small amount 6. Hardly at all 7. not at all

# **Appendix 2 Amendments to the Protocol**

**PROTOCOL OF THE CLINICAL STUDY**

**COVID-TRE-03**

| **Study Title:** | A multicenter, randomized, double-blind, placebo-controlled pilot study to evaluate the efficacy and safety of Treamide in the rehabilitation of patients after COVID-19 pneumonia |
| --- | --- |
| **Study Number:** | COVID-TRE-03 |
| **Study phase:** | II |
| **Name of the drug:** | Treamide |
| **Planned indication:** | Prevention of pulmonary fibrosis after COVID-19 pneumonia |
| **Sponsor:** | LLC "PHARMENTERPRISES" |
| **The contact person:** | Nebolsin Vladimir Evgenievich  Russia, 121205, Moscow,  The territory of the innovation center Skolkovo  Bolshoi boulevard, 42, building 1, office. 771, 772  Tel.: +7 (985) 728-75-72 |
| **Protocol date:** | July 20, 2020 |
| **Amendment 1:** | September 18, 2020 |

**Confidentiality Statement**

By receiving this document, you agree that it is the Confidential Information of PHARMENTERPRICES LLC. You must not directly or indirectly publish, distribute or otherwise disclose its contents or make available to third parties any part of this document. You must also use this document only for the purpose for which it was provided to you. You may disclose information only after the written permission of PHARMENTERPRICES LLC, or representatives of authorized government bodies, or by decision of the judicial authorities. The fact of any disclosure of the information contained in this document, authorized or not, must be immediately brought to the attention of PHARMENTERPRICES LLC.

PROTOCOL APPROVAL PAGE

| Signature | (signed) |  | Date | 30.09.2020 |
| --- | --- | --- | --- | --- |
| Nebolsin V.E.  General Manager  LLC "PHARMENTERPRISES" | |  |  |  |
|  |  |  |  |  |
| Signature | (signed) |  | Date | 30.09.2020 |
| Trakhtenberg Yu.A.  Medical director  IPHARMA LLC | |  |  |  |
|  |  |  |  |  |
| Signature | (signed) |  | Date | *30.09.2020* |
| Kasyanova O.V.  Medical Advisor  IPHARMA LLC | |  |  |  |
|  |  |  |  |  |
| Signature | (signed) |  | Date | *30.09.2020* |
| Samokhina M.V.  Project Manager  IPHARMA LLC | |  |  |  |
|  |  |  |  |  |

**STATEMENT FROM PRINCIPAL INVESTIGATOR**

| **FULL NAME.** : | |
| --- | --- |
| *I,* the undersigned, certify that I have read and understood this protocol. I agree to follow the COVID-TRE-03 protocol: "A multicenter, randomized, double-blind, placebo-controlled pilot study to evaluate the efficacy and safety of Treamide in the rehabilitation of patients after COVID-19 pneumonia."  I undertake to conduct this study in accordance with the requirements of the Good Clinical Practice Guidelines of the International Conference on Harmonization (ICH GCP) and the Eurasian Economic Union (EAEU), the principles set forth in the Declaration of Helsinki, as well as in accordance with the current legislation and applicable regulatory requirements of the Russian Federation. | |
| **Date:** | **Signature:** |

**CLINICAL STUDY CONTACT**

| Sponsor | LLC "PHARMENTERPRISES"  Russia, 121205, Moscow,  The territory of the innovation center Skolkovo  Bolshoi boulevard, 42, building 1, office. 771, 772 |
| --- | --- |
| Nebolsin V.E. | General Manager  Tel.: +7 (985) 728-75-72  Email address: nve1970@mail.ru |
| **CRO, MEDICAL EXAMINATION, PHARMACOVIGILANCE DATA PROCESSING AND BIOSTATISTICS** | IPHARMA LLC  Russia, 143026, Moscow,  The territory of the innovation center Skolkovo  st. Nobel, d.7  Tel.: +7 (495) 276-11-43  *Fax +7(495) 276-11-47*  Address for sending a safety report:  SAE@ipharma.ru |
| Vostokova N.V. | Chief Operations Officer  Tel.: +7 (926) 098-36-33  Email address: nv@ipharma.ru |
| Trakhtenberg Yu.A. | Medical director  Tel.: +7 (926) 098-21-07  Email address: jat@ipharma.ru |
| Samokhina M.V. | Project Manager  Tel.: +7 (926) 547-98-17  Email address: msam@ipharma.ru |
| Kasyanova O.V. | Medical Advisor  Tel.: +7 (925) 421-52-23  Email address: okas@ipharma.ru |
| Kholkin P.V. | Director of Data Management  Tel.: +7 (926) 208-85-82  Email e-mail: khopv@ipharma.ru |
| Egorova A.N. | biostatistician  Tel.: +7 (926) 010-42-11  Email address: ean@ipharma.ru |
| **BIOANALYTICAL LABORATORY** | LLC NII KhimRar  Russia, 141401, MO, city of Khimki, Khimki, st. Rabochaya, d. 2A, building 1  Tel.: +7 (495) 925-3074  *Fax +7(495) 626-9780* |
| Koryakova A.G. | Head of Bioanalytics Laboratory  Tel.: +7 (495) 925-3074  Email address: agk@iihr.ru |
| **{U>WAREHOUSE<U}** | JSC IIHR  Russia, 141400, Moscow region, Khimki, st. Rabochaya, d. 2A, building 1  Tel.: +7 (495) 995-49-41  Email: iihr@iihr.ru |
| Clinical sites | According to the approved list of centers |

**SYNOPSIS**

**Sponsor:** PHARMENTERPRICES LLC

**Investigational product name:** Treamide, film-coated tablets

**Active ingredient:** XC268BG

**Study Title:**

A multicenter, randomized, double-blind, placebo-controlled pilot study to evaluate the efficacy and safety of Treamide in the rehabilitation of patients after COVID-19 pneumonia

**Study Number:** COVID-TRE-03

**Research phase:** II

**Main goal**:

To evaluate the efficacy of Treamide versus placebo in patients with COVID-19 pneumonia based on the frequency of achieving a clinically significant change in forced vital capacity (FVC) and/or diffusive capacity of the lungs (DLCO) at Week 4 from baseline.

Clinically significant changes are considered to be a relative increase in FVC by ≥ 10% ***or*** a relative increase in FVC in the range from ≥ 5% to < 10% and a relative increase in DLCO by ≥ 15% [13, 14].

**Additional goals:**

To evaluate the efficacy and safety of Treamide versus placebo in patients with COVID-19 pneumonia based on the following parameters:

- Change in distance traveled in 6 minutes (6MWD) in Week 2 and Week 4 from baseline (based on the 6 Minute Walk Test);
- Change in Borg score at Week 2 and Week 4 from baseline (based on the 6 Minute Walk Test);
- Relative change in spirometry lung function parameters (forced expiratory volume in one second (FEV1), FVC, FEV1/FVC) at Week 1, Week 2, Week 3, and Week 4 from baseline;
- Relative change in body plethysmography lung function parameters at Week 2 and Week 4 from baseline (DLCO, total lung capacity (TLC), expiratory reserve volume (FRC));
- Change in the degree of lung damage according to computed tomography (CT) at Week 4 from baseline;
- Change in severity on the mMRC Breathlessness Scale at Week 1, Week 2, Week 3, and Week 4 from baseline;
- Change in total score on the King's Interstitial Lung Disease Questionnaire (KBILD) at Week 2 and Week 4 from baseline;
- Residual concentration Ctrough of the active substance of the drug Treamide (XC268BG);
- The frequency of adverse events (AE) and serious adverse events (SAE) of varying severity according to subjective complaints, physical examination, vital signs, laboratory tests and ECG.

**Rationale:**

Starting in December 2019, the novel coronavirus SARS-CoV-2 has caused an outbreak of a respiratory disease called COVID-19 in almost every country in the world.

Clinical and epidemiological observations of patients with COVID-19 indicate that SARS-CoV-2 infection can range from mild signs of respiratory disease to severe progressive pneumonia, multiple organ failure, and death [1, 2, 3, 4, 5]. The presence of foci of lung tissue seals of the "ground glass" type and / or seals of interlobular septa, and / or areas of reticular changes in the lung tissue, and / or the presence of areas (linear, focal) of lung tissue seals according to chest CT data leads to the need for long-term rehabilitation patients who have had COVID-19 pneumonia [43, 44, 45].

According to modern concepts, pulmonary fibrosis is the process of formation of fibrous (scar) tissue in the lungs, which leads to impaired respiratory function. With fibrosis, the elasticity and extensibility of the lung tissue decreases, the passage of oxygen and carbon dioxide through the wall of the alveoli (lung vesicles, in which the inhaled air contacts the blood) is difficult.

Patients who have undergone COVID-19 are characterized by the development of pulmonary edema, accompanied by the presence of neutrophil-macrophage inflammation [6], as well as changes in respiratory function, in particular, a decrease in forced expiratory volume (FEV1) [7], a decrease in forced vital capacity (FVC) [7] and maximum expiratory flow rate at the level of 50% forced vital capacity (MEF50) [7].

Currently, any specific pharmacotherapy for patients at the stage of rehabilitation after suffering from COVID-19 pneumonia has not been described. Thus, the search for effective and safe drugs that could be effective in patients with fibrosis remains an urgent task [16].

Antifibrotic therapy, which has demonstrated activity in a model of pulmonary fibrosis induced by intratracheal administration of bleomycin, may be useful in the treatment of COVID-19 both in the acute phase of the disease and in the prevention and treatment of long-term complications [9]. Indirect confirmation of this fact is the conduct of clinical trials of drugs developed for the treatment of chronic fibrotic diseases, in particular, drugs Nintedanib [10] and Pirfenidone [11] for the treatment of pulmonary fibrosis caused by COVID-19 infection.

PHARMENTERPRICES LLC is developing a new drug Treamide to correct the development of fibrotic changes in the lungs at the stage of rehabilitation of patients after COVID-19 pneumonia.

The innovative drug Treamide is a bis-amide derivative of dicarboxylic acid. It was developed for use in patients with metabolic syndrome and age-related androgen deficiency. The mechanism of action of the drug formed the basis of its preclinical evaluation with the aim of repositioning for use in patients with COVID-19 pneumonia.

According to preclinical data, Treamide has anti-inflammatory and antifibrotic effects in models of lung injury, prevents tissue destruction, and improves respiratory function. Under conditions of pneumofibrosis, Treamide significantly (p<0.05) reduces the levels of total collagen, type I collagen, hydroxyproline, and the amount of deposited connective tissue in the lungs in mice. In a model of pulmonary fibrosis induced by intratracheal administration of bleomycin in mice, Treamide showed pronounced antifibrotic activity at a dose of 10 mg/kg per day. The model of fibrosis induced by intratracheal administration of bleomycin reproduces well the fibrotic response after acute lung injury (characteristic, in particular, of COVID-19 infection), rather than *de novo* progressive fibrosis [12]. It is important to note that the introduction of Treamide at a lower dose of 1 mg/kg did not lead to significant changes in the lung tissue in mice on the 21st day of the experiment. A potentially effective dose of 10 mg/kg per day in mice is approximately equivalent to 50 mg per day in humans.

Possible mechanisms of action of Treamide are an inhibitory effect on the migration of inflammatory cells to the lungs, as well as a decrease in the activity of synthesis and deposition of connective tissue.

The basis of the regenerative effects of Treamide is an inhibitory effect on the Notch-mediated mechanism for the development of pulmonary fibrosis and stimulation of the differentiation of lung stem cells (CD45-CD117+) and VEGF2+ endothelial cells.

In a phase I clinical study in healthy volunteers, Treamide showed good tolerability and a favorable safety profile at doses of 5 mg to 50 mg per day in single and multiple doses for 14 days. 4 AEs (asymptomatic Grade 3 and 4 laboratory changes) were reported only in the low-dose 5 mg cohort; No AEs associated with Treamide have been reported.

Based on data on the efficacy of Treamide in the most relevant disease model (a model of pulmonary fibrosis induced by intratracheal administration of bleomycin), as well as clinical data on the safety of Treamide at a dose of 50 mg once a day for 14 days in healthy volunteers, a pilot study is planned. phase II clinical trial to evaluate the safety and efficacy of Treamide at a dose of 50 mg once a day for 4 weeks in patients at risk of developing fibrotic changes in the lungs caused by the development of COVID-19 infection.

After completion of this study, with confirmation of the pilot hypothesis (proof-of-concept), it is planned to continue evaluating the efficacy and safety of Treamide in a multicenter, placebo-controlled, randomized phase III clinical trial of the efficacy and safety of Treamide in patients at risk of developing fibrotic changes in the lungs after undergoing COVID - 19 pneumonia.

**Study Design:**

This study is a multicenter, randomized, double-blind, placebo-controlled phase II pilot study to evaluate the efficacy and safety of Treamide in the rehabilitation of patients after COVID-19 pneumonia [15].

The study will be conducted in approximately 6-10 Russian clinical centers.

A total of 60 patients are planned to be included in the study. Considering a possible 43% drop-out at the study selection stage, approximately 90 patients at risk of developing fibrotic changes in the lungs after suffering from COVID-19 pneumonia will be screened.

***Screening***

At the Week -2 visit, following the signing of the Patient Information Sheet and the Informed Consent Form, screening procedures will be performed to assess inclusion/non-inclusion criteria. Screening procedures include collection of demographic data, medical history and concomitant therapy, physical examination, measurement of height, body weight and determination of BMI, assessment of vital signs and SpO2, ECG, mMRC dyspnea score, clinical and biochemical blood tests, urinalysis. Women of childbearing potential will be given a pregnancy test.

All patients will undergo a qualitative determination of SARS-CoV-2 RNA by PCR. The material for laboratory testing will be a swab from the nasopharynx and / or oropharynx. Confirmation of two consecutive negative PCR results at least 24 hours apart is required for inclusion in the study. In the case of previously confirmed elimination of the SARS-CoV-2 virus according to medical records, one determination of SARS-CoV-2 RNA by PCR with a negative result will be sufficient.

Patients will be assessed using the mMRC Breathlessness Scale, chest CT, spirometry, and body plethysmography with DLCO. These examinations should be conducted as close as possible to the planned date of randomization (not earlier than 5 days before it).

**In the event that screening procedures are initiated as part of the patient's discharge from the hospital after hospitalization of a patient diagnosed with COVID-19 pneumonia, sufficient time should be allowed for the completion of quarantine measures (about 2 weeks) before assessing the extent of damage and lung function.**

**The screening may use the results of examinations carried out as part of routine medical practice before obtaining informed consent (taking into account the window of the screening visit), except for the assessment on the mMRC Breathlessness Scale, CT of the chest, spirometry and body plethysmography with the definition of DLCO - these examinations should be carried out as much as possible close to the planned date of randomization (no earlier than 5 days before it).**

Patients who meet all eligibility criteria will be included in the study.

***Study therapy period***

At visit Week 0 prior to study therapy, registration of AEs and concomitant therapy, physical examination, body weight measurement, vital signs and SpO2 assessment, mMRC dyspnoea score, ECG, clinical and biochemical blood tests, and urinalysis will be performed. Women of childbearing potential will be given a pregnancy test. Patients will complete a KBILD questionnaire, followed by a 6 Minute Walk Test, which measures the distance the patient walks in 6 minutes and is assessed using the Borg Scale.

After completing all the necessary visit procedures, patients will be randomized into two groups in a 1:1 ratio.

Group 1 Treamide 50 mg - 30 patients

Group 2 Placebo - 30 patients

Patients will be given the required amount of study drug until the next visit, according to the therapy group.

The duration of study therapy will be 4 weeks. Patients will be recommended to continue the standard program of medical rehabilitation in a day hospital or outpatient setting (Stage 3 according to the Interim Guidelines "Medical Rehabilitation for Novel Coronavirus Infection (COVID 19)", Version 1 dated 05/21/2020 or current at the time of the study) [ sixteen].

Patients will visit the study site once a week during the study therapy phase. At Week 1, Week 2, and Week 3 visits, registration of AEs and concomitant medications, study medication, body weight, vital signs and SpO2, mMRC dyspnea score, and spirometry will be performed.

At visit Week 2, a physical examination, ECG, clinical and biochemical blood tests, PK study, urinalysis, body plethysmography with DLCO will also be performed. Patients will complete the KBILD questionnaire. There will also be a 6 Minute Walk Test, which will measure the distance the patient walks in 6 minutes and evaluate with the Borg Scale. Women of childbearing potential will be given a pregnancy test.

At visit Week 4 (end of therapy), registration of AEs and concomitant therapy, registration of the study drug, physical examination, measurement of body weight, assessment of vital signs and SpO2, ECG, clinical and biochemical blood tests, PK study, urinalysis will be carried out. Patients will be assessed using the mMRC Breathlessness Scale, chest CT, spirometry, and body plethysmography with DLCO. Patients will complete a KBILD questionnaire, followed by a 6 Minute Walk Test, which measures the distance the patient walks in 6 minutes and is assessed using the Borg Scale. After completion of study therapy, patients will continue treatment in accordance with general recommendations. Women of childbearing potential will be given a pregnancy test.

***PK study***

A blood draw for the Ctrough PK study will be performed on all patients prior to PI administration at Week 0, Week 2, and Week 4.

***Follow-up period.***

Patient follow-up will continue for another two weeks. At Week 6, registration of AEs and concomitant therapy, physical examination, measurement of body weight, assessment of vital signs and SpO2, mMRC dyspnea score, ECG, clinical and biochemical blood tests, urinalysis will be carried out. Women of childbearing potential will be given a pregnancy test.

**Study population:**

The study is planned to include 60 patients at risk of developing fibrotic changes in the lungs after suffering COVID-19 pneumonia.

To be included in this clinical trial, patients must meet the following inclusion/exclusion criteria.

**Inclusion criteria:**

1. Signed Patient Information Sheet and informed consent form for participation in the study.
2. Men and women aged 18 to 75 inclusive.
3. The risk of developing fibrotic changes in the lungs after suffering COVID-19 pneumonia:
4. The diagnosis of COVID-19 in history was confirmed by a positive qualitative analysis of SARS-CoV-2 RNA by PCR;
5. The appearance of the first symptoms of COVID-19 no more than 2 months before screening;
6. The presence of foci of lung tissue compaction of the "ground glass" type and / or seals of the interlobular septa, and / or areas of reticular changes in the lung tissue, and / or the presence of areas (linear, focal) of lung tissue seals characteristic of COVID-19, confirmed according to the data CT scan of the chest for screening.
7. Negative test for COVID-19 at screening (confirmed).
8. Severity 2 (moderate) or 3 (severe) on the mMRC Breathlessness Scale at screening and randomization.
9. Decreased lung function FVC and/or DLCO < 80% predicted at screening.
10. Consent of patients to use adequate methods of contraception during the entire study and within 3 months after its completion. Adequate methods of contraception include the use of:

- oral or transdermal contraceptives;
- condom or diaphragm (barrier method) with spermicide;
- intrauterine device.

**Non-inclusion сriteria:**

1. Women who are pregnant or breastfeeding or planning to become pregnant during a clinical study; women of childbearing potential (including those who are not surgically sterilized and who are less than 2 years postmenopausal) who do not use adequate contraceptive methods.
2. The use of invasive mechanical ventilation (iALV), plasma transfusion (including convalescent plasma) and other blood components during COVID-19 therapy.
3. History of chronic respiratory disease, including idiopathic pulmonary fibrosis (IPF), asthma, chronic obstructive pulmonary disease (COPD), or pulmonary hypertension, diagnosed prior to COVID-19.
4. Severe cardiovascular disease at present or within 6 months prior to screening, including: NYHA class III or IV chronic heart failure, clinically significant ventricular arrhythmias (ventricular tachycardia, ventricular fibrillation), unstable angina pectoris, myocardial infarction, heart and coronary surgery, significant valvular heart disease, uncontrolled arterial hypertension with systolic blood pressure > 180 mm Hg. and diastolic blood pressure > 110 mmHg, pulmonary embolism (PE) or deep vein thrombosis.
5. Nephrotic syndrome, moderate to severe chronic renal failure, or significant kidney disease with GFR < 60 ml/min at screening.
6. Cirrhosis of the liver in history; an increase in alanine aminotransferase (ALT) and / or aspartate aminotransferase (Act) by 3 or more times from the upper limit of normal (ULN) at screening; an increase in the level of total bilirubin by 2 or more times from ULN at screening.
7. Hemoglobin level < 90 g/l at screening.
8. Severe diseases of the central nervous system, including a history of seizures or conditions that can lead to their development; stroke or transient ischemic attack within 6 months prior to screening; traumatic brain injury or loss of consciousness within 6 months prior to screening; a brain tumor.
9. Signs of significant uncontrolled comorbidity, such as disorders of the nervous system, kidneys, liver, endocrine system and gastrointestinal tract, which, in the opinion of the Investigator, could prevent the patient from participating in the study.
10. Malignant neoplasms requiring chemotherapy within 6 months prior to screening.
11. History of HIV infection.
12. Prostate cancer or benign prostatic hyperplasia (BPH) with a residual urine volume of more than 100 ml in history in men.
13. Hypersensitivity or intolerance to any of the components of the study drug.
14. Participation in other clinical trials within 2 months prior to screening.
15. Taking the following drugs: bronchodilators, anticholinergics, corticosteroids, cytostatics, colchicine, cyclosporine A, interferon-γ-1b, bosentan, macitentan, etanercept, sildenafil, imatinib, n-acetylcysteine, warfarin, ambrisentan, nintedanib, pirfenidone 1 month before screening [17].
16. inability to read or write; unwillingness to understand and follow study protocol procedures; non-compliance with drug regimens or procedures that, in the opinion of the Investigator, may affect the results of the study or patient safety and prevent the patient from continuing to participate in the study; any other medical or serious psychiatric condition that makes the patient unsuitable for participation in the clinical study, limits the eligibility of obtaining informed consent, or may affect the patient's ability to participate in the study.

**Investigational drugs, doses and route of administration:**

**Investigational Drugs:** Treamide or Placebo

Active ingredient: XC268BG

Dosage form: film-coated tablets

Dosage: 50mg or 0mg

How to use: orally, once a day, in the morning, 30 minutes before breakfast

Storage conditions: In a place protected from light, at a temperature not exceeding 25°C.

Keep out of reach of children

Producer: JSC "IIHR" and CJSC "OHFK" Russia (by order of LLC "PHARMINTERPRICEZ", Russia)

**Duration of treatment:**

Each patient will participate in the study for approximately 8 weeks: screening 2 weeks, study therapy period 4 weeks, follow-up period 2 weeks. The start of enrollment in the study is scheduled for August 2020. It is planned that all patients will complete all study visits by November 2020.

**Primary Endpoint:**

Frequency of achieving a clinically significant change in FVC and/or DLCO at Week 4 from baseline. Clinically significant changes are considered to be a relative increase in FVC by ≥ 10% or a relative increase in FVC in the range from ≥ 5% to < 10% and a relative increase in DLCO by ≥ 15% [13, 14].

**Secondary Endpoints:**

- Mean change in distance walked in 6 minutes (6MWD) in Week 2 and Week 4 from baseline (based on the 6 Minute Walk Test) [19];
- Mean change in Borg score at Week 2 and Week 4 from baseline (based on the 6 Minute Walk Test);
- Mean relative change in spirometry lung function parameters (FEV1, FVC, FEV1/FVC) at Week 1, Week 2, Week 3, and Week 4 from baseline;
- Mean relative change in body plethysmography lung function parameters at Week 2 and Week 4 from baseline (DLCO, TLC, and FRC);
- Rate of reduction in lung injury (improvement) on CT scan at Week 4 from baseline;
- Mean change in severity on the mMRC Breathlessness Scale at Week 1, Week 2, Week 3, and Week 4 from baseline;
- Mean change in total score on the KBILD Questionnaire in Week 2 and Week 4 from baseline;
- C _trough_ the active ingredient XC268BG.

**Safety ratings:**

Safety will be assessed based on the incidence and severity of AEs and SAEs based on subjective complaints, vital signs, SpO2, physical examination, laboratory findings, and ECG.

**Statistical methods:**

Analysis of the research results will be carried out in accordance with the approved Statistical Analysis Plan.

The safety population would be all patients who received at least one dose of study drug.

The Full Analysis Set (FAS) is defined as all randomized patients who received at least one dose of study drug and have at least one post-baseline efficacy score.

Per Protocol (PP) populations correspond to patients in the entire analysis population who will receive the study therapy in full and will not have significant deviations from the protocol.

All patients who received at least one dose of study drug and for whom sufficient PK samples were obtained to evaluate at least one PK parameter will be included in the pharmacokinetics population.

**Sample size**

The sample size was determined in accordance with the methodology of one-stage non-comparative design of phase II clinical trials (RP A’Hern, 2001).

At the moment, the process of recovery of lung function in patients who have undergone COVID-19 is not well understood [20]. In this study, 25% of patients in the Placebo group and 50% of patients in the Treamide group are expected to achieve a clinically significant change in FVC and/or DLCO at Week 4 from baseline. For α = 0.05 (one-sided), power 80%, p0=25% and p _1_ =50%, 26 patients must be included in the analysis to test the hypothesis. 11 or more answers in 26 included patients will allow us to reject the null hypothesis (_W0_: p ≤ p_0_ ) in favor of the alternative one (_W1_: p ≥ p_1_) and thus make a positive decision regarding the possibility of further study of Treamide in phase III.

Taking into account the possible early withdrawal (impossibility of evaluating the primary endpoint) not exceeding 13%, 30 patients will be included in each group of study therapy. Thus, 60 patients will be randomized into the study.

**Efficacy analysis**

According to the RP algorithm A'Hern for single-stage phase II studies with unilateral α=0.05 and 80% power, 11 or more of 26 patients included in the analysis who achieved a clinically significant change in FVC and/or DLCO at Week 4 from baseline are sufficient to accept decisions on the advisability of further study of this dose in phase III (taking into account the evaluation of additional parameters of efficacy and safety). If the placebo arm also has a Week 4 response rate of 11 or more in the 26 included patients, then the primary endpoint assessment may be deferred to an earlier study visit.

If the number of responses to therapy in the Treamide group of 26 included patients is 10 or less, it will be concluded that there is no sufficient efficacy.

For secondary endpoints, between-group comparisons of frequencies and categorical data will be made using chi- ^square^ test or Fisher's exact test, continuous data using non-parametric Mann-Whitney test or Student's t-test. Descriptive statistics will be used to assess FC.

**Safety analysis**

Study drug dosing information, including daily doses, time of exposure, and total dose, will be presented descriptively for each cohort.

Safety will be assessed based on the incidence of AEs and SAEs based on subjective complaints, vital signs, SpO2, physical examination, laboratory findings, and ECG. The number and percentage of patients with AEs and SAEs will be tabulated by organ system class and preferred term (according to MedDRA), by study drug association, and severity. The results of laboratory studies will be summarized in tables of changes. Vital and laboratory parameters, SpO2 and ECG data will be presented using descriptive statistics.

**Interim report**

Based on the results of all patients completing the treatment period (week 4), an interim report can be prepared. The analysis will be based on the evaluation of the endpoints: the frequency of achieving a clinically significant change in FVC and / or DLCO at Week 4 from baseline, the average change in distance traveled over 6 minutes (6MWD) at Week 2 and Week 4 from baseline (based on the Test 6 minute walk). mean change in Borg score at Week 2 and Week 4 from baseline (based on the 6 Minute Walk Test), mean relative change in spirometry lung function parameters (FEV1, FVC, FEV1/FVC) at Week 1, Week 2, Week 3 and Week 4 from baseline, mean relative change in body plethysmography lung function parameters at Week 2 and Week 4 from baseline (DLCO, TLC, and FRC), rate of decline (improvement) in CT at Week 4 from baseline , mean change in severity on the mMRC Breathlessness Scale at Week 1, Week 2, Week 3 and Week 4 from baseline, mean change from baseline on the KBILD Questionnaire total score at Week 2 and Week 4 from baseline. and also based on safety assessment (based on the incidence of AE and SAE according to subjective complaints, physical examination, vital signs, laboratory tests and ECG).

The interim report will be submitted for consideration to the Ministry of Health of the Russian Federation.

**Number and date of the current version of the protocol:**

Amendment 1 of 18 September 2020 to COVID-TRE-03 of 20 July 2020

**TABLE OF CONTENTS**

[PROTOCOL APPROVAL PAGE 2](#_Toc102121411)

[STATEMENT FROM PRINCIPAL INVESTIGATOR 3](#_Toc102121412)

[CLINICAL STUDY CONTACT 4](#_Toc102121413)

[SYNOPSIS 5](#_Toc102121414)

[TABLE OF CONTENTS 14](#_Toc102121415)

[LIST OF ABBREVIATIONS AND DEFINITIONS OF TERMS 17](#_Toc102121416)

[1 INTRODUCTION 19](#_Toc102121417)

[2 OBJECTIVES OF THE CLINICAL STUDY 21](#_Toc102121418)

[2.1 primary goal 21](#_Toc102121419)

[2.2 Additional goals 21](#_Toc102121420)

[3 RESEARCH PLAN 22](#_Toc102121421)

[3.1 General design and study plan 22](#_Toc102121422)

[3.1.1 Screening 22](#_Toc102121423)

[3.1.2 Study therapy period 22](#_Toc102121424)

[3.1.3 Pharmacokinetic study 23](#_Toc102121425)

[3.1.4 Follow-up period. 23](#_Toc102121426)

[3.2 Study Design Rationale 26](#_Toc102121427)

[3.2.1 Rationale for placebo 26](#_Toc102121428)

[3.2.2 Randomization 26](#_Toc102121429)

[3.2.3 Investigational drug, doses, duration of administration 27](#_Toc102121430)

[3.2.4 Substantiation of the relevance of the preclinical model 28](#_Toc102121431)

[3.2.5 Endpoints 28](#_Toc102121432)

[3.2.6 Plan for further clinical development 29](#_Toc102121433)

[3.3 Study duration and dates 29](#_Toc102121434)

[3.4 Previous application experience 29](#_Toc102121435)

[3.4.1 Preclinical studies 29](#_Toc102121436)

[3.4.2 Clinical researches 32](#_Toc102121437)

[3.4.3 Risk/benefit ratio 33](#_Toc102121438)

[4 POPULATION SELECTION 34](#_Toc102121439)

[4.1 Study population 34](#_Toc102121440)

[4.2 Inclusion Criteria 34](#_Toc102121441)

[4.3 Non-inclusion сriteria: 35](#_Toc102121442)

[4.4 Exclusion of patients from the study after randomization 36](#_Toc102121443)

[5 STUDY DRUG 37](#_Toc102121444)

[5.1 Description of study drug 37](#_Toc102121445)

[5.2 Study drug intake 37](#_Toc102121446)

[5.3 Method of allocation of patients to treatment groups 37](#_Toc102121447)

[5.4 Blinding of study drug 38](#_Toc102121448)

[5.5 dazzle 38](#_Toc102121449)

[5.6 Compliance with the therapy 38](#_Toc102121450)

[5.7 Concomitant Therapy 38](#_Toc102121451)

[5.8 Prohibited Therapy 38](#_Toc102121452)

[5.9 Packaging and labeling of investigational product and concomitant therapy 39](#_Toc102121453)

[5.10 Storage and record keeping of the study drug 39](#_Toc102121454)

[6 DESCRIPTION OF PROCEDURES 40](#_Toc102121455)

[6.1 Informed consent 40](#_Toc102121456)

[6.2 Patient Registration 40](#_Toc102121457)

[6.3 Demographics and medical history 40](#_Toc102121458)

[6.4 Physical examination 40](#_Toc102121459)

[6.5 Vital signs and measurement of blood oxygen saturation (SpO2) 40](#_Toc102121460)

[6.6 Electrocardiography 40](#_Toc102121461)

[6.7 CT scan of the chest 41](#_Toc102121462)

[6.8 Analyzes performed in the laboratory 42](#_Toc102121463)

[6.8.1 Laboratory parameters 42](#_Toc102121464)

[6.8.2 PCR for SARS-CoV-2 RNA 43](#_Toc102121465)

[6.8.3 Pharmacokinetic study 43](#_Toc102121466)

[6.8.4 Receipt, preparation, storage and dispatch of biosamples 43](#_Toc102121467)

[6.9 mMRC (modified Medical Research Council) breathlessness scale 44](#_Toc102121468)

[6.10 6 Minute Walk Test (6MWD, Borg Scale) 44](#_Toc102121469)

[6.11 Questionnaire KBILD 44](#_Toc102121470)

[6.12 Spirometry (FEV1, FVC, FEV1/FVC) 45](#_Toc102121471)

[6.13 Body plethysmography (DLCO, TLC, FRC) 45](#_Toc102121472)

[6.14 Assessment of adverse events (AEs) 45](#_Toc102121473)

[6.14.1 Definitions of AE 45](#_Toc102121474)

[6.14.2 Pregnancy 46](#_Toc102121475)

[6.14.3 AE reporting 46](#_Toc102121476)

[6.14.4 Severity assessment 47](#_Toc102121477)

[6.14.5 Association of AEs with study drug 47](#_Toc102121478)

[6.14.6 Reporting SAEs/Pregnancy 48](#_Toc102121479)

[6.14.7 Withdrawal of patients from the study 49](#_Toc102121480)

[6.14.8 Procedures for dealing with emergencies 49](#_Toc102121481)

[6.14.9 Registration deadlines for AEs 49](#_Toc102121482)

[6.14.10 Expectancy 49](#_Toc102121483)

[6.14.11 Clinically Significant Laboratory Abnormalities 49](#_Toc102121484)

[6.15 Early withdrawal of patients from the study or withdrawal of the study drug 49](#_Toc102121485)

[6.16 Validity of measurements 50](#_Toc102121486)

[7 RESEARCH PROCEDURES 51](#_Toc102121487)

[7.1 Screening 51](#_Toc102121488)

[7.2 Study therapy period 51](#_Toc102121489)

[7.2.1 Visit 2, Week 0 51](#_Toc102121490)

[7.2.2 Visit 3, Week 1, Visit window ± 1 day 52](#_Toc102121491)

[7.2.3 Visit 4, Week 2, Visit window ± 2 days 52](#_Toc102121492)

[7.2.4 Visit 5, Week 3, Visit window ± 1 day 53](#_Toc102121493)

[7.2.5 Visit 6, Week 4, Visit window ± 2 days (End of therapy) 53](#_Toc102121494)

[7.3 observation period. 54](#_Toc102121495)

[7.3.1 Visit 7, Week 6, Visit window ± 3 days 54](#_Toc102121496)

[7.4 Unscheduled examinations 54](#_Toc102121497)

[7.5 Early termination of the study 54](#_Toc102121498)

[8 QUALITY ASSURANCE 55](#_Toc102121499)

[9 PLANNED STATISTICAL METHODS 56](#_Toc102121500)

[9.1 General provisions 56](#_Toc102121501)

[9.2 Determination of the sample size 56](#_Toc102121502)

[9.3 Randomization 56](#_Toc102121503)

[9.4 Populations for analysis 56](#_Toc102121504)

[9.4.1 Full analysis set (FAS) 56](#_Toc102121505)

[9.4.2 Protocol Population (Per Protocol, PP) 56](#_Toc102121506)

[9.4.3 Safety Population 56](#_Toc102121507)

[9.4.4 PK Population 56](#_Toc102121508)

[9.5 Patient distribution, demographic characteristics and baseline analysis 57](#_Toc102121509)

[9.6 Procedures for accounting for missing, unanalysable and doubtful data 57](#_Toc102121510)

[9.7 Efficacy analysis 57](#_Toc102121511)

[9.7.1 Primary efficacy endpoint 57](#_Toc102121512)

[9.7.2 Secondary efficacy endpoints 57](#_Toc102121513)

[9.8 Safety analysis 58](#_Toc102121514)

[9.9 Pharmacokinetic analysis 58](#_Toc102121515)

[9.10 Interim report 58](#_Toc102121516)

[10 ADMINISTRATIVE PROCEDURES 59](#_Toc102121517)

[10.1 Legal aspects 59](#_Toc102121518)

[10.1.1 Responsibilities of the Investigator 59](#_Toc102121519)

[10.2 Monitoring procedures 59](#_Toc102121520)

[10.3 Data registration in eCRF 59](#_Toc102121521)

[10.4 Storage of documentation 59](#_Toc102121522)

[10.5 Ethical aspects 60](#_Toc102121523)

[10.5.1 Independent Ethics Committee 60](#_Toc102121524)

[10.5.2 Ethical Conduct of Clinical Research 60](#_Toc102121525)

[10.5.3 Informed Consent 60](#_Toc102121526)

[10.6 Research funding 61](#_Toc102121527)

[11 PLAN OF PUBLICATIONS 62](#_Toc102121528)

[12 REFERENCES 63](#_Toc102121529)

**LIST OF TABLES**

[Table 1. Schedule of procedures according to the COVID-TRE-03 protocol 25](#_Toc102121530)

[Table 2. List of indicators of laboratory tests of blood and urine 42](#_Toc102121531)

[Table 3 AE severity assessment 47](#_Toc102121532)

[Table 4 Assessing relation to the study drug use 48](#_Toc102121533)

**LIST OF FIGURES**

[Picture 1. COVID-TRE-03 Study Design 24](#_Toc102121534)

**LIST OF APPS**

[Appendix 1. mMRC (modified Medical Research Council) dyspnea score 66](#_Toc102121535)

[Annex 2. 6-minute walk test (6MWD, Borg scale) (sample) 67](#_Toc102121536)

[Annex 3. King Interstitial Lung Disease Scoring Questionnaire (KBILD) - Sample 69](#_Toc102121537)

LIST OF ABBREVIATIONS AND DEFINITIONS OF TERMS

| 6MWD | 6-min walk distance |
| --- | --- |
| CD117 | Protein Tyrosine Kinase Kit |
| CD45 | Tyrosine protein phosphatase |
| DLCO | Diffusing capacity of the lungs for carbon monoxide |
| EMEA | European Medicines Agency |
| ERV | Residual air in the lungs |
| FEV | Forced expiratory volume |
| FEV1 | Forced expiratory volume in one second of a forced expiratory maneuver |
| FRC | Functional residual lung capacity |
| IWRS | Interactive Web Response System |
| KBILD | King's brief interstitial lung disease scoring questionnaire |
| mMRC | Shortness of breath scale (modified medical research council) |
| NCI-CTCAE | Common Terminology Criteria for Adverse Events National Cancer Institute, USA |
| Notch | Human transmembrane receptor protein (notch homolog 1, translocation-associated (Drosophila)) |
| per os | oral intake |
| RV | Total expiratory reserve volume |
| TLC | Total lung capacity |
| VEGF | Vascular endothelial growth factor |
| BP | Blood pressure |
| BAD | Biologically active additives |
| HIV | Human immunodeficiency virus |
| VMR | Temporary guidelines. |
| WHO | World Health Organization |
| E/T | Early termination |
| EAEU | **EURASIAN ECONOMIC UNION** |
| IVL | Artificial lung ventilation |
| IPF | Idiopathic pulmonary fibrosis |
| KT | CT scan |
| MSME | International Health Regulations |
| NIVL | Non-invasive ventilation |
| AR | Adverse reaction |
| IEC | Independent Ethics Committee (local) |
| AE | Adverse event |
| ARDS | Acute Respiratory Distress Syndrome |
| ORIT | Department of intensive care and intensive care |
| OF | End of therapy |
| PCR | Polymerase chain reaction |
| RNA | Ribonucleic acid |
| CHNR | Serious unexpected adverse reaction |
| CHP | Serious adverse reaction |
| SAE | Serious adverse event |
| SOP | Standard Operating Procedures |
| TD | Therapeutic dose |
| RR | Respiration rate |
| HR | Heart rate |
| eCRF | Electronic Case Report Form |
| ECG | - electrocardiogram |

1. **INTRODUCTION**

Starting in December 2019, the novel coronavirus SARS-CoV-2 has caused an outbreak of a respiratory disease called COVID-19 in almost every country in the world.

Clinical and epidemiological observations of patients with COVID-19 indicate that SARS-CoV-2 infection can range from mild signs of respiratory disease to severe progressive pneumonia, multiple organ failure, and death [1, 2, 3, 4, 5]. The presence of foci of lung tissue seals of the "ground glass" type and / or seals of interlobular septa, and / or areas of reticular changes in the lung tissue, and / or the presence of areas (linear, focal) of lung tissue seals according to chest CT data leads to the need for long-term rehabilitation patients who have had COVID-19 pneumonia [43, 44, 45].

According to modern concepts, pulmonary fibrosis is the process of formation of fibrous (scar) tissue in the lungs, which leads to impaired respiratory function. With fibrosis, the elasticity and extensibility of the lung tissue decreases, the passage of oxygen and carbon dioxide through the wall of the alveoli (lung vesicles, in which the inhaled air contacts the blood) is difficult.

Patients who have undergone COVID-19 are characterized by the development of pulmonary edema, accompanied by the presence of neutrophil-macrophage inflammation [6], as well as changes in respiratory function, in particular, a decrease in forced expiratory volume (FEV1) [7], a decrease in forced vital capacity (FVC) [7] and maximum expiratory flow rate at the level of 50% forced vital capacity (MEF50) [8].

Currently, any specific pharmacotherapy for patients at the stage of rehabilitation after suffering from COVID-19 pneumonia has not been described. Thus, the search for effective and safe drugs that could be effective in patients with fibrosis remains an urgent task [16].

Antifibrotic therapy, which has demonstrated activity in a model of pulmonary fibrosis induced by intratracheal administration of bleomycin, may be useful in the treatment of COVID-19 both in the acute phase of the disease and in the prevention and treatment of long-term complications [9]. Indirect confirmation of this fact is the conduct of clinical trials of drugs developed for the treatment of chronic fibrotic diseases, in particular, drugs Nintedanib [10] and Pirfenidone [11] for the treatment of pulmonary fibrosis caused by COVID-19 infection.

PHARMENTERPRICES LLC is developing a new drug Treamide to correct the development of fibrotic changes in the lungs at the stage of rehabilitation of patients after COVID-19 pneumonia.

The innovative drug Treamide is a bis-amide derivative of dicarboxylic acid. It was developed for use in patients with metabolic syndrome and age-related androgen deficiency. The mechanism of action of the drug formed the basis of its preclinical evaluation with the aim of repositioning for use in patients with COVID-19 pneumonia.

According to preclinical data, Treamide has anti-inflammatory and antifibrotic effects in models of lung injury, prevents tissue destruction, and improves respiratory function. Under conditions of pneumofibrosis, Treamide significantly (p<0.05) reduces the levels of total collagen, type I collagen, hydroxyproline, and the amount of deposited connective tissue in the lungs in mice. In a model of pulmonary fibrosis induced by intratracheal administration of bleomycin in mice, Treamide showed pronounced antifibrotic activity at a dose of 10 mg/kg per day. The model of fibrosis induced by intratracheal administration of bleomycin reproduces well the fibrotic response after acute lung injury (characteristic, in particular, of COVID-19 infection), rather than *de novo* progressive fibrosis [12]. It is important to note that the introduction of Treamide at a lower dose of 1 mg/kg did not lead to significant changes in the lung tissue in mice on the 21st day of the experiment. A potentially effective dose of 10 mg/kg per day in mice is approximately equivalent to 50 mg per day in humans.

Possible mechanisms of action of Treamide are an inhibitory effect on the migration of inflammatory cells to the lungs, as well as a decrease in the activity of synthesis and deposition of connective tissue.

The basis of the regenerative effects of Treamide is an inhibitory effect on the Notch-mediated mechanism for the development of pulmonary fibrosis, and stimulation of the differentiation of lung stem cells (CD45-CD117+) and VEGF2+ endothelial cells.

In a phase I clinical study in healthy volunteers, Treamide showed good tolerability and a favorable safety profile at doses of 5 mg to 50 mg per day in single and multiple doses for 14 days. 4 AEs (asymptomatic grade 3 and 4 laboratory changes) were reported only in the low dose cohort; No AEs associated with Treamide have been reported.

Based on preclinical data on the efficacy of Treamide in the most relevant disease model (a model of pulmonary fibrosis induced by intratracheal administration of bleomycin), as well as clinical data on the safety of Treamide at a dose of 50 mg once daily in humans, a phase II pilot clinical study is planned. to assess the safety and efficacy of Treamide at a dose of 50 mg once a day for 4 weeks in patients with fibrotic lung damage caused by the development of COVID-19 infection.

After the completion of this study, with the confirmation of the pilot hypothesis (proof-of-concept), it is planned to continue evaluating the efficacy and safety of Treamide in a multicenter, placebo-controlled, randomized phase III clinical trial of the efficacy and safety of Treamide in patients with fibrotic changes in the lungs after suffering COVID-19 pneumonia. .

1. OBJECTIVES OF THE CLINICAL STUDY
   1. **primary goal**

The primary objective of the study is to evaluate the efficacy of Treamide versus placebo in patients with COVID-19 pneumonia based on the rate of achieving a clinically significant change in forced vital capacity (FVC) and/or diffusive capacity of the lungs (DLCO) at Week 4 relative to initial values.

Clinically significant changes are considered to be a relative increase in FVC by ≥ 10% ***or*** a relative increase in FVC in the range from ≥ 5% to < 10% and a relative increase in DLCO by ≥ 15% [13, 14].

- 1. **Additional goals**

Additional study objectives include evaluating the efficacy and safety of Treamide versus placebo in patients with COVID-19 pneumonia based on the following parameters:

- Change in distance traveled in 6 minutes (6MWD) in Week 2 and Week 4 from baseline (based on the 6 Minute Walk Test);
- Change in Borg score at Week 2 and Week 4 from baseline (based on the 6 Minute Walk Test);
- Relative change in spirometry lung function parameters (forced expiratory volume in one second (FEV1), FVC, FEV1/FVC) at Week 1, Week 2, Week 3, and Week 4 from baseline;
- Relative change in body plethysmography lung function parameters at Week 2 and Week 4 from baseline (DLCO, total lung capacity (TLC), expiratory reserve volume (FRC));
- Change in the degree of lung damage according to computed tomography (CT) at Week 4 from baseline;
- Change in severity on the mMRC Breathlessness Scale at Week 1, Week 2, Week 3, and Week 4 from baseline;
- Change in total score on the King's Interstitial Lung Disease Questionnaire (KBILD) at Week 2 and Week 4 from baseline;
- PK parameters of the active substance of the drug Treamide (XC268BG);
- The frequency of adverse events (AE) and serious adverse events (SAE) of varying severity according to subjective complaints, physical examination, vital signs, laboratory tests and ECG.

1. RESEARCH PLAN
   1. **General design and study plan**

This study is a multicenter, randomized, double-blind, placebo-controlled, phase II pilot study to evaluate the efficacy and safety of Treamide in the rehabilitation of patients after COVID-19 pneumonia [15].

The study will be conducted in approximately 6-10 Russian clinical centers.

A total of 60 patients are planned to be included in the study. Considering a possible 43% drop-out at the study selection stage, approximately 90 patients at risk of developing fibrotic changes in the lungs after suffering from COVID-19 pneumonia will be screened.

- - 1. **Screening**

At the Week -2 visit, following the signing of the Patient Information Sheet and the Informed Consent Form, screening procedures will be performed to assess inclusion/non-inclusion criteria. Screening procedures include the collection of demographic data, medical history and concomitant therapy, physical examination, measurement of height, body weight and BMI, assessment of vital signs and SpO2, ECG, mMRC dyspnea score, clinical and biochemical blood tests, urinalysis. Women of childbearing potential will be given a pregnancy test.

All patients will undergo a qualitative determination of SARS-CoV-2 RNA by PCR. The material for laboratory testing will be a swab from the nasopharynx and / or oropharynx. Confirmation of two consecutive negative PCR results at least 24 hours apart is required for inclusion in the study. In the case of previously confirmed elimination of the SARS-CoV-2 virus according to medical records, one determination of SARS-CoV-2 RNA by PCR with a negative result will be sufficient.

Patients will be assessed using the mMRC Breathlessness Scale, chest CT, spirometry, and body plethysmography with DLCO. These examinations should be conducted as close as possible to the planned date of randomization (not earlier than 5 days before it).

**In the event that screening procedures are initiated as part of a patient's discharge from the hospital after hospitalization for COVID-19 pneumonia, sufficient time should be allowed for the completion of quarantine measures (about 2 weeks) before assessing the extent of damage and lung function.**

**The screening may use the results of examinations carried out as part of routine medical practice before obtaining informed consent (taking into account the window of the screening visit), except for the assessment on the mMRC Breathlessness Scale, CT of the chest, spirometry and body plethysmography with the definition of DLCO - these examinations should be carried out as much as possible close to the planned date of randomization (no earlier than 5 days before it).**

Patients who meet all eligibility criteria will be included in the study.

- - 1. **Study therapy period**

At visit Week 0 prior to study therapy, registration of AEs and concomitant therapy, physical examination, body weight measurement, vital signs and SpO2 assessment, mMRC dyspnoea score, ECG, clinical and biochemical blood tests, and urinalysis will be performed. Women of childbearing potential will be given a pregnancy test. Patients will complete a KBILD questionnaire, followed by a 6 Minute Walk Test, which measures the distance the patient walks in 6 minutes and is assessed using the Borg Scale.

After completing all visit procedures, patients will be randomized into two groups in a 1:1 ratio.

Group 1 Treamide 50 mg - 30 patients

Group 2 Placebo - 30 patients

Patients will be given the required amount of study drug until the next visit, according to the therapy group.

The duration of study therapy will be 4 weeks. Patients will be recommended to continue the standard program of medical rehabilitation in a day hospital or outpatient setting (Stage 3 according to the Interim Guidelines "Medical Rehabilitation for Novel Coronavirus Infection (COVID 19)", Version 1 dated 05/21/2020 or current at the time of the study) [ sixteen].

Patients will visit the study site once a week during the study therapy phase. At Week 1, Week 2, and Week 3 visits, registration of AEs and concomitant medications, study medication, body weight, vital signs and SpO2, mMRC dyspnea score, and spirometry will be performed. Women of childbearing potential will be given a pregnancy test.

At the Week 2 visit, physical examination, ECG, CBC, CBC, PK blood sampling, urinalysis, body plethysmography with DLCO will also be performed. Patients will complete the KBILD questionnaire. There will also be a 6 Minute Walk Test, which will measure the distance the patient walks in 6 minutes and evaluate with the Borg Scale. Women of childbearing potential will be tested for pregnancy

At visit Week 4 (end of therapy), registration of AEs and concomitant therapy, accounting for study drug, physical examination, measurement of body weight, assessment of vital signs and SpO2, ECG, clinical and biochemical blood tests, blood sampling for PK, urinalysis . Patients will be assessed using the mMRC Breathlessness Scale, chest CT, spirometry, and body plethysmography with DLCO. Patients will complete a KBILD questionnaire, followed by a 6 Minute Walk Test, which measures the distance the patient walks in 6 minutes and is assessed using the Borg Scale. After completion of study therapy, patients will continue treatment in accordance with general recommendations. Women of childbearing potential will be tested for pregnancy

- - 1. **Pharmacokinetic study**

A blood draw for the Ctrough PK study will be performed on all patients prior to PI administration at Week 0, Week 2, and Week 4.

- - 1. **Follow-up period.**

Patient follow-up will continue for another two weeks. At Week 6, registration of AEs and concomitant therapy, physical examination, measurement of body weight, assessment of vital signs and SpO2, mMRC dyspnea score, ECG, clinical and biochemical blood tests, urinalysis will be carried out. Women of childbearing potential will be given a pregnancy test.

The figure below (Figure 1) shows the Schematic of the Clinical Study.

The schedule of examinations and the time of their implementation are presented in the table below (Table 1).

Picture 1. COVID-TRE-03 Study Design

|  | Screening | Investigational Therapy | | | | Follow-up |  |
| --- | --- | --- | --- | --- | --- | --- | --- |
|  |  |  | | | |  |  |
|  | ~90 patients | Treamide 50 mg (n=30) | | | |  |  |
|  |  | Placebo (n=30) | | | |  |  |
|  |  |  |  |  |  |  |  |
| Visit | V 1 | V 2 | V 3 | V 4 | V 5 | V 6 | V 7 |
| Week | W-2 | W0 | W1 | W2 | W3 | W4[[8]](#footnote-8) | W6 |

Table 1. Schedule of procedures according to the COVID-TRE-03 protocol

| **Procedures** | **Screening 2[[9]](#footnote-9)** | **Investigational Therapy** | | | | | **Follow-up** | **E/T** |
| --- | --- | --- | --- | --- | --- | --- | --- | --- |
| Visit (V) | V 1 | V 2 | V 3 | V 4 | V 5 | V 6 | V 7 |  |
| Week (W) | W-2 | W0 | W1 | W2 | W3 | W4 (ET) | W6 |  |
| Visit/procedures window | Day -28...0 3[[10]](#footnote-10) |  | ± 1 day | ± 2 days | ± 1 day | ± 2 days | ± 3 days |  |
| Informed consent | X |  |  |  |  |  |  |  |
| Demographic data | X |  |  |  |  |  |  |  |
| Medical history (including history of COVID-19) | X |  |  |  |  |  |  |  |
| Physical examination | X | X |  | X |  | X | X | X |
| Body weight, height, BMI 4[[11]](#footnote-11) | X | X | X | X | X | X | X | X |
| Vital signs, SpO2 | X | X | X | X | X | X | X | X |
| SARS-CoV-2 RNA by PCR 5[[12]](#footnote-12) | X |  |  |  |  |  |  |  |
| Complete blood count 6[[13]](#footnote-13) | X | X |  | X |  | X | X | X |
| Blood chemistry; | X | X |  | X |  | X | X | X |
| PK study 7[[14]](#footnote-14) |  | Х |  | Х |  | Х |  |  |
| General urine analysis | X | X |  | X |  | X | X | X |
| Pregnancy test 8[[15]](#footnote-15) | X | X |  | X |  | X | X | X |
| 12-lead ECG | X | X |  | X |  | X | X | X |
| mMRC breathlessness scale | Х | X | X | X | X | X | X | X |
| 6 Minute Walk Test (6MWD, Borg Scale) |  | X |  | X |  | X |  | X |
| Questionnaire KBILD |  | X |  | X |  | X |  | X |
| chest CT | X |  |  |  |  | X |  | X |
| Spirometry (FEV1, FVC, FEV1/FVC) | X | X | X | X | X | X |  | X |
| Body plethysmography (DLCO, TLC, FRC) | X |  |  | X |  | X |  | X |
| Evaluation of inclusion / non-inclusion criteria | X | X |  |  |  |  |  |  |
| Randomization |  | X |  |  |  |  |  |  |
| Distribution of the study drug |  | X | X | X | X |  |  |  |
| Study drug return and compliance assessment |  |  | X | X | X | X |  | X |
| Concomitant therapy | X | X | X | X | X | X | X | X |
|  |  |  |  |  |  |  |  |  |
| Adverse Events | X | X | X | X | X | X | X | X |

Abbreviations: W - week, D - day, ET - end of therapy, D/3 - early termination.

- 1. **Study Design Rationale**

This study is double-blind, randomized, placebo-controlled. The placebo control design was chosen to objectify the findings.

This study is a phase II pilot study in patients at risk of developing pulmonary fibrosis after prior COVID-19 pneumonia. This study will provide initial data on the efficacy and safety of Treamide 50 mg once daily with a view to further evaluating efficacy and safety in phase III.

Patients were selected as the study population, in whom, after clinical recovery from COVID-19 pneumonia, residual changes in the lungs persist, indicating a risk of developing fibrotic changes in the lung tissue. The diagnosis of COVID-19 in history should be confirmed by a positive qualitative analysis of SARS-CoV-2 RNA by PCR; the onset of the disease (appearance of the first symptoms) no more than 2 months before screening. The presence of foci of ground glass lung tissue compaction and / or interlobular septal seals, and / or areas of reticular changes in the lung tissue, and / or the presence of areas (linear, focal) lung tissue seals characteristic of COVID-19 should be confirmed according to chest CT at screening.

- - 1. **Rationale for placebo**

According to the WMA Declaration of Helsinki, the use of a placebo in a study is possible when, for compelling and scientifically sound methodological reasons, the use of a placebo is necessary to evaluate the efficacy or safety of the intervention under study. In this case, patients receiving placebo will not be exposed to the additional risk of causing serious or irreversible damage to health as a result of not receiving the best of the interventions already proven.

Subject to this requirement, only patients with confirmed negative SARS-CoV-2 RNA quantification by PCR at screening and with a decrease in FVC and/or DLCO lung function < 80% predicted will be included in the study.

According to the concept of the Belmont Report [21], in relation to this study, a placebo-controlled design is preferable because:

- Currently, any specific pharmacotherapy for patients at the stage of rehabilitation after suffering from COVID-19 pneumonia has not been described [16];
- the risks of taking a placebo are low (pulmonary rehabilitation for patients who have undergone new community-acquired pneumonia COVID-19 does not currently provide for specific pharmacotherapy) [16];
- The effectiveness of a drug with a similar mechanism of action (Nintedanib) has not yet been proven, the study of the efficacy and safety of Nintedanib in the treatment of pulmonary fibrosis in patients with moderate to severe COVID-19 will be completed no earlier than August 2020 [22].

Thus, the use of placebo in this study is ethical and evidence-based, which complies with the requirements of the WMA Declaration of Helsinki.

In addition, if the patient's condition deteriorates due to increased respiratory symptoms, decreased lung function, or episodes of acute respiratory deterioration, the patient will be withdrawn from the study and, if necessary, admitted to a hospital for medical care. Criteria for early withdrawal of patients from the study are presented in section 6.14.7.

- - 1. **Randomization**

Patients will be randomized into two groups in a 1:1 ratio using the IWRS system. Randomization will be stratified by

1. presence or absence of risk factors (age ≥ 60 years and/or presence of concomitant chronic diseases)
2. and the initial degree of lung damage according to CT data (CT 1, CT 2, CT 3 and CT 4)

The stratification method will be described in more detail in the randomization protocol.

The stratification will make the groups comparable in terms of these indicators and thus will allow to level the possible influence of these factors on the response rate in both groups of study therapy.

- - 1. **Investigational drug, doses, duration of administration**

The drug Treamide will be taken orally, once a day, in the morning, 30 minutes before breakfast. The duration of taking the drug Treamide will be 4 weeks (28 days). This time interval was chosen as necessary and sufficient to obtain primary data on the effectiveness of Treamide in patients at risk of developing fibrotic changes in the lungs after suffering COVID-19 pneumonia based on data from preclinical and clinical studies, as well as on the basis of the duration of treatment with registered drugs with a similar mechanism of action [17].

In a phase I study in healthy volunteers, the safety and tolerability of Treamide 50 mg administered once daily for 14 days of the study was shown. None of the AEs reported during the study met the criteria for SAEs and, in the opinion of the Investigators, were not related to study medication or placebo. According to a study of FC in healthy volunteers, with a single dose of Treamide at a dose of 50 mg, T1 / 2 was 11.6 hours, with multiple doses - 12.1 hours. The cumulation factor (Fc) was 1.17. With repeated administration of 50 mg 1 time per day, the total exposure of the drug Treamide in blood plasma increases by 20% compared with a single dose. Thus, it is assumed that taking once a day is reasonable [23].

According to preclinical data, Treamide has anti-inflammatory and antifibrotic effects in models of lung injury, prevents tissue destruction, and improves respiratory function. In a model of pulmonary fibrosis induced by intratracheal administration of bleomycin, Treamide at a dose of 10 mg/kg (corresponding to a dose of 50 mg per day in terms of a person) prevented the infiltration of the interstitium of the alveoli and alveolar ducts by inflammatory cells and the development of fibrosing alveolitis in the lung tissue of mice. In cytometric studies, a decrease in the number of alveolar and interstitial macrophages, dendritic cells and lymphocytes in the lungs of mice of the experimental group was found compared to the pathological control. Treamide at a dose of 10 mg/kg statistically significantly (p<0.05) inhibited the synthesis of total collagen, type I collagen, hydroxyproline and the deposition of fibrous masses in the interstitium of the lungs of animals with pulmonary fibrosis induced by intratracheal administration of bleomycin. It is important to note that the introduction of the drug at a lower dose of 1 mg/kg did not lead to significant changes in the lung tissue in mice on the 21st day of the experiment.

Thus, based on data on the efficacy of Treamide in the most relevant model of the disease (a model of pulmonary fibrosis induced by intratracheal administration of bleomycin), as well as clinical data on the safety of Treamide at a dose of 50 mg once a day in humans, it is planned to conduct a pilot clinical study on to assess the safety, tolerability and efficacy of Treamide 50 mg once daily for 28 days in patients at risk of developing fibrotic changes in the lungs caused by the development of COVID-19 infection.

- - 1. Substantiation of the relevance of the preclinical model

Among the currently used models of experimentally induced pulmonary fibrosis, the bleomycin-induced fibrosis model is the most commonly used [26,27]. The pathogenesis of pulmonary fibrosis induced by intratracheal administration of bleomycin is based on the ability of the drug to cause direct cell damage due to the induction of DNA strand breaks, the formation of free radicals, and subsequent oxidative stress [28]. Overproduction of reactive oxygen species due to metal ion chelation leads to epithelial cell death (days 1-3), excessive infiltration of lung inflammatory cells (neutrophils - days 3-9, lymphocytes on day 6), increased expression of profibrotic cytokines (including IL -1b, IL-6, IL-13, TGF-β) and ultimately to fibroblast activation, extracellular matrix deposition and fibrosis development (days 10-21 with a peak around day 14), these processes are detailed in the molecular [ 27, 29, 30] and histological [31, 32] levels.

It is important to note that the model of fibrosis induced by intratracheal administration of bleomycin reproduces well the fibrotic response after acute lung injury (characteristic, in particular, for COVID-19 infection), rather than de novo progressive fibrosis [34]. During the development of COVID-19 infection, the initial rapid viral replication causes massive death of epithelial and endothelial cells, which leads to increased vascular permeability and induces aberrant production of pro-inflammatory cytokines and chemokines. Excessive production of chemokines (in particular IL-1b, IL-6, IL-13, TGF-β) leads to aberrant chemotaxis of monocytes, macrophages and neutrophils, which produce a large number of reactive oxygen species and cause further damage to surrounding tissues and increase the death of epithelial and endothelial cells [35].

Thus, bleomycin-induced development of oxidative stress associated with damage and death of epithelial and endothelial cells, increased vascular permeability and aberrant infiltration of inflammatory cells in lung tissue well reflects the pathogenetic features of the development of COVID-19 infection [34,33]. Antifibrotic therapy, which has demonstrated activity in a model of pulmonary fibrosis induced by intratracheal administration of bleomycin, may be useful in the treatment of COVID-19 both in the acute phase of the disease and in the prevention and treatment of long-term complications [33]. Indirect confirmation of this fact is the conduct of clinical trials of drugs developed for the treatment of chronic fibrotic diseases, in particular, Nintedanib [36] and Pirfenidone [37] for the treatment of pulmonary fibrosis caused by COVID-19 infection.

- - 1. Endpoints

There are currently no specific guidelines for selecting the primary endpoint in studies in patients with COVID-19-associated pulmonary fibrosis. The primary end point for preliminary efficacy in this study was the frequency of achieving a clinically significant change in FVC and/or DLCO at Week 4 from baseline. This endpoint is a surrogate for patient progress assessments used in phase II studies in patients with IPF. This parameter is pharmacodynamic and allows assessing the direct effect of the study therapy on the function and diffusion capacity of the lungs, which indirectly indicates a possible clinical improvement in the patient's condition. The use of a surrogate endpoint is acceptable in phase II studies for preliminary evaluation of a pilot hypothesis (proof-of-concept) and a decision on the possibility of further study of the drug in phase III studies. Clinically significant changes are considered to be a relative increase in FVC by ≥ 10% *or* a relative increase in FVC in the range from ≥ 5% to < 10% and a relative increase in DLCO by ≥ 15% [13, 14].

In addition, additional efficacy parameters will be assessed in the study based on the 6 Minute Walk Test, lung function and diffusivity assessment, CT scan of the lungs, as well as the mMRC Breathlessness Scale and the KBILD Questionnaire. Based on the preliminary efficacy data obtained in this study, the optimal duration of treatment will be selected for further study and the most relevant endpoint, which will be used as primary in the phase III clinical trial.

The incidence of AEs and SAEs will be assessed based on subjective complaints, physical examination, vital signs, laboratory tests, and ECG.

- - 1. Plan for further clinical development

In case of successful completion of this study, it is planned to conduct a multicenter, double-blind, placebo-controlled, randomized Phase III clinical trial to evaluate the efficacy and safety of Treamide in patients with pulmonary fibrosis after COVID-19 pneumonia. In parallel, documents will be submitted for obtaining a temporary registration certificate for the Treamide drug as part of the procedure for Decree of the Government of the Russian Federation of 03.04.2020 N 441.

- 1. Study duration and dates

Each patient will participate in the study for approximately 8 weeks: screening 2 weeks, study therapy period 4 weeks, follow-up period 2 weeks. The start of enrollment in the study is scheduled for August 2020. It is planned that all patients will complete all study visits by November 2020.

- 1. Previous application experience
     1. Preclinical studies
        1. Preclinical pharmacology

The mechanism of action of Treamide is associated with the inhibition of notcW1-mediated proliferation of various types of progenitor notcW1+ cells and the induction of their differentiation into specialized (notcW1-) cells. This mechanism has been shown in *in vitro experiments* and confirmed in *in vivo* disease models.

Inhibition of notcW1-mediated proliferation and a decrease in the total number of notcW1+ cells, which occurs under the action of Treamid, leads to suppression of notcW1+-mediated production of connective tissue growth factor [38, 39] at an early stage of the disease, as well as components of the extracellular matrix (fibronectin and type I collagen) at an early stage of the disease. later stages of pathology. In addition, the suppression of notcW1+ leads to a decrease in the activation and migration of macrophages into lung tissue. Since macrophages play a key role in the synthesis of the extracellular matrix (macrophage arginase is the most important enzyme in the synthesis of proline and collagen), as well as in the development of the inflammatory response and the production of interleukins, a decrease in the migration of macrophages into lung tissues leads to the suppression of fibrosis.

In addition, activation of differentiation, leading to an increase in the number of mature epithelial and endothelial cells in lung tissue, is the central mechanism for accelerating lung regeneration in pulmonary fibrosis, which was confirmed by an increase in the number of CD31+ mature endothelial cells in lung tissue.

In *in vitro* experiments, the direct effect of the drug Treamide on the precursors of endothelial and epithelial cells of the lungs of intact control and pathological control was evaluated. In the culture of epithelial and endothelial cells isolated from the lungs of mice of the experimental group on the 21st day of the experiment, Treamide did not affect CD45-Ter119-CD49f+ epithelial progenitors and CD45-CD31+CD34+ endothelial progenitor cells expressing notcW1. Meanwhile, *in vitro*, the direct action of the substance reduced the number of angiogenesis precursor cells (CD45-CD117+Flk1+) and lung stem cells (CD45-CD117+) expressing notcW1. Thus, the regenerative effects of Treamide may be based on an inhibitory effect on the notch-mediated mechanism of IPF development, and stimulation of the differentiation of lung stem cells (CD45-CD117+) and VEGF2+ endothelial cells.

The study of the antifibrotic properties of the drug Treamide on the model of bleomycin-induced pneumofibrosis was carried out on mice. The model of fibrosis induced by intratracheal administration of bleomycin reproduces well the fibrotic response after acute lung injury (characteristic in particular for COVID-19 infection) rather than *de novo* progressive fibrosis [12]. The introduction of the drug at a dose of 1 mg/kg did not lead to significant changes in the lung tissue in mice on the 21st day of the experiment. Treamide at a dose of 10 mg/kg statistically significantly (p<0.05) inhibited the synthesis of total collagen, collagen type I, hydroxyproline and the deposition of fibrous masses in the interstitium of the lungs, as well as tissue destruction in the lungs in animals with pulmonary fibrosis induced by intratracheal administration of bleomycin .

The anti-inflammatory properties of Treamide were studied in a mouse model of bleomycin-induced pneumofibrosis. The introduction of Treamide at a dose of 10 mg/kg in the inflammation phase contributed to a decrease in the level of IL-13 and connective tissue growth factor, as well as to the normalization of the Clara cell protein level in lung homogenates of mice with pneumofibrosis compared with pathological control.

Also, the anti-inflammatory properties of the drug Treamide were studied on a model of neutrophil-macrophage inflammation of the lung, accompanied by impaired respiratory function in guinea pigs. The course administration of Treamide significantly increased the mid-expiratory airflow rate (EF50) and peak expiratory flow (PEF) to the intact level. The results obtained allow us to conclude that the pharmaceutical substance of Treamide has a pronounced anti-inflammatory effect and its pharmacological activity in relation to the normalization of respiratory function in pathologies accompanied by a decrease in peak expiratory flow rate (PEF) and mid-expiratory airflow rate (EF50), which may indicate a therapeutic potential drug for COVID-19 induced pulmonary dysfunction.

- - - 1. Preclinical pharmacokinetics

The study of the metabolism and pharmacokinetics of the drug substance Treamide included the determination of drug stability in human liver microsomes, rat and human hepatocytes, artificial gastric juice, binding to rat and human plasma proteins, inhibition of human liver cytochromes, permeability and Pgp-substrate specificity in the intestinal cell model Caco-2 line, study of pharmacokinetic parameters, excretion and distribution after a single intragastric administration to rats and accumulation after repeated intragastric administration to rats. The listed preclinical studies of metabolism and pharmacokinetics have shown that the drug is practically not metabolized, has low bioavailability, but, nevertheless, is significantly distributed in such animal organs as the testes, prostate gland and bone marrow.

A comparative study of the pharmacokinetics of Treamide when administered orally to dogs in 15 mg of the pharmaceutical substance and the finished dosage form (3 tablets of 5 mg) did not show statistically significant differences in the parameters T _max_, C _max_, AUC _0-t_ and AUC _0-inf_, T _1 / 2_, MRT _0-inf_.

- - - 1. Toxicology

A study of the acute toxicity of the pharmaceutical substance of Treamide in mice showed that the degree of intoxication is dose-dependent. For male and female mice, LD50 when administered intragastrically is more than 5500 mg/kg; LD50 for male mice with intraperitoneal injection is 1074±167 mg/kg; for females - 1000±167 mg/kg. Thus, the active substance of Treamide is a moderately toxic compound and belongs to the III class of toxicity in accordance with GOST 12.1.007-76.

A study of the acute toxicity of the pharmaceutical substance of Treamide in rats showed the absence of toxic effects of the substance when administered intragastrically (LD50 more than 5500 mg/kg). The degree of intoxication with intraperitoneal administration is dose-dependent. LD50 for females is 1324±199 mg/kg, for male rats - 1173±169 mg/kg. Thus, in accordance with GOST 12.1.007-76, the active substance of Treamide is a low-toxic compound and belongs to the IV class of toxicity.

A study of the chronic toxicity of Treamide in rats (3 months) showed that with intragastric administration of therapeutic doses (TD) - 5 mg/kg, as well as 5TD (25 mg/kg) and 10TD (50 mg/kg) for 3 months, the drug Treamide does not have any toxic effect on the functional state of organs and body systems of experimental animals.

The study of chronic toxicity of GLF preparation Treamide (5 mg tablets and 50 mg tablets) with intragastric administration was carried out in male rabbits (1 month, 3 months) and female rabbits (3 months). The results showed that the drug Treamide in TD for 4 weeks does not have a toxic effect on the organs and systems of male rabbits, with the exception of a slight increase in the level of glucose and cholesterol in the blood, as well as an increase in the weight gain of rabbits in the process of animal growth ; within 3 months does not have a pronounced toxic effect on the functional state of the organs and systems of the body of male rabbits and female rabbits.

A study of the chronic toxicity of GLF of the drug Treamide when administered orally in Beagle dogs for 6 months at doses of 1.6 mg/kg (TD) and 16 mg/kg (10TD) to males and 16 mg/kg (10TD) to females showed that Treamide in TD and 10TD had no toxic effects on the organs and systems of male and female dogs.

The study of the reproductive toxicity of the Treamide drug substance in rats included: the study of the effect on reproductive (generative) function, the study of embryo- and fetotoxic effects recorded in the antenatal period of development and the study of antenatal effects recorded in the postnatal period of development. The data obtained allow us to conclude that the substance of the preparation Treamide intragastrically in TD (4.3 mg/kg) and 10TD (43.0 mg/kg) does not have reproductive toxicity.

The study of the effect of Treamide intragastrically at 3 doses (0.5, 5 and 50 mg/kg) on the generative function of male rats included an assessment of the fertility index and the pregnancy index when mating male rats of experimental groups with intact females for 10 days. The results allowed us to conclude that the administration of Treamide at the stage of progenesis (the duration of the entire cycle of spermatogenesis) at doses of 0.5, 5, and 50 mg/kg does not have a toxic effect on the reproductive system of male rats.

The study of the allergenicity of the pharmaceutical substance Treamide in TD and 10TD intragastrically once a day for 14 days was performed on male and female guinea pigs, Balb/c mice, CBA/CaLac mice and outbred mice in the following temps: 1) reaction general anaphylaxis (anaphylactic shock) in guinea pigs; 2) active cutaneous anaphylaxis in mice; 3) indirect reaction of degranulation of mouse mast cells; 4) "delayed" type hypersensitivity reaction (DTH) in mice; 5) inflammatory response to concanavalin A; 6) conjunctival test on guinea pigs; 7) method of skin applications on guinea pigs. The drug Treamide in the studied dosages and under the selected experimental conditions did not have allergenic properties.

The mutagenic activity of the pharmaceutical substance of Treamide was studied in the *Salmonella* /microsome test (Ames test) and in the cytogenetic test for accounting for chromosomal aberrations in mouse bone marrow cells. The results of the study of the drug Treamide in the Ames test showed that it does not induce gene mutations on *Salmonella typhimurium* strains TA 97, TA 98 and TA 100 at doses of 0.5-5000 μg / cup in conditions: without and in the presence of a metabolic activation system. In a cytogenetic test for accounting for chromosomal aberrations in mouse bone marrow cells, it was shown that a single and 5-fold intragastric administration of Treamide to mice in TD (0.71 mg/kg), as well as a single intragastric administration at a dose of 1/5 of LD50 ( 1000 mg/kg) does not induce chromosomal aberrations in animal bone marrow cells. Thus, the active substance of Treamide does not have a mutagenic effect.

Evaluation of the carcinogenic activity of Treamide drug substance was carried out on the basis of the results of a battery of short-term screening tests, including the study of the mutagenic activity of Treamide drug and the study of its ability to induce DNA damage in bone marrow, liver, kidney and spleen cells of mice (DNA comet test). The study showed that Treamide does not induce DNA damage in the cells of the bone marrow, liver, kidneys and spleen of mice. Thus, Treamide does not have carcinogenic properties.

The study of the immunotoxicity of the pharmaceutical substance of Treamide included an assessment of the immunotropic potential after a single and 14-day intragastric administration. The study showed that Treamide does not have an immunotoxic effect.

- - 1. **Clinical researches**

Investigational drug Treamide was studied in humans in one phase I clinical trial (METS-TRE-01; approval of the Ministry of Health of the Russian Federation for conducting clinical trial No. 52 of January 25, 2016) in healthy volunteers (single and multiple doses).

The aim of this study was to study the safety profile, pharmacokinetics and tolerability of Treamide in single and multiple doses in healthy volunteers. The study had a double-blind design. The study included 3 consecutive cohorts of volunteers who received the study drug at a dose of 5 mg, 15 mg and 50 mg or placebo. The 5 mg and 15 mg cohorts included 5 volunteers each, and the 50 mg cohort included 10 volunteers. Treamide/placebo ratio in the 5 mg and 15 mg cohorts was 4:1, in the 50 mg cohort it was 8:2. Thus, 20 volunteers took part in the study, of which 4 volunteers took Treamide at doses of 5 mg, 15 mg and placebo, 8 volunteers took Treamide at a dose of 50 mg.

The study consistently examined the safety, tolerability and pharmacokinetics of Treamid. Volunteers received study drug once at a cohort dose and then, after a 6-day break, continued daily study drug at the same dose for 14 days.

Inclusion of volunteers in subsequent dose cohorts was possible only after a positive decision of the safety data monitoring committee.

Twenty safety population volunteers completed the study according to protocol, with no early dropouts. AEs were reported in volunteers from all dose cohorts and were not reported in volunteers who received placebo.

In general, Treamide showed a favorable safety profile and good tolerability in the study. No SAEs were noted during all phases of the study, **no AEs were related to study medication and did not require treatment, and all AEs resolved**. Four AEs rated as CTCAE 4.03 Grade 3 or 4 were reported in volunteers in the lowest laboratory dose cohort of 5 mg and were asymptomatic.

During the study, 13 AEs were registered in 7/16 (43.8%) volunteers who received Treamid:

- at a dose of 5 mg in 3/4 (75.0%) volunteers, 6 AEs were registered (1 AE "Chickenpox" of moderate severity, 2 AEs "Increased CPK levels" of severe severity, 2 AEs "Hyperkalemia" of moderate and severe severity, 1 AE "Hypernatremia" life-threatening severity);
- at a dose of 15 mg in 2/4 (50.0%) of volunteers, 5 AEs were registered (2 AEs "Indigestion" of mild severity, 1 AE "Discomfort in the epigastric region" of mild severity, 1 AE "Nausea" of mild severity and 1 AE " Increasing the level of creatine phosphokinase in the blood "of moderate severity;
- at a dose of 50 mg in 2/8 (25.0%) volunteers, 2 AEs were registered (1 AE "Hyperbilirubinemia" of moderate degree and 1 AE "Increased level of creatine phosphokinase in the blood" of moderate degree.

Significant differences in the main PK parameters with single and multiple dosing of Treamide were not observed. With an increase in the dose of Treamide from 5 to 15 mg, stationary C _max_ and AUC _0-t_ increase proportionally, however, at a dose of 50 mg, a deviation from linearity is observed: the values of C max and AUC _0-t_ are lower than expected. With a single dose of the drug, the linearity of the pharmacokinetics of the drug Treamide is apparent, due, obviously, to an underestimated value of the average C _max_ and AUC _0-t_ in the 15 mg cohort on Day 1 due to subjects 204 and 205.

The values of dose-normalized Cmax and AUC _0-t_ do not remain constant over the entire interval of the studied dose range - they decrease at a dose of 50 mg.

There is no pronounced dose dependence for _Tmax_. With a single dose, T _max_ averaged 1.3-2.5 hours, with multiple doses - 1.0 - 1.75 hours. For T1 / 2, there is a tendency to increase with increasing dose from 5 to 50 mg. With a single dose, T1 / 2 increased from 4.6 in the 5 mg cohort to 11.6 hours in the 50 mg cohort, with multiple doses, from 4.3 hours to 12.1 hours, respectively.

The cumulation factor (Fc) in the studied dose range of 5-50 mg is 1.15-1.17. Thus, with multiple doses of the drug once a day, the total exposure of the active substance of the drug Treamide in blood plasma increases by 20% compared with a single dose.

Thus, the data obtained during the clinical trial allow us to conclude that Treamide in daily doses of 5 mg, 15 mg and 50 mg has a favorable safety profile, good tolerability and can be recommended for further clinical trials.

- - 1. **Risk/benefit ratio**

This study is aimed at obtaining data on the efficacy and safety of Treamide when used in patients at risk of developing fibrotic changes in the lungs after suffering COVID-19 pneumonia.

Treamide suppresses notcW1-mediated proliferation of progenitor cells, which leads to a decrease in the production of key profibrous cytokines (TGF-beta, IL-13), a decrease in the severity of the inflammatory response and suppression of lung tissue destruction at the initial stage of pathology development. Also, inhibition of notcW1-mediated proliferation under the action of Treamide leads to suppression of the synthesis of extracellular matrix components (fibronectin and type I collagen) and the deposition of fibrous masses in lung tissues. In addition, activation of the differentiation of progenitor cells leads to an increase in the number of mature epithelial and endothelial cells in the lung tissue and accelerates the processes of lung regeneration.

**Thus, Treamide is expected to be effective in patients at risk of developing fibrotic changes in the lungs after suffering from COVID-19 pneumonia and will provide an earlier and / or complete restoration of respiratory function and diffusive capacity of the lungs, which in turn will lead to a decrease or elimination of shortness of breath, including during physical exertion**.

In the course of preclinical studies of general and specific toxicity for the most sensitive animal species - rabbits, the minimum dose without an observed negative effect (DBNOE) was determined - 23 mg / kg. In this case, the equivalent dose for a person with a body weight of 70 kg (EDH) is 519 mg.

In the conducted preclinical studies, Treamide did not have a toxic effect on the functional state of the organs and systems of the body of experimental animals; did not have a local irritating and allergenic effect; did not have mutagenic, carcinogenic and immunotoxic effects; did not have a toxic effect on reproductive function.

In a Phase I clinical trial, Treamide generally demonstrated a favorable safety profile and good tolerability. There were no SAEs during the study, no AEs were related to study medication or required treatment, and all AEs resolved. Most AEs were mild to moderate in severity; The 4 AEs, the severity of which corresponded to grade 3 or 4 according to CTCAE 4.03, were asymptomatic and did not require discontinuation of the drug or treatment.

Based on the AEs reported in the Phase I clinical trial, changes in laboratory parameters (including CPK, bilirubin, and electrolytes) will also be monitored in this trial.

Prescribing Placebo to patients in this study can be considered justified, since at present no specific pharmacotherapy for patients after COVID-19 pneumonia has been described, including as part of pulmonary rehabilitation [16].

Only those patients who agree to use adequate methods of contraception during the entire study period and for 3 months after its completion will be included in this study.

Thus, the overall risk/benefit ratio appears to be favorable for participants in this clinical trial.

1. POPULATION SELECTION
   1. **Study population**

The study is planned to include 60 patients at risk of developing fibrotic changes in the lungs after suffering COVID-19 pneumonia.

To be included in this clinical trial, patients must meet the following inclusion/exclusion criteria.

- 1. **Inclusion Criteria**

To participate in the study, patients must meet the following criteria:

1. Signed Patient Information Sheet and informed consent form for participation in the study.
2. Men and women aged 18 to 75 inclusive.
3. The risk of developing fibrotic changes in the lungs after suffering COVID-19 pneumonia:
4. The diagnosis of COVID-19 in history was confirmed by a positive qualitative analysis of SARS-CoV-2 RNA by PCR;
5. The appearance of the first symptoms of COVID-19 no more than 2 months before screening;
6. The presence of foci of lung tissue compaction of the "ground glass" type and / or seals of the interlobular septa, and / or areas of reticular changes in the lung tissue, and / or the presence of areas (linear, focal) of lung tissue seals characteristic of COVID-19, confirmed according to the data CT scan of the chest for screening.
7. Negative test for COVID-19 at screening (confirmed).
8. Severity 2 (moderate) or 3 (severe) on the mMRC Breathlessness Scale at screening and randomization.
9. Decreased lung function FVC and/or DLCO < 80% predicted at screening.
10. Consent of patients to use adequate methods of contraception during the entire study and within 3 months after its completion. Adequate methods of contraception include the use of:

- oral or transdermal contraceptives;
- condom or diaphragm (barrier method) with spermicide;
- intrauterine device.
  1. **Non-inclusion сriteria:**

A patient will be considered ineligible for the study if one of the following criteria is met:

1. Women who are pregnant or breastfeeding or planning to become pregnant during a clinical study; women of childbearing potential (including those who are not surgically sterilized and who are less than 2 years postmenopausal) who do not use adequate contraceptive methods.
2. The use of invasive mechanical ventilation (iALV), plasma transfusion (including convalescent plasma) and other blood components during COVID-19 therapy.
3. History of chronic respiratory disease, including idiopathic pulmonary fibrosis (IPF), asthma, chronic obstructive pulmonary disease (COPD), or pulmonary hypertension, diagnosed prior to COVID-19.
4. Severe cardiovascular disease at present or within 6 months prior to screening, including: NYHA class III or IV chronic heart failure, clinically significant ventricular arrhythmias (ventricular tachycardia, ventricular fibrillation), unstable angina pectoris, myocardial infarction, heart and coronary surgery, significant valvular heart disease, uncontrolled arterial hypertension with systolic blood pressure > 180 mm Hg. and diastolic blood pressure > 110 mmHg, pulmonary embolism (PE) or deep vein thrombosis.
5. Nephrotic syndrome, moderate to severe chronic renal failure, or significant kidney disease with creatinine > 1.5 mg/dL (132 µmol/L) or GFR < 60 mL/min at screening.
6. Cirrhosis of the liver in history; an increase in alanine aminotransferase (ALT) and / or aspartate aminotransferase (ACT) by 3 or more times from the upper limit of normal (ULN) at screening; an increase in the level of total bilirubin by 2 or more times from ULN at screening.
7. Hemoglobin level < 90 g/l at screening.
8. Severe diseases of the central nervous system, including a history of seizures or conditions that can lead to their development; stroke or transient ischemic attack within 6 months prior to screening; traumatic brain injury or loss of consciousness within 6 months prior to screening; a brain tumor.
9. Signs of significant uncontrolled comorbidity, such as disorders of the nervous system, kidneys, liver, endocrine system and gastrointestinal tract, which, in the opinion of the Investigator, could prevent the patient from participating in the study.
10. Malignant neoplasms requiring chemotherapy within 6 months prior to screening.
11. History of HIV infection.
12. Prostate cancer or benign prostatic hyperplasia (BPH) with a residual urine volume of more than 100 ml in history in men.
13. Hypersensitivity or intolerance to any of the components of the study drug.
14. Participation in other clinical trials within 2 months prior to screening.
15. Taking the following drugs: bronchodilators, anticholinergics, corticosteroids, cytostatics, colchicine, cyclosporine A, interferon-γ-1b, bosentan, macitentan, etanercept, sildenafil, imatinib, n-acetylcysteine, warfarin, ambrisentan, nintedanib, pirfenidone 1 month before screening [17].
16. inability to read or write; unwillingness to understand and follow study protocol procedures; non-compliance with drug regimens or procedures that, in the opinion of the Investigator, may affect the results of the study or patient safety and prevent the patient from continuing to participate in the study; any other medical or serious psychiatric condition that makes the patient unsuitable for participation in the clinical study, limits the eligibility of obtaining informed consent, or may affect the patient's ability to participate in the study.
    1. **Exclusion of patients from the study after randomization**

If the patient's condition deteriorates due to increased respiratory symptoms, decreased lung function, or episodes of acute respiratory deterioration, the patient will be removed from the study and, if necessary, admitted to a hospital for medical care. Criteria for early withdrawal of patients from the study are presented in Section 6.15.

Pulmonary fibrosis progression is defined by the occurrence of any of the following criteria [17]:

- relative reduction in FVC by ≥ 10%;
- a relative reduction in FVC ranging from ≥ 5% to < 10% and a relative reduction in DLCO by > 15%.

1. STUDY DRUG
   1. Description of study drug

| Investigated drugs: | Treamide or placebo | | |
| --- | --- | --- | --- |
| Drug composition | *Active ingredient:* Treamide (ХС268БГ) - 50 mg. Placebo - 0 mg. | |  |
|  |  | *Excipients:* |  |
|  |  | cellulose microcrystalline |  |
|  |  | pregelatinized starch |  |
|  |  | Sodium starch glycolate |  |
|  |  | Talc |  |
|  |  | Magnesium stearate |  |
|  |  | **Colloidal silicon dioxide** |  |
|  |  | *Shell:*  Opadry II white [hypromellose 34.0%, lactose monohydrate 28.0%, titanium dioxide 26.0%, macrogol 12.0%] |  |
| Pharmaceutical form | Film-coated tablets | | |
| Appearance: | Tablets are round, biconvex, film-coated white. Tablet core white or almost white | | |
| **Packaging.** | Blister Alu/Alu, №10 | | |
| Method of administration: | Orally, once a day, in the morning, 30 minutes before breakfast | | |
| Manufacturer: | IIHR JSC, Russia, OKHPC CJSC, Russia (by order of PHARMENTERPRICES LLC, Russia) | | |
| Storage conditions: | In a place protected from light, at a temperature not exceeding 25 ° C. Keep out of the reach of children | | |

- 1. **Study drug intake**

Study drug will begin at Visit 2, Week 0. Patients will receive either Treamide or Placebo for 4 weeks. Study therapy will be blinded by placebo masking. Patients will receive individual packs of study medication containing Treamide 50 mg or placebo.

Patients will be instructed to take study drug 1 tablet once daily in the morning 30 minutes before meals.

Patients will receive their first dose of study drug at the study site. Subsequent admission will be carried out independently. On Visit Day 4, the drug will be taken after blood sampling for general clinical and biochemical tests. Last dose of study drug on the day before Visit 6, Week 4.

- 1. **Method of allocation of patients to treatment groups**

Will be randomized into two groups in a 1:1 ratio using the IWRS system. Each group will eventually include 30 patients. Randomization will be stratified by

1. presence or absence of risk factors (age ≥ 60 years and/or presence of concomitant chronic diseases)
2. and the initial degree of lung damage according to CT data (CT 1, CT 2, CT 3 and CT 4)

The stratification method will be described in more detail in the randomization protocol.

After randomization, patients will receive the required amount of study drug until the next visit, according to the treatment group. Instructions for randomization and operation of the IWRS system will be provided to the investigator prior to the start of the study.

- 1. **Blinding of study drug**

This study is double-blind. Clinical center staff and patients will not know which therapy is prescribed for each individual patient. Blinding will be provided by placebo masking (each patient will take tablets corresponding to Treamide 50 mg or placebo) and dispensing the drug by IWRS by individual package number.

- 1. **dazzle**

The code for a specific patient can only be opened in an emergency if knowledge of the patient's specific therapy could affect his treatment. To open the code, there will be a special procedure for requesting the code through IWRS. The reason for opening the code should be described in detail in the patient's primary documentation. The need to blind the therapy of a particular patient should, if possible, be discussed with the Medical Monitor in advance or notify him no later than 24 hours after opening the code.

- 1. **Compliance with the therapy**

The first dose of study drug must be taken by the patient at the study site under the supervision of the Investigator (Visit 2, Week 0). Compliance with the recommendations for self-administration of investigational drug will be verified by the Investigator at visits based on the results of accounting for the returned investigational drug.

Compliance calculation will be carried out on visits according to the formula:

Compliance = (N issued - N returned) / N calculated x 100%

where N is issued. = number of tablets dispensed, N return. = number of returned tablets, N calc. = Estimated number of tablets the patient should have taken since the previous visit.

Patient compliance should be between 80% and 120%. If necessary, an additional conversation should be held with the patient about the correct administration of the drug. In the event of repeated violation of the study drug regimen and/or failure to follow the Investigator's recommendations, the patient must be early withdrawn from the study as agreed with the Sponsor or its representative.

- 1. **Concomitant Therapy**

Concomitant therapy for acute or chronic conditions may be continued as indicated. All concomitant drugs and biologically active additives (BAA) must be registered in the primary documentation and in the eCRF.

The name (preferably the name of the active substance), dosage, frequency of administration, route of administration, indication for use (including the underlying disease, concomitant condition, adverse event or prophylaxis), the start and end date of the concomitant drug should be recorded in the primary documentation and in eCRF. The study should reflect any changes in concomitant therapy. If concomitant therapy is ongoing at the end of the study, this should be noted in the eCRF.

- 1. **Prohibited Therapy**

Throughout the study, patients **should not** receive other study drugs, as well as drugs from the following groups: bronchodilators, anticholinergics, glucocorticosteroids, cytostatics, colchicine, cyclosporine A, interferon-γ-1b, bosentan, macitentan, etanercept, sildenafil, imatinib, n -acetylcysteine, warfarin, ambrisentan, nintedanib, pirfenidone [17].

- 1. **Packaging and labeling of investigational product and concomitant therapy**

The study drug will be provided by the Sponsor. The study drug will be packaged in aluminum blisters and labeled in accordance with applicable law and applicable regulations.

- 1. **Storage and record keeping of the study drug**

The investigator will be responsible for arranging the storage of the study product at the study site throughout the study. Limited access conditions and appropriate temperature conditions must be provided. Temperature control should be carried out using thermometers that record the minimum and maximum temperatures for the reporting period. Temperature monitoring data should be recorded regularly in a temperature log. At the end of the study, after the final count has been made, the study drug must be returned to the Sponsor or its representative or destroyed at the center at the discretion of the Sponsor.

The investigator will be responsible for maintaining investigational product records to ensure they are properly received, stored, distributed and returned.

1. **DESCRIPTION OF PROCEDURES**

All research procedures will be carried out in accordance with the regulatory requirements and recommendations of the Good Clinical Practice Guidelines of the International Conference on Harmonization (ICH GCP) and the Eurasian Economic Union (EAEU), the principles set forth in the Declaration of Helsinki, as well as in accordance with current legislation and applicable regulatory requirements of the Russian Federation. The Investigator must agree to conduct monitoring, audits and inspections in the research center and at any time, upon request, provide direct access to the study materials to the Sponsor and its representatives, the Independent Ethics Committee and authorized bodies.

- 1. **Informed consent**

Informed consent will be obtained at screening prior to any study-related procedures. If screening procedures are carried out as part of a patient’s discharge from the hospital after treatment for COVID-19 pneumonia, it is necessary to provide for the procedure and signing of the informed consent form in a “clean zone” to ensure the safety of primary documentation and prevent its contamination.

- 1. **Patient Registration**

Each patient who signs the Patient Information Sheet and the informed consent form will be assigned an individual registration number consisting of four digits 0-0 O: the first two digits correspond to the center number, the second two digits - the patient's serial screening number in this center (01, 02, 03 etc.). Instructions for registering patients will be provided to the Investigator prior to the commencement of the study.

- 1. **Demographics and medical history**

To assess whether patients meet inclusion/non-inclusion criteria, patient demographics (gender, date of birth/age, race) and a complete medical and epidemiological history, duration of disease symptoms, significant acute and chronic diseases and conditions (e.g. menopause), surgical interventions, allergic reactions and smoking status. All new diagnoses and conditions identified at the screening (including the results of laboratory and instrumental studies) should be attributed to the patient's medical history.

- 1. **Physical examination**

Physical examination includes evaluation of general appearance, condition of the skin and mucous membranes, eyes, ears, nose and throat, as well as an examination of the cardiovascular, musculoskeletal, respiratory system, gastrointestinal tract and nervous system.

Clinically significant changes in the physical examination compared to baseline should be considered as AEs and recorded appropriately in the primary documentation and in the eRCI.

- 1. Vital signs and measurement of blood oxygen saturation (SpO2)

This examination includes the measurement of axial body temperature, as well as blood pressure (BP), pulse, respiratory rate and blood oxygen saturation (SpO2) in a sitting position after 10 minutes of rest.

Clinically significant changes in vital signs from baseline should be regarded as AEs and recorded appropriately in the primary documentation and in the eRCI.

- 1. Electrocardiography

Electrocardiography (ECG) in 12 leads is performed with the patient in the supine position after 10 minutes of rest. The electrodes should be placed on the same points throughout the study. To record an ECG, you must use an electrocardiograph with automatic interval assessment. The following intervals will be assessed on the ECG: HR, PQ, QRS, QT, QTc(B).

Parameter QTc( B ) will be calculated automatically in eCRF according to the formula:

Bazett's formula: QTc (B) = QT / √RR

The RR parameter for calculating QTc( B ) will also be calculated automatically in eCRF using the formula:

|  | *RR* | = | 60 |
| --- | --- | --- | --- |
|  |  |  | HR |

The Investigator must review the ECG record, in case of deviations, evaluate their clinical significance, sign and date the conclusion. Significant changes on the ECG should be rechecked, repeated studies should be carried out.

Clinically significant changes in the ECG compared to baseline should be regarded as AEs and recorded accordingly in the primary documentation and in the eRCI.

- 1. CT scan of the chest

Computed tomography (CT) of the chest will be used to assess lung involvement. The procedure will be performed at each center according to standard CT protocol. The assessment will be performed by a qualified radiologist at the center.

The assessment will be carried out in accordance with the classification according to the degree of changes detected.

| **Computed tomography of the chest** | | | | |
| --- | --- | --- | --- | --- |
| **The main manifestations of viral pneumonia** | **Frosted glass** | **Consolidation** | **Other additional features** | **Involvement of the lung parenchyma** |
| **CT 0**  The norm and absence of CT signs of viral pneumonia against the background of a typical clinical picture and a relevant epidemiological history | No | No | No | No |
| **CT1**  Areas of ground-glass compaction Involvement of the lung parenchyma < 25%  Or the absence of CT features against the background of a typical clinical picture and a relevant epidemiological history | Yes | no, single small size | Single zones of small size of reticular changes | < 25% |
| **CT2**  Ground glass seal zones  Involvement of the lung parenchyma 25-50% | Yes | Yes  single | Single zones of small reticular changes, there may be a reverse "halo" | < 50% |
| **CT3**  Ground glass seal zones  Consolidation zones  Involvement of the lung parenchyma 50-75%  An increase in the volume of the lesion up to 50% in 2448 hours against the background of respiratory disorders, if the studies are performed in dynamics | Yes | yes, possibly massive | Areas of reticular changes, may be reversible "halo" May be air bronchogram symptom Minimal hydrothorax not associated with pneumonia | 50-75% |
| **CT 4**  Diffuse ground-glass thickening of the lung tissue and consolidation in combination with reticular changes  Hydrothorax (bilateral, predominant on the left)  Parenchymal involvement lung ≥ 75% | Yes | Yes, dominated by massive | Zones of reticular changes, there may be a reverse “halo”, Symptom of an air bronchogram Hydrothorax predominantly on the left | ≥ 75% |

The imaging records, description, and CT report should be evaluated by the Investigator. Changes to the CT must be properly recorded in the primary documentation and in the eCRF.

- 1. Analyzes performed in the laboratory

All laboratory studies provided for in the protocol will be performed in the local laboratory of the research centers.

Before the start of the study, the local laboratory of each research center will be required to provide the required certificates and laboratory standards. Upon receipt of a laboratory report, the Investigator will be required to review it, if deviations of indicators from normal values, assess their clinical significance, sign and date the report. Clinically significant changes in laboratory values from baseline should be considered an AE (eg, if laboratory abnormalities result in medical intervention, treatment delay, treatment interruption, hospitalization, or complications).

- - 1. **Laboratory parameters**

Necessary laboratory tests will be carried out at certain visits in accordance with the Procedure Schedule. Blood samples for analysis will be taken on an empty stomach in accordance with the center's regulatory procedures.

The table below (Table 2) presents the blood and urine laboratory values that will be assessed in this clinical trial.

Table 2. List of indicators of laboratory tests of blood and urine

| **- Complete Blood Count** | **Blood chemistry;** | | | **Special Studies** | **General urine analysis** |
| --- | --- | --- | --- | --- | --- |
|  | General | Hepatic status | Kidney status |  |  |
| Hemoglobin  Hematocrit  Red blood cells  White blood cells  Neutrophils  Lymphocytes  Monocytes  Eosinophils  Basophils  Platelets  ESR | Glucose  Total protein  Sodium  Potassium  Chlorine  CPK | Total bilirubin  Alkaline phosphatase  ALT  AST  GGT | Blood urea nitrogen  Serum creatinine  GFR (according to the Cockcroft-Gault formula) | Qualitative determination of SARS-CoV-2 RNA | General properties: color, transparency, specific gravity, pH, protein, glucose, bilirubin, urobilinogen, ketone bodies, nitrites, hemoglobin  Sediment microscopy: epithelium, erythrocytes, leukocytes, casts, bacteria, salts |

*rapid test or enzyme immunoassay

Abbreviations: ESR - erythrocyte sedimentation rate, CPK - creatine phosphokinase, ALT - alanine aminotransferase, AST - aspartate aminotransferase, GGT - gamma-glutamyl transferase, GFR - glomerular filtration rate

A pregnancy test is only given to women of childbearing potential (including women less than two years of menopause). The test is performed directly at the center using a test strip in a urine sample.

- - 1. **PCR for SARS-CoV-2 RNA**

Qualitative determination of SARS-CoV-2 RNA will be carried out by PCR in the local laboratory of the center, or will be sent by the centers to laboratories accredited by Rospotrebnadzor in accordance with the routine practice of the center. The material for the study will be a swab from the nasopharynx and / or oropharynx according to the standard technique.

A swab from the nasopharynx or oropharynx (pharynx) is taken with a sterile swab, which, after taking the material, is placed in a sterile plastic tube with a transport medium (taking into account the manufacturer's recommendations, the test systems / reagent kits used).

Confirmation of two consecutive negative PCR results at least 24 hours apart is required for inclusion in the study. In the case of previously confirmed elimination of the SARS-CoV-2 virus according to medical records, one determination of SARS-CoV-2 RNA by PCR with a negative result within the study will be sufficient.

- - 1. **Pharmacokinetic study**

All patients will have a blood sample taken on the morning of Week 0, Week 2 and Week 4 before their next dose of study drug to determine the concentration of the active substance XC268BG and calculate the PK parameter Residual Ct _rough_.

Receipt, sample preparation, storage and shipment of samples for PK testing will be carried out in accordance with regulatory requirements and laboratory guidelines.

To evaluate pharmacokinetic parameters, HPLC/MS/MS will be used using a QTRAP 5500 system (Applied Biosystems) with an Agilent 1290 chromatograph (Agilent Technologies).

Investigational therapy blinding will be performed prior to conducting the PK study and after closing the database. PK analysis for patients receiving placebo will only be performed if there is doubt about the correctness of the blinding procedure. To confirm the absence of the study drug in the blood plasma of patients treated with placebo, analysis can be performed at points corresponding to the calculated values of T _max_.

- - 1. **Receipt, preparation, storage and dispatch of biosamples**

Receipt, preparation, storage and shipment of biosamples will be carried out in accordance with regulatory requirements and laboratory guidelines.

The total volume of blood collected from each patient during scheduled study visits will be approximately 102 ml.

Patients who will have additional PK sampling at Week 0 will have approximately 102 mL of blood collected during their scheduled study visits.

| **Blood volume for various research procedures, ml** | Screening | **Investigational Therapy** | | | | | **Follow-up** |  |
| --- | --- | --- | --- | --- | --- | --- | --- | --- |
| Visit (V) | V 1 | V 2 | V 3 | V 4 | V 5 | V 6 | V 7 |  |
| Week (W) | W-2 | W0 | W1 | W2 | W3 | W4 (ET) | W6 | Total, ml: |
| - Complete Blood Count | 6 | 6 |  | 6 |  | 6 | 6 |  |
| Blood chemistry; | 12 | 12 |  | 12 |  | 12 | 12 |  |
| PK study (all patients) |  | 4 |  | 4 |  | 4 |  | 102 |

- 1. **mMRC (modified Medical Research Council) breathlessness scale**

The severity of breathlessness will be assessed using the mMRC Breathlessness Scale (Appendix 1). The patient must choose one of the five responses presented on the scale, characterizing the shortness of breath that he is currently experiencing. Scores on the scale characterize the severity of dyspnea from 0 (no dyspnea) to 4 (very severe dyspnea). Each point on the scale is accompanied by explanations that allow the patient to make the most accurate choice.

The examiner must review the scale completed by the patient and sign and date the form. Clinically significant changes in the severity of dyspnea from baseline should be regarded as AEs and recorded appropriately in the primary documentation and in the eRCI.

- 1. **6 Minute Walk Test (6MWD, Borg Scale)**

The 6-minute walking test is based on measuring the walking distance (6-minute walking distance - 6MWD) with turns along a long straight corridor (≥30 m), at the patient's own pace. Allows you to evaluate submaximal exercise tolerance, which corresponds to the ability to perform daily work.

To assess dyspnea on exertion, the Borg Scale is used, which is assessed by the patient immediately before and after the end of the test. This is a 12-point ordinal scale (from 0 to 10 with an intermediate value of 0.5). Before starting 6MWD, you need to show the scale to the patient and ask him to determine the degree of respiratory discomfort and general fatigue on it. At the end of testing, the patient should be reminded of their initial scores and asked to re-evaluate these symptoms.

Detailed instructions for the 6 Minute Walk Test and a sample Borg Scale are provided in Appendix 2.

The test should be stopped immediately if the patient develops: chest pain, severe shortness of breath, spasm of the muscles of the lower extremities, imbalance (stability), profuse sweating, sudden pallor, or a decrease in hemoglobin oxygen saturation (when using a pulse oximeter).

Clinically significant changes in the patient's condition during the test should be regarded as AEs and recorded accordingly in the primary documentation and in the eCRА.

- 1. **Questionnaire KBILD**

The King Interstitial Lung Disease Scoring Inventory (KBILD) is a short 15-item questionnaire. For each question, the patient must choose one of seven possible answers. A sample KBILD questionnaire is provided in Annex 3.

- 1. **Spirometry (FEV1, FVC, FEV1/FVC)**

Pulmonary function will be assessed using regularly calibrated spirometry equipment in accordance with the Russian Respiratory Society Federal Clinical Guidelines for the Use of Spirometry, 2013 [41]

Spirometry readings will include:

- Formed expiratory volume in 1 second (FEV1)
- Forced vital capacity (FVC)
- FEV_1_/FVC

Spirometry should be taken in the morning at about the same time during the study. In the case of an upper respiratory tract infection, a routine pulmonary function assessment should be rescheduled and performed no earlier than 7 days after resolution of symptoms.

Lung function estimates should use height measured at screening and body weight measured directly at the visit.

Tests must meet quality criteria for technical acceptability and reproducibility. To obtain reproducible results, at least three technically satisfactory maneuvers must be obtained that meet the acceptance criteria; the best (greatest) result must be registered in the CRF.

The investigator must review the spirometry report, evaluate the test results, and sign and date the report. Changes in spirometry values from baseline, regarded as AEs, should be recorded appropriately in the primary documentation and in the CRF.

- 1. **Body plethysmography (DLCO, TLC, FRC)**

Evaluation of lung function using a body plethysmograph in accordance with the guide "Functional diagnostics in pulmonology" ed. Z.R. Aisanova, A.V. Chernyaka, 2016 [42].

Body plethysmography allows you to fully determine all respiratory volumes, including those that cannot be obtained with spirography. The procedure is carried out using a body plethysmograph, which includes a body camera (where the patient sits) with a pneumotograph and a computer that displays data.

In this study, total lung capacity (TLC) and functional residual lung capacity (FRC) will be assessed. As part of the body plethysmography procedure, the lung diffusing capacity for carbon monoxide (DLCO) will also be assessed.

Tests must meet quality criteria for technical acceptability and reproducibility. To assess the DLCO parameter, a hemoglobin value is required, so the results of a complete blood count should be available at the time of the body plethysmography.

The Investigator will review the indicators, in case of deviations, evaluate their significance, sign and date the conclusion. Changes in DLCO, TLC, or FRC from baseline that are considered AEs should be recorded appropriately in primary documentation and in the eCRF.

- 1. **Assessment of adverse events (AEs)**
     1. **Definitions of AE**

**Adverse event (AE)** - Any adverse medical event identified in a clinical trial subject after use of a medicinal product, which may not have a causal relationship with its use. An AE can be any adverse symptom (including a laboratory abnormality), complaint, or disease.

In this study, AE registration will be carried out from the moment the patient signs the informed consent form and up to 30 days after the patient's last visit to the study center or the last protocol procedure. All AEs will be monitored during the investigational therapy phase; at the follow-up stage - only SAE.

**Adverse reaction (HP)** - all adverse reactions associated with the use of any dose of the study drug. In other words, if there is at least a minimal possibility of a causal relationship between the study drug and the AE, i.e. relationship is not excluded.

**Serious Adverse Event (SAE) and/or Serious Adverse Reaction (SAR)** - Any adverse medical event that, regardless of study drug dose:

- led to death;
- poses a threat to life;
- requires hospitalization or its extension;
- has resulted in permanent or significant disability or disability, or
- is a congenital anomaly or birth defect
- other important medical cases

Significant medical events that do not pose an immediate threat to life, do not lead to death or hospitalization, but put the patient at risk or require interventions aimed at preventing the above outcomes, can also be classified as SAE/SAR. Examples of such phenomena can be allergic bronchospasm, convulsions, malignant neoplasms.

**NOTE:** Hospitalization for social reasons, visits to a day hospital, and hospitalization or surgery planned prior to enrollment in a study for treatment of a preexisting condition are not considered SAEs.

**Unexpected adverse events** - An adverse event whose nature or severity is not consistent with known product information (eg, an Investigator's Brochure for an unregistered investigational product, or a package insert for an approved medicinal product). This group also includes adverse events that are mentioned in the Investigator's Brochure as being characteristic of this drug class, or expected due to the pharmacological properties of the study drug, but not previously observed.

- - 1. **Pregnancy**

Pregnancy is not an AE, but is an event requiring urgent reporting to the Sponsor. In case of pregnancy of the patient or partner of the study patient, the course of pregnancy will be monitored up to 30 days after its resolution. Relevant information will be recorded in the primary documentation, in the eCRF, as well as in the pregnancy registration form. Reporting the pregnancy of a patient/partner of a study patient must be sent to the Sponsor within the same time frame as reporting an SAE.

Throughout the study and for 3 months after its completion, patients, including men and women of childbearing potential, must use adequate methods of contraception (oral or transdermal contraceptives; condom or diaphragm (barrier method) with spermicide; intrauterine device).

- - 1. **AE reporting**

During each visit and telephone contact, patients should report any AEs in response to open-ended, non-leading questions (eg, "How have you been since your last visit?"). For each patient-reported AE, the investigator should collect and record in the primary documentation and in the eCRF all relevant information, including diagnosis or symptoms, date of onset and end date, outcome, severity, presence of severity criteria, circumstances that may indicate a possible association investigational drug or concomitant therapy, underlying diseases or comorbid conditions, study procedures or other causes, investigational drug activity, drug therapy, medical interventions, laboratory and instrumental findings for AEs, and other circumstances that will help maximize fully describe the event.

An increase in the severity of an ongoing AE should be considered as a new AE. The date of the change in severity will be considered the start date of the new AE, and the previous day will be considered the end date of the original AE. The start date of the SAE is the day the severity criterion occurs. The previous condition, if applicable, should be reported as a non-serious AE.

If the AE is serious, the Investigator must also complete the SAE report form and send it to the Sponsor in a timely manner. New SAE information that becomes known at a later date must be registered and communicated to Sponsor in the same manner and within the same time frame. Also, the Sponsor must be provided with certified copies of the primary documents for this SAE (discharge summary, autopsy data, death certificate, etc.).

AEs should be observed and reported from the time the patient signed the informed consent until 30 days after the last visit or procedure related to the study. All AEs are monitored during the investigational therapy phase. During the follow-up phase, only SAEs are monitored. AEs and SAEs should be monitored until resolution or stabilization of the patient's condition.

The monitor is responsible for verifying that the eCRF data and SAE reporting forms are consistent with the primary documentation.

- - 1. **Severity assessment**

The severity of the AE will be assigned in accordance with the criteria presented in the table below (Table 3) (corresponding to the NCI-CTCAE classification). The use of the classifier itself for grading the severity of AE is not required.

Table 3 AE severity assessment

| **AE Severity** | | **Definition (meets one or more criteria)** |
| --- | --- | --- |
| Degree 1 | Mild | - Asymptomatic or mild symptoms - Only clinical or diagnostic observation is needed - Medical intervention not indicated |
| Degree 2 | Moderate | - Minimal, local or non-invasive intervention indicated - Limits age-appropriate important daily activities |
| Degree 3 | Severe /  Medically significant non-life threatening AE | - Hospitalization or its extension is indicated - AE leading to disability/disability - Limits the ability for daily self-care |
| Degree 4 | Life-threatening consequences | - Requires immediate medical attention |
| Degree 5 | Death associated with AE | - AE with fatal outcome |

- - 1. **Association of AEs with study drug**

For each AE, the Investigator should evaluate its possible association with the investigational product according to the categories presented below - Table 4.

Table 4 Assessing relation to the study drug use

| Category of AE | Definition |
| --- | --- |
| 1. Related | - occurs in a certain significant time interval after the use of the study drug; - accompanies a known reaction to the use of the investigational drug; - resolved after discontinuation of the drug and - reoccurs after restarting study drug (depending on the nature of the AE, repeat use may not be possible) |
| 2. Possibly related | - Occurs within a significant time interval after administration of the study drug - accompanies a known reaction to the use of the investigational drug or drugs of the same pharmacological group - could also be related to the patient's disease or condition, concomitant therapy or procedure |
| 3. Unlikely related | - there is sufficient information indicating that there is no causal relationship with the investigational product - possible other cause |
| 4. Not connected | - no temporal association with study drug (eg, AE occurring in a patient who did not receive study drug) - another cause is known |

- - 1. **Reporting SAEs/Pregnancy**

All SAEs, regardless of severity and association with study drug, as well as pregnancy of the patient/partner of the study patient, must be reported to the Sponsor or his representative within 24 hours from the moment the Investigator receives information about their occurrence. SAEs must be entered into the eCHR in the SAE reporting section of the form. Pregnancy data must also be entered in the eCRF in the Pregnancy Reporting section of the form. If necessary, the Investigator may contact the Medical Monitor for advice or clarification.

Reporting SAEs/Pregnancy

Company: LLC "IFARMA"

Medical Monitor: Kasyanova O.V.

Phone: +7 (495) 276-11-43

Mobile: +7 (925) 421-52-23

*Fax +7(495) 276-11-47*

Email e-mail: SAE@ipharma.ru

The Investigator must instruct each patient to promptly report the occurrence of an SAE or pregnancy during the study and within 30 days of the last study site visit or last study procedure. The investigator should record on the SAE/pregnancy report form all available information about the event. Each SAE reporting form should include, at a minimum, the following data: patient information, event name, severity criteria, association with investigational drug, and outcome at the time of reporting.

- - 1. **Withdrawal of patients from the study**

The patient must be withdrawn from the study if deemed medically necessary by the Investigator or if the patient has withdrawn consent (reasons for early withdrawal are described in Section 6.15). If possible, the Investigator should first discuss the exclusion of the patient with the Medical Monitor. Otherwise, the investigator must discuss the patient's exclusion with the Medical Monitor within 24 hours.

- - 1. **Procedures for dealing with emergencies**

The investigator is responsible for obtaining information about all urgent medical conditions of patients during the course of the study. The text of the Patient Information Sheet and the informed consent form contains the contact information of the Investigator. Patients will be advised to contact the Investigator in the event of any medical emergencies during the study.

- - 1. **Registration deadlines for AEs**

In this study, AE registration will be carried out from the moment the patient signs the informed consent form and up to 30 days after the patient's last visit to the study center or the last protocol procedure.

- - 1. **Expectancy**

Please refer to Section 3.4 "Previous Application Experience". At the moment, there have been no specific adverse reactions to Treamide that could be attributed to the expected adverse reactions.

- - 1. **Clinically Significant Laboratory Abnormalities**

When evaluating the results of laboratory tests, the Investigator may not classify single violations of the reference limits of laboratory parameters as deviations if they are not accompanied by any clinical manifestations, do not require additional examination or treatment, and are not confirmed by the values of interrelated laboratory parameters (clinically insignificant deviations).

Any clinically significant laboratory abnormalities noted at screening should be attributed to the patient's medical history. After screening, new or worsening clinically significant laboratory abnormalities should be reported as AEs in the primary documentation and in the CRF.

- 1. **Early withdrawal of patients from the study or withdrawal of the study drug**

The patient has the right to refuse participation in the study at any stage and without explanation.

A patient should be excluded from a clinical trial if:

- Investigator considers it medically necessary
- The patient withdraws his consent
- Patient fails to comply with prescribed doses of study drug or study-related procedures
- An AE or SAE has occurred that could adversely affect the safety and well-being of the patient
- Decrease in FEV1 by > 15% of baseline or < 50% of predicted
- Lower respiratory tract infection

The following reasons warrant immediate discontinuation of the study drug:

- Patient started another study drug
- The onset of pregnancy in the patient
- Significant protocol violations that may affect patient safety and the integrity of study data

The Investigator should, if possible, discuss the need for exclusion of the patient with the Medical Monitor in advance or communicate the exclusion of the patient within 48 hours. The reason for exclusion of the patient should be indicated in the primary documentation and in the CRF. If a patient withdraws from the study for more than one reason, only the underlying reason should be reported. The reason for withdrawal of consent by a patient due to an AE or SAE should be the occurrence of an AE or SAE.

In addition, the Sponsor has the right to terminate the study at any time. The Investigator has the right to terminate the study at any time for medical or regulatory reasons. The completion of the study should only take place after mutual consultation between the Investigator and the Sponsor.

If the study is terminated early, all patients must undergo early termination visit procedures and all study materials must be returned to the Sponsor or its representative.

Follow-up for AEs, SAEs, and pregnancy ongoing at the time of termination of the study should be carried out according to the Protocol, unless otherwise safety follow-up procedures are not established by mutual agreement between the Investigator and the Sponsor.

- 1. **Validity of measurements**

1. Evaluations of the efficacy and safety of therapy aimed at preventing fibrotic changes after suffering COVID-19 pneumonia were proposed in accordance with the Interim Guidelines "Medical Rehabilitation in New Coronavirus Infection (COVID-19)" and recommendations for conducting clinical trials in patients with IPF [13, sixteen]. Double-blind placebo control will allow to maximally objectify the obtained data on the efficacy and safety of the study drug.
2. RESEARCH PROCEDURES
   1. **Screening**

**(Visit 1, Week -2, Visit window -28 days)**

- Procedure for obtaining informed consent
- Collection of demographic data and medical history
- Physical examination
- Body weight, height, BMI
- Vital Signs Assessment
- Pulse oximetry with SpO2 measurement
- Assessment of the patient's condition using the mMRC Breathlessness Scale
- Nasopharyngeal and/or oropharyngeal swab for PCR for SARS-CoV-2 RNA
- Taking blood and urine samples for laboratory tests: general and biochemical blood tests, general urinalysis
- Pregnancy test for women of childbearing potential (including women less than two years of menopause)
- 12-lead ECG
- CT scan of the chest
- Spirometry (FEV1, FVC, FEV1/FVC)
- Body plethysmography (DLCO, TLC, FRC)
- Evaluation of inclusion / non-inclusion criteria
- Evaluation of AEs and concomitant therapy

**In the event that screening procedures are initiated as part of the patient's discharge from the hospital after hospitalization of a patient diagnosed with COVID-19 pneumonia, sufficient time should be allowed for the completion of quarantine measures (about 2 weeks) before assessing the extent of damage and lung function.**

The screening may use the results of examinations carried out as part of routine medical practice before obtaining informed consent (taking into account the window of the screening visit), except for the assessment on the mMRC Breathlessness Scale, CT of the chest, spirometry and body plethysmography with the definition of DLCO - these examinations should be carried out as much as possible close to the planned date of randomization (no earlier than 5 days before it).

After the Investigator receives the results of all studies and confirms compliance with all inclusion/non-inclusion criteria, patients will be included in the study.

- 1. **Study therapy period**
     1. **Visit 2, Week 0**
- Physical examination
- Body mass
- Vital Signs Assessment
- Pulse oximetry with SpO2 measurement
- Assessment of the patient's condition using the mMRC Breathlessness Scale
- Assessment of the patient's condition using the KBILD questionnaire
- 6 Minute Walk Test (6MWD, Borg Scale)
- Spirometry (FEV1, FVC, FEV1/FVC)
- 12-lead ECG
- Taking blood and urine samples for laboratory tests: general and biochemical blood tests, general urinalysis
- Obtaining a blood sample from all patients for the PK study of the C _trough_ parameter before taking the study drug
- Pregnancy test for women of childbearing potential (including women less than two years of menopause)
- Final assessment of inclusion/non-inclusion criteria
- Patient Randomization
- Distribution of the study drug
- Initiation of investigational therapy according to treatment regimen
  - 1. **Visit 3, Week 1, Visit window ± 1 day**
- Evaluation of AEs and concomitant therapy
- Vital Signs Assessment
- Body mass
- Pulse oximetry with SpO2 measurement
- Assessment of the patient's condition using the mMRC Breathlessness Scale
- Spirometry (FEV1, FVC, FEV1/FVC)
- Study drug return and compliance assessment
- Release of the investigational drug
  - 1. **Visit 4, Week 2, Visit window ± 2 days**
- Evaluation of AEs and concomitant therapy
- Physical examination
- Body mass
- Vital Signs Assessment
- Pulse oximetry with SpO2 measurement
- Assessment of the patient's condition using the mMRC Breathlessness Scale
- Assessment of the patient's condition using the K-BILD questionnaire
- 6 Minute Walk Test (6MWD, Borg Scale)
- Spirometry (FEV1, FVC, FEV1/FVC)
- 12-lead ECG
- Body plethysmography (DLCO, TLC, FRC)
- Taking blood and urine samples for laboratory tests: general and biochemical blood tests, general urinalysis
- Obtaining a blood sample from all patients for the PK study of the Ctrough parameter prior to taking the study drug
- Pregnancy test for women of childbearing potential (including women less than two years of menopause)

•

- Study drug return and compliance assessment
- Release of the investigational drug
  - 1. **Visit 5, Week 3, Visit window ± 1 day**
- Evaluation of AEs and concomitant therapy
- Vital Signs Assessment
- Body mass
- Pulse oximetry with SpO2 measurement
- Assessment of the patient's condition using the mMRC Breathlessness Scale
- Spirometry (FEV1, FVC, FEV1/FVC)
- Study drug return and compliance assessment
- Release of the investigational drug
  - 1. **Visit 6, Week 4, Visit window ± 2 days (End of therapy)**
- Evaluation of AEs and concomitant therapy
- Physical examination
- Body mass
- Vital Signs Assessment
- Pulse oximetry with SpO2 measurement
- Assessment of the patient's condition using the mMRC Breathlessness Scale
- 6 Minute Walk Test (6MWD, Borg Scale)
- Assessment of the patient's condition using the K-BILD questionnaire
- Nasopharyngeal and/or oropharyngeal swab for PCR for SARS-CoV-2 RNA
- Taking blood and urine samples for laboratory tests: general and biochemical blood tests, general urinalysis
- Obtaining a blood sample from all patients for the PK study parameter C _trough_
- Pregnancy test for women of childbearing potential (including women less than two years of menopause)
- 12-lead ECG
- CT scan of the chest
- Spirometry (FEV1, FVC, FEV1/FVC)
- Body plethysmography (DLCO, TLC, FRC)
- Study drug return and compliance assessment

Thereafter, patients will continue treatment in accordance with applicable general guidelines.

- 1. **observation period.**
     1. **Visit 7, Week 6, Visit window ± 3 days**
- Evaluation of **SAEs** and concomitant therapy alone
- Physical examination
- Body mass
- Vital Signs Assessment
- Pulse oximetry with SpO2 measurement
- Assessment of the patient's condition using the mMRC Breathlessness Scale
- Taking blood and urine samples for laboratory tests: general and biochemical blood tests, general urinalysis
- Pregnancy test for women of childbearing potential (including women less than two years of menopause)
- 12-lead ECG
  1. **Unscheduled examinations**

At the discretion of the Investigator, unscheduled examinations may be performed on patients at any time during the course of the clinical trial for safety reasons, if a repeat examination or procedure is necessary. The Investigator can carry out the necessary procedures, including laboratory and instrumental studies. Unscheduled examinations should be registered in the primary documentation and in the eCRF. Conducting unscheduled examinations should not affect the schedule of scheduled examinations provided for in the protocol of this clinical trial.

- 1. **Early termination of the study**

Early termination of study participation after Visit 2 and at least one dose of study drug should be performed, if possible, at an early termination visit (EC). As part of an early end visit, if possible, follow the End of Therapy visit procedures (section 7.2.5).

1. **QUALITY ASSURANCE**

This clinical trial will be conducted in accordance with the Standard Operating Procedures (SOPs) of the Sponsor and/or its representative, the Good Clinical Practice Guidelines of the International Conference on Harmonization (ICH GCP) and the Eurasian Economic Union (EAEU), the principles set forth in the Declaration of Helsinki, and also in accordance with applicable law and applicable regulatory requirements of the Russian Federation. Compliance will be ensured through audits of study sites and study data.

The Investigator will enter the data required by this protocol into the eCRF provided by the Sponsor or its representative. Monitors should visit each study site at the frequency described in the monitoring plan to check the eCRF for completeness and accuracy. Any discrepancies between the original documentation and the completed eCRF should be asked clarifying questions and the Investigator should answer and/or correct these discrepancies. After entering all the necessary information into the eCRF and resolving all clarifying questions, the Investigator must sign the eCRF for each patient. Upon completion of the study, the database (eCRF) will be provided to the Sponsor, as well as to the research centers.

1. PLANNED STATISTICAL METHODS
   1. **General provisions**

A statistical analysis plan will be prepared before the database of this study is closed for final analysis. Any deviations from the planned analysis will be described and justified in the overall final study report.

- 1. **Determination of the sample size**

The sample size was determined in accordance with the methodology of one-stage non-comparative design of phase II clinical trials (RP A’Hern, 2001).

At the moment, the process of recovery of lung function in patients who have undergone COVID-19 is not well understood [20]. In this study, 25% of patients in the Placebo group and 50% of patients in the Treamide group are expected to achieve a clinically significant change in FVC and/or DLCO at Week 4 from baseline. For α = 0.05 (one-tailed), power 80%, p0=25% and p1=50%, 26 patients must be included in the analysis to test the hypothesis. 11 or more responses in 26 included patients will allow us to reject the null hypothesis (Н _0_ : p ≤ p _0_ ) in favor of the alternative one (W1: p ≥ p _1_ ) and thus make a positive decision regarding the possibility of further study of Treamide in phase III.

Taking into account the possible early withdrawal (impossibility of evaluating the primary endpoint) not exceeding 13%, 30 patients will be included in each group of study therapy. Thus, 6o patients will be randomized into the study.

- 1. **Randomization**

Will be randomized into two groups in a 1:1 ratio using the IWRS system. Randomization will be stratified by risk factors (age ≥ 60 years and/or chronic comorbidity) and baseline CT lung involvement.

- 1. **Populations for analysis**
     1. **Full analysis set (FAS)**

The Full Analysis Set (FAS) is defined as all randomized patients who received at least one dose of study drug and have at least one post-baseline efficacy score. The FAS population is the main population for efficacy analysis.

- - 1. **Protocol Population (Per Protocol, PP)**

Per Protocol (PP) populations correspond to patients in the entire analysis population who will receive the study therapy in full and will not have significant deviations from the protocol. Major protocol violations will be described in more detail in the Statistical Analysis Plan and identified prior to closing the database.

- - 1. **Safety Population**

All patients who received at least one dose of study drug will be included in the safety analysis.

- - 1. **PK Population**

All patients who received at least one dose of study drug and for whom sufficient PK samples were obtained to evaluate at least one PK parameter will be included in the pharmacokinetics population.

- 1. **Patient distribution, demographic characteristics and baseline analysis**

Patient distribution, demographics, and baseline characteristics will be presented using descriptive statistics. The number and percentage of patients taking concomitant medications will be presented in frequency tables by therapeutic class and name of the active substance. The number of patients with pre-existing and comorbid medical conditions will be presented. Dosing information, including daily doses, exposure time, and total dose, will be presented descriptively by treatment group.

- 1. **Procedures for accounting for missing, unanalysable and doubtful data**

Missing data will not be replaced.

- 1. **Efficacy analysis**
     1. **Primary efficacy endpoint**

The primary endpoint is the frequency of achieving a clinically meaningful change in FVC and/or DLCO at Week 4 from baseline. Clinically significant changes are considered to be a relative increase in FVC of ≥10% or

a relative increase in FVC ranging from ≥ 5% to < 10% and a relative increase in DLCO by > 15% [13, 14].

According to the RP algorithm A'Hern for single-stage phase II studies with unilateral α=0.05 and 80% power, 11 or more of 26 patients included in the analysis who achieved a clinically significant change in FVC and/or DLCO at Week 4 from baseline are sufficient to accept decisions on the advisability of further study of this dose in phase III (taking into account the evaluation of additional parameters of efficacy and safety). If the placebo arm also has a Week 4 response rate of 11 or more in the 26 included patients, then the primary endpoint assessment may be deferred to an earlier study visit.

If the number of responses to therapy in the Treamide group of 26 included patients is 10 or less, it will be concluded that there is no sufficient efficacy.

- - 1. **Secondary efficacy endpoints**

Secondary efficacy endpoints include:

- Average change in distance walked in 6 minutes (6MWD) in Week 2 and Week 4 from baseline (based on the 6 Minute Walk Test);
- Mean change in Borg score at Week 2 and Week 4 from baseline (based on the 6 Minute Walk Test);
- Mean relative change in spirometry lung function parameters (FEV1, FVC, FEV1/FVC) at Week 1, Week 2, Week 3, and Week 4 from baseline;
- Mean relative change in body plethysmography lung function parameters at Week 2 and Week 4 from baseline (DLCO, TLC, and FRC);
- Rate of reduction in lung injury (improvement) on CT scan at Week 4 from baseline;
- Mean change in severity on the mMRC Breathlessness Scale at Week 1, Week 2, Week 3, and Week 4 from baseline;
- The mean change in total scores on the KBILD Questionnaire in Week 2 and Week 4 from baseline.

For secondary endpoints, intergroup comparisons of frequencies and categorical data will be made using the chi-square test or Fisher's exact test, continuous data using the non-parametric Mann-Whitney test or Student's t-test.

- 1. **Safety analysis**

Study drug dosing information, including daily doses, time of exposure, and total dose, will be presented descriptively for each cohort.

Safety will be assessed based on the incidence of AEs and SAEs based on subjective complaints, physical examination, vital signs, laboratory tests, and ECG. The number and percentage of patients with AEs and SAEs will be tabulated by organ system class and preferred term (according to MedDRA), by study drug association, and severity. The results of laboratory studies will be summarized in tables of changes. Vital and laboratory parameters, SpO2 and ECG data will be presented using descriptive statistics.

- 1. **Pharmacokinetic analysis**

The following PK parameters of the study drug will be assessed: C _max_, T _max_, AUC _0-t_ and C _trough_. Validated software ™ WinNonlin ® (CERTARA, Pharsight, USA) will be used to calculate pharmacokinetic parameters. The analysis will be carried out using descriptive statistics methods.

The study therapy will be blinded prior to the PK study. PK analysis for patients receiving placebo will only be performed if there is doubt about the correctness of the blinding procedure. To confirm the absence of the study drug in the blood plasma of patients who received placebo, analysis can be performed at points corresponding to the calculated Tmax values.

- 1. **Interim report**

Based on the results of all patients completing the treatment period (week 4), an interim report can be prepared. The analysis will be based on the evaluation of the endpoints: the frequency of achieving a clinically significant change in FVC and / or DLCO at Week 4 from baseline, the average change in distance traveled over 6 minutes (6MWD) at Week 2 and Week 4 from baseline (based on the Test 6 minute walk). mean change in Borg score at Week 2 and Week 4 from baseline (based on the 6 Minute Walk Test), mean relative change in spirometry lung function parameters (FEV1, FVC, FEV1/FVC) at Week 1, Week 2, Week 3 and Week 4 from baseline, mean relative change in body plethysmography lung function parameters at Week 2 and Week 4 from baseline (DLCO, TLC, and FRC), rate of decline (improvement) in CT at Week 4 from baseline , mean change in severity on the mMRC Breathlessness Scale at Week 1, Week 2, Week 3 and Week 4 from baseline, mean change from baseline on the KBILD Questionnaire total score at Week 2 and Week 4 from baseline. and also based on safety assessment (based on the incidence of AE and SAE according to subjective complaints, physical examination, vital signs, laboratory tests and ECG).

The interim report will be submitted for consideration to the Ministry of Health of the Russian Federation.

1. ADMINISTRATIVE PROCEDURES
   1. **Legal aspects**

The names of all patients must be kept strictly confidential. Patients are identified by their assigned number, initials and date of birth. Patients should be advised that any information held by the study site, the Sponsor or its representatives will be held in strict compliance with the principle of confidentiality in accordance with regulatory requirements.

- - 1. **Responsibilities of the Investigator**

The investigator is required to conduct the clinical trial in accordance with the requirements of this protocol, the Good Clinical Practice Guidelines of the International Conference on Harmonization (ICH GCP) and the Eurasian Economic Union (EAEU), the principles laid down in the Declaration of Helsinki, as well as in accordance with the current legislation and applicable regulations. requirements.

The Investigator must agree to conduct monitoring, audits and inspections in the research center and at any time, upon request, provide direct access to the study materials to the Sponsor and its representatives, the Independent Ethics Committee and authorized bodies.

The investigator must keep the originals of all informed consent forms signed as part of the study, as well as a complete list of study subjects, including full name, assigned number, initials, date of birth, address, and telephone number, so that they can be identified later, if necessary. These documents must not be copied for distribution to the Sponsor or its representatives.

- 1. **Monitoring procedures**

The Investigator must provide direct access to all primary documentation, the eCRF and the Investigator File to the Sponsor and its representatives, the IEC and authorized bodies. Primary documentation includes source documents, data and records, including medical histories, outpatient records, laboratory records, notes, drug dispensing journals, records of automatic devices, verified and certified copies or extracts, photo negatives, microfilms or magnetic media, CT scans. , any records related to the patient, including those stored in a pharmacy, laboratories and departments of instrumental diagnostics used in a clinical trial.

Monitoring will be conducted by the Sponsor or its representative in accordance with their SOP and the Monitoring Plan developed for this study.

- 1. **Data registration in eCRF**

The investigator should transfer the data obtained during the clinical trial from the primary documentation to the eCRF to the extent required. The rules for completing the eCRF will be reviewed by the Investigator during the Investigator meeting and/or during the initiation visit. If the Investigator delegates the authority to complete the eCRF to other research facility staff, their names, titles, signatures, and initials must be entered on the study facility staff assignment sheet and provided to the Sponsor or its representative.

When corrections are made to the eCRF, a documentary trace must remain, i.e. Before and after the change, reason (if applicable), date and who made the change should be visible. Completed eCRFs must be signed by the Investigator.

- 1. Storage of documentation

Primary documentation, eCRF data, and the Investigator's File must be kept at the research facility or in a specially designed archive with limited access for at least 25 years after the completion of the study or until the date specified by the Sponsor. The sponsor must be informed if the documentation is transferred to other persons or to another institution. Materials related to the clinical study will be kept by the Sponsor and its representatives in accordance with regulatory requirements.

- 1. Ethical aspects
     1. Independent Ethics Committee

Prior to the commencement of a clinical trial, all study sites will receive written approval from Independent Ethics Committees (IECs) in accordance with the Good Clinical Practice Guidelines of the International Conference on Harmonization (ICH GCP) and the Eurasian Economic Union (EAEU), the principles set out in the Declaration of Helsinki, and also in accordance with applicable law and applicable regulatory requirements of the Russian Federation. The following documents must be submitted to the IEC for review: study protocol and amendments to it, patient information sheet and informed consent form, written materials to be provided to patients, investigator's brochure, information on the safety of the use of the investigational drug, information on payments and compensation to patients, scientific biography of the Investigator and other documents on request.

A list of IEC members and a statement of its organization and operation in accordance with the principles of good clinical practice and regulatory requirements must be provided to the Sponsor.

- - 1. Ethical Conduct of Clinical Research

The procedures described in the clinical trial protocol relating to its conduct, evaluation and documentation of results are designed to ensure that the Sponsor and Investigator follow the International Conference on Harmonization (ICH GCP) and the Eurasian Economic Union (EAEU) Good Clinical Practice Guidelines. This clinical study will also be conducted in accordance with applicable law and applicable regulatory requirements. This includes the possibility of audits and inspections by representatives of the Sponsor and/or authorized bodies. The Investigator must agree to conduct monitoring, audits and inspections in the research center and at any time, upon request, provide direct access to the study materials to the Sponsor and its representatives, the Independent Ethics Committee and authorized bodies.

- - 1. Informed Consent

Before starting a clinical investigation, the Investigator must obtain written approval from the IEC of the Patient Information Sheet and Informed Consent Form, as well as any other written information to be provided to patients. The IEC's written approval and approved documents must be included in the Study File.

The process of obtaining informed consent must be carried out in accordance with the Good Clinical Practice Guidelines of the International Conference on Harmonization (ICH GCP) and the Eurasian Economic Union (EAEU), the principles set out in the Declaration of Helsinki, as well as in accordance with the current legislation and applicable regulatory requirements of the Russian Federation .

The informed consent form must be hand-signed and dated by the patient prior to the commencement of any study procedures, and must be retained with the patient's primary documentation. The process of obtaining informed consent should be described in detail in the primary documentation, including the fact that the patient consented to participate in this clinical study, the date of signing and the version of the informed consent form.

- 1. **Research funding**

The study is being conducted and fully funded by PHARMENTERPRICES LLC. All costs associated with the conduct of the study, including the payment of state fees, insurance premiums, payments to research centers, Investigators and third parties involved in the study, as well as the provision of the study drug and other study materials, are covered by PHARMENTERPRICES LLC.

Participation in the study does not require any financial costs from patients. All research procedures, including visits to the center, laboratory and instrumental studies, will be performed free of charge. Participation in this study does not imply the payment of monetary rewards to patients.

The life and health of patients participating in this clinical trial will be insured in accordance with the legislation of the Russian Federation (Article 44 of the Federal Law "On the Circulation of Medicines" No. 61-FZ of April 12, 2010, Government Decree No. 714 of September 13, 2010 d. subject to current changes). Upon signing the informed consent form, patients will receive a Compulsory Life and Health Insurance Policy for a patient participating in clinical trials of a medicinal product.

1. **PLAN OF PUBLICATIONS**

Both complete and partial research results obtained under this protocol, as well as any other information provided by the Sponsor for the conduct of the study, must not be published or transferred to a third party without the consent of the Sponsor. Investigators involved in the study are required to provide the Sponsor with complete and accurate data obtained during the course of the study.

1. **REFERENCES**
2. MINISTRY OF HEALTH OF THE RUSSIAN FEDERATION. Temporary guidelines. Prevention, diagnosis and treatment of new coronavirus infection (COVID-19). Version 7.0 from 06/03/2020
3. Wang D., et al. Clinical characteristics of 138 hospitalized patients with 2019 novel coronavirus-infected pneumonia in Wuhan, China. JAMA. 2020 Mar 17; 323(11): 10611069.
4. Huang C., et al. Clinical features of patients infected with 2019 novel coronavirus in Wuhan, China. Lancet 2020;395(10223):497-506.
5. Li Q. et al. Early transmission dynamics in Wuhan, China, of novel coronavirus-infected pneumonia. N Engl J Med. 2020 Mar 26;382(13):1199-1207.
6. Chen N. et al. Epidemiological and clinical characteristics of 99 cases of 2019 novel coronavirus pneumonia in Wuhan, China: a descriptive study. Lancet

2020;395(10223):507-13.

1. Miriam Merad, Jerome C Martin, Pathological Inflammation in Patients With COVID-19: A Key Role for Monocytes and Macrophages, Nat Rev Immunol. 2020 May 6;1-8. https://www.nature.com/articles/s41577-020-0331-4.
2. Kai Liu et al. Respiratory Rehabilitation in Elderly Patients With COVID-19: A Randomized Controlled Study Complement Ther Clin Pract. 2020 May;39:101166, https://doi.org/10.1016/j.ctcp.2020.101166
3. Mo, X., Jian, W., Su, Z., Chen, M., Peng, H., Peng, P., Zhong, N. Abnormal pulmonary function in COVID-19 patients at time of hospital discharge. Eur Respir J. 2020 Jun 18;55(6):2001217
4. George, P. M., Wells, A. U., Jenkins, R. G. Pulmonary fibrosis and COVID-19: the potential role for antifibrotic therapy. Lancet Respir Med. 2020 May 15
5. Efficacy and Safety of Nintedanib in the Treatment of Pulmonary Fibrosis in Patients With Moderate to Severe COVID-19. https://clinicaltrials.gov/ct2/show/NCT04338802
6. A Study to Evaluate the Efficacy and Safety of Pirfenidone With Novel Coronavirus Infection. https://clinicaltrials.gov/ct2/show/NCT04282902
7. Spagnolo, P., Balestro, E., Aliberti, S., et al. Pulmonary fibrosis secondary to COVID-19: a call to arms? Lancet Respir Med. 2020 May 15
8. Raghu, G., Collard, H. R., Anstrom, K. J., et al. Idiopathic Pulmonary Fibrosis: Clinically Meaningful Primary Endpoints in Phase 3 Clinical Trials. Am J Respir Crit Care Med. 2012 May 15; 185(10): 1044-1048.
9. Yamano, Y., Taniguchi, H., Kondoh, Y., et al. Multidimensional improvement in connective tissue disease-associated interstitial lung disease: Two courses of pulse dose methylprednisolonefollowedbylow-doseprednisoneand tacrolimus. Respirology. 2018 Nov;23(11):1041-1048
10. WHO R&D Blueprint/novel Coronavirus/COVID-19 Therapeutic Trial Synopsis/Draft February 18,2020. https://www.who.int/docs/default-source/blue-print/covid-19- therapeutic-trial-synopsis.pdf?sfvrsn=44b83344_1&download=true
11. Temporary guidelines. Medical rehabilitation for novel coronavirus infection (COVID 19). Version 1 (05/21/2020).
12. Clinical guidelines: Idiopathic pulmonary fibrosis. Russian Respiratory Society, 2016.
13. American Thoracic Society. ATS Statement: Guidelines for the Six-Minute Walk Test. Am J Respir Crit Care Med Vol 166. pp 111-117, 2002 DOI: 10.1164/rccm.166/1/111 Internet address: www.atsjournals.org
14. Chikina S.Yu. Out-of-laboratory stress tests in pathology of the respiratory system. Creative. surg. and oncol. 2010;1:820-95. Available at: https://cyberleninka.ru/article/n/vnelaboratornye-nagruzochnye-testy-pri-patologii-organov-dyhaniya
15. Liu, K., Zhang, W., Yang, Y., Zhang, J., Li, Y., & Chen, Y. (2020). Respiratory rehabilitation in elderly patients with COVID-19: A randomized controlled study. ComplementaryTherapiesinClinicalPractice,39,101166. doi:10.1016/j.ctcp.2020.101166
16. National Commission for the Protection of Human Subjects of Biomedical and Behavioral Research. The Belmont Report: ethical principles and guidelines for the protection of human subjects of research. Washington, DC: Dept of Health, Education, and Welfare (DHEW) publication nos. (OS) 78-0012, appendix I; (OS) 78-0013, appendix II; and (OS) 78-0014; 1978
17. Efficacy and Safety of Nintedanib in the Treatment of Pulmonary Fibrosis in Patients With ModeratetoSevereCOVID-19. https://clinicaltrials.gov/ct2/show/record/NCT04338802?term=nintedanib&draw=2&rank=12&view=record
18. Rules of Good Clinical Practice of the Eurasian Economic Union. Approved by the Decision of the Council of the Eurasian Economic Commission dated November 3, 2016 No. 79.
19. Collard HR, King TE Jr, Bartelson BB, Vourlekis JS, Schwarz MI, Brown KK. Changes in clinical and physiologic variables predict survival in idiopathic pulmonary fibrosis. Am J Respir Crit Care Med 2003; 168: 538-542.
20. King TE Jr, Safrin S, Starko KM, Brown KK, Noble PW, Raghu G, Schwartz DA. Analyzes of efficacy end points in a controlled trial of interferon-gamma1b for idiopathic pulmonary fibrosis. Chest 2005; 127: 171- 177.
21. Bethany B Moore, Cory M Hogaboam. “Murine models of pulmonary fibrosis”. Am J Physiol Lung Cell Mol Physiol. 2008 Feb;294(2):L152-60,
22. Amber L Degryse, William E Lawson. “Progress toward improving animal models for idiopathic pulmonary fibrosis”. Am J Med Sci. 2011 Jun;341(6):444-9
23. Antje Moeller, Kjetil Ask, David Warburton, Jack Gauldie, Martin Kolb. “The bleomycin animal model: a useful tool to investigate treatment options for idiopathic pulmonary fibrosis?” Int J Biochem Cell Biol. 2008;40(3):362-82
24. Yan Cai, Lei Zhu, Fan Zhang, Gang Niu, Seulki Lee, Shioko Kimura, Xiaoyuan Chen. “Noninvasive monitoring of pulmonary fibrosis by targeting matrix metalloproteinases (MMPs)”, Mol Pharm. 2013 Jun 3;10(6):2237-47,
25. Viranuj Sueblinvong, David C Neujahr, S Todd Mills, Susanne Roser-Page, Jeffrey D Ritzenthaler, David Guidot, Mauricio Rojas, Jesse Roman. “Predisposition for disrepair in the aged lung“ Am J Med Sci. 2012 Jul;344(1):41-51
26. William E Lawson, James E Loyd. “The genetic approach in pulmonary fibrosis: can it provide clues to this complex disease?” Proc Am Thorac Soc. 2006 Jun;3(4):345-9,
27. William E Lawson, Vasiliy V Polosukhin, Georgios T Stathopoulos, Ornella Zoia, Wei Han, Kirk B Lane, Bo Li, Edwin F Donnelly, George E Holburn, Kenneth G Lewis, Robert D Collins, William M Hull, Stephan W Glasser, Jeffrey A Whitsett, Timothy S Blackwell. “Increased and advanced pulmonary fibrosis in surfactant protein C-deficient mice following intratracheal bleomycin” Am J Pathol . 2005 Nov;167(5):1267-77
28. Peter M George, Athol U Wells, R Gisli Jenkins. “Pulmonary fibrosis and COVID-19: the potential role for antifibrotic therapy” Lancet Respir Med. 2020 May 15;S2213- 2600(20)30225-3
29. Paolo Spagnolo, Elisabetta Balestro, Stefano Aliberti, Elisabetta Cocconcelli, Davide Biondini, Giovanni Della Casa, Nicola Sverzellati, Toby M Maher. “Pulmonary fibrosis secondary to COVID-19: a call to arms?” Lancet Respir Med. 2020 May 15;S2213- 2600(20)30222-8
30. FN Novikov, VS Stroylov, IV Svitanko, VE Nebolsin, "Molecular bases of COVID-19 pathogenesis", RUSS CHEM REV, 2020, 89 (8), 858-878
31. Efficacy and Safety of Nintedanib in the Treatment of Pulmonary Fibrosis in Patients With Moderate to Severe COVID-19. Sponsor: Huilan Zhang. ClinicalTrials.gov Identifier: NCT04338802. April 2020
32. A Study to Evaluate the Efficacy and Safety of Pirfenidone With Novel Coronavirus Infection. Sponsor: Huilan Zhang. ClinicalTrials.gov Identifier: NCT04282902. February 25, 2020
33. Jessica Wagner, C Leah Kline, Lanlan Zhou, Vladimir Khazak, Wafik S El-Deiry. “Antitumor effects of ONC201 in combination with VEGF-inhibitors significantly impacts colorectal cancer growth and survival in vivo through complementary non-overlapping mechanisms.” J Exp Clin Cancer Res. 2018: 37, 1.
34. Yi Wang , Ruo-Wu Shen , Bing Han , Zhen Li , Le Xiong , Feng-Yu Zhang , Bei-Bei Cong, Bei Zhang. “Notch signaling mediated by TGF-β/Smad pathway in concanavalin A- induced liver fibrosis in rats”. World J Gastroenterol. 2017 Apr 7; 23(13): 2330-2336
35. Guidelines for the provision and maintenance of peripheral venous access. Developed and approved by the Association of Nurses of Russia. http://medsestre34.ru/assets/files/files/method_rekom.pdf
36. Russian respiratory society. FEDERAL CLINICAL RECOMMENDATIONS FOR THE USE OF THE SPIROMETRY METHOD, 2013
37. A series of monographs of the Russian Respiratory Society, ed. A.G. Chuchalin. Functional diagnostics in pulmonology. Ed. Z.R. Aisanova, A.V. Chernyak. 2016. - 184 p., ill.
38. Ojo A. S., Balogun S. A., Williams O. T., et al. Pulmonary Fibrosis in COVID-19 Survivors: Predictive Factors and Risk Reduction Strategies. Pulmonary Medicine, 2020, 1-10. doi:10.1155/2020/6175964
39. Das K.M., Lee E.Y., Singh R., et al. Follow-up chest radiographic findings in patients with MERS-CoV after recovery. Indian J Radiol Imaging. 2017 Jul-Sep;27(3):342-349
40. Choi W.J., Lee K.N., Kang E.J., et al. Middle East respiratory syndrome Coronavirus infection: a case report of serial computed tomographic findings in a young male patient. Korean J Radiol 2016;17(1):166-170

Appendix 1. mMRC (modified Medical Research Council) dyspnea score

| Degree | Severity | Appearance |
| --- | --- | --- |
| 0 | No | I only feel short of breath when I exert myself heavily |
| 1 | light | I get out of breath when I walk quickly on level ground or climb a gentle hill |
| 2 | average | Because of my shortness of breath, I walk more slowly on level ground than people of the same age, or I stop breathing when I walk on level ground at my normal pace |
| 3 | heavy | I am out of breath after walking about 100m or after walking for a few minutes on level ground |
| 4 | very heavy | I am too short of breath to leave the house or suffocate when I get dressed or undressed |

Source: Chronic obstructive pulmonary disease, Clinical guidelines. Russian respiratory society.

**Annex 2. 6-minute walk test (6MWD, Borg scale) (sample)**

The 6MWD (6-minute walk) test is carried out in a level, straight, closed hallway with a hard floor that is easy to walk on. Weather permitting, the test can be performed outdoors. The corridor is marked every 3 m. The beginning and end of the distance are marked with cones (similar to the orange cones used in road repairs), which are placed at a distance of 0.5 m from the ends of the corridor.

Necessary equipment

1. Chronometer or stopwatch.
2. Two small cones to indicate turning points.
3. Mechanical distance counter (pedometer).
4. A chair that easily moves along the distance.
5. Source of oxygen.
6. Tonomanometer.
7. Telephone.
8. Portable defibrillator.
9. Emergency aid.

Preparing the Patient for 6MWD

1. Comfortable clothing that does not restrict movement.
2. Shoes that are comfortable for walking.
3. The patient is allowed to use walking aids (canes, etc.) that he uses on a daily basis.
4. The patient's usual regimen of medication is maintained.
5. Before the test, the patient is allowed to take a light breakfast (lunch).
6. Vigorous physical activity is not recommended for 2 hours before the test.

Testing

Prior to the start of the study, the patient should sit quietly in a chair near the starting line for at least 10 minutes. During this time, it is necessary to evaluate the contraindications for the study, measure the pulse and pressure, and make sure that the patient's clothing and shoes are suitable for the test.

Pulse oximetry should preferably be carried out during the entire test. To monitor saturation, the Investigator does not need to walk the entire distance with the patient. The pulse oximeter should be attached to the patient's clothing, be light (no more than 900 g) and comfortable in shape so that the patient does not have to hold it, and so that it does not interfere with walking.

To assess dyspnea on exertion, either the Borg scale or the visual analogue scale is used. Before starting 6MWD, you need to show the scale to the patient and ask him to determine the degree of respiratory discomfort and general fatigue on it. At the end of testing, the patient should be reminded of their initial scores and asked to re-evaluate these symptoms.

**Borg scale for assessing dyspnea**

| **Points** | **Severity of shortness of breath** |
| --- | --- |
| 0 | No shortness of breath at all |
| 0.5 | Very, very light (almost imperceptible) |
| 1 | Very mild shortness of breath |
| 2 | Mild (mild) shortness of breath |
| 3 | Average shortness of breath |
| 4 | Moderate shortness of breath |
| 5 | severe shortness of breath |
| 6 | Fairly severe shortness of breath |
| 7 | Very severe shortness of breath |
| 8 | Very, very severe shortness of breath |
| 9 | Extremely severe shortness of breath |
| 10 | Unbearable (extremely severe) shortness of breath |

The examiner should instruct the patient as follows: “The aim of this study is to walk as fast as possible within 6 minutes. This is a long enough time for walking, so you yourself determine the intensity of your load. You may experience increased shortness of breath or fatigue. At the same time, you can slow down the pace of walking, and if necessary, stop and rest. During rest, you can lean against the wall, but then you should continue walking. You need to walk back and forth along the corridor, bypassing the cones. You need to turn around the cones quickly and then without delay continue to move in the opposite direction. It is recommended to walk one circle and demonstrate to the patient how to walk.

When the patient starts walking, you need to turn on the timer.

It is impossible even with gestures to demand from the patient to increase the speed of walking. If the patient stopped during the study and needs to rest, the timer is not turned off for this time. If the patient refuses to continue walking (or the investigator believes that the test should be terminated), the chair should be moved and the patient should be seated in it and the distance covered, the time and the reason for the premature termination of the study should be recorded in the protocol. For 15 sec. before the end of the test, the patient should be warned so that, after the request to stop, he stops where he will be at that moment, after which the Investigator himself approaches the patient and, if necessary, rolls up a chair to him. The stopping point is marked on the floor with a piece of bright tape or another marker.

After the test is completed, the saturation, pulse rate, degree of dyspnea and fatigue are again measured on the Borg scale or visual analog scale, and the patient is also asked about the reasons that prevented him from walking faster, and indicate them in the protocol.

To calculate the distance traveled, use the readings of a pedometer or markings on the wall of the corridor.

Appendix 3 K-BILD developed at King's College

**This questionnaire is designed to assess the impact of your lung disease on various aspects of your life. Read each question carefully and answer it by CIRCING the answer that best fits your situation. Please answer ALL questions as honestly as possible.**

1. In the past 2 weeks, I have experienced shortness of breath when climbing stairs, inclines, or uphill.
2. Everytime
3. In most cases
4. Repeatedly
5. Sometimes
6. From time to time
7. Rarely
8. Never
9. For the past 2 weeks, I have felt tightness in my chest due to my lung condition.
10. Always
11. Almost all the time
12. Often
13. Sometimes
14. Rarely
15. Almost never
16. Never
17. In the past 2 weeks, have you been concerned about the severity of your lung symptoms?
18. Always
19. Almost all the time
20. Often
21. Sometimes
22. Rarely
23. Almost never
24. Never
25. In the past 2 weeks, have you stopped doing breathless activities?
26. Always
27. Almost all the time
28. Often
29. Sometimes
30. Rarely
31. Almost never
32. Never
33. In the past 2 weeks, have you felt that you are in control of your lung disease?
34. Never
35. Almost never
36. Rarely
37. Sometimes
38. Often
39. Almost all the time
40. Always
41. In the past 2 weeks, have your lung symptoms been irritating or depressed?
42. Always
43. Almost all the time
44. Often
45. Sometimes
46. Rarely
47. Almost never
48. Never
49. Over the past 2 weeks, it happened that I really wanted to inhale, but it was difficult for me to do it.
50. Always
51. Almost all the time
52. Often
53. Sometimes
54. Rarely
55. Almost never
56. Never
57. For the past 2 weeks, my lung condition has been causing me anxiety.
58. Always
59. Almost all the time
60. Often
61. Sometimes
62. Rarely
63. Almost never
64. Never
65. In the past 2 weeks, how often have you experienced wheezing or whistling sounds coming from your chest?
66. Always
67. Almost all the time
68. Often
69. Sometimes
70. Rarely
71. Almost never
72. Never
73. In the past 2 weeks, how often did you feel like your lung condition was getting worse?
74. Always
75. Almost all the time
76. Often
77. Sometimes
78. Rarely
79. Almost never
80. Never
81. In the past 2 weeks, has your lung disease prevented you from doing your work or other daily tasks?
82. Always
83. Almost all the time
84. Often
85. Sometimes
86. Rarely
87. Almost never
88. Never
89. In the past 2 weeks, did you expect your lung symptoms to worsen?
90. Always
91. Almost all the time
92. Often
93. Sometimes
94. Rarely
95. Almost never
96. Never
97. In the past 2 weeks, how often have you found it difficult to carry something, such as grocery bags, because of your lung condition?
98. Always
99. Almost all the time
100. Often
101. Sometimes
102. Rarely
103. Almost never
104. Never
105. In the past 2 weeks, how often has your lung disease made you think that your life is coming to an end?
106. Always
107. Almost all the time
108. Often
109. Sometimes
110. Rarely
111. Almost never
112. Never
113. Has your financial situation worsened due to your lung disease?
114. To a large extent
115. To a large extent
116. Moderately
117. To an acceptable extent
118. To a small extent
119. Hardly ever
120. Not at all

# **Appendix 3 Statistical Methods Documentation**

**STATISTICAL ANALYSIS PLAN**

| **Protocol Number:** | COVID-TRE-03 |
| --- | --- |
| **Protocol name:** | A multicenter, randomized, double-blind, placebo-controlled pilot study to evaluate the efficacy and safety of Treamide in the rehabilitation of patients after COVID-19 pneumonia |
| **Study phase:** | II |
| **Therapeutic groups:** | Treamide, placebo |
| **Sponsor:** | **LLC "PHARMENTERPRISES"** |
| **PSA version:** | 1.0 |
| **Effective date:** | 03.12.2020 |

**Prepared by:**

| **biostatistician**  **Full name: Kholkin P.V.** | Signature and date: | (signed) 03.12.20 |
| --- | --- | --- |

**Approved:**

| **Data Processing Manager**  **Full name: Tikhonova U.V.** | Signature and date: | (signed) 03.12.20 |
| --- | --- | --- |
| **Medical Advisor**  **Full name: Kasyanova O.V.** | Signature and date: | (signed) 03.12.20 |
| **Project Manager**  **Name: Samokhina M.V.** | Signature and date: | (signed) 03.12.20 |
| **Sponsor Representative, if applicable**  **Name: Nebolsin V.E.** | Signature and date: | (signed) 03.12.20 |

| **By receiving this document, you agree that it is the Confidential Information of PHARMENTERPRICES LLC. You must not directly or indirectly publish, distribute or in any other way disclose the content or make available to third parties any part of this document. You must also use this document only for the purpose for which it was provided to you. You may disclose information only after the written permission of PHARMENTERPRICES LLC, or representatives of authorized government bodies, or by decision of the judicial authorities. The fact of any disclosure of the information contained in this document, authorized or not, must be immediately brought to the attention of PHARMENTERPRICES LLC.** |
| --- |

TABLE OF CONTENTS

[1 LIST OF ABBREVIATIONS AND DEFINITIONS OF TERMS 7](#_Toc102121679)

[2 INTRODUCTION 9](#_Toc102121680)

[3 GOALS AND OBJECTIVES OF THE RESEARCH 9](#_Toc102121681)

[3.1 Primary objective 9](#_Toc102121682)

[3.2 Additional objectives 9](#_Toc102121683)

[4 Study Design 9](#_Toc102121684)

[4.1 Randomization 12](#_Toc102121685)

[4.2 Blinding 12](#_Toc102121686)

[5 SAMPLE SIZING 12](#_Toc102121687)

[6 POPULATIONS ANALYZED 12](#_Toc102121688)

[6.1 Population of all randomized subjects 12](#_Toc102121689)

[6.2 Safety Population 12](#_Toc102121690)

[6.3 Efficacy population 12](#_Toc102121691)

[6.4 Population per protocol 12](#_Toc102121692)

[6.5 Pharmacokinetics population 12](#_Toc102121693)

[7 EFFICACY, SAFETY AND PHARMACOKINETICS VARIABLES 12](#_Toc102121694)

[7.1 Primary Efficacy Variable 13](#_Toc102121695)

[7.2 Secondary Efficacy Variables 13](#_Toc102121696)

[7.3 Safety Variables 13](#_Toc102121697)

[8 STATISTICAL METHODS AND STATISTICAL DATA ANALYSIS 13](#_Toc102121698)

[8.1 Disposition 13](#_Toc102121699)

[8.2 Demographic and other background characteristics 13](#_Toc102121700)

[8.3 Assessment of treatment compliance (compliance) 14](#_Toc102121701)

[8.4 EFFICACY ANALYSIS 15](#_Toc102121702)

[8.4.1 Primary Efficacy Variable 15](#_Toc102121703)

[8.4.2 Secondary Efficacy Variables 16](#_Toc102121704)

[8.5 SAFETY ANALYSIS 21](#_Toc102121705)

[8.5.1 Assessment of the duration of drug exposure 22](#_Toc102121706)

[8.5.2 Evaluation of adverse events and serious adverse events 22](#_Toc102121707)

[8.5.3 Concomitant Therapy and Related Procedures 22](#_Toc102121708)

[8.5.4 Assessment of clinical and laboratory parameters 23](#_Toc102121709)

[8.5.5 Physical examination 23](#_Toc102121710)

[8.5.6 Body mass 23](#_Toc102121711)

[8.5.7 Vital Signs and SpO2 Measurement 24](#_Toc102121712)

[8.5.8 ECG assessment 24](#_Toc102121713)

[9 REPLACING MISSING DATA 24](#_Toc102121714)

[10 INTERIM ANALYSIS 24](#_Toc102121715)

[11 AGREEMENTS 25](#_Toc102121716)

[12 EXAMPLES OF TABLES AND LISTINGS 25](#_Toc102121717)

[12.1 Disposition of research subjects 25](#_Toc102121718)

[12.2 Baseline 25](#_Toc102121719)

[12.2.1 General medical and allergic history 26](#_Toc102121720)

[12.2.2 Prior Therapy and Prior Procedures 26](#_Toc102121721)

[12.2.3 Adverse Events 26](#_Toc102121722)

[12.2.4 Other Baseline Characteristics 27](#_Toc102121723)

[12.2.5 Laboratory indicators 27](#_Toc102121724)

[12.3 Efficacy analysis 28](#_Toc102121725)

[12.3.1 Primary Endpoint 28](#_Toc102121726)

[12.3.2 Secondary Endpoints 29](#_Toc102121727)

[12.4 Safety Analysis 31](#_Toc102121728)

[12.4.1 Duration of study drug exposure 31](#_Toc102121729)

[12.4.2 Adverse Events 31](#_Toc102121730)

[12.4.3 Concomitant Therapy and Related Procedures 32](#_Toc102121731)

[12.4.4 Laboratory indicators 33](#_Toc102121732)

[12.4.5 Other Safety Options 34](#_Toc102121733)

[12.5 Listings 37](#_Toc102121734)

[13 REFERENCES TO LITERATURE 46](#_Toc102121735)

[14 LIST OF REVISIONS OF THE STATISTICAL ANALYSIS PLAN 46](#_Toc102121736)

LIST OF TABLES

[Table 4.1 COVID-TRE-03 Study Design 10](#_Toc102121596)

[Table 4.2 Schedule of COVID-TRE-03 procedures 11](#_Toc102121597)

[Table 8.1 Criteria for achieving the primary endpoint by population size in the Treamide and Placebo groups 15](#_Toc102121598)

[Table 8.2 Possible Discrete Data Values for the KBILD Questionnaire 19](#_Toc102121599)

[Table 8.3 Scale . transformation of the initial discrete data of the KBILD questionnaire into scores 20](#_Toc102121600)

[Table 8.4 List of laboratory parameters for blood and urine tests 23](#_Toc102121601)

[Table 12.1 Disposition 25](#_Toc102121602)

[Table 12.2 Demographic and other baseline characteristics. V1/W-2/Screening 25](#_Toc102121603)

[Table 12.3 General medical and allergic history. V1/W-2/Screening 26](#_Toc102121604)

[Table 12.4 Data on the underlying disease (PCR for SARS-COV-2 RNA) 26](#_Toc102121605)

[Table 12.5 Prior Therapy 26](#_Toc102121606)

[Table 12.6 Concomitant Therapies Registered Before ID 26](#_Toc102121607)

[Table 12.7 Prior Procedures Recorded Before Taking ID 26](#_Toc102121608)

[Table 12.8 Concomitant procedures registered before ID intake 26](#_Toc102121609)

[Table 12.9 Adverse events (non-TEAE). 26](#_Toc102121610)

[Table 12.10 Serious adverse events (non-TESAE) 27](#_Toc102121611)

[Table 12.11 Physical examination. Before taking ID 27](#_Toc102121612)

[Table 12.12 Vital Signs and SpO2 Measurement. Before taking ID 27](#_Toc102121613)

[Table 12.13 ECG (12 leads). Before taking ID 27](#_Toc102121614)

[Table 12.14 Complete blood count. Before taking ID 27](#_Toc102121615)

[Table 12.15 Biochemical analysis of blood. Before taking ID 28](#_Toc102121616)

[Table 12.16 Urinalysis. Before taking ID 28](#_Toc102121617)

[Table 12.17 Pregnancy test 28](#_Toc102121618)

[Table 12.18 Frequency of achieving a clinically significant change in FVC and/or DLCO at Week 4 from baseline 28](#_Toc102121619)

[Table 12.19 Frequency of achieving a clinically significant change in FVC and/or DLCO at Week 4 from baseline 28](#_Toc102121620)

[Table 12.20 Mean Change in Distance Walked in 6 Minutes (6MWD) Week 2 and Week 4 from Baseline 29](#_Toc102121621)

[Table 12.21 Mean change in Borg score at Week 2 and Week 4 from baseline (based on the 6 Minute Walk Test) 29](#_Toc102121622)

[Table 12.22 Mean relative change in spirometry lung function parameters (FEV1, FVC, FEV1/FVC) at Week 1, Week 2, Week 3, and Week 4 from baseline 29](#_Toc102121623)

[Table 12.23 Mean relative change in body plethysmography lung function parameters at Week 2 and Week 4 from baseline (DLCO, TLC, and FRC) 30](#_Toc102121624)

[Table 12.24 Rate of reduction in lung injury (improvement) by CT scan at Week 4 from baseline 30](#_Toc102121625)

[Table 12.25 Mean change in severity on the mMRC Breathlessness Scale at Week 1, Week 2, Week 3, and Week 4 from baseline 30](#_Toc102121626)

[Table 12.26 Mean change in total score as well as scores for the 3 domains of the KBILD questionnaire in Week 2 and Week 4 from baseline 30](#_Toc102121627)

[Table 12.27 Exposure assessment 31](#_Toc102121628)

[Table 12.28 Adverse events (TEAE). AE outcome statistics 31](#_Toc102121629)

[Table 12.29 Adverse events (TEAE). total stats 31](#_Toc102121630)

[Table 12.30 Adverse events (TEAE) 32](#_Toc102121631)

[Table 12.31 ID-related adverse events (TEAE) 32](#_Toc102121632)

[Table 12.32 Adverse events (TEAE) by severity and association with ID 32](#_Toc102121633)

[Table 12.33 Serious AEs (TESAE) 32](#_Toc102121634)

[Table 12.34 Serious AEs (TESAE) associated with ID 32](#_Toc102121635)

[Table 12.35 Serious AEs (TESAE) by severity and association with ID 32](#_Toc102121636)

[Table 12.36 Concomitant Therapy (start after ID) 32](#_Toc102121637)

[Table 12.37 Concomitant procedures (beginning after taking the ID) 33](#_Toc102121638)

[Table 12.38 Complete blood count. During the study 33](#_Toc102121639)

[Table 12.39 Complete blood count (absolute values). During the study 33](#_Toc102121640)

[Table 12.40 Biochemical analysis of blood. During the study 33](#_Toc102121641)

[Table 12.41 Biochemical analysis of blood (absolute values). During the study 33](#_Toc102121642)

[Table 12.42 Urinalysis. During the study 34](#_Toc102121643)

[Table 12.43 Pregnancy test 34](#_Toc102121644)

[Table 12.44 Patient Compliance Assessment. During the study 34](#_Toc102121645)

[Table 12.45 Physical examination. During the study 34](#_Toc102121646)

[Table 12.46 Measurement of body weight. During the study 34](#_Toc102121647)

[Table 12.47 Vital Signs and SpO2 Measurement. During the study 35](#_Toc102121648)

[Table 12.48 Vital signs and SpO2 measurements (absolute values). During research 35](#_Toc102121649)

[Table 12.49 ECG (12 leads). During the study 35](#_Toc102121650)

[Table 12.50 ECG (12 leads) (absolute values). During the study 36](#_Toc102121651)

**LISTING LIST**

[Listing 1 Demographics and Other Background Characteristics 37](#_Toc102121652)

[Listing 2 Dates of actual visits 37](#_Toc102121653)

[Listing 3 Distribution of subjects by population 37](#_Toc102121654)

[Listing 4 Protocol deviations 37](#_Toc102121655)

[Listing 5 Early termination 38](#_Toc102121656)

[Listing 6 Exposure evaluation 38](#_Toc102121657)

[Listing 7 Novel coronavirus infection (COVID-19) 38](#_Toc102121658)

[Listing 8 Medical history 38](#_Toc102121659)

[Listing 9 Prior and concomitant therapy 39](#_Toc102121660)

[Listing 10 Preceding and related procedures 39](#_Toc102121661)

[Listing 11 Adverse events 40](#_Toc102121662)

[Listing 12 Physical examination 40](#_Toc102121663)

[Listing 13 Vital signs and measurement of blood oxygen saturation 41](#_Toc102121664)

[Listing 14 PCR for SARS-COV-2 RNA 41](#_Toc102121665)

[Listing 15 Lab examination. - Complete Blood Count 41](#_Toc102121666)

[Listing 16 Laboratory examination. Blood chemistry; 41](#_Toc102121667)

[Listing 17 Lab examination. General urine analysis 42](#_Toc102121668)

[Listing 18 Pregnancy test 42](#_Toc102121669)

[Listing 19 ECG (12 leads) 42](#_Toc102121670)

[Listing 20 mMRC breathlessness scale 43](#_Toc102121671)

[Listing 21 Chest CT scan 43](#_Toc102121672)

[Listing 22 Spirometry 43](#_Toc102121673)

[Listing 23 Body plethysmography 43](#_Toc102121674)

[Listing 24 6-minute walk test (6MWD, Borg scale) 44](#_Toc102121675)

[Listing 25 KBILD questionnaire 44](#_Toc102121676)

[Listing 26 Body weight (kg) 45](#_Toc102121677)

[Listing 27 Compliance assessment 45](#_Toc102121678)

1. LIST OF ABBREVIATIONS AND DEFINITIONS OF TERMS

| **Parameter** | **Description** |
| --- | --- |
| **6MWD** | 6-min walk distance |
| **COVID-19** | Coronavirus Disease 2019 (acute respiratory infection caused by SARS-CoV-2 coronavirus (2019-nCoV) |
| **DLCO** | Diffusing capacity of the lungs for carbon monoxide |
| **E** | Number of events |
| **FAS** | Full Analysis Set |
| **FEV** | Forced expiratory volume |
| **FEV1** | Forced expiratory volume in 1 second of forced expiratory maneuver |
| **FRC** | Expiratory reserve volume |
| **FVC** | Forced vital capacity |
| **ICH** | International Conference on Harmonization |
| **ICH GCP** | International Conference on Harmonization Good Clinical Practice Guidelines |
| **KBILD** | King's brief interstitial lung disease scoring questionnaire |
| **MedDRA** | Medical Dictionary for Regulatory Activities |
| **mMRC** | Shortness of breath scale (modified medical research council) |
| **n** | Number of non-zero values of the estimated parameter |
| **N** | Number of Subjects in a Population by Treatment Group |
| **PPS** | Per Protocol Set |
| **PT** | Preferred Term |
| **RAN** | Randomized Set |
| **SAF** | Safety Set |
| **SOC** | System Organ Class |
| **SpO2** | Saturation (saturation of blood with oxygen) |
| **TEAE** | Treatment Emergent Adverse Events |
| **TLC** | Total lung capacity |
| **ATC** | Anatomical Therapeutic Chemical |
| **ALT** | alanine aminotransferase |
| **AST** | aspartate aminotransferase |
| **V** | Visit |
| **GGT** | Gamma-glutamyl transpeptidase |
| **DBP** | diastolic blood pressure |
| **ET** | Early termination |
| **BMI** | Body Mass Index = Body weight (kg) / Height^2^ (m^2^) |
| **SD** | Study Drug |
| **CRF** | Case report form |
| **IC** | Informed consent |
| **CBD** | Clinical Database |
| **CS** | Clinically significant deviation |
| **CI** | Clinically insignificant deviation |
| **KT** | CT scan |
| **CPK** | Creatine phosphokinase |
| **max** | Maximum value |
| **min** | Minimum value |
| **N (Primary State Registration Number)** | Week |
| **AE** | Adverse event |
| **ETer** | End of therapy |
| **PCR** | Polymerase chain reaction |
| **RNA** | Ribonucleic acid |
| **SBP** | systolic blood pressure |
| **GFR** | Glomerular filtration rate |
| **SAE** | Serious adverse event |
| **CO** | Standard deviation |
| **SOP** | Standard Operating Procedures |
| **Mean** | Arithmetic mean |
| **FC** | Pharmacokinetics |
| **ALP** | Alkaline phosphatase |
| **RR** | Respiratory rate |
| **eCRF** | Electronic Case Report Form |
| **ECG** | Electrocardiogram/electrocardiography |

1. INTRODUCTION

This document describes the planned statistical analysis of data from a clinical trial according to the COVID-TRE-03 protocol dated July 20, 2020 with amendment 1 dated September 18, 2020, sponsored by PHARMENTERPRICES LLC.

1. GOALS AND OBJECTIVES OF THE RESEARCH
   1. Primary objective

The primary objective of this study is to evaluate the efficacy of Treamide versus placebo in patients with a history of COVID-19 pneumonia based on the incidence of a clinically meaningful change in forced vital capacity (FVC) and/or diffusing capacity of the lungs (DLCO) at Week 4. relative to the original values.

- 1. Additional objectives

An additional study objective is to evaluate the efficacy and safety of Treamide versus placebo in patients with COVID-19 pneumonia based on the following parameters:

- Change in distance traveled in 6 minutes (6MWD) in Week 2 and Week 4 from baseline (based on the 6 Minute Walk Test);
- Change in Borg score at Week 2 and Week 4 from baseline (based on the 6 Minute Walk Test);
- Relative change in spirometry lung function parameters (forced expiratory volume in one second (FEV1), FVC, FEV1/FVC) at Week 1, Week 2, Week 3, and Week 4 from baseline;
- Relative change in body plethysmography lung function parameters at Week 2 and Week 4 from baseline (DLCO, total lung capacity (TLC), expiratory reserve volume (FRC));
- Change in the degree of lung damage according to computed tomography (CT) at Week 4 from baseline;
- Change in severity on the mMRC Breathlessness Scale at Week 1, Week 2, Week 3, and Week 4 from baseline;
- Change in total score (total score on the scale) on the King Questionnaire for Interstitial Lung Disease (KBILD) at Week 2 and Week 4 from baseline;
- Residual concentration Ctrough of the active substance of the drug Treamide (XC268BG);
- The frequency of adverse events (AE) and serious adverse events (SAE) of varying severity according to subjective complaints, physical examination, vital signs, laboratory tests and electrocardiography (ECG).

1. Study Design

This clinical trial is a multicenter, randomized, double-blind, placebo-controlled phase II pilot study to evaluate the efficacy and safety of Treamide in the rehabilitation of patients after COVID-19 pneumonia.

It was planned that the study would be conducted in approximately 6-10 Russian clinical centers.

The study was planned to include 60 patients. Considering a possible 43% dropout at the study selection stage, up to 90 patients with fibrotic changes in the lungs after suffering from COVID-19 pneumonia will be screened.

The study consists of the following periods: screening, study therapy period, pharmacokinetic (PK) study, and follow-up period.

***Screening***

At the Week -2 visit, following the signing of the Patient Information Sheet and the informed consent form, screening procedures are performed to assess inclusion/non-inclusion criteria. Screening procedures include collection of demographic data, medical history and concomitant therapy, physical examination, measurement of height, body weight and determination of BMI, assessment of vital signs and SpO2, ECG, mMRC dyspnea score, clinical and biochemical blood tests, urinalysis. Women of childbearing potential are given a pregnancy test.

All patients undergo a qualitative determination of SARS-CoV-2 RNA by polymerase chain reaction (PCR). The material for laboratory testing is a swab from the nasopharynx and / or oropharynx. Confirmation of two consecutive negative PCR results at least 24 hours apart is required for inclusion in the study. In the case of previously confirmed elimination of the SARS-CoV-2 virus according to medical records, one determination of SARS-CoV-2 RNA by PCR with a negative result is sufficient.

Patients are also assessed by the mMRC Breathlessness Scale, chest CT, spirometry and body plethysmography with the definition of DLCO. These examinations are carried out as close as possible to the planned date of randomization (not earlier than 5 days before it).

**In the event that screening procedures are initiated as part of a patient's discharge from the hospital following hospitalization of a patient diagnosed with COVID-19 pneumonia, sufficient time should have been allowed for the completion of quarantine measures (about 2 weeks) before assessing the extent of damage and lung function.**

**The screening could use the results of examinations conducted as part of routine medical practice before obtaining informed consent (taking into account the window of the screening visit), except for the assessment according to the mMRC Breathlessness Scale, CT of the chest, spirometry and body plethysmography with the determination of DLCO - these examinations should be carried out as much as possible close to the planned date of randomization (no earlier than 5 days before it).**

Patients meeting all eligibility criteria were included in the study.

***Study therapy period***

At visit Week 0 prior to study therapy, registration of AEs and concomitant therapy, physical examination, measurement of body weight, assessment of vital signs and SpO2, mMRC dyspnea score, ECG, clinical and biochemical blood tests, and urinalysis. Women of childbearing potential are given a pregnancy test. Patients complete the KBILD questionnaire, followed by the 6 Minute Walk Test, which measures the distance the patient walks in 6 minutes and is assessed using the Borg Scale.

After completing all the necessary visit procedures, patients are randomized into two groups in a 1:1 ratio.

Group 1 Treamide 50 mg - 30 patients

Group 2 Placebo - 30 patients

Patients are given the required amount of study drug until the next visit, according to the therapy group.

The duration of study therapy was 4 weeks. Patients are recommended to continue the standard program of medical rehabilitation in a day hospital or outpatient setting (Stage 3 according to the Interim Guidelines "Medical Rehabilitation for Novel Coronavirus Infection (COVID-19)", Version 2 dated July 31, 2020 or current at the time of the study).

During the study therapy phase, patients visit the study center once a week. At Week 1, Week 2, and Week 3 visits, registration of AEs and concomitant therapy, study medication, body weight, vital signs and SpO2, mMRC dyspnea score, and spirometry are performed.

At visit Week 2, physical examination, ECG, clinical and biochemical blood tests, PK study, urinalysis, body plethysmography with DLCO are also performed. Patients complete the KBILD questionnaire. There is also the 6 Minute Walk Test, which measures the distance the patient walks in 6 minutes and is assessed using the Borg Scale. Women of childbearing potential are given a pregnancy test.

At visit Week 4 (end of therapy), registration of AEs and concomitant therapy, registration of the study drug, physical examination, measurement of body weight, assessment of vital signs and SpO2, ECG, clinical and biochemical blood tests, PK study, urinalysis are performed. Patients are assessed according to the mMRC Breathlessness Scale, chest CT, spirometry and body plethysmography with the definition of DLCO. Patients complete the KBILD questionnaire, followed by the 6 Minute Walk Test, which measures the distance the patient walks in 6 minutes and is assessed using the Borg Scale. After completion of study therapy, patients continue treatment according to general guidelines. Women of childbearing potential are given a pregnancy test.

***PK study***

Blood sampling for the Ctrough PK study is performed on all patients prior to investigational drug (ID) administration at Week 0, Week 2, and Week 4.

***Follow-up period.***

Patient follow-up continues for two more weeks. At Week 6, registration of AEs and concomitant therapy, physical examination, measurement of body weight, assessment of vital signs and SpO2, mMRC dyspnea score, ECG, clinical and biochemical blood tests, and urinalysis are performed. Women of childbearing potential are given a pregnancy test.

Below is the Study Scheme and Schedule of Procedures:

Table 4.1 COVID-TRE-03 Study Design

|  | Screening | Investigational Therapy | | | | Follow-up |  |
| --- | --- | --- | --- | --- | --- | --- | --- |
|  |  |  | | | |  |  |
|  | ~90patients | Treamide 50 mg (n=30) | | | |  |  |
|  |  | Placebo (n=30) | | | |  |  |
|  |  |  |  |  |  |  |  |
| Visit | V 1 | V 2 | V 3 | V 4 | V 5 | V 6 | V 7 |
| Week | W-2 | W0 | W1 | W2 | W3 | W4 | W6 |

Table 4.2 Schedule of COVID-TRE-03 procedures

| **Procedures** | **Screening**[[16]](#footnote-16) | **Investigational Therapy** | | | | | **Follow-up** | **E/T** |
| --- | --- | --- | --- | --- | --- | --- | --- | --- |
| Visit (V) | V 1 | V 2 | V 3 | V 4 | V 5 | V 6 | V 7 |  |
| Week (W) | W-2 | W0 | W1 | W2 | W3 | W4 (ET) | W6 |  |
| Visit/procedures window | Day -28.0**[[17]](#footnote-17)** |  | ± 1 day | ± 2 days | ± 1 day | ± 2 days | ± 3 days |  |
| Informed consent | X |  |  |  |  |  |  |  |
| Demographic data | X |  |  |  |  |  |  |  |
| Medical history (including history of COVID-19) | X |  |  |  |  |  |  |  |
| Physical examination | X | X |  | X |  | X | X | X |
| Body weight, height, BMI[[18]](#footnote-18) | X | X | X | X | X | X | X | X |
| Vital signs, SpO2 | X | X | X | X | X | X | X | X |
| SARS-CoV-2 RNA by PCR**[[19]](#footnote-19)** | X |  |  |  |  | Х |  |  |
| Complete Blood Count[[20]](#footnote-20) | X | X |  | X |  | X | X | X |
| Blood chemistry; | X | X |  | X |  | X | X | X |
| PK study**[[21]](#footnote-21)** |  | Х |  | Х |  | Х |  |  |
| General urine analysis | X | X |  | X |  | X | X | X |
| Pregnancy test**[[22]](#footnote-22)** | X | X |  | X |  | X | X | X |
| 12-lead ECG | X | X |  | X |  | X | X | X |
| mMRC breathlessness scale | Х | X | X | X | X | X | X | X |
| 6 Minute Walk Test (6MWD, Borg Scale) |  | X |  | X |  | X |  | X |
| Questionnaire KBILD |  | X |  | X |  | X |  | X |
| chest CT | X |  |  |  |  | X |  | X |
| Spirometry (FEV1, FVC, FEV1/FVC) | X | X | X | X | X | X |  | X |
| Body plethysmography (DLCO, TLC, FRC) | X |  |  | X |  | X |  | X |
| Evaluation of inclusion / non-inclusion criteria | X | X |  |  |  |  |  |  |
| Randomization |  | X |  |  |  |  |  |  |
| Distribution of the study drug |  | X | X | X | X |  |  |  |
| Study drug return and compliance assessment |  |  | X | X | X | X |  | X |
| Concomitant therapy | X | X | X | X | X | X | X | X |
| Adverse Events | X | X | X | X | X | X | X | X |

Abbreviations: W - week, D - day, ET - end of therapy, ETe - early termination.

- 1. Randomization

The study uses a 1:1 distribution into two groups using the IWRS system based on the principle of randomization with stratification by:

- presence or absence of risk factors (age ≥ 60 years and/or presence of concomitant chronic diseases)
- and the initial degree of lung damage according to CT (CT 1, CT 2, CT 3 and CT 4).

The stratification method will be described in more detail in the randomization protocol.

- 1. Blinding

This study is double-blind. This means that clinical staff and patients will not know what is in the individual vial. Blinding is provided by placebo masking (each patient taking tablets corresponding to Treamide 50 mg or placebo) and dispensing the drug by IWRS by individual package number.

1. SAMPLE SIZING

The sample size was determined in accordance with the methodology of one-stage non-comparative design of phase II clinical trials (RP A’Hern, 2001).

At the moment, the process of recovery of lung function in patients who have undergone COVID-19 is not well understood. In this study, 25% of patients in the Placebo group and 50% of patients in the Treamide group are expected to achieve a clinically significant change in FVC and/or DLCO at Week 4 from baseline. For α = 0.05 (one-sided), power 80%, p_0_ =25%, and p_1_ =50%, 26 patients must be included in the analysis to test the hypothesis. 11 or more answers in 26 included patients will allow us to reject the null hypothesis (_W0_: p ≤ p_0_ ) in favor of the alternative one (_W1_: p ≥ p_1_) and thus make a positive decision regarding the possibility of further study of Treamide in phase III.

Taking into account the possible early withdrawal (impossibility of evaluating the primary endpoint), not exceeding 13%, 30 patients will be included in each group of the study therapy. Thus, 60 patients are planned to be randomized into the study.

1. POPULATIONS ANALYZED
   1. Population of all randomized subjects

All patients randomized to the study will be included in the population of all randomized subjects (Randomized Set, RAN).

- 1. Safety Population

All subjects who received at least one dose of study drug will be included in the Safety Set (SAF) population.

- 1. Efficacy population

The Full Analysis Set (FAS) is defined as all randomized patients who received at least one dose of study drug and have at least one post-baseline efficacy rating. The FAS population is the main population for efficacy analysis.

- 1. Population per protocol

The Per Protocol Set (PPS) corresponds to patients in the entire analysis population who will receive the study therapy in full and will not have significant deviations from the protocol.

Deviations from the protocol will be monitored by the clinical team throughout the duration of the study. A complete list of protocol deviations will be summarized and reviewed to identify major and minor violations before the database is closed and statistical analysis begins. The main violations are: non-compliance with the study protocol and / or study procedures, which may affect the safety of the study subject and the integrity of the data.

All deviations will be summarized and presented as a listing. The reasons for exclusion from the analyzed populations will also be summarized and presented in the form of a listing.

- 1. Pharmacokinetics population

All patients who have received at least one dose of study drug and for whom sufficient PK samples have been obtained to evaluate at least one PK parameter will be included in the PK population.

1. EFFICACY, SAFETY AND PHARMACOKINETICS VARIABLES
   1. Primary Efficacy Variable

The primary efficacy variable is:

- Frequency of achieving a clinically significant change in FVC and/or DLCO at Week 4 from baseline.

Clinically significant changes are considered to be a relative increase in FVC by ≥10% or a relative increase in FVC in the range from ≥5% to <10% and a relative increase in DLCO by ≥15%.

- 1. Secondary Efficacy Variables

Secondary efficacy variables include:

- Change in distance traveled in 6 minutes (6MWD) in Week 2 and Week 4 from baseline (based on the 6 Minute Walk Test);
- Change in Borg score at Week 2 and Week 4 from baseline (based on the 6 Minute Walk Test);
- Relative change in spirometry lung function parameters (forced expiratory volume in one second (FEV1), FVC, FEV1/FVC) at Week 1, Week 2, Week 3, and Week 4 from baseline;
- Relative change in body plethysmography lung function parameters at Week 2 and Week 4 from baseline (DLCO, total lung capacity (TLC), expiratory reserve volume (FRC));
- Change in the degree of lung damage according to computed tomography (CT) at Week 4 from baseline;
- Change in severity on the mMRC Breathlessness Scale at Week 1, Week 2, Week 3, and Week 4 from baseline;
- Change in total score (total score on the scale) on the King's Interstitial Lung Disease Questionnaire (KBILD) at Week 2 and Week 4 from baseline.
  1. Safety Variables

The safety of the investigational product will be assessed based on the incidence and severity of AEs and SAEs assigned to the TEAE group of varying severity based on subjective complaints.

Safety will also be assessed based on ECG results, laboratory tests and subjects' vital signs, as well as a physical examination.

1. STATISTICAL METHODS AND STATISTICAL DATA ANALYSIS

All performance and safety indicators will be presented by descriptive statistics:

continuous data will be represented by the number of non-missing values, mean with standard deviation, median, minimum and maximum values. For continuous data required for performance analysis, in addition to the statistics described above, Q1 and Q3 quartiles will also be calculated.

nominal/discrete data will be represented by absolute and relative (percentage) frequencies.

Intra- and between-group comparisons, calculation of confidence intervals and p-values will be described for efficacy and safety variables separately (if applicable).

Data analysis will be carried out using specialized software SAS ® version 9.4 in accordance with SOP-DM-15.01 and SOP-DM-16.01 of the IPHARMA LLC company.

- 1. Disposition

*Source:* Table 12.1

The number of subjects screened by absolute frequency, the number of subjects randomized (enrolled in the study), the number of subjects in all randomized (RAN), safety (SAF), complete population for analysis (FAS) and per protocol (PPS) populations, and the number completed the study subjects by therapy groups ("Treamide" and "Placebo") using absolute and relative frequencies.

Additionally, for each subject, a listing with the dates of all scheduled visits and a listing with the data of all subjects who dropped out of the study ahead of schedule will be presented. All listings (hereinafter) will be presented in the population of all randomized subjects (RAN) in order to more fully reflect the information on the study.

- 1. Demographic and other background characteristics

*Source:* Table 12.2 *,* Table 12.3 *,* Table 12.4 *,* Table 12.5 *,* Table 12.6 *,* Table 12.7 *,* Table 12.8 *,* Table 12.9 *,* Table 12.10 *,* Table 12.11 *,* Table 12.12 *,* Table 12.13 *,* Table 12.14 *,* Table 12.15 *,* Table *12.16*

This section will analyze all parameters collected at the V1/W-2/Screening and V1/W0 visits assessed prior to the first ID dose. The exception will be the absolute values of parameters and scales that are assessed not only at the initial level (before taking the first dose of ID), but also further in the study. Such parameters and scales include physical examination, vital signs, body weight measurement, etc. All of these parameters, as absolute values, will be described in section 8.5 SAFETY ANALYSIS along with the rest of the data collected after the first dose of PI.

All baseline characteristics will be presented in the **Full Analysis Population (FAS)**.

Demographic characteristics will be described for Treamide and Placebo treatment groups. The Treamide therapy group will include, according to the “intention to treat” principle, all patients randomized to the Treamide drug group, and the Placebo group will include patients randomized to the Placebo drug group.

The section will present, using descriptive statistics on therapy groups, the following data of study subjects:

- Age
- Floor:
- Race
- Height, cm
- body weight (kg) (data collected at the Screening/W-2 visit only);
- BMI (body mass index) (kg/ m^2^);
- general medical and allergic history;
- data on the underlying disease (PCR for SARS-COV-2 RNA)**[[23]](#footnote-23)** ;
- prior and concomitant therapies recorded prior to PI administration;
- previous and concomitant procedures registered before taking the PI;
- AEs and SAEs before the first dose of PI (not TEAE);
- physical examination results**[[24]](#footnote-24)** ;
- vital signs and SpO2 measurements**[[25]](#footnote-25)**;
- ECG (12 leads);
- clinical and laboratory parameters (complete blood count, biochemical blood test, urinalysis)**[[26]](#footnote-26)** ;
- pregnancy test (women only)**[[27]](#footnote-27)** .

Age, height, weight, BMI will be reported by treatment group as continuous data: number of observations, mean with standard deviation, median, and maximum and minimum values.

Other parameters: gender, race, underlying disease data, physical examination results, vital signs and SpO2 measurements, ECG (12-lead), pregnancy test results and laboratory values (CBC, blood chemistry and urine analysis) will be presented by treatment group as nominal data with absolute and relative frequencies.

History, previous and concomitant procedures, and adverse events that began before ID will be coded according to MedDRA (version 23 RUS) and presented by System Organ Class (SOC) therapy groups and preferred medical terms (Preferred Term - PT).

Data on parameters such as concomitant therapy and adverse events will be described in this section if they are recorded at the screening stage (V1/W-2) and at the stage of investigational therapy (V2/W0) (provided that they were started before taking the ID). More detailed analysis of adverse events and concomitant therapy is described in section 8.5 "SAFETY ANALYSIS".

Prior and concomitant therapies recorded in study subjects prior to the first ID dose will be coded and tabulated as absolute and relative frequencies by therapeutic (ATC code level 2) and chemical subgroups (ATC code level 4); the corresponding ATC codes will be listed there. Also, the information will be presented in the form of a listing, where, in addition to the above groups, the name of therapy, dose, units of measurement, frequency of administration, route of administration, start and end dates of administration, concomitant therapy was taken before or after the first dose of PI and indication, and also, the case applies to prior or concomitant therapy.

Additionally, a listing will be provided for each subject containing individual data by race, gender, date of birth, age, body weight, height, BMI, treatment group, and date the informed consent form was signed. Listings will also be provided for other parameters analyzed in this section. All listings will be presented in **the population of all randomized patients (RAN)** in order to more fully and fully reflect information on the study.

- 1. Assessment of treatment compliance (compliance)

Source: Table 12.44

The first dose of study drug will be taken by the patient at the study site under the supervision of the Investigator (V2/W0). Compliance with the recommendations for self-administration of investigational drug will be verified by the Investigator at visits based on the results of accounting for the returned investigational drug.

Compliance calculation will be carried out on visits according to the formula (1):

| Compliance = | *Ν*_given_ - *N* _returned_ | х 100% |  | (1), |
| --- | --- | --- | --- | --- |
|  | *N*_est_. |  |  |  |

where *Ν*_given_ - the number of tablets given,

*N*_returened_ - the number of returned tablets,

*N*_est_. - the estimated number of tablets that the patient should have taken since the previous visit.

Patient compliance should be between 80% and 120%. In this case, the patient is considered compliant. Otherwise, the patient will be marked as non-compliant at the study visit.

Compliance assessment data will be presented in the form of a listing (absolute and nominal values) and a table using descriptive statistics (nominal (compliant/non-compliant/study not performed) values only).

Compliance assessment is carried out at V7/W1, V4/W2, V5/W3, V6/W4/ET visits and, if applicable, at the early termination visit, and therefore will be described in Section 8.5 SAFETY ANALYSIS.

- 1. EFFICACY ANALYSIS

This is a Phase II study to evaluate the efficacy of Treamide versus Placebo in patients with COVID-19 pneumonia based on the frequency of achieving a clinically significant change in FVC and/or DLCO at Week 4 from baseline.

Clinically significant changes are considered to be a relative increase in FVC by ≥10% or a relative increase in FVC in the range from ≥5% to <10% and a relative increase in DLCO by ≥15%.

Secondary efficacy points will be analyzed using statistical tests at all visits after baseline visits (V1/W-2/Screening and/or V2/W0). However, the data obtained at the early termination visit (E/T) will not be analyzed using statistical criteria due to too small sample size for their application.

- - 1. Primary Efficacy Variable

The primary efficacy endpoint (primary efficacy assay) will be analyzed in the **complete assay population (FAS)** and also, to test the sensitivity of the assay, in **the protocol population (PP).**

- **Frequency of achieving a clinically significant change in FVC and/or DLCO at Week 4 from baseline:**

*Source:* Table 12.18, Table 12.19

Clinically significant changes are considered to be a relative increase in FVC by ≥10% or a relative increase in FVC in the range from ≥5% to <10% and a relative increase in DLCO by >15%.

The analysis of the primary endpoint in each group will be carried out in accordance with the algorithm OF RP A'Hern for single-stage phase II studies with a one-sided α=0.05 and a power of 80%, according to which the sample size was calculated (see section 5 Determining the sample size) depending on the number of patients included in the analyzed population (see See Table 8.1).

Table 8.1 Criteria for achieving the primary endpoint by population size in the Treamide and Placebo groups

| Number of patients, Treamide group | Expected response probability, Treamide group | Response rate corresponding to PEP achievement, Treamide group | Number of patients, placebo group | Response rate corresponding to PEP achievement, placebo group |
| --- | --- | --- | --- | --- |
| 26 and under | ≥50% | ≥11 | 25-27 | <11 |
|  |  |  | 28 or 29 | <12 |
|  |  |  | 30 | <13 |
| 27 | ≥48% | ≥11 | 25-27 | <11 |
|  |  |  | 28 or 29 | <12 |
|  |  |  | 30 | <13 |
| 28 | ≥50% | ≥12 | 25-27 | <11 |
|  |  |  | 28 or 29 | <12 |
|  |  |  | 30 | <13 |
| 29 | ≥48% | ≥12 | 25-27 | <11 |
|  |  |  | 28 or 29 | <12 |
|  |  |  | 30 | <13 |
| 30 | ≥50% | ≥13 | 25-27 | <11 |
|  |  |  | 28 or 29 | <12 |
|  |  |  | 30 | <13 |

In the event that the number of subjects who achieve a clinically significant change in FVC and/or DLCO at Week 4 from baseline satisfies the criteria in Table 8.1, a decision will be made to achieve PEP and further study of this dose of the drug in phase III. The decision on the achievement of the PEP will be made if:

- with a Treamide group size of 27 or less, 11 or more of them will achieve a clinically significant change in FVC and / or DLCO;

- with a Treamide group size of 28 or 29 subjects, 12 of them or more will achieve a clinically significant change in FVC and / or DLCO;

- with a Treamide group size of 30 subjects, 13 or more of them will achieve a clinically significant change in FVC and / or DLCO.

However, if the response rate in the placebo group exceeds the expected response rate shown in Table 8.1, then the PEP assessment will be deferred to an earlier study visit. Those. PEP evaluation will be rescheduled to an earlier study visit if:

- with a placebo group size of 27 or less, 11 or more of them will achieve a clinically significant change in FVC and / or DLCO;

- with a placebo group size of 28 or 29 subjects, 12 of them or more will achieve a clinically significant change in FVC and / or DLCO;

- with a placebo group size of 30 subjects, 13 or more of them will achieve a clinically significant change in FVC and / or DLCO.

For these purposes, the FVC parameter will be taken from the data collected at the V5/W3 visit, and the DLCO parameter from the V4/W2 visit. Thus, the assessment as a whole will be carried out at visit V5/W3, however, if necessary, data on the DLCO parameter will be taken from the previous visit (V4/W2). If the number of responses to therapy in the Treamide group of 26 included patients is 10 or less (out of 27 - 10 or less, out of 28 or 29 - 11 or less, out of 30 - 12 or less), it will be concluded that there is no sufficient efficacy according to the described end point.

The response to therapy will be presented in the tables with absolute and relative frequencies for Treamide and Placebo groups. Additionally, information will be provided on the relative change in the percentage of due [1] parameters at the assessed visit compared to baseline (V1/W-2/Screening for FVC and DLCO) as nominal values for the FVC parameter:

The absence of any changes

Relative decrease in parameter values

Relative increase of <5%/

Relative increase in the range from ≥5% to <10%/

Relative increase of ≥10%

and for the DLCO parameter

The absence of any changes

Relative decrease in parameter values

Relative increase of <15%

Relative increase of ≥15%

using absolute and relative frequencies by therapy group.

The relative change in the result of the parameter at the evaluated visit compared to the baseline will be calculated using the formula (2):

| Δ = | α_x_- α_1_ | х 100% |  |  | (2), |
| --- | --- | --- | --- | --- | --- |
|  | α_1_ |  |  |  |  |

where

α _x_ - calculated "% of due" parameter at the evaluated visit;

α _1_ - baseline "% predicted" parameter at visit V1/W-2/Screening for FVC and DLCO;

Δ - the change in the “percentage of due” (result) at the evaluated visit compared to the initial value, expressed as a percentage.

In the event that there is no data for the subject at the evaluation visit, the subject will be declared a non-responder, so no data will be replaced.

Additionally, frequencies will be compared between the Treamide and Placebo groups using the ^χ2^ test or Fisher's exact test (depending on whether there is at least one value less than 5 in the cells of the 2x2 table of expected values).

- - 1. Secondary Efficacy Variables

All secondary efficacy endpoints will be analyzed in the **complete analysis population (FAS)**.

- **Average change in distance walked in 6 minutes (6MWD) in Week 2 and Week 4 from baseline (based on the 6 Minute Walk Test):**

*Source:* Table 12.20

The results of the measurement of the distance covered in 6 minutes will be presented in a table with continuous data on therapy groups and assessed visits.

Data analysis will be presented using descriptive statistics (see paragraph 8). Also, descriptive statistics will be calculated for the change in parameter at visits V4/W2 and V6/W4/ET from baseline (V2/W0, before ID).

In addition to the fact that the data will be presented in the form of tables, they will also be presented by the listing of the evaluated parameters in the form of discrete values grouped by therapy group, subject and visit.

An assessment of the statistical significance of differences in the mean change in distance between the two groups at visits V4/W2 and V6/W4/ET compared to baseline (V2/W0, pre-ID) will be performed using an unpaired Student's T-test for data having a normal distribution, or using the non-parametric Mann-Whitney U-test with a non-normal distribution. The variances of samples that have a normal distribution will be tested for homogeneity using the Levene test. In the absence of homogeneity of the variances, the unpaired Student's T-test will be supplemented by the Welch correction. No intragroup comparisons are planned.

- **Average change in Borg score at Week 2 and Week 4 from baseline (based on the 6 Minute Walk Test):**

*Source:* Table 12.21

The results of the Borg score will be presented in the table as continuous (after recoding the discrete responses of patients) data for treatment groups and assessed visits. Discrete data will be recoded as follows:

- No shortness of breath at all = 0 points
- Very, very light (almost imperceptible) = 0.5 points
- Very mild shortness of breath = 1 point
- Mild (weak) dyspnea = 2 points
- Average dyspnea = 3 points
- Moderate shortness of breath = 4 points
- Severe dyspnea = 5 points
- Fairly severe dyspnea = 6 points
- Very severe dyspnea = 7 points
- Very, very severe shortness of breath = 8 points
- Extremely severe dyspnea = 9 points
- Unbearable (extremely severe) shortness of breath = 10 points

The data recoded into scores will be converted for analysis as follows according to formula (3):

| A = Result _after testing_ - Result _before testing_ | (3), |
| --- | --- |

where A - the analyzed variable;

Result before testing - the degree of respiratory discomfort and general fatigue before starting the 6-minute walk test;

Result after testing - the degree of respiratory discomfort and general fatigue at the end of testing.

An analysis of the data obtained from the evaluated visits (transformed using formula (3)) will be presented using descriptive statistics (mean, SD, median, minimum and maximum). Also, descriptive statistics will be calculated for the change in parameter at visits V4/W2 and V6/W4/ET from baseline (V2/W0, before ID).

In addition to the fact that the data will be presented in the form of tables, they will also be presented by the listing of the evaluated parameters in the form of discrete values grouped by therapy group, subject and visit.

Evaluation of the statistical significance of differences in the mean change in the Borg score between the two groups at visits V4/W2 and V6/W4/ET from baseline (V2/W0, before ID) will be performed using an unpaired Student's t-test for data, having a normal distribution, or using the non-parametric Mann-Whitney U-test with a non-normal distribution. The variances of samples that have a normal distribution will be tested for homogeneity using the Levene test. In the absence of homogeneity of the variances, the unpaired Student's T-test will be supplemented by the Welch correction. No intragroup comparisons are planned.

- **Mean relative change in spirometry lung function parameters** **(FEV1, FVC, FEV1/FVC) at Week 1, Week 2, Week 3, and Week 4 from baseline:**

*Source:* Table 12.22

The results of measuring the "% of predicted" characteristic of the spirometry parameters (FEV1, FVC and FEV1/FVC) will be presented in a table with continuous data by therapy group and assessed visits.

The relative change in the result of the parameters at the evaluated visit compared to the baseline will be calculated using the formula (4):

| Δ= | *а_х_* - *а_1_* | х 100% |  | (4), |
| --- | --- | --- | --- | --- |
|  | *а_1_* |  |  |  |

where

*а* *_x_* - the calculated “% of due” parameter at the evaluated visit;

*a* *_1_* — baseline “% of predicted” parameter at visit V1/W-2/Screening;

Δ is the change in the “percentage of due” (result) at the evaluated visit compared to the initial value, expressed as a percentage.

Data analysis will be presented using descriptive statistics (see paragraph 8). Also, descriptive statistics will be calculated for parameter change at V1/W0, V3/W1, V4/W2, V5/W3 and V6/W4/ET visits from baseline (V1/W-2/Screening).

In addition to the fact that the data will be presented in the form of tables, they will also be presented in the form of a listing of the evaluated parameters in the form of discrete values grouped by therapy group, subject and visit, as well as with a calculated relative change in the parameter at the evaluated visit from baseline.

Assessment of the statistical significance of differences in the average relative change in lung function parameters between the two groups at visits V2/W0, V3/W1, V4/W2, V5/W3 and V6/W4/ET compared to baseline (V1/W-2/Screening) will be performed using an unpaired Student's T-test for data with a normal distribution, or using a non-parametric Mann-Whitney U-test for non-normally distributed data. The variances of samples that have a normal distribution will be tested for homogeneity using the Levene test. If there is no homogeneity of the variances, the unpaired Student's T-test will be supplemented by the Welch correction. No intragroup comparisons are planned.

- **Mean relative change in body plethysmography lung function parameters at Week 2 and Week 4 from baseline (DLCO, TLC, and FRC):**

*Source:* Table 12.23

The results of measuring the "% of predicted" characteristic of body plethysmography parameters ( DLCO, TLC and FRC) will be presented in a table with continuous data by treatment groups and assessed visits.

The relative change in the result of the parameters at the evaluated visit compared to the baseline will be calculated using the formula (5):

| Δ= | *а_x_* - *а_1_* | х 100% |  | (5), |
| --- | --- | --- | --- | --- |
|  | *а_1_* |  |  |  |

where

*and* *_x_* - the calculated "% of due" parameter on the evaluated visit;

*a* *_1_* — baseline “% of predicted” parameter at visit V1/W-2/Screening;

Δ is the change in the “percentage of due” (result) at the evaluated visit compared to the initial value, expressed as a percentage.

Data analysis will be presented using descriptive statistics (see paragraph 8). Also, descriptive statistics will be calculated for parameter change at V4/W2 and V6/W4/ET visits from baseline (V1/W-2/Screening).

In addition to the fact that the data will be presented in the form of tables, they will also be presented in the form of a listing of the evaluated parameters in the form of discrete values grouped by therapy group, subject and visit, as well as with a calculated relative change in the parameter at the evaluated visit from baseline.

Evaluation of the statistical significance of the mean relative change in lung function parameters between the two groups at visits V4/W2 and V6/W4/ET from baseline (visit V1/W-2/Screening) will be performed using an unpaired Student's t-test for data , having a normal distribution, or using the non-parametric Mann-Whitney U-test with a distribution other than normal. The variances of samples that have a normal distribution will be tested for homogeneity using the Levene test. In the absence of homogeneity of the variances, the unpaired Student's T-test will be supplemented by the Welch correction. No intragroup comparisons are planned.

- **Rate of reduction in lung injury (improvement) on CT scan at Week 4 from baseline:**

*Source* : Table 12.24

Treatment response/non-response rates will be tabulated with absolute and relative rates.

Response to therapy will be defined as a reduction in lung injury at V6/W4/ET from baseline at V1/W-2/Screening. A decrease in the degree of lung damage is considered to be a CT result less than the initial one by at least 1. For example, if CT 3 was recorded at visit V1/W-2/Screening, then the result of CT 2, CT 1, or CT 0 at visit V6/W4/ will be considered an improvement (response to therapy). FROM.

A non-response to therapy will be considered a result identical to that recorded at baseline or an increase in it by at least 1. For example, if a “CT 3” is recorded at a V1/W-2/Screening visit, a “CT 3” or “CT 4” result at a V6/W4/ET visit will be considered a non-response to therapy.

Evaluation of the statistical significance of differences in the rate of reduction in the degree of lung disease between groups at the V6/W4/ET visit compared to baseline (V1/W-2 visit/Screening) will be performed using Fisher's exact test or χ ^2^ (chi-square) test. Additionally, the data will be presented for each subject as a listing in the form of discrete and continuous values grouped by therapy and visit.

- **Mean change in severity on the mMRC Breathlessness Scale at Week 1, Week 2, Week 3, and Week 4 from baseline** :

*Source:* Table 12.25

The results of the mMRC score will be presented in the table as continuous (after recoding discrete patient responses) data across treatment groups and assessed visits. Discrete data will be recoded as follows:

- No - Shortness of breath is not bothersome, except for very intense exercise = 0 points
- Mild - Shortness of breath when walking fast or climbing a small hill = 1 point
- Moderate - Shortness of breath causes the patient to walk more slowly than other people of the same age, or there is a need to stop while walking at their own pace on a level surface = 2 points
- Severe - Shortness of breath causes the patient to stop when walking for a distance of about 100 m or after a few minutes of walking on a level surface = 3 points
- Very severe - Shortness of breath makes it impossible for the patient to leave their home, or shortness of breath occurs when dressing and undressing = 4 points

An analysis of the data obtained from the evaluation visits will be presented using descriptive statistics (see paragraph 8). Also, descriptive statistics will be calculated for parameter change at visits V3/W1, V4/W2, V5/W3, and V6/W4/ET compared to baseline (V2/W0, before ID).

In addition to the fact that the data will be presented in the form of tables, they will also be presented by the listing of the evaluated parameters in the form of discrete values grouped by therapy group, subject and visit.

An assessment of the statistical significance of differences in the mean change in severity on the mMRC **_** dyspnea score between the two groups at visits V3/W1, V4/W2, V5/W3 and V6/W4/ET compared to baseline (V2/W0, before ID) will be conducted using an unpaired Student's T-test for data with a normal distribution, or using a non-parametric Mann-Whitney U-test for non-normally distributed data. The variances of samples that have a normal distribution will be tested for homogeneity using the Levene test. In the absence of homogeneity of the variances, the unpaired Student's T-test will be supplemented by the Welch correction. No intra-group comparisons planned

- **Mean change in overall KBILD score at Week 2 and Week 4 from baseline:**

*Source* : Table 12.26

The results of the assessment using the KBILD questionnaire [2] will be presented in the table as continuous data for treatment groups and assessed visits.

For each patient, 4 summary scores for each visit will be presented: "sum of scores" for the "dyspnea and daily activities" domain (points 1, 4, 11 and 13), the "chest symptoms" domain (points 2, 7 and 9) , the “psychological symptoms” domain (items 3, 5, 6, 8, 10, 12 and 14) and the overall score on the scale as a whole. Each domain and overall score ranges from 0 to 100 points, with higher scores indicating better health.

The responses to the scale questions will be converted to primary discrete scores in the manner described below (Table 8.2). Translated in accordance with Table 8.2, the primary discrete data will be transformed in accordance with the transformation scale (Table 8.3) to obtain the "sum of points", which will then be analyzed. The transformation was carried out according to the instructions of the King's College Hospital NHS Foundation Trust.

Table 8.2 Possible Discrete Data Values for the KBILD Questionnaire

| **Parameter Answer options Parameter Answer options** | | | |
| --- | --- | --- | --- |
[truncated: 99,150 more chars]
