# Supplementary material for: Efficacy and safety of Treamid in the rehabilitation of patients after COVID-19 pneumonia: a phase 2, randomized, double-blind, placebo-controlled trial
Source: J Transl Med. 2022 Nov 3;20:506. doi: 10.1186/s12967-022-03660-9 (PMC9632561; doi:10.1186/s12967-022-03660-9)
Supplement: Supplementary file 2 — Additional file 2. Supplementary Tables and Figures. [file 12967_2022_3660_MOESM2_ESM.pdf]

**Supplement 2 for**

# Efficacy and Safety of Treamid in the Rehabilitation of Patients After COVID-19 Pneumonia: a phase 2, randomized, double-blind, placebo-controlled trial

*Evgeny Bazdyrev[1], , Maria Panova[2], Maria Brachs[3], Elena Smolyarchuk[4], Daria Tsygankova[1], Liudmila Gofman[5], Yana Abdyusheva[2], Fedor Novikov[2]*

[1] Research Institute for Complex Issues of Cardiovascular Diseases, 6, Sosnoviy Blvd., 650002 Kemerovo, Russia

[2] PHARMENTERPRISES LLC, Skolkovo Innovation Center, Bolshoi Blvd., 42 (1), 143026 Moscow, Russia; m.panova@pharmenterprises.ru (M.P.); fnovikov@pharmenterprises.ru (F.N.); ya.abdyusheva@pharmenterprises.ru (Y.A.);

[3] Treamid Therapeutics GmbH, c/o CoLaborator (Bayer), Building S141, Muellerstr. 178, 13353 Berlin, Germany

[4] I.M. Sechenov First Moscow State Medical University (Sechenov University). Trubetskaya ul. 8, Moscow, 119991 Russia

[5] Kemerovo Regional Clinical Hospital named after S.V. Belyaev, 22, Oktyabskiy pr., 650066, Kemerovo, Russia

# Table of contents

Appendixes ... 3

Appendix 1 Assessment of dyspnea by the mMRC scale (modified medical research council) ... 3

Appendix 2 Modified Borg Dyspnea Scale ... 4

Appendix 3 King's Brief Interstitial Lung Disease Questionnaire (KBILD) ... 5

Appendix 4 Chest computed tomography (CT) ... 7

Appendix 5 Calculation of the percentage of predicted normal values ... 8

Appendix 6 Reasons for patients' exclusion from study populations ... 9

Appendix 7. Eight linear models that considered different variants of interaction between fixed factors ... 10

Appendix 8. Parameters of selected linear models for a change in modified Borg score, TLC and lung damage ... 11

Appendix 9. Results of evaluated adjusted means for selected models for change in modified Borg score, TLC and lung damage ... 12

Appendix 10. Linear models diagnostics for change in modified Borg score, TLC and lung damage ... 13

Appendix 11. Linear models for change in modified dyspnea score including baseline value of lung damage (initCT), DLCO (initDLCO) and 6MWD (init6MWD) as covariate and corresponding Treamid superiority in women according to least-squares means differences between Treamid and placebo groups adjusted to two different baseline values (low and high). ... 14

Appendix 12. Treamid superiority in change in lung damage in women ... 15

Appendix 13. Smoking history for Treamid and placebo groups. ... 16

Appendix 14. Cardiovascular diseases (hypertension) for Treamid and placebo groups. ... 17

Appendix 15. Difference in analyzed parameters change from baseline to week 4 between patients who achieved and did not achieve clinical benefits ... 18

Appendix 16. Exploratory analysis of age, hospitalization time and time after the onset of the first symptoms. ... 20

Appendix 17. Rationale for study drug dose selection based on PK/PD models ... 22

Figures ... 25

Figure 1. Correlation of change in a) lung damage and b) TLC with change in FVC in women. ... 25

Tables ... 26

Table 1. Demographics and baseline clinical characteristics of PPS population by study cohort. ... 26

Table 2. The frequency (n (%)) of clinically significant improvement in DLCO after 4-week treatment period in subgroup of patients with gas exchange impairment at baseline. ... 27

Table 3. Means of baseline values and change for 4 weeks of therapy for ITT population, women, and patients with baseline DLCO < 80%. ... 28

Table 4. List of protocol deviations ... 20

References ... 67

## Appendixes

**Appendix S1** Assessment of dyspnea by the mMRC scale (modified medical research council) [1]

| Grade | Description |
|---|---|
| 0 | I only get breathless with strenuous exercise |
| 1 | I get short of breath when hurrying on level ground or walking up a slight hill |
| 2 | On level ground, I walk slower than people of the same age because of breathlessness, or have to stop for breath when walking at my own pace |
| 3 | I stop for breath after walking about 100 yards or after a few minutes on level ground |
| 4 | I am too breathless to leave the house or I am breathless when dressing/undressing |

**Appendix S2** Modified Borg Dyspnea Scale [2]

| Rating | Shortness of breath |
|---|---|
| 0 | Nothing at all |
| 0.5 | Very, very slight (just noticeable) |
| 1 | Very slight |
| 2 | Slight |
| 3 | Moderate |
| 4 | Somewhat severe |
| 5 | Severe |
| 6 | |
| 7 | Very severe |
| 8 | |
| 9 | Very, very severe (almost maximal) |
| 10 | Maximal |

**Appendix S3** King's Brief Interstitial Lung Disease Questionnaire (KBILD) [3]

This questionnaire is designed to assess the impact of your lung disease on various aspects of your life. Please circle the response that best applies to you for each question.

| **1. In the last 2 weeks, I have been breathless climbing stairs or walking up an incline or hill.** | | | | | | |
|---|---|---|---|---|---|---|
| 1. Every time | 2. Most times | 3. Several Times | 4. Some times | 5. Occasionally | 6. Rarely | 7. Never |
| **2. In the last 2 weeks, because of my lung condition, my chest has felt tight.** | | | | | | |
| 1. All of the time | 2. Most of the time | 3. A good bit of the time | 4. Some of the time | 5. A little of the time | 6. Hardly any of the time | 7. None of the time |
| **3. In the last 2 weeks have you worried about the seriousness of your lung complaint?** | | | | | | |
| 1. All of the time | 2. Most of the time | 3. A good bit of the time | 4. Some of the time | 5. A little of the time | 6. Hardly any of the time | 7. None of the time |
| **4. In the last 2 weeks have you avoided doing things that make you breathless?** | | | | | | |
| 1. All of the time | 2. Most of the time | 3. A good bit of the time | 4. Some of the time | 5. A little of the time | 6. Hardly any of the time | 7. None of the time |
| **5. In the last 2 weeks have you felt in control of your lung condition?** | | | | | | |
| 1. None of the time | 2. Hardly any of the time | 3. A little of the time | 4. Some of the time | 5. A good bit of the time | 6. Most of the time | 7. All of the time |
| **6. In the last 2 weeks, has your lung complaint made you feel fed up or down in the dumps?** | | | | | | |
| 1. All of the time | 2. Most of the time | 3. A good bit of the time | 4. Some of the time | 5. A little of the time | 6. Hardly any of the time | 7. None of the time |
| **7. In the last 2 weeks, I have felt the urge to breathe, also known as 'air hunger'.** | | | | | | |
| 1. All of the time | 2. Most of the time | 3. A good bit of the time | 4. Some of the time | 5. A little of the time | 6. Hardly any of the time | 7. None of the time |
| **8. In the last 2 weeks, my lung condition has made me feel anxious.** | | | | | | |
| 1. All of the time | 2. Most of the time | 3. A good bit of the time | 4. Some of the time | 5. A little of the time | 6. Hardly any of the time | 7. None of the time |
| **9. In the last 2 weeks, how often have you experienced 'wheeze' or whistling sounds from your chest?** | | | | | | |
| 1. All of the time | 2. Most of the time | 3. A good bit of the time | 4. Some of the time | 5. A little of the time | 6. Hardly any of the time | 7. None of the time |
| **10. In the last two weeks, how much of the time have you felt your lung disease is getting worse?** | | | | | | |
| 1. All of the time | 2. Most of the time | 3. A good bit of the time | 4. Some of the time | 5. A little of the time | 6. Hardly any of the time | 7. None of the time |
| **11. In the last 2 weeks has your lung condition interfered with your job or other daily tasks?** | | | | | | |
| 1. All of the time | 2. Most of the time | 3. A good bit of the time | 4. Some of the time | 5. A little of the time | 6. Hardly any of the time | 7. None of the time |
| **12. In the last 2 weeks have you expected your lung complaint to get worse?** | | | | | | |
| 1. All of the time | 2. Most of the time | 3. A good bit of the time | 4. Some of the time | 5. A little of the time | 6. Hardly any of the time | 7. None of the time |
| **13. In the last 2 weeks, how much has your lung condition limited you carrying things, for example, groceries?** | | | | | | |
| 1. All of the time | 2. Most of the time | 3. A good bit of the time | 4. Some of the time | 5. A little of the time | 6. Hardly any of the time | 7. None of the time |
| **14. In the last 2 weeks, has your lung condition made you think more about the end of your life?** | | | | | | |
| 1. All of the time | 2. Most of the time | 3. A good bit of the time | 4. Some of the time | 5. A little of the time | 6. Hardly any of the time | 7. None of the time |
| **15. Are you financially worse off because of your lung condition?** | | | | | | |
| 1. A significant amount | 2. A large amount | 3. A considerable amount | 4.A reasonable amount | 5. A small amount | 6. Hardly at all | 7. Not at all |

**Appendix S4** Chest computed tomography (CT)

The assessment of lung damage was performed in accordance with the classification according to the degree of recorded changes.

| Chest computer tomography | | | | |
|---|---|---|---|---|
| **Chest CT Score** | **Ground glass** | **Consolidation** | **Signs of viral pneumonia** | **Involvement of the lung parenchyma, %** |
| **CT 0** | No | No | No<br>(The norm and the absence of CT signs of viral pneumonia against a typical clinical pattern and a relevant epidemiological history) | No |
| **CT 1** | Yes | no, single small ones | The absence of CT signs against a typical clinical pattern and a relevant epidemiological history<br>Single areas of small size reticular changes | < 25 |
| **CT 2** | Yes | yes, single | Single areas of small size reticular changes, can be reverse "halo" | 25-49 |
| **CT 3** | Yes | yes, possible solid | Areas of reticular changes, can be reverse "halo",<br>A symptom of an air bronchogram is possible<br>Minimal hydrothorax not associated with pneumonia | 50-74 |
| **CT 4** | Yes | yes, solid predominant | Hydrothorax (bilateral, predominates on the left)<br>Areas of reticular changes, can be reverse "halo",<br>Symptom of air bronchogram<br>Hydrothorax mainly on the left | ≥ 75% |

Image recording, description, and CT conclusion shall be evaluated by the Investigator. Changes in the CT image shall be registered in accordance with the procedure in the primary documentation and in the eCRF.

**Appendix S5** Calculation of the percentage of predicted normal values

| Pulmonary function | Equation |
|---|---|
| **DLCO**[1] | **Men**: $0.142*Height - 0.232*Age + 16.30$ [4]<br>**Women**: $0.219*Height - 0.115*Age - 5.97$ [4] |
| **FVC**[2] | **Men**: $-0.1933 + 0.00064*Age - 0.000269*Age^2 + 0.00018642*Height^2$ [5]<br>**Women**: $-0.3560 + 0.01870*Age - 0.000382*Age^2 + 0.00014815*Height$ [5] |
| **FEV1**[3] | **Men**: $0.5536 - 0.01303*Age - 0.000172*Age^2 + 0.00014098*Height$ [6]<br>**Women**: $0.4333 - 0.00361*Age - 0.000194*Age^2 + 0.00011496*Height$ [6] |
| **TLC**[4] | **Men**: $exp(2.2633*log(Height)+0.00585*Age-7.228*10^{-5}*Age^{2}-9.9529)$ [7]<br>**Women**: $exp(2.246*log(Height)+0.0064*Age-9.386*10^{-5}*Age^{2-9.99})$ [7] |
| **FRC**[5] | **Men**: $0.0234*Height + 0.00009*Age – 0.0109$ [8]<br>**Women**: $0.0224*Height + 0.00001*Age – 0.01$ [8] |

[1]**DLCO**: diffusing capacity for carbon monoxide

[2]**FVC**: forced vital capacity

[3]**FEV1**: forced expiratory volume in one second

[4]**TLC**: total lung capacity

[5]**FRC**: functional residual capacity

**Appendix S6** Reasons for patients' exclusion from study populations

| Patient | Group | Sex | Age | RAN[1] | SAF[2] | FAS[3] | PPS[4] | Reason for exclusion |
|---|---|---|---|---|---|---|---|---|
| 09-02 | Placebo | M | 60 | Yes[5] | Yes[5] | No[6] | No[6] | No efficacy data after baseline. |
| 15-13 | Placebo | M | 61 | Yes[5] | Yes[5] | Yes[5] | No[6] | CT data were erroneously entered at the screening visit. Instead of CT2, CT1 (15-13) was introduced. Instead of CT3, CT1 (15-14) was introduced. Because of this, an incorrect stratification was carried out. |
| 15-14 | Placebo | F | 68 | Yes[5] | Yes[5] | Yes[5] | No[6] | |
| 18-01 | Placebo | M | 56 | Yes[5] | Yes[5] | Yes[5] | No[6] | Incorrectly performed 6-minute walk tests on all visits (patients were stopped after walking a distance of 42 meters). The researcher did not fully understand how to perform this test and did not contact the monitor for clarification. |
| 18-02 | Placebo | M | 65 | Yes[5] | Yes[5] | Yes[5] | No[6] | |
| 18-03 | Treamid | M | 57 | Yes[5] | Yes[5] | Yes[5] | No[6] | |
| 18-04 | Placebo | M | 69 | Yes[5] | Yes[5] | Yes[5] | No[6] | |
| 11-17 | Treamid | F | 57 | Yes[5] | Yes[5] | Yes[5] | No[6] | Serious adverse event: ulcerative keratitis of the left eye. |
| 15-02 | Treamid | F | 60 | Yes[5] | Yes[5] | Yes[5] | No[6] | Spirometry was performed on B1 as part of body plethysmography, followed by spirometry performed on a spirometer in the center. |
| 15-15 | Treamid | F | 58 | Yes[5] | Yes[5] | Yes[5] | No[6] | The patient had an AE of ARVI on 25.10.2020, spirometry was performed on V4 on 26.10.20, and body plethysmography was performed on 30.10. Spirometry was performed on B5 dated 11.03.2020. The protocol states that in case of upper respiratory disease, spirometry can be performed only 7 days after the last symptoms of the disease. |

[1]**RAN**: all randomised patients;

[2]**SAF**: Safety Analysis Set;

[3]**FAS**: Full Analysis Set;

[4]**PPS**: Per-protocol Set.

[5]**Yes**: the patient is included in the set

[6]**No**: the patient is not included in the set

**Appendix 7.** Eight linear models that considered different variants of interaction between fixed factors (Group – group of therapy, Sex – patient sex) and covariate (init – baseline values of corresponding parameter) for a change in a) modified Borg dyspnea scale, b) TLC and c) lung damage as a predictive value. Models marked in bold (with Delta_AICc < 1) were used for the adjusted means calculations. The selection was made based on AICc (Akaike information criterion, corrected for small sample sizes). K – number of parameters (including intercept) + 1, using for AIC calculation. Delta_AICc – difference between AIC of corresponding model and the model with minimal AIC value. AIC weight can be interpreted as weight of evidence in favor of a given model being the best one for the given candidate model set. n – number of patients. ":" – interaction term without main effect, "*" – interaction term with main effect.

**a) Change in dyspnea modified Borg scale (n = 55)**

| Formular | K | AICc | Delta_AICc | AIC weight |
|---|---|---|---|---|
| **init:Group+init:Sex+Group:Sex** | 6 | 99.57 | **0** | **0.26** |
| **init:Sex+Group:Sex** | 6 | 99.88 | **0.31** | **0.22** |
| **init:Group+Group:Sex** | 6 | 99.93 | **0.36** | **0.21** |
| **init+Group*Sex** | 6 | 100.45 | **0.88** | **0.17** |
| init:Sex+Group*Sex | 7 | 102.47 | 2.9 | 0.06 |
| init:Group+Group*Sex | 7 | 102.49 | 2.92 | 0.06 |
| init:Group+init:Sex+Group*Sex | 8 | 104.75 | 5.18 | 0.02 |
| init*Group*Sex | 9 | 107.19 | 7.62 | 0.01 |

**b) Change in TLC, % pred. (n = 58)**

| Formular | K | AICc | Delta_AICc | AIC weight | AIC (median regression) |
|---|---|---|---|---|---|
| **init:Group+Group:Sex** | 6 | 441.34 | **0** | **0.23** | 431.29 |
| **init:Group+init:Sex+Group:Sex** | 6 | 441.56 | **0.23** | **0.2** | 431.25 |
| **init:Group+Group*Sex** | 7 | 441.93 | **0.59** | **0.17** | **425.87** |
| init+Group*Sex | 6 | 442.46 | 1.13 | 0.13 | 432.22 |
| init:Sex+Group:Sex | 6 | 442.68 | 1.34 | 0.12 | 432.17 |
| ***init*Group*Sex*** | 9 | 443.29 | 1.95 | 0.08 | **422.97** |
| ***init:Group+init:Sex+Group*Sex*** | 8 | 444.63 | 3.29 | 0.04 | **425.57** |
| init:Sex+Group*Sex | 7 | 445.05 | 3.71 | 0.04 | 434.16 |

**c) Change in lung damage, % (n = 58)**

| Formular | K | AICc | Delta_AICc | AIC weight |
|---|---|---|---|---|
| **init+Group*Sex** | 6 | 455.59 | **0** | **0.31** |
| **init:Group+Group*Sex** | 7 | 456.19 | **0.6** | **0.23** |
| **init:Sex+Group:Sex** | 6 | 456.41 | **0.82** | **0.2** |
| init:Sex+Group*Sex | 7 | 458.18 | 2.59 | 0.08 |
| init:Group+init:Sex+Group*Sex | 8 | 458.83 | 3.23 | 0.06 |
| init:Group+Group:Sex | 6 | 459.26 | 3.67 | 0.05 |
| init:Group+init:Sex+Group:Sex | 6 | 459.37 | 3.78 | 0.05 |
| init*Group*Sex | 9 | 460.5 | 4.91 | 0.03 |

**Appendix S8.** Parameters of selected linear models for a change in a) modified Borg score, b) TLC and c) lung damage. Group – group of therapy, Sex – patient sex, init – baseline value of corresponding parameter.

**a) Change in modified Borg score**

| Formular | R2 (R2adj), % | F (DF), p value | Coefficient of Group:Sex interaction, p value |
|---|---|---|---|
| init:Group+init:Sex+Group:Sex | 57.0 (53.6) | 16.6 (4,50), < 0.001 | −0.98, 0.001** |
| init:Sex+Group:Sex | 56.8 (53.3) | 16.4 (4,50), < 0.001 | −0.63, 0.002** |
| init:Group+Group:Sex | 56.8 (53.3) | 16.4 (4,50), < 0.001 | −0.79, <0.001*** |
| init+Group*Sex | 56.3 (52.9) | 16.1 (4,50), < 0.001 | −0.95, 0.004** |

**b) TLC, % pred.**

| Formular | R2 (R2adj), % | F (DF), p value | Coefficient of Group:Sex interaction, p value |
|---|---|---|---|
| init:Group+Group*Sex | 24.3 (17.0) | 3.33 (5,52), 0.011 | 14.36, 0.012* |
| init:Group+Group:Sex | 21.6 (15.7) | 3.65 (4,53), 0.011 | 4.09, 0.301 |
| init:Group+init:Sex+Group:Sex | 21.3 (15.4) | 3.59 (4,53), 0.012 | 15.07, 0.008** |

**c) Lung damage, %**

| Formular | R2 (R2adj), % | F (DF), p value | Coefficient of Group:Sex interaction, p value |
|---|---|---|---|
| init:Group+Group*Sex | 22.9 (15.5) | 3.09 (5,52), 0.016 | −15.50, 0.015* |
| init+Group*Sex | 20.2 (14.2) | 3.36 (4,53), 0.016 | −14.65, 0.022* |
| init:Sex+Group:Sex | 19.1 (13.0) | 3.12 (4,53), 0.022 | −4.60, 0.240 |

**Appendix 9.** Results of evaluated adjusted means for selected models for change in a) modified Borg score, b) TLC and c) lung damage. Group – group of therapy, Sex – patient sex, init – baseline value of corresponding parameter.

**a) Change in modified Borg scale**

| model | R2 (R2adj), % | Placebo [95% CI] | Treamid [95% CI] | Treamid superiority [95% CI] | p-value |
|---|---|---|---|---|---|
| init:Group+init:Sex+Group:Sex | 57.0 (53.6) | -0.46 [-0.73; -0.19] | -1.11 [-1.36; -0.85] | 0.64 [0.27; 1.02] | 0.001** |
| init:Sex+Group:Sex | 56.8 (53.3) | -0.46 [-0.73; -0.19] | -1.08 [-1.34; -0.83] | 0.63 [0.25; 1.00] | 0.002** |
| init:Group+Group:Sex | 56.8 (53.3) | -0.46 [-0.76; -0.16] | -1.10 [-1.36; -0.84] | 0.64 [0.25; 1.04] | 0.002** |
| init+Group*Sex | 56.3 (52.9) | -0.47 [-0.77; -0.16] | -1.08 [-1.34; -0.82] | 0.61 [0.22; 1.01] | 0.003** |

**b) Change in TLC, % pred.**

*Linear models:*

| model | R2 (R2adj), % | Placebo [95% CI] | Treamid [95% CI] | Treamid superiority [95% CI] | p-value |
|---|---|---|---|---|---|
| init:Group+Group:Sex | 21.6 (15.7) | -0.42 [-5.92; 5.08] | 7.72 [2.92; 12.52] | 8.14 [0.79; 15.50] | 0.031* |
| init:Group+init:Sex+Group:Sex | 21.3 (15.4) | -0.06 [-5.43; 5.31] | 7.62 [2.82; 12.43] | 7.69 [0.46; 14.91] | 0.038* |
| init:Group+Group*Sex | 24.3 (17.0) | -0.94 [-6.46; 4.58] | 7.38 [2.59; 12.17] | 8.32 [1.01; 15.63] | 0.026* |

*Median regression:*

| model | pseudo-R2, % | Placebo [95% CI] | Treamid [95% CI] | Treamid superiority [95% CI] | p-value |
|---|---|---|---|---|---|
| Init*Group*Sex | 21.4 | -3.96 [-8.49; 0.56] | 7.07 [3.47; 10.68] | 11.04 [5.25; 16.82] | <0.001*** |
| init:Group+init:Sex+Group*Sex | 18.2 | -1.73 [-5.43; 1.98] | 7.61 [0.74; 14.48] | 9.34 [1.66; 17.02] | 0.018* |
| init:Group+Group*Sex | 16.5 | -2.39 [-6.11; 1.33] | 6.19 [1.01; 11.37] | 8.58 [2.20; 14.95] | 0.009** |

**c) Change in lung damage, %**

| model | R2 (R2adj), % | Placebo [95% CI] | Treamid [95% CI] | Treamid superiority [95% CI] | p-value |
|---|---|---|---|---|---|
| init+Group*Sex | 20.2 (14.2) | -11.29 [-17.70; -4.91] | -17.20 [-22.50; -11.94] | 5.91 [-2.35; 14.18] | 0.157 |
| init:Group+Group*Sex | 22.9 (15.5) | -10.89 [-17.30; -4.53] | -17.25 [-22.50; -12.03] | 6.36 [-1.88; 14.59] | 0.127 |
| init:Sex+Group:Sex | 19.1 (13.0) | -12.59 [-18.30; -6.89] | -17.19 [-22.50; -11.89] | 4.60 [-3.16; 12.35] | 0.240 |

**Appendix 10.** Linear models diagnostics for change in a) modified Borg score, b) TLC and c) lung damage. The following parameters are given: maximum variance inflation factor (VIF) value as a measure of collinearity, normality of residuals according to Shapiro–Wilk test, maximum value of Cook's distance as a measure of the influence of a data point and the equality of variance between treatment groups according to F-test.

**a) Change in modified Borg score**

| Formular | Max. VIF value | Normality of residuals (Shapiro–Wilk test) | Max. Cook's distance | Equality of variance F (p-value) |
|---|---|---|---|---|
| init:Group+init:Sex+Group:Sex | 1.82 | W = 0.978, p = 0.400 | 0.29 | 1.82 (0.130) |
| init:Sex+Group:Sex | 1.20 | W = 0.967, p = 0.129 | 0.27 | 1.78 (0.143) |
| init:Group+Group:Sex | 1.25 | W = 0.984, p = 0.684 | 0.19 | 1.77 (0.147) |
| init+Group*Sex | 3.96 | W = 0.974, p = 0.289 | 0.15 | 1.71 (0.171) |

**b) TLC, % pred.**

| Formular | Max. VIF value | Normality of residuals (Shapiro–Wilk test) | Max. Cook's distance | Equality of variance F (p-value) |
|---|---|---|---|---|
| init:Group+Group*Sex | 7.42 | W = 0.931, p = 0.003 | 0.16 | 2.22 (0.040) |
| init:Group+Group:Sex | 1.25 | W = 0.938, p = 0.005 | 0.18 | 2.17 (0.047) |
| init:Group+init:Sex+Group:Sex | 1.92 | W = 0.940, p = 0.006 | 0.25 | 2.40 (0.025) |

**c) Lung damage, %**

| Formular | Max. VIF value | Normality of residuals (Shapiro–Wilk test) | Max. Cook's distance | Equality of variance F (p-value) |
|---|---|---|---|---|
| init:Group+Group*Sex | 2.15 | W = 0.985, p = 0.714 | 0.15 | 1.70 (0.166) |
| init+Group*Sex | 3.75 | W = 0.979, p = 0.398 | 0.16 | 1.68 (0.175) |
| init:Sex+Group:Sex | 1.11 | W = 0.983, p = 0.590 | 0.24 | 1.63 (0.202) |

**Appendix S11**. Linear models for change in modified dyspnea score including baseline value of lung damage (initCT), DLCO (initDLCO) and 6MWD (init6MWD) as covariate and corresponding Treamid superiority in women according to least-squares means differences between Treamid and placebo groups adjusted to two different baseline values (low and high).

| Baseline parameter | Formular | Treamid superiority in women [95% CI], p-value |
|---|---|---|
| Lung damage, % | initCT:Group + Group:Sex | initCT=10%: 0.58 [0.01, 1.16], 0.048*<br>initCT=50%: 0.74 [0.13, 1.35], 0.019* |
| DLCO, % pred. | initDLCO:Group+initDLCO:Sex+Group:Sex | initDLCO=100%: 0.62 [0.04, 1.19], 0.036*<br>initDLCO=60%: 0.66 [0.13, 1.20], 0.016* |
| 6MWD, % pred. | init6MWD:Group + Group:Sex | init6MWD=100%: 0.61 [0.07, 1.15], 0.027*<br>init6MWD=60%: 0.69 [0.16, 1.23], 0.012* |

**Appendix S12**. Treamid superiority in change in lung damage in women (least-squares means differences between Treamid and placebo groups with 95% CI of difference and corresponding p-values) adjusted to different baseline lung damage according to init:Group+Group*Sex linear model (for the model parameter see Appendix 8, c).

| Baseline lung damage, % | Treamid superiority [95% CI] | p-value |
|---|---|---|
| 10 | 2.7 [−6.7, 12.2] | 0.564 |
| 20 | 4.8 [−3.6, 13.1] | 0.261 |
| 30 | 6.8 [−1.6, 15.1] | 0.109 |
| 40 | 8.8 [−0.5, 18.0] | **0.063** |
| 50 | 10.8 [−0.2, 21.7] | **0.053** |

**Appendix S13**. Smoking history for Treamid and placebo groups.

There were no statistically significant differences in smoking history between the cohorts (Table S13.1). Most patients (81%) never smoked. Only two patients in the placebo group were smokers.

**Table S13.1**. Smoking history of **ITT** population.

| **Smoking history** | **Treamid (n = 29)** | **Placebo (n = 30)** | **P value** | **Total (n = 59)** |
|---|---|---|---|---|
| Current | 0 (0.0%) | 2 (6.6%) | 0.492 (f) | 2 (3.4%) |
| Former (≥ 10 pack-years) | 2 (6.9%) | 2 (6.6%) | 1.000 (f) | 4 (6.8%) |
| Former (< 10 pack-years) | 2 (6.9%) | 3 (10.0%) | 1.000 (f) | 5 (8.4%) |
| Never | 25 (86.2%) | 23 (76.8%) | 0.347 (c) | 48 (81.4%) |

[a]In brackets: c, chi-squared test; f, Fisher's exact test.

For non-smokers, the clinically significant improvement at the primary endpoint was observed in the Treamid group compared to placebo in ITT and PPS populations (Table S13.2). This is consistent with the result obtained for whole study cohort.

**Table S13.2.** Primary efficacy outcome for **non-smokers**.

| | Primary efficacy outcome (≥10% increase in FVC or [5–10% increase in FVC and ≥15% increase in DLCO]) | |
|---|---|---|
| mITT | **Placebo**: 5/28 (18%) | **Treamid**: 12/29 (41%) |
| | $p = 0.052$ ($\chi^2$) | |
| PPS | **Placebo**: 4/24 (17%) | **Treamid**: 11/25 (44%) |
| | $p = 0.038$ ($\chi^2$) | |

**Appendix S14**. Cardiovascular diseases (hypertension) for Treamid and placebo groups.

Approximately one half of the randomized patients had a history of hypertension. However, Stage 3 hypertension was diagnosed only in two patients in the placebo group (Table S14.1).

**Table S14.1**. Hypertension in **ITT** population.

| **Hypertension** | **Treamid (n = 29)** | **Placebo (n = 30)** | **P value**[a] | **Total (n = 59)** |
|---|---|---|---|---|
| No | 17 (58.62%) | 11 (36.7%) | 0.091 (c) | 28 (47%) |
| Stage 1 | 7 (24.14%) | 13 (43.3%) | 0.119 (c) | 20 (34%) |
| Stage 2 | 5 (17.24%) | 4 (13.3%) | 0.731 (f) | 9 (15%) |

[a]In brackets: c, chi-squared test; f, Fisher's exact test.

After exclusion of Stage 3 patients, the clinically significant improvement of the primary endpoint was observed in the Treamid group compared to placebo in ITT and PPS populations (Table S14.2). The effect was statistically significant for PPS population and «almost significant» for mITT.

**Table S14.2**. Primary efficacy outcome **excluding patients with hypertension stage 3**.

| | Primary efficacy outcome (≥10% increase in FVC or [5–10% increase in FVC and ≥15% increase in DLCO]) | |
|---|---|---|
| mITT | **Placebo**: 5/28 (18%) | **Treamid**: 12/29 (41%) |
| | p = 0.052 ($\chi^2$) | |
| PPS | **Placebo**: 4/24 (17%) | **Treamid**: 11/25 (44%) |
| | p = 0.038 ($\chi^2$) | |

**Appendix S15.** Difference in analyzed parameters change from baseline to week 4 between patients who achieved and did not achieve clinical benefits.

**Table S15.1** Difference in analyzed parameters change from baseline to week 4 between patients from **mITT** population who achieved (responders) and did not achieve (non-responders) clinical benefits from treatment (M ± SD or n (%)).

| Parameter | Change from baseline to week 4 | | |
|---|---|---|---|
| | ***Responders*** | ***Non-responders*** | ***P value***[a] |
| Number of patients | 17 | 42 | |
| Age (year) | 56 ± 11 | 55 ± 11 | 0.839 (t) |
| Male, n (%) | 7 (41.2) | 19 (45.2) | 0.776 (c) |
| BMI (kg/m$^2$) | 27.4 ± 4.0 | 28.5 ± 4.9 | 0.415 (t) |
| PneumDays | 37 ± 17 | 43 ± 14 | 0.183 (w) |
| SympDays | 44 ± 16 | 48 ± 14 | 0.421 (w) |
| Hospitalization time (day) | 13 ± 5 | 12 ± 8 | 0.906 (w) |
| 6MWD (m) | 63.1 ± 55.3 | 79.8 ± 66.6 | 0.650 (w) |
| 6MWD (%, predicted)[a] | 17.7 ± 17.2 | 21.6 ± 21.1 | 0.674 (w) |
| Borg scale (score) | −0.8 ± 0.5 | −0.6 ± 0.9 | 0.500 (w) |
| mMRC scale (score) | −1.2 ± 0.4 | −1.0 ± 0.6 | 0.293 (w) |
| KBILD questionnaire (total score) | 12.1 ± 6.3 | 13.0 ± 9.5 | 0.763 (w) |
| Breathlessness and activities | 15.1 ± 8.5 | 17.7 ± 12.4 | 0.362 (w) |
| Chest symptoms | 20.2 ± 13.2 | 20.1 ± 16.5 | 0.873 (w) |
| Psychological symptoms | 14.0 ± 8.0 | 15.2 ± 12.9 | 0.722 (t) |
| Pulmonary function: | | | |
| **FEV1 (l)** | 0.3 ± 0.3 | 0.0 ± 0.3 | <0.001 (w) |
| **FVC (l)** | 0.5 ± 0.3 | 0.0 ± 0.2 | <0.001 (t) |
| FEV1/FVC, % | −2.8 ± 4.9 | 2.2 ± 11.5 | 0.061 (w) |
| **TLC (l)** | 0.5 ± 0.4 | 0.2 ± 0.7 | 0.043 (w) |
| FRC (l) | 0.0 ± 0.4 | 0.1 ± 0.6 | 0.969 (w) |
| DLCO (mmol/min/kPa) | 2.3 ± 2.7 | 1.5 ± 2.2 | 0.286 (t) |
| Pulmonary function (%, predicted)[a]: | | | |
| **FEV1** | 10.9 ± 11.1 | 2.1 ± 15.3 | 0.001 (w) |
| **FVC** | 14.7 ± 6.9 | −0.2 ± 5.9 | <0.001 (t) |
| **TLC** | 8.7 ± 9.6 | 4.8 ± 11.5 | 0.043 (w) |
| FRC | 0.2 ± 14.5 | 2.2 ± 17.1 | 0.977 (w) |
| DLCO | 12.9 ± 13.4 | 9.0 ± 12.6 | 0.096 (w) |
| Lung damage, % | −17.4 ± 11.1 | −12.8 ± 12.7 | 0.131 (w) |
| CT score: | | | |
| Increase or no change | 4 (23.5) | 14 (33) | |
| Decrease | 13 (76.5) | 28 (67) | 0.459 (c) |

[a]Data are given in relation to baseline level. Data in bold indicates significant p value (< 0.05).

[a]Test is put in brackets: c – Chi-squared test, t – Student's t-test, u – Mann–Whitney test.

**Table S15.2.** Difference in analyzed parameters change from baseline to week 4 between patients from **Treamid group** who achieved (responders) and did not achieve (non-responders) clinical benefits from treatment (M ± SD or n (%)).

| Parameter | Change from baseline to week 4 | | |
|---|---|---|---|
| | ***Responders*** | ***Non-responders*** | ***P value***[a] |
| Number of patients | 12 | 17 | |
| Age (year) | 55.2 ± 10.7 | 53.5 ± 9.9 | 0.665 (t) |
| Male, n (%) | 4 (33) | 6 (35) | 1.000 (f) |
| BMI ($kg/m^2$) | 28.0 ± 3.8 | 28.9 ± 5.7 | 0.631 (t) |
| PneumDays | 39.4 ± 16.2 | 43.9 ± 10.6 | 0.378 (t) |
| SympDays | 47.2 ± 14.2 | 49.3 ± 10.2 | 0.655 (t) |
| Hospitalization time (day) | 13.8 ± 5.0 | 11.2 ± 8.7 | 0.362 (t) |
| 6MWD (m) | 51.4 ± 54.3 | 96.2 ± 82.8 | 0.221 (w) |
| 6MWD (%, predicted)[a] | 14.7 ± 16.8 | 25.6 ± 26.1 | 0.404 (w) |
| Borg scale (score) | −1.0 ± 0.4 | −0.9 ± 0.9 | 1.000 (w) |
| mMRC scale (score) | −1.2 ± 0.4 | −1.1 ± 0.6 | 0.887 (w) |
| KBILD questionnaire (total score) | 12.7 ± 6.6 | 14.5 ± 7.5 | 0.522 (t) |
| Breathlessness and activities | 16.2 ± 9.2 | 18.3 ± 9.1 | 0.560 (t) |
| Chest symptoms | 22.2 ± 11.8 | 22.0 ± 19.8 | 0.548 (w) |
| Psychological symptoms | 14.2 ± 8.3 | 18.5 ± 10.4 | 0.252 (t) |
| Pulmonary function: | | | |
| **FEV1 (l)** | 0.2 ± 0.2 | 0.1 ± 0.4 | 0.012 (w) |
| **FVC (l)** | 0.4 ± 0.2 | −0.1 ± 0.3 | <0.001 (w) |
| **FEV1/FVC, %** | −2.4 ± 3.7 | 5.9 ± 17.3 | 0.037 (w) |
| TLC (l) | 0.5 ± 0.4 | 0.2 ± 0.5 | 0.189 (t) |
| FRC (l) | 0.1 ± 0.4 | 0.0 ± 0.5 | 0.918 (t) |
| DLCO (mmol/min/kPa) | 2.6 ± 2.8 | 1.5 ± 2.0 | 0.264 (t) |
| Pulmonary function (%, predicted)[a]: | | | |
| **FEV1** | 9.2 ± 9.6 | 3.8 ± 24.0 | 0.007 (w) |
| **FVC** | 12.2 ± 6.2 | −2.2 ± 7.0 | <0.001 (w) |
| TLC | 9.1 ± 8.6 | 4.7 ± 9.0 | 0.110 (w) |
| FRC | 1.9 ± 15.9 | 3.1 ± 17.3 | 0.852 (t) |
| DLCO | 14.3 ± 12.7 | 7.5 ± 10.1 | 0.125 (t) |
| Lung damage, % | −17.9 ± 11.8 | −11.2 ± 11.7 | 0.111 (w) |
| CT score: | | | |
| Increase or no change | 4 (33) | 5 (29) | |
| Decrease | 8 (67) | 12 (71) | 1.000 (f) |

[a]Data are given in relation to baseline level. Data in bold indicates significant p value (< 0.05).

[a]Test is put in brackets: c – Chi-squared test, f – Fisher's exact test, t – Student's t-test, u – Mann–Whitney test.

**Appendix S16.** Exploratory analysis of age, hospitalization time and time after the onset of the first symptoms.

**Table S16.1**. Result summary of the exploratory analysis of a) age, b) hospitalization time and c) time after the onset of the first symptoms: parameters of selected models for a change in modified Borg score, TLC and lung damage. Group – group of therapy, init – baseline value of corresponding parameter. The models were chosen by the analogy of the exploratory analysis of sex.

a) Result summary of the exploratory analysis of **age.**

| Predicted parameter | Characteristics of model | | |
|---|---|---|---|
| | Formular[a] | $R^2$ ($R^2$adj.), %<br>F-statistic (df),<br>p-value | Coefficient of Group:Age interaction,<br>p-value |
| Dyspnea Borg scale score | init:Group +init:Age+ Group*Age<br>(median regression) | 33.7 (pseudo-$R^2$); F (6,48) = 14.6,<br>p < 0.001*** | 0.00,<br>1.000 |
| TLC,<br>% pred. | init*Group*Age<br>(median regression) | 14.9 (pseudo-$R^2$); F (7,50) = 1.99,<br>p = 0.075 | – |
| Lung damage, % | init:Group +init:Age+ Group:Age | 13.6 (7.03); F (4,53) = 2.08,<br>p = 0.097 | – |

[a] ":" – interaction term without main effect, "*" – interaction term with main effect, Age – patient age as descrete factor with two levels (≥58 years old, <58 years old). Patients from Placebo group <58 years old were used as baseline level.

b) Result summary of the exploratory analysis of **hospitalization time**

| Predicted parameter | Characteristics of model | | |
|---|---|---|---|
| | Formular[a] | $R^2$ ($R^2$adj.), %<br>F-statistic (df),<br>p-value | Coefficient of Group:Hosp interaction,<br>p-value |
| Dyspnea Borg scale score | init:Subst+init:Hosp+Subst*Hosp<br>(median regression) | 31.7 (pseudo-$R^2$); F (6,48) = 6.97,<br>p < 0.001*** | 0.00,<br>1.000 |
| TLC,<br>% pred. | init+Subst*Hosp<br>(median regression) | 3.99 (pseudo-$R^2$); F (4,53) = 0.79,<br>p = 0.534 | – |
| Lung damage, % | init+Subst*Hosp | 12.8 (6.16); F (4,53) = 1.94,<br>p = 0.118 | – |

[a] ":" – interaction term without main effect, "*" – interaction term with main effect, Hosp – hospitalization time as discrete factor with two levels (≥14 days, <14 days). Patients from Placebo group with <2 weeks of hospitalization old were used as baseline level.

c) Result summary of the exploratory analysis of **hospitalization time**

| Predicted parameter | Characteristics of model | | |
|---|---|---|---|
| | Formular[a] | $R^2$ ($R^2$adj.), %<br>F-statistic (df),<br>p-value | Coefficient of Group:Symp interaction,<br>p-value |
| Dyspnea Borg scale score | init:Symp+Subst*Symp | 47.4 (42.1); F (5,49) = 8.84,<br>p < 0.001*** | 0.12,<br>0.736 |
| TLC,<br>% pred. | init+Subst*Symp<br>(median regression) | 6.44 (pseudo-$R^2$); F (4,53) = 1.20,<br>p = 0.323 | – |
| Lung damage, % | init+Subst*Symp | 19.0 (12.9); F (4,53) = 3.11,<br>p = 0.023* | 2.41,<br>0.693 |

[a] ":" – interaction term without main effect, "*" – interaction term with main effect. Symp – time after the onset of the first symptoms as discrete factor with two levels (≥53 days, <53 days). Patients from Placebo group with <53 days after the onset of the first symptoms were used as baseline level.

**Table S16.2**. Primary efficacy outcome evaluation for subgroups of patients divided by a) age, b) hospitalization time and c) time after the onset of the first symptoms.

a) Primary efficacy outcome evaluation **for subgroups of patients divided by age**.

| | | | |
|---|---|---|---|
| | **Older patients (≥58 years old)** | | |
| mITT | **Placebo**: 3 out of 15 (20%) | **Treamid**: 7 out of 15 (47%) | p = 0.245 (Fisher's exact test) |
| PPS | **Placebo**: 2 out of 11 (18%) | **Treamid**: 7 out of 13 (54%) | p = 0.105 (Fisher's exact test) |
| | **Younger patients (<58 years old)** | | |
| mITT | **Placebo**: 2 out of 15 (13%) | **Treamid**: 5 out of 14 (36%) | p = 0.215 (Fisher's exact test) |
| PPS | **Placebo**: 2 out of 14 (14%) | **Treamid**: 4 out of 12 (33%) | p = 0.365 (Fisher's exact test) |

b) Primary efficacy outcome evaluation **for subgroups of patients divided by hospitalization time**.

| | | | |
|---|---|---|---|
| | **Hospitalization ≥14 days** | | |
| mITT | **Placebo**: 2 out of 14 (14%) | **Treamid**: 7 out of 15 (47%) | p = 0.109 (Fisher's exact test) |
| PPS | **Placebo**: 2 out of 13 (15%) | **Treamid**: 7 out of 15 (47%) | p = 0.114 (Fisher's exact test) |
| | **Hospitalization <14 days** | | |
| mITT | **Placebo**: 3 out of 16 (19%) | **Treamid**: 5 out of 14 (36%) | p = 0.417 (Fisher's exact test) |
| PPS | **Placebo**: 2 out of 12 (17%) | **Treamid**: 4 out of 10 (40%) | p = 0.348 (Fisher's exact test) |

c) Primary efficacy outcome evaluation for subgroups of patients divided by **time after the onset of the first symptoms.**

| | | | |
|---|---|---|---|
| | **Symptoms onset ≥53 days** | | |
| mITT | **Placebo**: 2 out of 17 (12%) | **Treamid**: 6 out of 14 (43%) | p = 0.097 (Fisher's exact test) |
| PPS | **Placebo**: 2 out of 16 (13%) | **Treamid**: 6 out of 13 (46%) | p = 0.092 (Fisher's exact test) |
| | **Symptoms onset <53 days** | | |
| mITT | **Placebo**: 3 out of 13 (23%) | **Treamid**: 6 out of 15 (40%) | p = 0.435 (Fisher's exact test) |
| PPS | **Placebo**: 2 out of 9 (22%) | **Treamid**: 5 out of 12 (42%) | p = 0.642 (Fisher's exact test) |

**Appendix S17**. Rationale for study drug dose selection based on PK/PD models

Dose selection for the exploratory clinical study was based on data from PK and preclinical efficacy studies. An oral dose of 10 mg/kg showed a therapeutic effect in all mouse models studied. Preclinical tissue distribution data showed that daily administration of this dose administered led to mean respiratory concentration of 0.50 - 0.75 μM. According to preclinical data, oral administration led to uneven distribution between tissue and blood, resulting in higher concentration in tissues. The concentration measured in blood plasma was about 5 times lower compared to lung tissue. Thus, to describe the pharmacokinetics of the drug, it is reasonable to use a multicompartment model. Drug elimination is well described by a two-compartment model with first-order kinetics for absorption and elimination (Figure S16.1) and the ratio of constants $k_{12}/k_{21}>>1$.

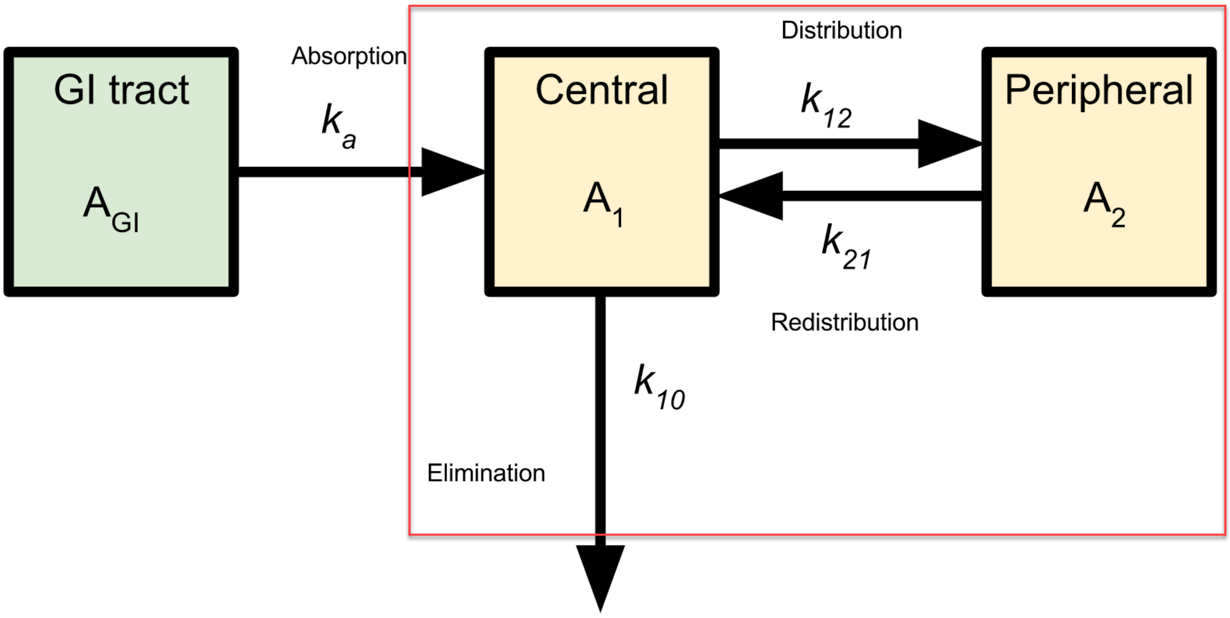


Figure S16.1 General scheme of a two-compartment model of oral pharmacokinetics

Based on the results of the preclinilca pharmacokinetic studies, a model of the pharmacokinetics of the drug in humans with repeated oral administration was built. Data on the concentrations of the substance in human plasma at different time points were taken from the results of phase I clinical trials (NCT04428593).

To build the model, we selected the kinetic constants of adsorption, elimination, and transfer of a substance between compartments at fixed dose parameters (5, 15, and 50 mg), bioavailability (0.02, 0.01, and 0.01, respectively), and the volume of the central compartment (5 L), in order to minimize equation according to the formula:

$$\text{loss} = (\max(C_{\text{pred}}, 0) - C(t))^2/CV$$

where

Cpred is the predicted plasma concentration of the drug,

C(t) – experimentally measured drug concentration in blood plasma.

As a result, the values of the constants characterizing the pharmacokinetics of the drug were found: $k_e = 0.8\ h^{-1}$, $k_a = 1.2\ h^{-1}$, $k_{12} = 8.5E{+}06\ h^{-1}$, $k_{21} = 3.4E{+}06\ h^{-1}$ (Figure S16.2) .

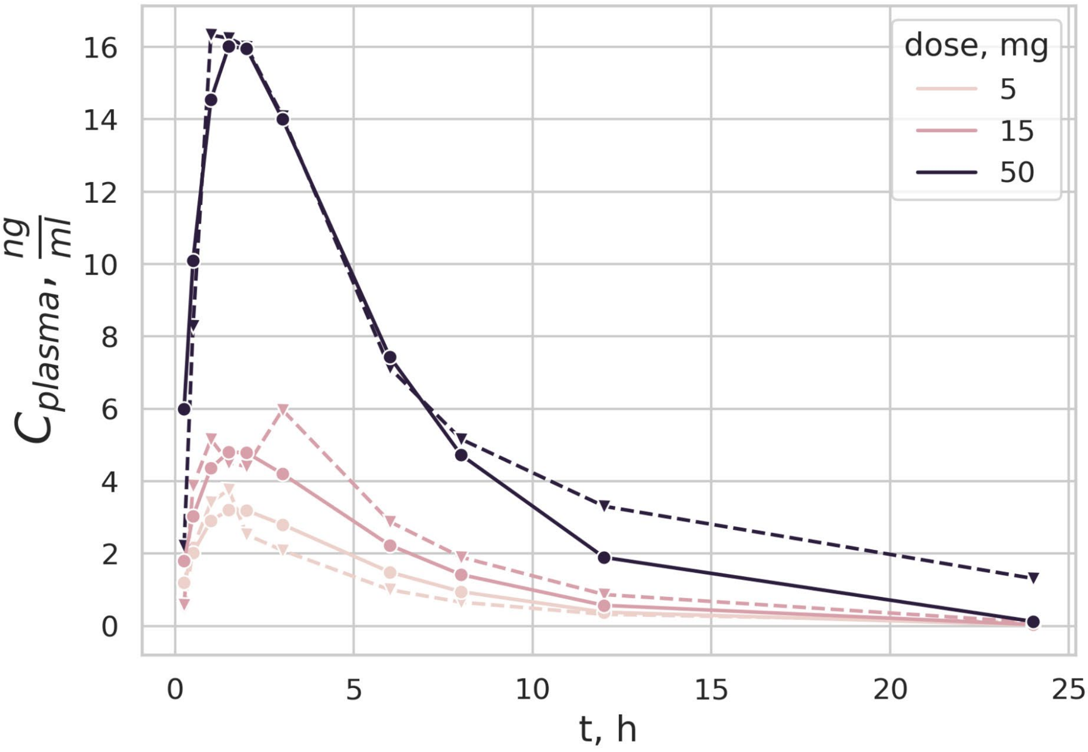


Figure S16.2 Human plasma concentrations obtained in Ph I clinical trial (dashed line) and predicted by the PK model (solid line)

To calculate the concentration of the substance in the lung tissue, data on the distribution of the drug in mice were used. Oral application of 10 mg/kg in mice revealed a linear relation between blood plasma and lung tissue concentration ($R^2$=0.81 for a linear relationship between lung concentration and plasma concentration). This mice plasma concentration is corresponding to the human concentration in dose of 50mg per day. This indicates that the relation ke, $k_a$ << $k_{12}$, $k_{21}$ is preserved for mice. Based on the protocol given in (Proceedings of the National Academy of Sciences Feb 2002, 99 (suppl 1) 2473-2478; DOI: 10.1073/pnas.012579799), the distribution of the substance between the lungs and human blood plasma was recalculated.

$$C_{lung} = C_{plasm} \times 3.6 \times 2 \times 10^3 - 30$$

where

$C_{lung}$ – concentration of the drug in the lungs,

$C_{plasm}$ – plasma concentration,

$3.6 \times 10^3$ –distribution coeffitient,

2 – coefficient of interspecies transfer taking into account the volumes of compartments.

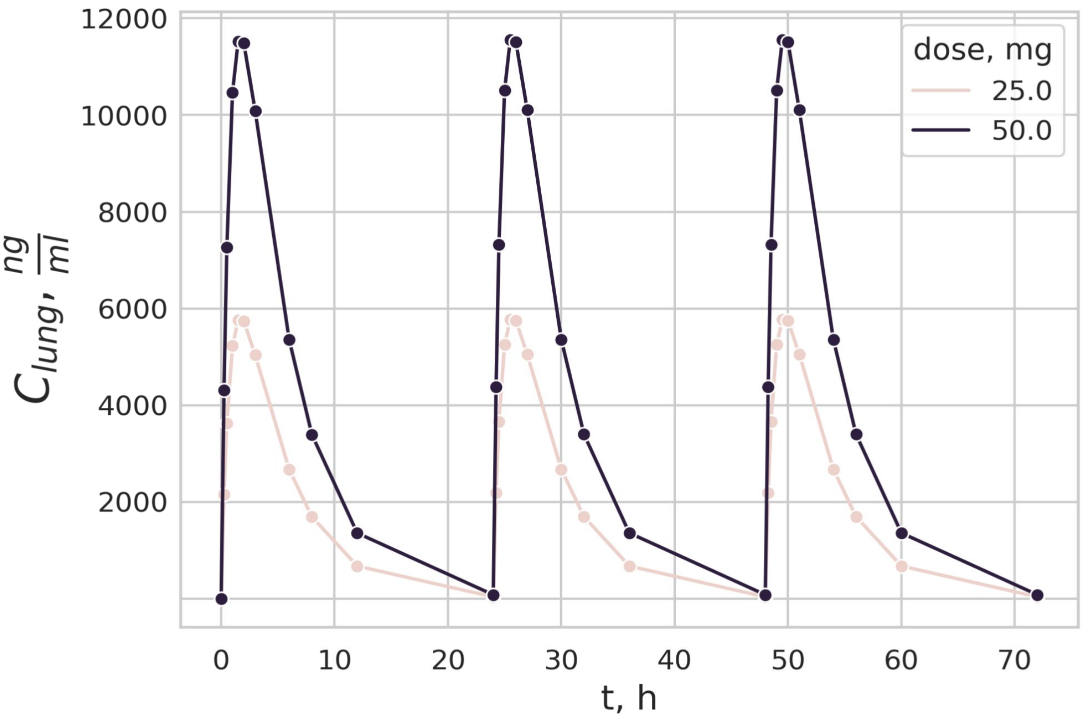

Figure S16.3 Predicted concentration in the human lungs after daily oral administration of Treamid

As a result, it was shown that the administration of Treamid (50 mg daily) allows reaching the average drug concentration in the human respiratory organs at 0.75-1 μM, which correlates with the lung concentrations estimated in mice PK/PD study.

## Figures

**Figure S1.** Correlation of change in a) lung damage and b) TLC with change in FVC in women.

a)

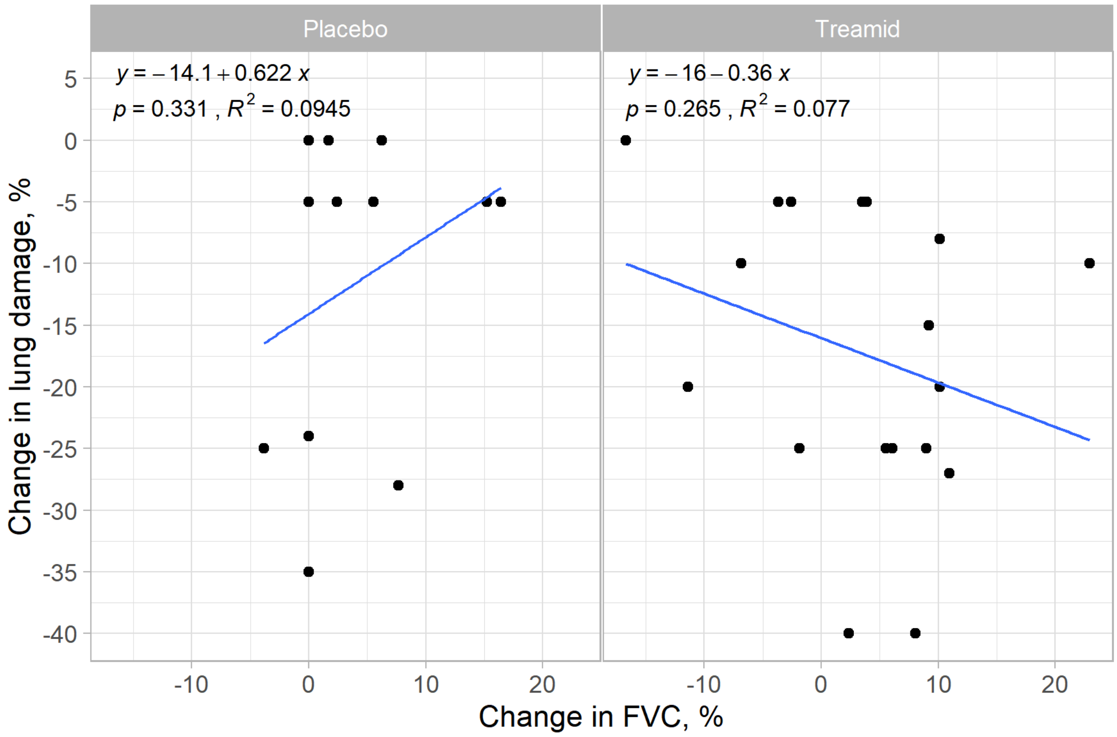


b)

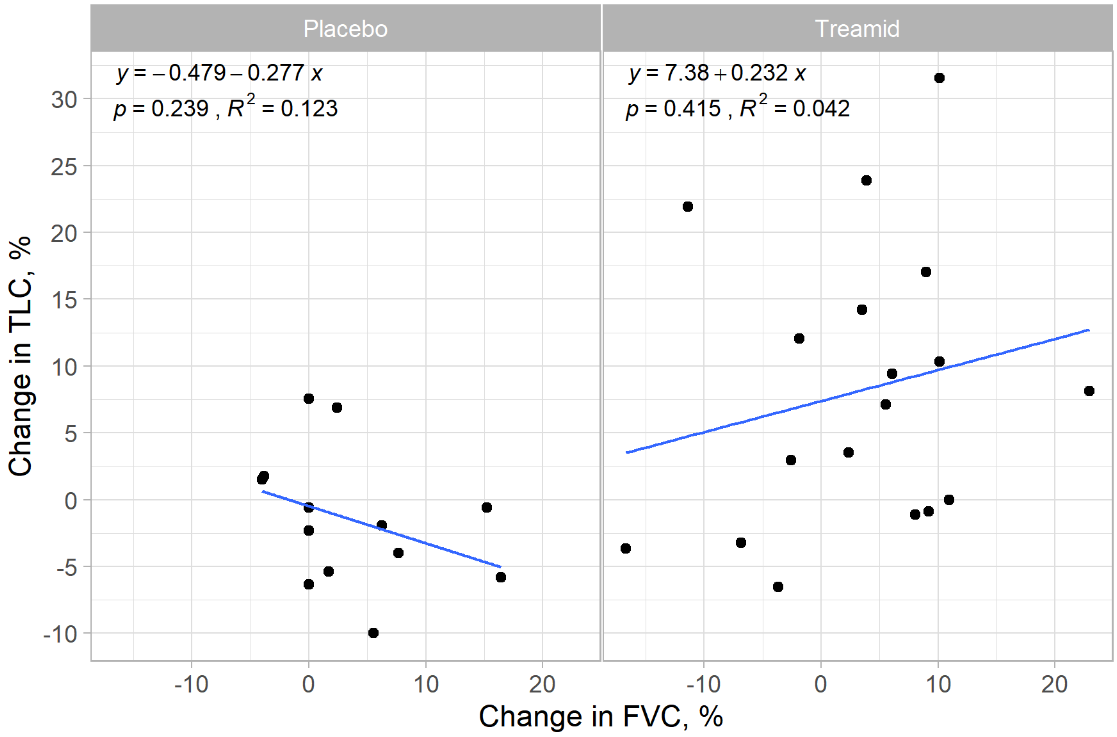

# Tables

**Table S1.** Demographics and baseline clinical characteristics of PPS population by study cohort.

| Parameter | Treamid (n = 25) | Placebo (n = 25) | p-value[a] | Overall (n = 50) |
|---|---|---|---|---|
| Age (year) | 53.6 ± 10.8 | 54.5 ± 12.6 | 0.783 (t) | 54 ± 12 |
| Male, n (%) | 9 (36.0) | 12 (48.0) | 0.390 (c) | 21 (42) |
| BMI (kg/m$^2$) | 28.7 ± 5.1 | 27.3 ± 4.3 | 0.289 (u) | 28 ± 4.7 |
| Lung damage | 27.4 ± 21.6 | 26.1 ± 19.7 | 0.822 (t) | 26.7 ± 20.5 |
| CT score: | | | | |
| 1 | 15 (60.0) | 14 (56.0) | 0.774 (c) | 29 (58) |
| 2 | 7 (28.0) | 10 (40.0) | 0.370 (c) | 17 (34) |
| 3 | 3 (12.0) | 1 (4.0) | 0.609 (f) | 4 (8) |
| Pulmonary function (abs. | | | | |
| FEV1 (l) | 2.7 ± 0.7 | 2.9 ± 0.8 | 0.497 (t) | 2.8 ± 0.7 |
| FVC (l) | 3.4 ± 0.8 | 3.5 ± 0.9 | 0.726 (u) | 3.4 ± 0.8 |
| FEV1/FVC ratio | 80.4 ± 11.0 | 82.3 ± 10.5 | 0.524 (t) | 81.3 ± 10.7 |
| TLC (l) | 5.8 ± 1.4 | 6.0 ± 1.2 | 0.557 (t) | 5.9 ± 1.3 |
| FRC (l) | 3.2 ± 0.9 | 3.4 ± 1.2 | 0.764 (u) | 3.3 ± 1.0 |
| DLCO (mmol/min/kPa) | 20.4 ± 5.2 | 20.2 ± 5.6 | 0.884 (u) | 20.3 ± 5.4 |
| Pulmonary function (%, | | | | |
| FEV1 | 88.1 ± 18.8 | 89.3 ± 16.7 | 0.819 (t) | 88.7 ± 17.7 |
| FVC | 87.0 ± 14.5 | 85.7 ± 15.3 | 0.528 (u) | 86.4 ± 14.7 |
| FEV1/FVC | 100.9 ± 11.4 | 104.5 ± 11.9 | 0.276 (t) | 102.7 ± 11.7 |
| TLC | 97.8 ± 13.7 | 98.5 ± 16.2 | 0.859 (t) | 98.1 ± 14.9 |
| FRC | 102.3 ± 20.6 | 110.8 ± 36.6 | 0.560 (u) | 106.5 ± 29.7 |
| DLCO | 74.6 ± 16.5 | 73.5 ± 15.2 | 0.846 (u) | 74.1 ± 15.7 |
| 6MWD (m) | 426.2 ± 84.3 | 422.1 ± 97.8 | 0.876 (t) | 424.1 ± 90.4 |
| 6MWD (%, predicted) | 78.0 ± 15.5 | 75.7 ± 19.2 | 0.647 (t) | 76.9 ± 17.3 |
| Borg scale (score) | 2.1 ± 0.9 | 1.8 ± 1.1 | 0.227 (t) | 2.0 ± 1.0 |
| mMRC scale (score) | 2.0 ± 0.0 | 2.1 ± 0.3 | 0.161 (u) | 2.0 ± 0.2 |
| KBILD questionnaire (score) | 49.4 ± 3.7 | 51.5 ± 6.2 | 0.148 (t) | 50.5 ± 5.2 |

Data are n (%) or mean ± SD. Baseline defined as the mean assessments at screening (or at randomization for 6MWD, Borg scale, mMRC scale and KBILD questionnaire). **Abbreviations:** 6MWD – distance walked in 6 min walk test; BMI – body mass index; CT – computed tomography; DLCO – diffusing capacity of the lungs for carbon monoxide, adjusted for blood hemoglobin concentration; FEV1 – forced expiratory volume in one second; FRC – functional residual capacity; FVC – forced vital capacity; Hb – hemoglobin; K-BILD – King's brief interstitial lung disease questionnaire; mMRC – modified Medical Research Council dyspnea scale; TLC – total lung capacity.

[a]Test is in brackets: c – Chi-squared test, f – Fisher's exact test, t – Student's t-test, u – Mann–Whitney test.

**Table S2.** The frequency (n (%)) of clinically significant improvement in DLCO after 4-week treatment period in subgroup of patients with gas exchange impairment at baseline.

| **Population** | **Relative change in DLCO, % pred.** | **Treamid n = 23** | **Placebo n = 25** |
|---|---|---|---|
| Baseline DLCO < 80% | **Decrease, no change or <15% increase**, n (%) | 14 (61) | 22 (88) |
| | **≥ 15% increase, n (%)** | 9 (39) | 3 (12) |
| | p-value ($\chi^2$) | 0.030 | |

**Table S3.** Means of baseline values and change for 4 weeks of therapy for ITT population, women, and patients with baseline DLCO < 80%.

| Parameter | Population | Baseline values | | | Change to Week 4 | | |
|---|---|---|---|---|---|---|---|
| | | Treamid | Placebo | p-value[a] | Treamid | Placebo | p-value[a] |
| MWD (m) | mITT | 423.6±80.2 | 418.9±96.7 | 0.844 (t) | 78.6±75.2 | 71.8±50.2 | 0.960 (u) |
| | DLCO < 80% | 405.6±76.8 | 409.3±95.0 | 0.886 (t) | 89.2±74.1 | 81.0±50.7 | 0.981 (u) |
| | Women | 422.9±84.4 | 428.8±96.2 | 0.854 (t) | 83.5±74.7 | 59.5±36.2 | 0.623 (u) |
| MWD (% pred.) | mITT | 78.3±14.8 | 76.0±18.6 | 0.609 (t) | 21.3±23.1 | 19.7±16.5 | 0.720 (u) |
| | DLCO < 80% | 75.5±14.5 | 76.1±18.7 | 0.892 (t) | 25.1±24.1 | 22.5±17.0 | 0.954 (u) |
| | Women | 81.3±15.3 | 80.9±16.2 | 0.952 (t) | 22.4±23.7 | 15.5±11.0 | 0.815 (u) |
| Borg scale (score) | mITT | 2.2±0.8 | 1.8±1.1 | 0.144 (u) | −0.9±0.7 | −0.4±0.8 | **0.018* (u)** |
| | DLCO < 80% | 2.2±0.8 | 1.8±1.1 | 0.114 (u) | −0.9±0.7 | −0.4±0.9 | **0.020* (u)** |
| | Women | 2.2±0.9 | 2.1±1.1 | 0.748 (u) | −1.2±0.5 | −0.5±0.9 | **0.010* (u)** |
| mMRC (score) | mITT | 2.0±0.0 | 2.1±0.3 | 0.168 (u) | −1.1±0.5 | −1.1±0.6 | 0.683 (u) |
| | DLCO < 80% | 2.0±0.0 | 2.1±0.3 | 0.180 (u) | −1.1±0.5 | −1.0±0.7 | 0.827 (u) |
| | Women | 2.0±0.0 | 2.0±0.0 | | −1.1±0.4 | −1.0±0.7 | 0.816 (u) |
| K-BILD, breathlessness and activities | mITT | 37.8±5.5 | 38.8±8.6 | 0.594 (t) | 17.4±9.1 | 16.5±13.4 | 0.383 (u) |
| | DLCO < 80% | 37.1±5.8 | 38.3±9.1 | 0.590 (t) | 18.3±9.0 | 17.3±14.5 | 0.327 (u) |
| | Women | 37.9±5.4 | 37.6±5.4 | 0.903 (t) | 16.3±7.0 | 14.4±5.5 | 0.408 (t) |
| K-BILD, chest symptoms (score) | mITT | 51.6±14.4 | 56.1±14.0 | 0.218 (u) | 22.1±16.7 | 18.3±14.3 | 0.134 (u) |
| | DLCO < 80% | 51.8±16.1 | 56.2±14.5 | 0.264 (u) | 22.2±17.9 | 18.8±15.3 | 0.162 (u) |
| | Women | 51.8±10.4 | 53.2±10.5 | 0.817 (u) | 23.5±12.3 | 18.4±13.6 | 0.272 (t) |
| K-BILD, psychologic (score) | mITT | 49.6±7.7 | 54.3±11.3 | 0.204 (u) | 16.7±9.7 | 13.1±13.2 | 0.145 (u) |
| | DLCO < 80% | 50.1±7.9 | 54.7±12.2 | 0.396 (u) | 17.0±9.6 | 13.8±14.3 | 0.373 (t) |
| | Women | 49.0±6.3 | 52.9±12.0 | 0.232 (t) | 16.9±8.6 | 15.0±9.5 | 0.551 (t) |
| K-BILD (total score) | mITT | 50.2±4.5 | 52.8±6.7 | **0.089** (t) | 13.8±7.1 | 11.7±10.0 | 0.117 (u) |
| | DLCO < 80% | 50.2±4.6 | 52.8±7.2 | 0.283 (u) | 14.2±7.2 | 12.2±10.9 | 0.183 (u) |
| | Women | 50.1±3.9 | 51.6±5.7 | 0.396 (t) | 13.3±5.1 | 11.8±5.5 | 0.439 (t) |
| FEV1 (% pred.) | mITT | 87.5±17.8 | 91.1±17.0 | 0.439 (t) | 6.1±19.1 | 3.4±8.7 | 1.000 (u) |
| | DLCO < 80% | 92.6±15.5 | 92.0±18.4 | 0.904 (t) | 0.7±6.5 | 4.0±9.3 | 0.413 (u) |
| | Women | 86.7±18.0 | 84.3±12.4 | 0.681 (t) | 6.6±22.6 | 1.8±5.9 | 0.936 (u) |
| FVC (% pred.) | mITT | 86.0±14.0 | 87.4±16.2 | 0.932 (u) | 4.0±9.8 | 4.4±8.8 | 0.805 (u) |
| | DLCO < 80% | 88.9±14.3 | 90.0±16.7 | 0.924 (u) | 1.6±8.2 | 4.6±9.6 | 0.668 (u) |
| | Women | 85.1±13.8 | 81.0±9.9 | 0.502 (u) | 3.3±9.3 | 3.6±6.4 | 0.901 (t) |
| FEV1/FVC (% pred.) | mITT | 101.5±10.9 | 104.6±11.2 | 0.283 (t) | 2.1±15.2 | −0.8±5.6 | 0.968 (u) |
| | DLCO < 80% | 104.3±8.3 | 102.3±9.5 | 0.439 (t) | −0.6±5.5 | −0.4±5.6 | 0.478 (u) |
| | Women | 101.7±12.2 | 104.1±8.7 | 0.545 (t) | 3.2±18.8 | −1.6±5.0 | 0.708 (u) |
| TLC (% pred.) | mITT | 96.6±13.8 | 98.5±14.9 | 0.596 (u) | 6.6±8.9 | 5.3±12.8 | 0.255 (u) |
| | DLCO < 80% | 93.7±12.8 | 99.9±15.7 | **0.090** (u) | 6.3±9.1 | 6.0±13.5 | 0.440 (u) |
| | Women | 94.6±14.1 | 102.6±16.9 | 0.117 (u) | 8.1±10.5 | −0.9±5.3 | **0.004* (t)** |
| FRC (% pred.) | mITT | 100.8±21.9 | 108.6±34.7 | 0.601 (u) | 2.6±16.4 | 0.6±16.4 | 0.421 (u) |
| | DLCO < 80% | 97.6±18.3 | 111.8±36.7 | 0.201 (u) | 2.0±18.1 | −2.8±12.2 | 0.311 (t) |
| | Women | 95.2±20.2 | 106.5±45.5 | 0.884 (u) | 2.3±18.0 | −2.4±11.2 | 0.405 (t) |
| DLCO (% pred.) | mITT | 73.9±15.7 | 73.7±14.1 | 0.559 (u) | 10.4±11.6 | 9.9±14.1 | 0.575 (u) |
| | DLCO < 80% | 67.2±7.4 | 69.0±9.6 | 0.231 (u) | 12.8±10.1 | 11.4±14.2 | 0.380 (u) |
| | Women | 73.2±17.3 | 72.6±12.9 | 0.536 (u) | 10.6±12.6 | 9.5±17.7 | 0.595 (u) |
| Lung damage (%) | mITT | 27.9±20.7 | 29.6±21.5 | 0.736 (u) | −14.0±12.0 | −14.3±12.9 | 0.962 (u) |
| | DLCO < 80% | 32.3±20.7 | 32.3±22.3 | 0.975 (u) | −15.4±12.8 | −14.3±13.7 | 0.812 (u) |
| | Women | 27.5±21.5 | 27.4±24.7 | 0.739 (u) | −17.1±11.7 | −10.5±12.6 | **0.073** (u) |

Data in bold indicates p-value < 0.100. [a]Test is put in brackets: t – Student's t-test, u – Mann–Whitney test.

**Table S4.** List of protocol deviations

| ID | Date* | Patient ID | Description of the deviation | Deviation category | The main reason | Planned deviation? (Yes/No) | Corrective action | Preventive action | Significant deviation? (Yes/No) | Comments |
|---|---|---|---|---|---|---|---|---|---|---|
| **1** | **2** | **3** | **4** | **5** | **6** | **7** | **8** | **9** | **10** | **11** |
| 5-1 | 03.11 | 05-01 | On the visit 6 patient was not swabbed nasopharynx and/or oropharynx for PCR for SARS-CoV-2 RNA | **Protocol procedures** | Investigator | No | No | The investigators were interviewed about the protocol procedures | No | If possible, take a swab at visit 7 |
| 5-2 | 20.11 | 05-02 | The patient made the visit 6 two days later scheduled | **Visits windows** | Patient | Yes | No | It was discussed with investigators to instruct patients on the timing of visits in order to avoid deviations. | No | |
| 5-2 | 20.11 | 05-01 | On visits 1, 2, 4, 6, 7 the patient did not have a haemoglobin level determined as part of the urine test due to technical reasons. | **Protocol procedures** | Investigator | Yes | No | No | No | |
| 5-2 | 20.11 | 05-02 | On visits 1, 2, 4, 6, 7 the patient did not have a haemoglobin level determined as part of the urine test due to technical reasons. | **Protocol procedures** | Investigator | Yes | No | No | No | |
| 7-1 | 25.11 | 07-01 | On the visit 6 patient was not swabbed nasopharynx and/or oropharynx for PCR for SARS-CoV-2 RNA | **Protocol procedures** | Investigator | No | No | The investigators were interviewed about the protocol procedures | No | |
| 9-1 | 03.10 | 09-01 | Patient 09-01 made visit 3 two days later than the window of the visit envisaged by the schedule of procedures | **Visits windows** | Patient | No | No | It was re-discussed with the investigator to instruct patients on the timing of visits in order to avoid deviations. | No | |

| 1 | 2 | 3 | 4 | 5 | 6 | 7 | 8 | 9 | 10 | 11 |
|---|---|---|---|---|---|---|---|---|---|---|
| 9-2 | 29.10 | 09-01 | Patient 09-01 made visit 4 a day later than the window of the visit envisaged by the schedule of procedures | **Visits windows** | Patient | No | No | It was re-discussed with the investigator to instruct patients on the timing of visits in order to avoid deviations. | No | |
| 9-2 | 29.10 | 09-01 | Patient 09-01 made visit 5 five days later than the window of the visit envisaged by the schedule of procedures | **Visits windows** | Patient | No | No | It was re-discussed with the investigator to instruct patients on the timing of visits in order to avoid deviations. | No | |
| 9-2 | 29.10 | 09-01 | On the visit 6 patient was not swabbed nasopharynx and/or oropharynx for PCR for SARS-CoV-2 RNA | **Protocol procedures** | Investigator | No | No | The investigators were interviewed about the protocol procedures | No | |
| 9-3 | 27.11 | 09-03 | Patient 09-03 made visit 6 a day later than the window of the visit envisaged by the schedule of procedures | **Visits windows** | Patient | No | No | It was re-discussed with the investigator to instruct patients on the timing of visits in order to avoid deviations. | No | |
| 9-3 | 27.11 | 09-01 | The presence of concomitant chronic diseases in patient 09-01 was not taken into account during stratification | **Randomization** | Investigator | No | No | There was discussion about the need to enter accurate data into the IRC, especially data for stratification. | No | |
| 9-3 | 27.11 | 09-02 | The presence of concomitant chronic diseases in patient 09-02 was not taken into account during stratification | **Randomization** | Investigator | No | No | There was discussion about the need to enter accurate data into the IRC, especially data for stratification | No | |
| 11-1 | 11.11 | 11-01 | On the visit 6 patients were not swabbed nasopharynx and/or oropharynx for PCR for SARS-CoV-2 RNA | **Protocol procedures** | Investigator | No | No | The investigators were interviewed about the protocol procedures | No | If possible, take a swab at visit 7 |

| 1 | 2 | 3 | 4 | 5 | 6 | 7 | 8 | 9 | 10 | 11 |
|---|---|---|---|---|---|---|---|---|---|---|
| 11-1 | 11.11 | 11-09 | On the visit 6 patients were not swabbed nasopharynx and/or oropharynx for PCR for SARS-CoV-2 RNA | **Protocol procedures** | Investigator | No | No | The investigators were interviewed about the protocol procedures | No | If possible, take a swab at visit 7 |
| 11-2 | 24.11 | 11-10 | On the visit 6 patient were not swabbed nasopharynx and/or oropharynx for PCR for SARS-CoV-2 RNA | **Protocol procedures** | Investigator | No | No | The investigators were interviewed about the protocol procedures | No | If possible, take a swab at visit 7 |
| 11-2 | 24.11 | 11-11 | On the visit 6 patient were not swabbed nasopharynx and/or oropharynx for PCR for SARS-CoV-2 RNA | **Protocol procedures** | Investigator | No | No | The investigators were interviewed about the protocol procedures | No | If possible, take a swab at visit 7 |
| 11-2 | 24.11 | 11-12 | On the visit 6 patients were not swabbed nasopharynx and/or oropharynx for PCR for SARS-CoV-2 RNA | **Protocol procedures** | Investigator | No | No | The investigators were interviewed about the protocol procedures | No | If possible, take a swab at visit 7 |
| 11-2 | 24.11 | 11-13 | On the visit 6 patients were not swabbed nasopharynx and/or oropharynx for PCR for SARS-CoV-2 RNA | **Protocol procedures** | Investigator | No | No | The investigators were interviewed about the protocol procedures | No | If possible, take a swab at visit 7 |
| 11-2 | 24.11 | 11-16 | On the visit 6 patients were not swabbed nasopharynx and/or oropharynx for PCR for SARS-CoV-2 RNA | **Protocol procedures** | Investigator | No | No | The investigators were interviewed about the protocol procedures | No | If possible, take a swab at visit 7 |
| 11-2 | 24.11 | 11-17 | On the visit 6 patient were not swabbed nasopharynx and/or oropharynx for PCR for SARS-CoV-2 RNA | **Protocol procedures** | Investigator | No | No | The investigators were interviewed about the protocol procedures | No | If possible, take a swab at visit 7 |
| 11-2 | 24.11 | 11-02 | The presence of concomitant chronic diseases in patients was not taken into account during stratification | **Randomization** | Investigator | No | No | There was discussion about the need to enter accurate data into the IRC, especially data for stratification | No | |
| 11-2 | 24.11 | 11-06 | The presence of concomitant chronic diseases in patients was not taken into account during stratification | **Randomization** | Investigator | No | No | There was discussion about the need to enter accurate data into the | No | |

| 1 | 2 | 3 | 4 | 5 | 6 | 7 | 8 | 9 | 10 | 11 |
|---|---|---|---|---|---|---|---|---|---|---|
| | | | | | | | | IRC, especially data for stratification | | |
| 11-2 | 24.11 | 11-10 | The presence of concomitant chronic diseases in patients was not taken into account during stratification | **Randomization** | Investigator | No | No | There was discussion about the need to enter accurate data into the IRC, especially data for stratification | No | |
| 11-2 | 24.11 | 11-17 | The presence of concomitant chronic diseases in patients was not taken into account during stratification | **Randomization** | Investigator | No | No | There was discussion about the need to enter accurate data into the IRC, especially data for stratification | No | |
| 11-2 | 24.11 | 11-06 | Patient made visit 3 a day later than the window of the visit envisaged by the schedule of procedures | **Visits windows** | Patient | No | No | It was discussed with investigators to instruct patients on the timing of visits in order to avoid deviations. | No | |
| 11-2 | 24.11 | 11-10 | Patient made visit 6 a day later than the window of the visit envisaged by the schedule of procedures | **Visits windows** | Patient | No | No | It was discussed with investigators to instruct patients on the timing of visits in order to avoid deviations. | No | |
| 11-2 | 24.11 | 11-11 | Patient made visit 6 a day later than the window of the visit envisaged by the schedule of procedures | **Visits windows** | Patient | No | No | It was discussed with investigators to instruct patients on the timing of visits in order to avoid deviations. | No | |
| 11-2 | 24.11 | 11-16 | Patient made visit 5 a day later than the window of the visit envisaged by the schedule of procedures | **Visits windows** | Patient | No | No | It was discussed with investigators to instruct patients on the timing | No | |

| 1 | 2 | 3 | 4 | 5 | 6 | 7 | 8 | 9 | 10 | 11 |
|---|---|---|---|---|---|---|---|---|---|---|
| | | | | | | | | of visits in order to avoid deviations. | | |
| 13-1 | 20.11 | 13-08 | B7 was conducted with a deviation of 1 week earlier | **Visits windows** | Investigator | No | No | It was discussed with the principal investigator that visit windows must be strictly observed. | No | |
| 13-1 | 20.11 | 13-03 | The stratification of patients was disturbed. These patients have chronic diseases, and the presence of a risk factor is marked as "no". | **Randomization** | Investigator | No | No | It was discussed with the principal investigator that stratification must be respected and data must be entered into the IRC more carefully. | No | |
| 13-1 | 20.11 | 13-10 | The stratification of patients was disturbed. These patients have chronic diseases, and the presence of a risk factor is marked as "no". | **Randomization** | Investigator | No | No | It was discussed with the principal investigator that stratification must be respected and data must be entered into the IRC more carefully. | No | |
| 13-1 | 20.11 | 13-11 | The stratification of patients was disturbed. These patients have chronic diseases, and the presence of a risk factor is marked as "no". | **Randomization** | Investigator | No | No | It was discussed with the principal investigator that stratification must be respected and data must be entered into the IRC more carefully. | No | |
| 13-1 | 20.11 | 13-12 | The stratification of patients was disturbed. These patients have chronic diseases, and the presence of a risk factor is marked as "no". | **Randomization** | Investigator | No | No | It was discussed with the principal investigator that stratification must be | No | |

| 1 | 2 | 3 | 4 | 5 | 6 | 7 | 8 | 9 | 10 | 11 |
|---|---|---|---|---|---|---|---|---|---|---|
| | | | | | | | | respected and data must be entered into the IRC more carefully. | | |
| 13-1 | 20.11 | 13-05 | The stratification of patients was disturbed. Patients are over 60 years of age and have chronic diseases, and the presence of a risk factor is marked as "no". | **Randomization** | Investigator | No | No | It was discussed with the principal investigator that stratification must be respected and data must be entered into the IRC more carefully. | No | |
| 13-1 | 20.11 | 13-06 | The stratification of patients was disturbed. Patients are over 60 years of age and have chronic diseases, and the presence of a risk factor is marked as "no". | **Randomization** | Investigator | No | No | It was discussed with the principal investigator that stratification must be respected and data must be entered into the IRC more carefully. | No | |
| 13-1 | 20.11 | 13-08 | The stratification of patients was disturbed. Patients are over 60 years of age and have chronic diseases, and the presence of a risk factor is marked as "no". | **Randomization** | Investigator | No | No | It was discussed with the principal investigator that stratification must be respected and data must be entered into the IRC more carefully. | No | |
| 13-1 | 20.11 | 13-09 | The stratification of patients was disturbed. Patients are over 60 years of age and have chronic diseases, and the presence of a risk factor is marked as "no". | **Randomization** | Investigator | No | No | It was discussed with the principal investigator that stratification must be respected and data must be entered into the IRC more carefully. | No | |

| 1 | 2 | 3 | 4 | 5 | 6 | 7 | 8 | 9 | 10 | 11 |
|---|---|---|---|---|---|---|---|---|---|---|
| 15-1 | 19.10 | 15-01 | No body plethysmography on B4 due to acute rhinopharyngitis | **Protocol procedures** | Patient | No | No | No | No | Such non-compliance with investigation procedures does not affect patient safety. |
| 15-1 | 19.10 | 15-01 | Patient had spirometry on B5 dated 12.10.2020. The patient developed an adverse event (ARI (acute rhinopharyngitis)) dated 06.10.2020. The protocol states that spirometry must not be performed earlier than 7 days after the last symptoms of upper respiratory tract disease | **Protocol procedures** | Investigator | No | No | It was discussed with the principal investigator that it was necessary to operate strictly within the protocol | No | Deviation compromises the integrity of the study data, but does not affect the assessment of the primary endpoint. When assessing the secondary endpoint, the data will be assessed as missing and will not be analysed. |
| 15-2 | 03.11 | 15-02 | The patient had spirometry on B3 (01.10) and on B4 (08.10). The patient developed an adverse event (ARI) from 26.09.2020 to 06.10.2020. The protocol states that spirometry must not be performed earlier than 7 days after the last symptoms of upper respiratory tract disease | **Protocol procedures** | Investigator | No | No | It was discussed with the principal investigator that it was necessary to operate strictly within the protocol | No | |

| 1 | 2 | 3 | 4 | 5 | 6 | 7 | 8 | 9 | 10 | 11 |
|---|---|---|---|---|---|---|---|---|---|---|
| 15-2 | 03.11 | 15-02 | Spirometry was performed on B1 as part of a body plethysmography, with subsequent spirometry performed on a spirometer in the centre. | **Protocol procedures** | Investigator | No | No | It was discussed with the investigators that it is important in spirometry to use one device for all measurements. | Yes | |
| 15-2 | 03.11 | 15-13 | Patient had a CT scan mistakenly entered at the screening visit. CT2 was replaced by CT1 (15-13) and CT3 was replaced by CT1 (15-14). As a result, incorrect stratification was carried out. | **Randomization** | Investigator | No | No | Clarifying questions were created in the IRC on these fields. A discussion was held on the need to enter accurate data in the IRC, especially data for stratification. | Yes | |
| 15-2 | 03.11 | 15-14 | Patient at the screening visits had a CT scan mistakenly entered CT scan. CT2 was replaced by CT1 (15-13), CT3 was replaced by CT1 (15-14). CT3 was replaced by CT1 (15-14). As a result, incorrect stratification was carried out. | **Randomization** | Investigator | No | No | Clarifying questions were created in the IRC on these fields. A discussion was held on the need to enter accurate data in the IRC, especially data for stratification. | Yes | |
| 15-3 | 13.11 | 15-09 | Patient 15-10 signed the FIS before patient 15- 09. | **Informed consent** | Investigator | No | No | It was discussed with the principal investigator that it was necessary to register the patient alternately in the study. | No | |
| 15-3 | 13.11 | 15-10 | Patient 15-10 signed the FIS before patient 15- 09. | **Informed consent** | Investigator | No | No | It was discussed with the principal investigator that it was necessary to register | No | |

| 1 | 2 | 3 | 4 | 5 | 6 | 7 | 8 | 9 | 10 | 11 |
|---|---|---|---|---|---|---|---|---|---|---|
| | | | | | | | | the patient alternately in the study. | | |
| 15-3 | 13.11 | 15-11 | Due to a failure in the eIRC and the unavailability of the IWRS, manual distribution of drug packages was carried out by a blinded employee. Patient 15-11 on B5 was given package 0244. According to the protocol, the drug must be dispensed through eIRC. | **Protocol procedures** | Other | No | No | No | No | |
| 15-3 | 13.11 | 15-17 | Due to a failure in the eIRC and the unavailability of the IWRS, manual distribution of drug packages was carried out by a blinded employee. Patient 15-11 on B5 was given package 0239. According to the protocol, the drug must be dispensed through eIRC. | **Protocol procedures** | Other | No | No | No | No | |
| 15-4 | 27.11 | 15-12 | The stratification of patients was disturbed. These patients have chronic diseases, and the presence of a risk factor is marked as "no". | **Randomization** | Investigator | No | No | It was discussed with the principal investigator that the data in the IRC must be noted more carefully. | No | |
| 15-4 | 27.11 | 15-10 | Patient's body plethysmography on B4 was performed with a 7-day deviation (instead of 23.10.2020, it was performed on 30.10.2020) due to lack of recording places at a third-party organisation | **Protocol procedures** | Investigator | No | No | The principal investigator was advised to plan procedures in a timely manner | No | |
| 15-4 | 27.11 | 15-14 | Blood tests at visits 1, 2, 3 in a diabetic patient were not performed on an empty stomach because the patient felt a loss of energy. | **Protocol procedures** | Investigator | No | Performed an empty stomach test on 03.11.2020. | It is recommended that patients should be better informed about compliance with protocol procedures. | Yes | |

| 1 | 2 | 3 | 4 | 5 | 6 | 7 | 8 | 9 | 10 | 11 |
|---|---|---|---|---|---|---|---|---|---|---|
| 15-4 | 27.11 | 15-15 | The patient had an ARI adverse event on 25.10.2020, on B4 of 26.10.20 spirometry was carried out, and on 30.10 body plethysmography was carried out. On B5 of 03.11.2020 spirometry was performed. The protocol states that in case of upper respiratory tract disease, spirometry can only be performed 7 days after the last symptoms of the disease. | **Protocol procedures** | Investigator | No | No | It was discussed with the researchers that this time range must be respected. | Yes | |
| 16-1 | 17.11 | 16-05 | Patient 16-05 did not take into account the presence of comorbidity chronic diseases, which resulted in an incorrect stratification. | **Randomization** | Patient | No | No | A discussion was held on the need to enter accurate data into the IRC, especially data for stratification. | No | |
| 16-1 | 17.11 | 16-03 | Patients 16-03 made visit 6 a day later than the window of the visit envisaged by the schedule of procedures | **Protocol procedures** | Patient | No | No | The investigators were interviewed about the need to instruct patients on the timing of their visits in order to avoid deviations. | No | |
| 16-1 | 17.11 | 16-04 | Patients 16-04 made visit 6 a day later than the window of the visit envisaged by the schedule of procedures | **Protocol procedures** | Patient | No | No | The investigators were interviewed about the need to instruct patients on the timing of their visits in order to avoid deviations. | No | |
| 16-1 | 17.11 | 16-06 | Patient 16-06 was not swabbed nasopharynx and/or oropharynx for PCR for SARS-CoV-2 RNA due to technical reasons | **Protocol procedures** | Investigator | No | No | The researchers were interviewed about protocol procedures | No | |
| 18-1 | 23.11 | 18-02 | B4 in a patient was carried out 1 day late with a deviation. The investigator | **Protocol procedures** | Investigator | No | No | It was discussed with the researcher that | No | |

| 1 | 2 | 3 | 4 | 5 | 6 | 7 | 8 | 9 | 10 | 11 |
|---|---|---|---|---|---|---|---|---|---|---|
| | | | attributed this to the fact that the referral for admission to hospital could not be made in time. | | | | | visitation windows must be respected. | | |
| 18-1 | 23.11 | 18-03 | B4 in a patient was carried out 1 day late with a deviation. The investigator attributed this to the fact that the referral for admission to hospital could not be made in time. | **Protocol procedures** | Investigator | No | No | It was discussed with the researcher that visitation windows must be respected. | No | |
| 18-1 | 23.11 | 18-01 | The 6 minute walk test was not performed correctly at all visits (patients were stopped after walking 42 metres). The investigator attributed this to the fact that she did not fully understand how to perform this test and did not contact the monitor for clarification. | **Protocol procedures** | Investigator | No | No | It was discussed with the principal investigator that protocol procedures must be followed and followed correctly. | Yes | |
| 18-1 | 23.11 | 18-02 | B4 in a patient was carried out 1 day late with a deviation. The investigator attributed this to the fact that the referral for admission to hospital could not be made in time. | **Protocol procedures** | Investigator | No | No | It was discussed with the researcher that visitation windows must be respected. | No | |
| 18-1 | 23.11 | 18-02 | The 6 minute walk test was not performed correctly at all visits (patients were stopped after walking 42 metres). The investigator attributed this to the fact that she did not fully understand how to perform this test and did not contact the monitor for clarification. | **Protocol procedures** | Investigator | No | No | It was discussed with the principal investigator that protocol procedures must be followed and followed correctly. | Yes | |
| 18-1 | 23.11 | 18-03 | The 6 minute walk test was not performed correctly at all visits (patients were stopped after walking 42 metres). The investigator attributed this to the fact that she did not fully understand how to | **Protocol procedures** | Investigator | No | No | It was discussed with the principal investigator that protocol procedures | Yes | |

| 1 | 2 | 3 | 4 | 5 | 6 | 7 | 8 | 9 | 10 | 11 |
|---|---|---|---|---|---|---|---|---|---|---|
| | | | perform this test and did not contact the monitor for clarification. | | | | | must be followed and followed correctly. | | |
| 18-1 | 23.11 | 18-04 | The 6 minute walk test was not performed correctly at all visits (patients were stopped after walking 42 metres). The investigator attributed this to the fact that she did not fully understand how to perform this test and did not contact the monitor for clarification. | **Protocol procedures** | Investigator | No | No | It was discussed with the principal investigator that protocol procedures must be followed and followed correctly. | Yes | |
| 18-1 | 23.11 | 18-01 | The stratification of patients was disturbed. These patients have chronic diseases, and the presence of a risk factor is marked as "no". | **Randomization** | Investigator | No | No | It was discussed that stratification in the eIRC needs to be marked more carefully. | No | |
| 18-1 | 23.11 | 18-02 | The stratification of patients was disturbed. These patients have chronic diseases, and the presence of a risk factor is marked as "no". | **Randomization** | Investigator | No | No | It was discussed that stratification in the eIRC needs to be marked more carefully. | No | |
| 18-1 | 23.11 | 18-03 | The stratification of patients was disturbed. These patients have chronic diseases, and the presence of a risk factor is marked as "no". | **Randomization** | Investigator | No | No | It was discussed that stratification in the eIRC needs to be marked more carefully. | No | |
| 13-2 | 11.12 | 13-01 | B7 was carried out with a deviation of 1 week early. | **Visits windows** | Investigator | No | No | It was discussed with the principal investigator that the visiting windows must be strictly respected. | No | |
| 13-2 | 11.12 | 13-03 | B7 was carried out with a deviation of 1 week early. | **Visits windows** | Investigator | No | No | It was discussed with the principal investigator that the visiting windows must be strictly respected | No | |

| 1 | 2 | 3 | 4 | 5 | 6 | 7 | 8 | 9 | 10 | 11 |
|---|---|---|---|---|---|---|---|---|---|---|
| 13-2 | 11.12 | 13-04 | B7 was carried out with a deviation of 1 week early. | **Visits windows** | Investigator | No | No | It was discussed with the principal investigator that the visiting windows must be strictly respected | No | |
| 13-2 | 11.12 | 13-02 | The patient did not have an FRC body plethysmography score on B1 for technical reasons. | **Protocol procedures** | Investigator | No | No | No | No | |
| 13-2 | 11.12 | 13-01 | Patients did not have: bacteria, leucocytes, salts, cylinders, epithelials and erythrocytes on B1, 2, 4, 6, 7 in the general urine sample due to technical reasons. | **Protocol procedures** | Other | No | No | No | No | |
| 13-2 | 11.12 | 13-03 | Patients did not have: bacteria, leucocytes, salts, cylinders, epithelials and erythrocytes on B1, 2, 4, 6, 7 in the general urine sample due to technical reasons | **Protocol procedures** | Other | No | No | No | No | |
| 13-2 | 11.12 | 13-04 | Patients did not have: bacteria, leucocytes, salts, cylinders, epithelials and erythrocytes on B1, 2, 4, 6, 7 in the general urine sample due to technical reasons | **Protocol procedures** | Other | No | No | No | No | |
| 13-2 | 11.12 | 13-05 | Patients did not have: bacteria, leucocytes, salts, cylinders, epithelials and erythrocytes on B1, 2, 4, 6, 7 in the general urine sample due to technical reasons | **Protocol procedures** | Other | No | No | No | No | |
| 13-2 | 11.12 | 13-06 | Patients did not have: bacteria, leucocytes, salts, cylinders, epithelials and erythrocytes on B1, 2, 4, 6, 7 in the | **Protocol procedures** | Other | No | No | No | No | |

| 1 | 2 | 3 | 4 | 5 | 6 | 7 | 8 | 9 | 10 | 11 |
|---|---|---|---|---|---|---|---|---|---|---|
| | | | general urine sample due to technical reasons | | | | | | | |
| 13-2 | 11.12 | 13-07 | Patients did not have: bacteria, leucocytes, salts, cylinders, epithelials and erythrocytes on B1, 2, 4, 6, 7 in the general urine sample due to technical reasons | **Protocol procedures** | Other | No | No | No | No | |
| 13-2 | 11.12 | 13-08 | Patients did not have: bacteria, leucocytes, salts, cylinders, epithelials and erythrocytes on B1, 2, 4, 6, 7 in the general urine sample due to technical reasons | **Protocol procedures** | Other | No | No | No | No | |
| 13-2 | 11.12 | 13-09 | Patients did not have: bacteria, leucocytes, salts, cylinders, epithelials and erythrocytes on B1, 2, 4, 6, 7 in the general urine sample due to technical reasons | **Protocol procedures** | Other | No | No | No | No | |
| 13-2 | 11.12 | 13-10 | Patients did not have: bacteria, leucocytes, salts, cylinders, epithelials and erythrocytes on B1, 2, 4, 6, 7 in the general urine sample due to technical reasons | **Protocol procedures** | Other | No | No | No | No | |
| 13-2 | 11.12 | 13-11 | Patients did not have: bacteria, leucocytes, salts, cylinders, epithelials and erythrocytes on B1, 2, 4, 6, 7 in the general urine sample due to technical reasons | **Protocol procedures** | Other | No | No | No | No | |
| 13-2 | 11.12 | 13-12 | Patients did not have: bacteria, leucocytes, salts, cylinders, epithelials and erythrocytes on B1, 2, 4, 6, 7 in the | **Protocol procedures** | Other | No | No | No | No | |

| 1 | 2 | 3 | 4 | 5 | 6 | 7 | 8 | 9 | 10 | 11 |
|---|---|---|---|---|---|---|---|---|---|---|
| | | | general urine sample due to technical reasons | | | | | | | |
| 13-2 | 11.12 | 13-13 | Patients did not have: bacteria, leucocytes, salts, cylinders, epithelials and erythrocytes on B1, 2, 4, 6, 7 in the general urine sample due to technical reasons | **Protocol procedures** | Other | No | No | No | No | |
| 13-2 | 11.12 | 13-14 | Patients did not have: bacteria, leucocytes, salts, cylinders, epithelials and erythrocytes on B1, 2, 4, 6, 7 in the general urine sample due to technical reasons | **Protocol procedures** | Other | No | No | No | No | |
| 13-2 | 11.12 | 13-03 | No erythrocyte sedimentation rate was performed in patients on B1 due to technical reasons | **Protocol procedures** | Other | No | No | No | No | |
| 13-2 | 11.12 | 13-04 | No erythrocyte sedimentation rate was performed in patients on B1 due to technical reasons | **Protocol procedures** | Other | No | No | No | No | |
| 13-2 | 11.12 | 13-08 | No erythrocyte sedimentation rate was performed in patients on B1 due to technical reasons | **Protocol procedures** | Other | No | No | No | No | |
| 13-2 | 11.12 | 13-09 | No erythrocyte sedimentation rate was performed in patients on B1 due to technical reasons | **Protocol procedures** | Other | No | No | No | No | |
| 13-2 | 11.12 | 13-10 | No erythrocyte sedimentation rate was performed in patients on B1 due to technical reasons | **Protocol procedures** | Other | No | No | No | No | |

| 1 | 2 | 3 | 4 | 5 | 6 | 7 | 8 | 9 | 10 | 11 |
|---|---|---|---|---|---|---|---|---|---|---|
| 13-2 | 11.12 | 13-01, | No erythrocyte sedimentation rate was performed in patients on B2 due to technical reasons | **Protocol procedures** | Other | No | No | No | No | |
| 13-2 | 11.12 | 13-03 | No erythrocyte sedimentation rate was performed in patients on B2 due to technical reasons | **Protocol procedures** | Other | No | No | No | No | |
| 13-2 | 11.12 | 13-06 | No erythrocyte sedimentation rate was performed in patients on B2 due to technical reasons | **Protocol procedures** | Other | No | No | No | No | |
| 13-2 | 11.12 | 13-07 | No erythrocyte sedimentation rate was performed in patients on B2 due to technical reasons | **Protocol procedures** | Other | No | No | No | No | |
| 13-2 | 11.12 | 13-08 | No erythrocyte sedimentation rate was performed in patients on B2 due to technical reasons | **Protocol procedures** | Other | No | No | No | No | |
| 13-2 | 11.12 | 13-12 | No erythrocyte sedimentation rate was performed in patients on B6 due to technical reasons | **Protocol procedures** | Other | No | No | No | No | |
| 13-2 | 11.12 | 13-04 | Patients on B2 did not have basophils in CBC for technical reasons | **Protocol procedures** | Other | No | No | No | No | |
| 13-2 | 11.12 | 13-12 | Patients on B2 did not have basophils in CBC for technical reasons | **Protocol procedures** | Other | No | No | No | No | |
| 13-2 | 11.12 | 13-14 | Patients on B2 did not have basophils in CBC for technical reasons | **Protocol procedures** | Other | No | No | No | No | |
| 13-2 | 11.12 | 13-14 | Patients on B1 did not have basophils in CBC for technical reasons | **Protocol procedures** | Other | No | No | No | No | |

| 1 | 2 | 3 | 4 | 5 | 6 | 7 | 8 | 9 | 10 | 11 |
|---|---|---|---|---|---|---|---|---|---|---|
| 13-2 | 11.12 | 13-12 | Patients on B7 did not have monocytes in CBC due to technical reasons | **Protocol procedures** | Other | No | No | No | No | |
| 13-2 | 11.12 | 13-14 | Patients on B7 did not have monocytes in CBC due to technical reasons | **Protocol procedures** | Other | No | No | No | No | |
| 13-2 | 11.12 | 13-04 | Patient 13-04 on B1 did not have eosinophins in CDC due to technical reasons | **Protocol procedures** | Other | No | No | No | No | |
| 13-2 | 11.12 | 13-12 | Patient 13-12 on B1 did not have eosinophins in CDC due to technical reasons | **Protocol procedures** | Other | No | No | No | No | |
| 13-2 | 11.12 | 13-14 | Patient 13-14 on B1 did not have eosinophins in CDC due to technical reasons | **Protocol procedures** | Other | No | No | No | No | |
| 13-2 | 11.12 | 13-01 | GGT, chlorine, sodium, alkaline phosphatase, creatine phosphokinase in BC on B1,2,4 not performed due to technical reasons | **Protocol procedures** | Other | No | No | No | No | |
| 13-2 | 11.12 | 13-05 | GGT, chlorine, sodium, alkaline phosphatase, creatine phosphokinase in BC on B1,2,4 not performed due to technical reasons | **Protocol procedures** | Other | No | No | No | No | |
| 13-2 | 11.12 | 13-06 | GGT, chlorine, sodium, alkaline phosphatase, creatine phosphokinase in BC on B1,2,4 not performed due to technical reasons | **Protocol procedures** | Other | No | No | No | No | |
| 13-2 | 11.12 | 13-08 | GGT, chlorine, sodium, alkaline phosphatase, creatine phosphokinase in BC on B1,2,4 not performed due to technical reasons | **Protocol procedures** | Other | No | No | No | No | |

| 1 | 2 | 3 | 4 | 5 | 6 | 7 | 8 | 9 | 10 | 11 |
|---|---|---|---|---|---|---|---|---|---|---|
| 13-2 | 11.12 | 13-09 | GGT, chlorine, sodium, alkaline phosphatase, creatine phosphokinase in BC on B1,2,4 not performed due to technical reasons | **Protocol procedures** | Other | No | No | No | No | |
| 13-2 | 11.12 | 13-10 | GGT, chlorine, sodium, alkaline phosphatase, creatine phosphokinase in BC on B1,2,4 not performed due to technical reasons | **Protocol procedures** | Other | No | No | No | No | |
| 13-2 | 11.12 | 13-11 | GGT, chlorine, sodium, alkaline phosphatase, creatine phosphokinase in BC on B1,2,4 not performed due to technical reasons | **Protocol procedures** | Other | No | No | No | No | |
| 13-2 | 11.12 | 13-12 | GGT, chlorine, sodium, alkaline phosphatase, creatine phosphokinase in BC on B1,2,4 not performed due to technical reasons | **Protocol procedures** | Other | No | No | No | No | |
| 15-5 | 08.12 | 15-01 | CT scan was performed later than B6 because the CT scan was performed at an external organisation and due to the epidemiological situation the recording was limited. | **Protocol procedures** | Investigator | No | No | It was discussed with the principal investigator that patients must be registered in advance for protocol procedures with external organisations | No | |
| 15-5 | 08.12 | 15-02 | CT scan was performed later than B6 because the CT scan was performed at an external organisation and due to the epidemiological situation the recording was limited. | **Protocol procedures** | Investigator | No | No | It was discussed with the principal investigator that patients must be registered in advance for protocol procedures with external organisations | No | |

| 1 | 2 | 3 | 4 | 5 | 6 | 7 | 8 | 9 | 10 | 11 |
|---|---|---|---|---|---|---|---|---|---|---|
| 15-5 | 08.12 | 15-03 | CT scan was performed later than B6 because the CT scan was performed at an external organisation and due to the epidemiological situation the recording was limited. | **Protocol procedures** | Investigator | No | No | It was discussed with the principal investigator that patients must be registered in advance for protocol procedures with external organisations | No | |
| 15-5 | 08.12 | 15-05 | CT scan was performed later than B6 because the CT scan was performed at an external organisation and due to the epidemiological situation the recording was limited. | **Protocol procedures** | Investigator | No | No | It was discussed with the principal investigator that patients must be registered in advance for protocol procedures with external organisations | No | |
| 15-5 | 08.12 | 15-06 | CT scan was performed later than B6 because the CT scan was performed at an external organisation and due to the epidemiological situation the recording was limited. | **Protocol procedures** | Investigator | No | No | It was discussed with the principal investigator that patients must be registered in advance for protocol procedures with external organisations | No | |
| 15-5 | 08.12 | 15-07 | CT scan was performed later than B6 because the CT scan was performed at an external organisation and due to the epidemiological situation the recording was limited. | **Protocol procedures** | Investigator | No | No | It was discussed with the principal investigator that patients must be registered in advance for protocol procedures with external organisations | No | |

| 1 | 2 | 3 | 4 | 5 | 6 | 7 | 8 | 9 | 10 | 11 |
|---|---|---|---|---|---|---|---|---|---|---|
| 15-5 | 08.12 | 15-08 | CT scan was performed later than B6 because the CT scan was performed at an external organisation and due to the epidemiological situation the recording was limited. | **Protocol procedures** | Investigator | No | No | It was discussed with the principal investigator that patients must be registered in advance for protocol procedures with external organisations | No | |
| 15-5 | 08.12 | 15-09 | CT scan was performed later than B6 because the CT scan was performed at an external organisation and due to the epidemiological situation the recording was limited. | **Protocol procedures** | Investigator | No | No | It was discussed with the principal investigator that patients must be registered in advance for protocol procedures with external organisations | No | |
| 15-5 | 08.12 | 15-10 | CT scan was performed later than B6 because the CT scan was performed at an external organisation and due to the epidemiological situation the recording was limited. | **Protocol procedures** | Investigator | No | No | It was discussed with the principal investigator that patients must be registered in advance for protocol procedures with external organisations | No | |
| 15-5 | 08.12 | 15-11 | CT scan was performed later than B6 because the CT scan was performed at an external organisation and due to the epidemiological situation the recording was limited. | **Protocol procedures** | Investigator | No | No | It was discussed with the principal investigator that patients must be registered in advance for protocol procedures with external organisations | No | |

| 1 | 2 | 3 | 4 | 5 | 6 | 7 | 8 | 9 | 10 | 11 |
|---|---|---|---|---|---|---|---|---|---|---|
| 15-5 | 08.12 | 15-12 | CT scan was performed later than B6 because the CT scan was performed at an external organisation and due to the epidemiological situation the recording was limited. | **Protocol procedures** | Investigator | No | No | It was discussed with the principal investigator that patients must be registered in advance for protocol procedures with external organisations | No | |
| 15-5 | 08.12 | 15-13 | CT scan was performed later than B6 because the CT scan was performed at an external organisation and due to the epidemiological situation the recording was limited. | **Protocol procedures** | Investigator | No | No | It was discussed with the principal investigator that patients must be registered in advance for protocol procedures with external organisations | No | |
| 15-5 | 08.12 | 15-14 | CT scan was performed later than B6 because the CT scan was performed at an external organisation and due to the epidemiological situation the recording was limited. | **Protocol procedures** | Investigator | No | No | It was discussed with the principal investigator that patients must be registered in advance for protocol procedures with external organisations | No | |
| 15-5 | 08.12 | 15-15 | CT scan was performed later than B6 because the CT scan was performed at an external organisation and due to the epidemiological situation the recording was limited. | **Protocol procedures** | Investigator | No | No | It was discussed with the principal investigator that patients must be registered in advance for protocol procedures with external organisations | No | |

| 1 | 2 | 3 | 4 | 5 | 6 | 7 | 8 | 9 | 10 | 11 |
|---|---|---|---|---|---|---|---|---|---|---|
| 15-5 | 08.12 | 15-16 | CT scan was performed later than B6 because the CT scan was performed at an external organisation and due to the epidemiological situation the recording was limited. | **Protocol procedures** | Investigator | No | No | It was discussed with the principal investigator that patients must be registered in advance for protocol procedures with external organisations | No | |
| 15-5 | 08.12 | 15-17 | CT scan was performed later than B6 because the CT scan was performed at an external organisation and due to the epidemiological situation the recording was limited. | **Protocol procedures** | Investigator | No | No | It was discussed with the principal investigator that patients must be registered in advance for protocol procedures with external organisations | No | |
| 15-5 | 08.12 | 15-05 | Patient came to B7 later than planned due to personal circumstances. 1505 3 days late. | **Visits windows** | Patient | No | No | It was discussed with the principal investigator that patients needed to be instructed about the visit windows in more detail and asked to better follow these windows. | No | |
| 15-5 | 08.12 | 15-06 | Patient came to B7 later than planned due to personal circumstances. 1506 2 days late | **Visits windows** | Patient | No | No | It was discussed with the principal investigator that patients needed to be instructed about the visit windows in more detail and asked to | No | |

| 1 | 2 | 3 | 4 | 5 | 6 | 7 | 8 | 9 | 10 | 11 |
|---|---|---|---|---|---|---|---|---|---|---|
| | | | | | | | | better follow these windows. | | |
| 15-5 | 08.12 | 15-07 | Patient came to B7 later than planned due to personal circumstances. 1507 2 days late | **Visits windows** | Patient | No | No | It was discussed with the principal investigator that patients needed to be instructed about the visit windows in more detail and asked to better follow these windows. | No | |
| 15-5 | 08.12 | 15-09 | Patient came to B7 later than planned due to personal circumstances. 1509 2 days late | **Visits windows** | Patient | No | No | It was discussed with the principal investigator that patients needed to be instructed about the visit windows in more detail and asked to better follow these windows. | No | |
| 15-5 | 08.12 | 15-12 | Patient came to B7 later than planned due to personal circumstances. 1512 4 days late | **Visits windows** | Patient | No | No | It was discussed with the principal investigator that patients needed to be instructed about the visit windows in more detail and asked to better follow these windows. | No | |
| 15-5 | 08.12 | 15-15 | Patient came to B7 later than planned due to personal circumstances. 1515 12 days late | **Visits windows** | Patient | No | No | It was discussed with the principal investigator that patients needed to be | No | |

| 1 | 2 | 3 | 4 | 5 | 6 | 7 | 8 | 9 | 10 | 11 |
|---|---|---|---|---|---|---|---|---|---|---|
| | | | | | | | | instructed about the visit windows in more detail and asked to better follow these windows. | | |
| 18-2 | 10.12 | 18-04 | The patient did not have an FRC body plethysmography score on B6 for technical reasons. | **Protocol procedures** | Investigator | No | No | No | No | |
| 18-2 | 10.12 | 18-03 | The patient did not have an FRC body plethysmography score on B6 for technical reasons. | **Protocol procedures** | Investigator | No | No | No | Yes | |
| 18-2 | 10.12 | 18-02 | The patient did not have an FRC body plethysmography score on B6 for technical reasons. | **Protocol procedures** | Investigator | No | No | No | No | |
| 18-2 | 10.12 | 18-01 | The patient did not have an FRC body plethysmography score on B4 for technical reasons. | **Protocol procedures** | Investigator | No | No | No | Yes | |
| 18-2 | 10.12 | 18-02 | Patient did not have sodium parameter in BC on B1 due to technical reasons | **Protocol procedures** | Other | No | No | No | No | |
| 16-2 | 04.12 | 16-06 | Patient 16-06 on Visits 1, 2, 4 did not have pH determined as part of the general urinalysis due to technical reasons. | **Protocol procedures** | Investigator | No | No | No | No | |
| 16-2 | 04.12 | 16-06 | Patient 16-06 on Visit 7 did not have transparency and color determined as part of the general urinalysis due to technical reasons. | **Protocol procedures** | Investigator | No | No | | No | |
| 16-2 | 04.12 | 16-01 | Patient 16-01 on Visits 1, 2, 4, 6, 7 did not have gamma-glutamyltransferase levels | **Protocol procedures** | Investigator | No | No | No | No | |

| 1 | 2 | 3 | 4 | 5 | 6 | 7 | 8 | 9 | 10 | 11 |
|---|---|---|---|---|---|---|---|---|---|---|
| | | | determined as part of the biochemical blood test due to technical reasons. | | | | | | | |
| 16-2 | 04.12 | 16-02 | Patient 16-02 on Visits 1, 2, 4, 6, 7 did not have gamma-glutamyltransferase levels determined as part of the biochemical blood test due to technical reasons. | **Protocol procedures** | Investigator | No | No | No | No | |
| 16-2 | 04.12 | 16-03 | Patient 16-03 on Visits 1, 2, 4, 6, 7 did not have gamma-glutamyltransferase levels determined as part of the biochemical blood test due to technical reasons. | **Protocol procedures** | Investigator | No | No | No | No | |
| 16-2 | 04.12 | 16-04 | Patient 16-04 on Visits 1, 2, 4, 6, 7 did not have gamma-glutamyltransferase levels determined as part of the biochemical blood test due to technical reasons. | **Protocol procedures** | Investigator | No | No | No | No | |
| 16-2 | 04.12 | 16-05 | Patient 16-05 on Visits 1, 2, 4, 6, 7 did not have gamma-glutamyltransferase levels determined as part of the biochemical blood test due to technical reasons. | **Protocol procedures** | Investigator | No | No | No | No | |
| 16-2 | 04.12 | 16-06 | Patient 16-06 on Visits 1, 2, 4, 6, 7 did not have gamma-glutamyltransferase levels determined as part of the biochemical blood test due to technical reasons. | **Protocol procedures** | Investigator | No | No | No | No | |
| 16-2 | 04.12 | 16-01 | Patient 16-01 on Visit 4 did not have potassium levels determined as part of the biochemical blood test due to technical reasons. | **Protocol procedures** | Investigator | No | No | No | No | |
| 16-2 | 04.12 | 16-02 | Patient 16-02 on Visit 6 did not have potassium, sodium, chlorine levels determined as part of the biochemical blood test due to technical reasons. | **Protocol procedures** | Investigator | No | No | No | No | |

| 1 | 2 | 3 | 4 | 5 | 6 | 7 | 8 | 9 | 10 | 11 |
|---|---|---|---|---|---|---|---|---|---|---|
| 16-2 | 04.12 | 16-05 | Patient 16-05 on Visit 6 did not have creatine phosphokinase, alkaline phosphatase levels determined as part of the biochemical blood test due to technical reasons. | **Protocol procedures** | Investigator | No | No | No | No | |
| 16-2 | 04.12 | 16-06 | Patient 16-06 on Visit 6 did not have creatine phosphokinase, alkaline phosphatase levels determined as part of the biochemical blood test due to technical reasons. | **Protocol procedures** | Investigator | No | No | No | No | |
| 16-2 | 04.12 | 16-06 | Patient 16-06 on Visit 4 did not have a complete blood count and biochemical blood count due to technical reasons. | **Protocol procedures** | Investigator | No | No | No | No | |
| 16-2 | 04.12 | 16-01 | Patient 16-01 on Visit 4 did not have an erythrocyte sedimentation rate determination as part of the general blood test for technical reasons. | **Protocol procedures** | Investigator | No | No | No | No | |
| 16-2 | 04.12 | 16-05 | Patient 16-05 on Visit 4 did not have an erythrocyte sedimentation rate determination as part of the general blood test for technical reasons. | **Protocol procedures** | Investigator | No | No | No | No | |
| 16-2 | 04.12 | 16-06 | Patient 16-06 on Visit 4 did not have an erythrocyte sedimentation rate determination as part of the general blood test for technical reasons. | **Protocol procedures** | Investigator | No | No | No | No | |
| 9-2 | 29.10 | 09-03 | Patient 09-03 made visit 6 a day later than the window of the visit envisaged by the schedule of procedures | **Visits windows** | Patient | No | No | It was re-discussed with the investigators to instruct patients on the timing of visits in order to avoid deviations. | No | |

| 1 | 2 | 3 | 4 | 5 | 6 | 7 | 8 | 9 | 10 | 11 |
|---|---|---|---|---|---|---|---|---|---|---|
| 9-3 | 27.11 | 09-01 | Patient 09-01 made visit 7 two days later than the window of the visit envisaged by the schedule of procedures | **Visits windows** | Patient | No | No | It was re-discussed with the investigators to instruct patients on the timing of visits in order to avoid deviations. | No | |
| 9-3 | 27.11 | 09-01 | Patients 09-01 on visits 1, 2, 4, 6, 7 did not have haemoglobin levels determined as part of their urinalysis due to technical reasons. | **Protocol procedures** | Investigator | No | No | No | No | |
| 9-3 | 27.11 | 09-03 | Patients 09-03 on visits 1, 2, 4, 6, 7 did not have haemoglobin levels determined as part of their urinalysis due to technical reasons. | **Protocol procedures** | Investigator | No | No | No | No | |
| 9-3 | 27.11 | 09-02 | Patients 09-02 on visits 1, 2, did not have haemoglobin levels determined as part of their urinalysis due to technical reasons. | **Protocol procedures** | Investigator | No | No | No | No | |
| 5-3 | 08.12 | 05-02 | Patient made visit 7 two days later than the window of the visit envisaged by the schedule of procedures | **Visits windows** | Patient | No | No | It was discussed with the investigators to instruct patients on the timing of visits in order to avoid deviations. | No | |
| 11-3 | 07.12 | 11-03 | Patient 11-03 made visit 7 two days later than the window of the visit envisaged by the schedule of procedures | **Visits windows** | Patient | No | No | It was discussed with the investigators to instruct patients on the timing of visits in order to avoid deviations. | No | |
| 11-3 | 07.12 | 11-09 | Patient 11-09 made visit 7 two days later than the window of the visit envisaged by the schedule of procedures | **Visits windows** | Patient | No | No | It was discussed with the investigators to instruct patients on the | No | |

| 1 | 2 | 3 | 4 | 5 | 6 | 7 | 8 | 9 | 10 | 11 |
|---|---|---|---|---|---|---|---|---|---|---|
| | | | | | | | | timing of visits in order to avoid deviations. | | |
| 11-3 | 07.12 | 11-17 | Patient 11-17 made visit 7 two days later than the window of the visit envisaged by the schedule of procedures | **Visits windows** | Patient | No | No | It was discussed with the investigators to instruct patients on the timing of visits in order to avoid deviations. | No | |
| 11-3 | 07.12 | 11-01 | Patient 11-01 did not have a bacterial count on Visits 1, 2, 7 and did not have a haemoglobin count on Visit 2 as part of the urinalysis for technical reasons. | **Protocol procedures** | Investigator | No | No | No | No | |
| 11-3 | 07.12 | 11-02 | Patient 11-02 did not have a bacterial count on Visits 1, 2, 4, 7 and did not have a haemoglobin count on Visits 2 and 7 as part of the urinalysis due to technical reasons. | **Protocol procedures** | Investigator | No | No | No | No | |
| 11-3 | 07.12 | 11-03 | Patient 11-03 did not have a bacterial count on Visits 1, 2, 4, 7 and did not have a haemoglobin count on Visits 1 and 7 as part of the urinalysis due to technical reasons. | **Protocol procedures** | Investigator | No | No | No | No | |
| 11-3 | 07.12 | 11-04 | Patient 11-04 did not have a bacterial count on Visits 1, 4, 7 and did not have a haemoglobin count on Visits 1 and 7 as part of the urinalysis due to technical reasons. | **Protocol procedures** | Investigator | No | No | No | No | |
| 11-3 | 07.12 | 11-05 | Patient 11-05 did not have a bacterial count on Visits 2, 6 and did not have a haemoglobin count on Visit 1 as part of the urinalysis due to technical reasons. | **Protocol procedures** | Investigator | No | No | No | No | |

| 1 | 2 | 3 | 4 | 5 | 6 | 7 | 8 | 9 | 10 | 11 |
|---|---|---|---|---|---|---|---|---|---|---|
| 11-3 | 07.12 | 11-06 | Patient 11-06 did not have a bacterial count on Visits 1, 2 and did not have a haemoglobin count on Visit 1 as part of the urinalysis due to technical reasons. | **Protocol procedures** | Investigator | No | No | No | No | |
| 11-3 | 07.12 | 11-07 | Patient 11-07 did not have a bacterial count on Visits 1, 4, 7 and did not have a haemoglobin count on Visits 1, 2 as part of the urinalysis due to technical reasons. | **Protocol procedures** | Investigator | No | No | No | No | |
| 11-3 | 07.12 | 11-08 | Patient 11-08 did not have a bacterial count on Visits 1, 2, 4, 7 and did not have a haemoglobin count on Visits 1, 2 as part of the urinalysis due to technical reasons. | **Protocol procedures** | Investigator | No | No | No | No | |
| 11-3 | 07.12 | 11-09 | Patient 11-09 did not have a bacterial count on Visits 2, 6, 7 and did not have a haemoglobin count on Visit 7 as part of the urinalysis due to technical reasons. | **Protocol procedures** | Investigator | No | No | No | No | |
| 11-3 | 07.12 | 11-10 | Patient 11-10 did not have a bacterial count on Visits 1, 6, 7 and did not have a haemoglobin count on Visit 1 as part of the urinalysis due to technical reasons. | **Protocol procedures** | Investigator | No | No | No | No | |
| 11-3 | 07.12 | 11-11 | Patient 11-11 did not have a bacterial count on Visits 1, 2, 6, 7 and did not have a haemoglobin count on Visit 1 as part of the urinalysis due to technical reasons. | **Protocol procedures** | Investigator | No | No | No | No | |
| 11-3 | 07.12 | 11-12 | Patient 11-12 did not have a bacterial count on Visits 1, 2, 7 and did not have a haemoglobin count on Visit 1 as part of the urinalysis due to technical reasons. | **Protocol procedures** | Investigator | No | No | No | No | |
| 11-3 | 07.12 | 11-13 | Patient 11-13 did not have a bacterial count on Visits 1, 4, 7 and did not have a | **Protocol procedures** | Investigator | No | No | No | No | |

| 1 | 2 | 3 | 4 | 5 | 6 | 7 | 8 | 9 | 10 | 11 |
|---|---|---|---|---|---|---|---|---|---|---|
| | | | haemoglobin count on Visit 1 as part of the urinalysis due to technical reasons. | | | | | | | |
| 11-3 | 07.12 | 11-14 | Patient 11-14 did not have a bacterial and haemoglobin count on Visit 1 as part of the urinalysis due to technical reasons. | **Protocol procedures** | Investigator | No | No | No | No | |
| 11-3 | 07.12 | 11-15 | Patient 11-15 did not have a bacterial and haemoglobin count on Visit 1 as part of the urinalysis due to technical reasons. | **Protocol procedures** | Investigator | No | No | No | No | |
| 11-3 | 07.12 | 11-16 | Patient 11-16 did not have a bacterial count on Visits 2, 7 and did not have a haemoglobin count on Visit 7 as part of the urinalysis due to technical reasons. | **Protocol procedures** | Investigator | No | No | No | No | |
| 11-3 | 07.12 | 11-17 | Patient 11-17 did not have a bacterial count on Visits 1, 2, 6 and did not have a haemoglobin count on Visit 1 as part of the urinalysis due to technical reasons. | **Protocol procedures** | Investigator | No | No | No | No | |
| 11-3 | 07.12 | 11-17 | Patient 11-17 treated with prohibited therapy during the study (dexamethasone 0.3 ml parabulbar) as part of treatment for a serious adverse event | **Other** | Investigator | No | No | The investigators were interviewed about the prohibited therapy in the study | No | |

*__Date of the deviation__: all of the trials in the table were conducted in 2020

**Table S5.** Rate of relative DLCO and FVC changes in mITT and PPS populations as n (%) and primary efficacy outcome evaluation.

| Population | Relative FVC change in **placebo** group | | | | Relative FVC change in **Treamid** group | | | |
|---|---|---|---|---|---|---|---|---|
| | Decrease or no change | Increase: | | | Decrease or no change | Increase: | | |
| | | <5% | 5–10% | ≥10% | | <5% | 5–10% | ≥10% |
| mITT | 14 (47%) | 4 (13%) | 7 (23%) | 5 (17%) | 11 (38%) | 4 (14%) | 7 (24%) | 7 (24%) |
| PPS | 13 (52%) | 3 (12%) | 5 (20%) | 4 (16%) | 10 (40%) | 2 (8%) | 6 (24%) | 7 (28%) |

| Population | Relative DLCO change in **placebo** group | | | Relative DLCO change in **Treamid** group | | |
|---|---|---|---|---|---|---|
| | Decrease or no change | Increase: | | Decrease or no change | Increase: | |
| | | <15% | ≥15% | | <15% | ≥15% |
| mITT | 7 (23.3%) | 19 (63.3%) | 4 (13.3%) | 8 (28%) | 11 (38%) | 10 (34%) |
| PPS | 7 (28%) | 14 (56%) | 4 (16%) | 6 (24%) | 10 (40%) | 9 (36%) |

| Population | Primary efficacy outcome (≥10% increase in FVC or [5–10% increase in FVC and ≥15% increase in DLCO]) | |
|---|---|---|
| mITT | **Placebo**: 5 out of 30 (17%) | **Treamid**: 12 out of 29 (41%) |
| | p-value = 0.036 ($\chi^2$) | |
| PPS | **Placebo**: 4 out of 25 (16%) | **Treamid**: 11 out of 25 (44%) |
| | p-value = 0.031 ($\chi^2$) | |

**References**


1. Brooks S.M. Task group on surveillance for respiratory hazards in the occupational setting. Surveillance for respiratory hazards. ATS News 1982; 8: 12-16.
2. Am Rev Respir Dis. 126(5), Burdon, et al. The perception of breathlessness in asthma, 825–828; Copyright (1982)
3. Patel, Amit S., et al. The development and validation of the King's Brief Interstitial Lung Disease (K-BILD) health status questionnaire. *Thorax* 67.9 (2012): 804-810.
4. Salorinne Y. Single breath pulmonary diffusing capacity; Reference values and application in connective tissue diseases and in various lung diseases. Scand J Respir Dis 56:165. 1975.
5. Hankinson JL, Odencrantz JR, Fedan KB. Spirometric reference values from a sample of the general US population. *Am J Respir Crit Care Med.* 1999;159(1):179-87.
6. Hankinson JL, Odencrantz JR, Fedan KB. Spirometric reference values from a sample of the general US population. *Am J Respir Crit Care Med.* 1999;159(1):179-87.
7. Respiration 2015;89:226–234.
8. Quanjer PH, Tammeling GJ, Cotes JE, Pedersen OF, Peslin R, Yernault JC. Lung volumes and forced ventilatory flows. *Eur Respir J.* 1993;6(Suppl 16):5–40
